# Enantioselective benzylic C–H arylation via photoredox and nickel dual catalysis

**DOI:** 10.1038/s41467-019-11392-6

**Published:** 2019-08-07

**Authors:** Xiaokai Cheng, Huangzhe Lu, Zhan Lu

**Affiliations:** Department of Chemistry, Zhejiang University, 310058 Hangzhou, China

**Keywords:** Asymmetric catalysis, Synthetic chemistry methodology, Photocatalysis

## Abstract

The asymmetric cross-coupling reaction is developed as a straightforward strategy toward 1,1-diaryl alkanes, which are a key skeleton in a series of natural products and bioactive molecules in recent years. Here we report an enantioselective benzylic C(sp^3^)−H bond arylation via photoredox/nickel dual catalysis. Sterically hindered chiral biimidazoline ligands are designed for this asymmetric cross-coupling reaction. Readily available alkyl benzenes and aryl bromides with various functional groups tolerance can be easily and directly transferred to useful chiral 1,1-diaryl alkanes including pharmaceutical intermediates and bioactive molecules. This reaction proceeds smoothly under mild conditions without the use of external redox reagents.

## Abstract (web summary)

Chiral 1,1-diaryl alkanes are important targets in pharmaceutical industry. Here, the authors report report a redox-neutral enantioselective benzylic C−H bond arylation via photoredox/nickel dual catalysis accessing chiral 1,1-diarylalkane compounds under mild conditions.

## Introduction

**Fig. 1:**
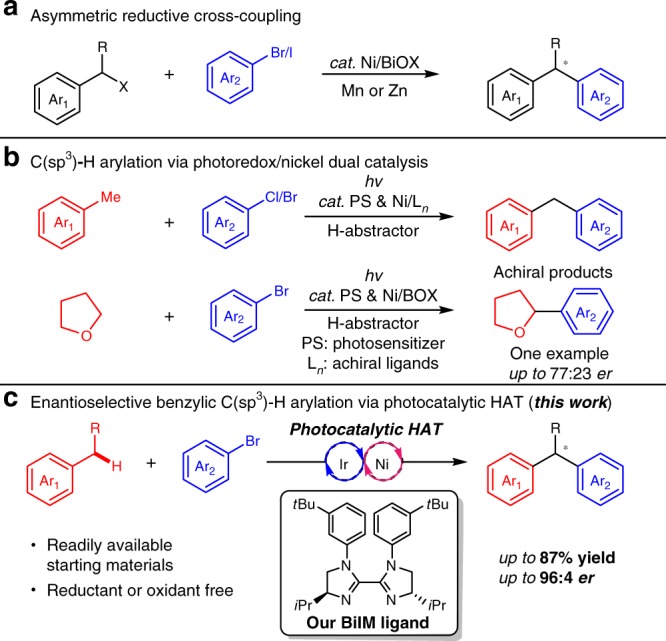
Strategies for nickel-catalyzed asymmetric arylation of benzylic position or C–H bond reactions. **a** Asymmetric reductive cross-coupling strategies toward 1,1-diaryl alkanes with BiOX ligands. **b** Achiral C(sp^3^)−H arylation via photo/nickel dual catalysis and example for the asymmetric form. **c** The enantioselective benzylic C(*sp*^3^)−H arylation based on designed biimidazoline ligand

Enantioenriched 1,1-diaryl alkanes are a key skeleton in a series of natural products and bio-active molecules, such as sertraline^1^, tolterodine^2,3^, podophyllotoxins^4^, etc^5–8^. Due to the broad application of 1,1-diaryl alkanes in pharmaceutical industry, their asymmetric synthesis has attracted intensive interests in organic chemistry community and multiple strategies have been developed^9–17^. As a highly efficient and direct methodology for generating stereogenic centers in target molecules, transition-metal-catalyzed enantioselective cross-coupling reactions of electrophiles with organometallic reagents have been developed by Fu and colleagues^18^, and Molander and colleagues^19,20^ to furnish 1,1-diaryl alkanes using chiral bioxazolines (BiOX) as ligands. In addition, stereospecific cross-coupling reactions could also deliver this class of compounds^21–25^. Recently, nickel-catalyzed asymmetric reductive cross-coupling strategies of racemic benzylic electrophiles with aryl halides were reported by Weix and colleagues^26^, Reisman and colleagues^27^, Sigman and Doyle^28^ to provide an alternative strategy using chiral BiOXs as ligands and stoichiometric reductive transition metals (Fig. 1a). Compared with the well-established methologies with alkenes or electrophiles, using alkane as a substrate, the direct C–H arylation is considered a preferable step- and atom-economic method for the construction of C(*sp*^3^)–C(*sp*^2^) bonds^29–38^. During the preparation of this manuscript, the Cu/BOX-catalyzed radical relay strategy was used by Liu and colleagues^39^ to realize an elegant enantioselective arylation of C–H bonds on a methylene group adjacent to a naphthalene moiety^40^. By the merge of photocatalysis and transition-metal catalysis^20,41–56^, the milestone of C–H arylation reactions via hydrogen atom transfer (HAT) process has been recently marked by Molander and colleagues^57^, Shields and Doyle^58^, MacMillan and colleagues^59^, and Martin and colleagues^60^ (Fig. 1b) to provide an alternative for the direct construction of 1,1-diaryl alkanes with readily available starting materials in a mild reaction condition. However, by lack of development of ligands able to differentiate between competing diastereomeric transition states, asymmetric cross-coupling reaction via this photocatalytic HAT process is quite challenging. So far, the best enantioselectivity of C–H arylation via photoredox/nickel dual catalysis is 77:23 enantiometric ratio (*er*)^60^. Our researches focus on asymmetric earth-abundant transition metal catalysis via chiral ligand design^61–66^. It is noted that the oxazoline derivated chiral ligands (BOX or BiOX), which have been well established in the cross-coupling strategies toward 1,1-diaryl alkanes, performed unsatisfactorily in controlling enantioselectivity in the visible-light-induced C–H arylation methology^60^. Thus, an effective chiral ligand is to be discovered for the enantioselective construction of 1,1-diaryl alkanes under photoredox/nickel dual catalysis.

Here we report the enantioselective benzylic C–H arylation of readily available alkyl benzene with commercially available aryl bromides by using our designed chiral biimidazoline (BiIM) ligand (Fig. 1c). In addition, this protocol is redox neutral without using any additional single-electron oxidant or reductant.

## Results

### Reaction optimization

At the beginning of our study, the reaction of ethyl benzene **1a** with methyl 4-bromobenzoate **2a** using iridium photocatalyst with bis(4-methoxyphenyl)methanone (DMBP) as a co-photocatalyst^57^ promoting the yield of **3aa** (see Supplementary Table 3) under the irradiation of blue LEDs, and nickel dichloride-dimethoxyethane complex and chiral ligand as the cross-coupling catalyst in the presence of K_2_HPO_4_ as a base in a solution of dioxane/ethyl benzene was chosen as a model reaction (Table 1). Inspired by previous reports on nickel-catalyzed asymmetric cross-coupling reactions using chiral BiOX ligand, we were so excited to find that the chiral BiOX ligands **LS1** could accelerate the reaction to deliver **3aa** in 79% yield, however, with a moderate enantiomeric ratio (69:31 *er*) (Table 1, entry 1). The more electron-rich chiral BiIM ligands^67^ were then applied as an alternative for the improvement of enantioselectivity due to the easy modification of electronic and steric effects. The reaction using *N*-isopropyl protected *N*-*i*PrBiIM (**LS2**) as a ligand afforded **3aa** in 33% yield and 62.5:37.5 *er* (Table 1, entry 2). To our delight, when the *N*-aryl BiIM ligand **L1a** was used as a ligand, the reaction afforded the products **3aa** in 44% yield with 92.5:7.5 *er* (Table 1, entry 3). The steric hindrance and possible *π*–*π* effect of the phenyl group on nitrogen atom increased the inflexibility of the BiIM, which might improve the enantioselectivity^65,66^. The homocoupling product from ethylbenzene was also observed, which illustrated that the reaction might undergo radical pathway. After screening various substitution effects on BiIM ligands (Table 1, entries 4–7), the sterically hinder *N*-3-*t*BuPh-*i*PrBiIM ligand (**L1e**) was designed as the best ligand that delivered **3aa** in 44% yield and 95:5 *er*. When the reaction time was extended to 34 h, the reaction afforded **3aa** in 62% yield with 94.5:5.5 *er*, which was established as standard conditions A (Table 1, entry 8). The reaction using 4.0 equivalent of ethyl benzene for 96 h afforded **3aa** in 62% yield with a slightly lower *er* (92.5:7.5), which was established as standard conditions B (Table 1, entry 9). Control experiments (see Supplementary Table 3, entries 1–3) indicated that the iridium photocatalyst, nickel complex, and light was essential. Reactions were demonstrated to occur successfully with a lower yield in the absence of DMBP. In addition, the mixed solvent of dioxane and ethylbenzene is proved effective by inhibiting homocoupling of ethylbenzne (see Supplementary Table 3, entry 7)

**Table 1:** Selected chiral ligand screening results^a^


Entry	Ln	Yield of 3aa (%)	*er*
1	**LS1**	79	69:31
2	**LS2**	33	62.5:37.5
3	**L1a**	44	92.5:7.5
4	**L1b**	62	86:14
5	**L1c**	35	92:8
6	**L1d**	44	92.5:7.5
7	**L1e**	44	95:5
8^*b*^	**L1e**	62	94.5:5.5
9^*c*^	**L1e**	62	92.5:7.5


^a^General reaction conditions: **1a** (1.0 mL), **2a** (0.2 mmol), Ir(dFCF_3_ppy)_2_(dtbbpy)Cl (2.2 mol%), NiCl_2_•DME (20 mol%), Ln (20 mol%), DMBP (25 mol%), and K_2_HPO_4_ (2.0 equiv.) in dioxane (3 mL) under the irradiation of 8 W blue LEDs for 24 h. Yields determined by ^1^H-NMR using TMSPh as an internal standard. Enantiometric ratio (*er*) determined by chiral HPLC. ^b^Run for 34 h. ^c^Using **1a** (0.8 mmol) for 96 h

### Substrate scope

**Fig. 2:**
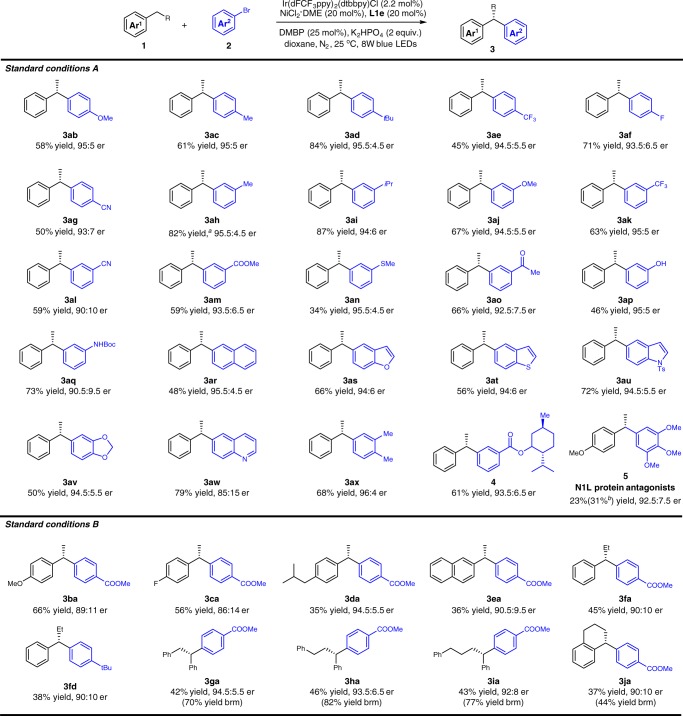
Substrate scope. Standard conditions A: **1** (1.0 mL), **2** (0.2 mmol), Ir(dFCF_3_ppy)_2_(dtbbpy)Cl (2.2 mol%), NiCl_2_·DME (20 mol%), **L1e** (20 mol%), DMBP (25 mol%), and K_2_HPO_4_ (2.0 eq.) in dioxane (3 mL) under the irradiation of 8 W blue LEDs for 34 h. Standard conditions B: **1** (0.8 mmol), **2** (0.2 mmol), Ir(dFCF_3_ppy)_2_(dtbbpy)Cl (2.2 mol%), NiCl_2_·DME (20 mol%), **L1e** (20 mol%), DMBP (25 mol%), and K_2_HPO_4_ (2.0 eq.) in dioxane (3 mL) under the irradiation of 8 W blue LEDs for 96 h. Isolated yield, the er was determined by HPLC. ^a^For 48 h. ^b^NMR yield using TMSPh as an internal standard

With optimized conditions in hands, we explored the substrate scope of the reaction with both aryl bromides and alkanes. As shown in Fig. 2, under standard conditions, the visible-light-induced asymmetric C−H arylation of ethyl benzene underwent smoothly with various coupling partners. Aryl bromides with either electron-donating (**3ab**–**3ad**) or electron-withdrawing functional groups (**3ae**–**3ag**) at the *para*-position were suitable in this reaction, delivering the corresponding chiral 1,1-diaryl ethanes in 45–84% yields with 93:7 to 95.5:4.5 *ers* (**3ab**–**3ag**). The reaction of aryl bromides with *meta*-substituents such as methyl and isopropyl groups gave **3ah** and **3ai** in 82% and 87% yields with 95.5:4.5 and 94:6 *er*. It is worth noting that 1° or 3° benzylic C(*sp*^3^)−H bonds could be differentiated, as only secondary benzylic C(*sp*^3^)−H bonds were directly activated under these conditions^68^. Various functional groups, such as methoxyl, trifluoromethyl, cyano, ester, thio ether, aceto, hydroxy, and Boc-protected amino groups, were well tolerated (**3aj**–**3aq**). The polycyclic rings and heterocycles such as 2-naphenyl, 5-benzothiophyl, 5-benzofuranyl, 5-indyl, 6-quinolyl, and 1,3-benzodioxole substrates could be delivered to corresponding products (**3ar**–**3aw**) in 48–79% yields with up to 95.5:4.5 *er*. The coupling of ethyl benzene and 3,4-dimethyl benzyl bromide could give **3ax** in 68% yield with 96:4 *er*.

For the substituted benzenes, ethyl benzene with methoxyl, flouro, alkyl groups, and 2-ethylnaphthalene also serve as effective substrates in asymmetric benzylic C(*sp*^3^)−H arylation under standard conditions B to convert to **3ba**–**3ca** in moderate yields with 86:14 to 89:11 *ers*. It should be noted that the chemoselective benzylic C–H arylation of the ethyl group rather than the isobutyl group on 1-ethyl-4-isobutylbenzene (**1d**) proceeded to deliver **3da** in 35% yield and 94.5:5.5 *er*. The propyl and butyl benzenes were also used to give the corresponding arylation products (**3fa**, **3fd**) in moderate yields with 90:10 *er*. The 1,2-diphenylethane, 1,3-diphenylpropane, and 1,4-diphenylbutane were mono-activated, providing 1,1,*x* (*x* = 2,3,4) triaryl alkanes in 42–46% yields with 92:8 to 94.5:5.5 *ers*. The asymmetric arylation of cyclic substrate **1j** performed smoothly to afford **3ja** in 37% yield with 90:10 *er*. Although low yields were observed in some cases under standard conditions B, the mass balances of alkyl benzenes were mostly quantitative.

The application of this protocol was also investigated by using readily available alkyl benzenes. A Menthol-derived substrate could be utilized to deliver **4** in 61% yield with good *er*. This strategy was also available in the synthesis of pharmaceutical active molecules such as compound **5**, which was reported as a N1L protein (potent vaccinia and variola (smallpox) virulence factor) antagonists^8^.

### Mechanistic studies

**Fig. 3:**
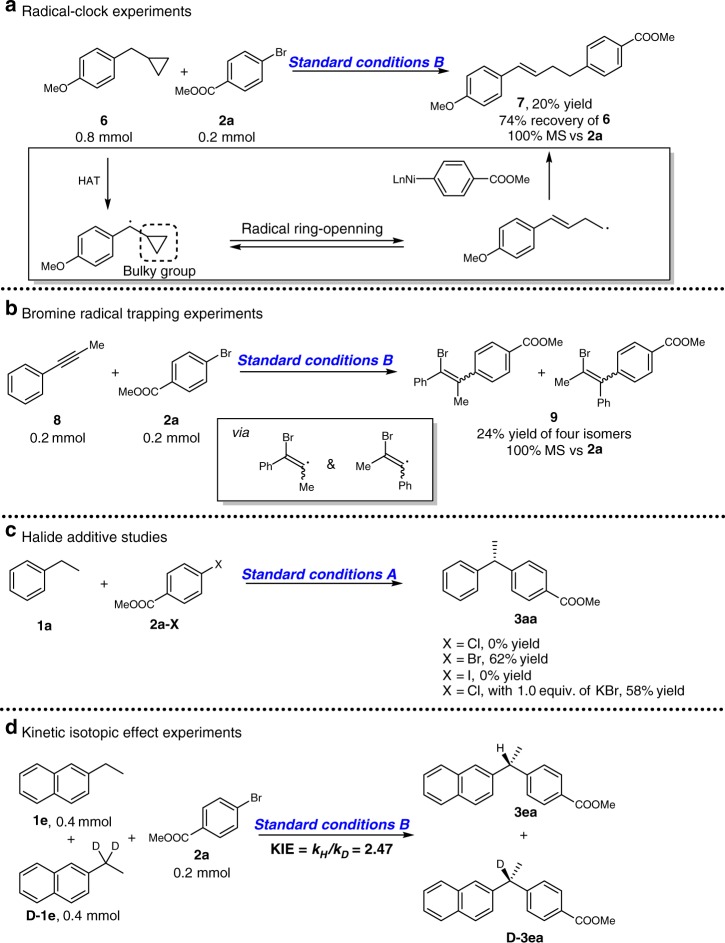
Mechanistic studies. **a** Radical-clock experiments consistent with the existence of benzylic radicals. **b** Compound **8** was added in the absence of ethylbenzene **1a** for trapping bromine-free radical under conditions B. **c** Halide additive studies. **d** Kinetic isotopic effect was evaluated with **1e** and deuterate **1e** as substrate, indicating HAT process might be the turnover-limiting step

Several experiments were designed to figure out the reaction process. The observation of homocoupling byproduct is consistent with the existence of benzylic radical. The reaction of 1-(cyclopropylmethyl)-4-methoxybenzene **6** as a radical clock afforded **7** in 20% yield and 100% mass balance vs. **2a** through a radical-ring-opening process followed by a irreversible capture by nickel species^27^, which strengthened the possibility on radical pathway (Fig. 3a). Yet, we cannot exclude the possibility of a β-carbon elimination pathway to afford the same product. The phenyl methyl ethyne **8** was used as a bromine atom-trapping agent under the standard conditions to afford a mixture of bromo-substituted alkenes **9** in 24% yield and 100% mass balance vs. **2a**, which illustrated the existence of bromine free radical and aryl-nickel bromide species (Fig. 3b). The halide additive studies (Fig. 3c, also see Supplementary Table 3) with aryl chloride **2a-Cl** or aryl iodide **2a-I** could not afford **3aa** under standard conditions A. 1.0 equivalent of KBr was added in the reaction of **2a-Cl** to initiate nickel bromide by halide exchange affording **3aa** with 58% yield. This is also an evidence for the bromine radical initiating HAT of benzylic C−H bond. The deuterium experiment (Fig. 3d) using a 1:1 mixture of **1e** and **D-1e** was carried out and kinetic isotope effect (KIE) was 2.47, which indicated that H-atom abstraction might be the turnover limiting step. Kinetic experiments of ethylbenzene and 4-*t*Bu-phenyl bromide (see Supplementary Figs. 145-147) illustrated zero order on the concentration of aryl bromide and first order on the concentration of ethyl benzene, which were alternative evidences of turnover-limiting HAT process.

**Fig. 4:**
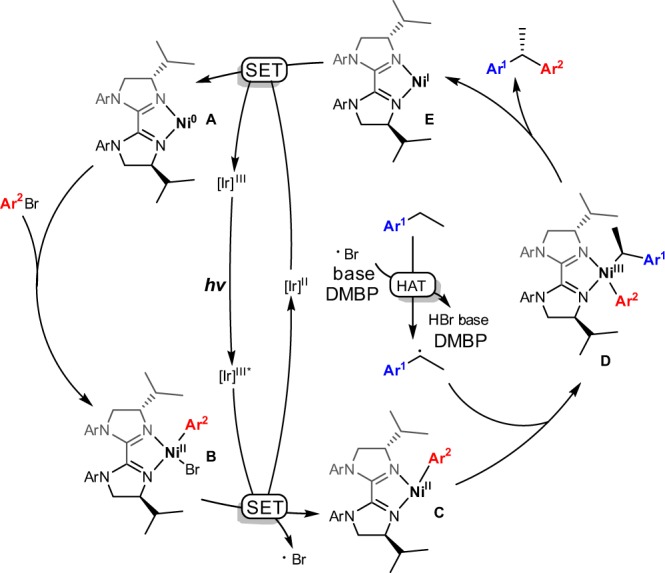
Proposed mechanism. Visible light induced HAT by in-situ-generated bromine radical followed by the asymmetric cross-coupling to 1,1-diaryl alkanes

Based on mechanistic studies (also see Supplementary Discussion section in Supplementary Information) and previously reported literatures^56–60^, the proposed mechanism was shown in Fig. 4. The in-situ generated Ni(0) complex **A** could undergo oxidative addition with aryl bromide to generate aryl Ni(II) bromide species **B**, which could undergo visible-light-induced single-electron oxidation to give aryl Ni(II) species **C** and active bromine atom.

Simultaneously, the photoexcited iridium complex was reduced to iridium(II) species. Yet, we cannot rule out the mechanism of ET process in the initiation of bromine-free radical from nickel-aryl adduct. The HAT process occurred between bromine-free radical (BDE (Bond Dissociation Energies) of H−Br is 366 kJ/mol)^69^ and alkyl benzene (BDE of benzylic C−H bond of ethylbenzene is 357 kJ/mol)^69^ rather than between bromine and dioxane (BDE of the C−H bond of dioxane is 406 kJ/mol)^70^ using DMBP as co-catalyst to deliver benzylic radical which was trapped by aryl Ni(II) species **C** to afford Ni(III) complex **D**. The reductive elimination of Ni(III) complex **D** could afford the chiral 1,1-diaryl alkanes and produce Ni(I) complex **E**, which could undergo single-electron reduction by iridium(II) species to regenerate Ni(0) species **A** and photocatalyst iridium(III) complex.

### Conclusion

A direct enantioselective benzylic C−H arylation under photoredox/nickel dual catalysis was reported with a broad substrate scope and good level of enantioselectivity. This protocol provides an effective method for the asymmetric synthesis of 1,1-diaryl alkanes with preferable step- and atom economy. In addition, this protocol is redox neutral without using any additional single-electron oxidant or reductant. Furthermore, this method could be applied for the synthesis of pharmaceutical molecules and modification of complex compounds. A primary mechanism was proposed based on the previously reported literatures and mechanistic studies. Further studies on enantioselective C−H functionalization with photocatalysis are undergoing in our laboratory.

## Methods

### Materials

For NMR spectra of compounds in this manuscript, see Supplementary Figs. 1–107. For HPLC spectra of compounds in this manuscript, see Supplementary Figures 108-144. For the optimization of reaction conditions, see Supplementary Tables 1, 2. For control experiments, see Supplementary Table 3. For kinetic experiments, see Supplementary Figs. 145–147 and Supplementary Tables 4, 5. For radical-clock experiment, bromine radical-trapping experiment, KIE experiment, and catalytic active species experiment, see Supplementary Figs. 148–155. For the experimental procedures and analytic data of compounds synthesized, see Supplementary Methods.

### Standard conditions A for chiral 1,1-diaryl alkanes

To a 20 mL vial with a stir bar was added **L1e** (0.04 mmol), NiCl_2_•DME (0.04 mmol) and 1 mL of dioxane in a N_2_-filled glovebox. The reaction was stirred at 50 °C for 30 min before cooling to room temperature. Dioxane (2 mL), **1** (1 mL), benzyl bromide **2** (0.2 mmol), Ir(dFCF_3_ppy)_2_(dtbbpy)Cl (0.0044 mmol), DMBP (0.05 mmol), and K_2_HPO_4_ (0.4 mmol) was added consistently. The vial was sealed with a Teflon cap and then allowed to remove from the glovebox. The reaction was stirred at 600 r.p.m. under the irradiation of 8 W blue LEDs in a distance of 5 cm at room temperature (25 °C) for 34 h. The reaction was quenched by adding Et_2_O, filtered through a short pad of silica, and eluted with Et_2_O. The solution was concentrated under reduced pressure to afford the crude residue, which was purified by flash column chromatography.

### Standard conditions B for chiral 1,1-diaryl alkanes

To a 20 mL vial with a stir bar was added **L1e** (0.04 mmol), NiCl_2_•DME (0.04 mmol), and 1 mL of dioxane in a N_2_-filled glovebox. The reaction was stirred at 50 °C for 30 min before cooled to room temperature. Dioxane (3 mL), **1** (0.8 mmol), benzyl bromide **2** (0.2 mmol), Ir(dFCF_3_ppy)_2_(dtbbpy)Cl (0.0044 mmol), DMBP (0.05 mmol), and K_2_HPO_4_ (0.4 mmol) was added consistently. The vial was sealed with a Teflon cap and then allowed to remove from the glovebox. The reaction was stirred at 600 r.p.m. under the irradiation of 8 W blue LEDs in a distance of 5 cm at room temperature (25 °C) for 96 h. The reaction was quenched by adding Et_2_O, filtered through a short pad of silica, and eluted with Et_2_O. The solution was concentrated under reduced pressure to afford the crude residue, which was purified by flash column chromatography.

## Supplementary information

Supplementary Information

## Data Availability

The authors declare that the data supporting the findings of this study are available within the paper and its Supplementary Information file, and from the corresponding authors upon reasonable request. The experimental procedures and characterization of all new compounds are provided in the Supplementary Information.
