# Supplementary material for: Enantioselective benzylic C–H arylation via photoredox and nickel dual catalysis
Source: Nat Commun. 2019 Aug 7;10:3549. doi: 10.1038/s41467-019-11392-6 (PMC6685991; doi:10.1038/s41467-019-11392-6)
Supplement: Supplementary file 1 — Supplementary Information [file 41467_2019_11392_MOESM1_ESM.pdf]

Supplementary Information

Enantioselective Benzylic C–H Arylation via Photoredox and Nickel Dual Catalysis

*Xiaokai Cheng, Huangzhe Lu, and Zhan Lu**

Department of Chemistry, Zhejiang University, Hangzhou 310058, China

Supplementary Figures

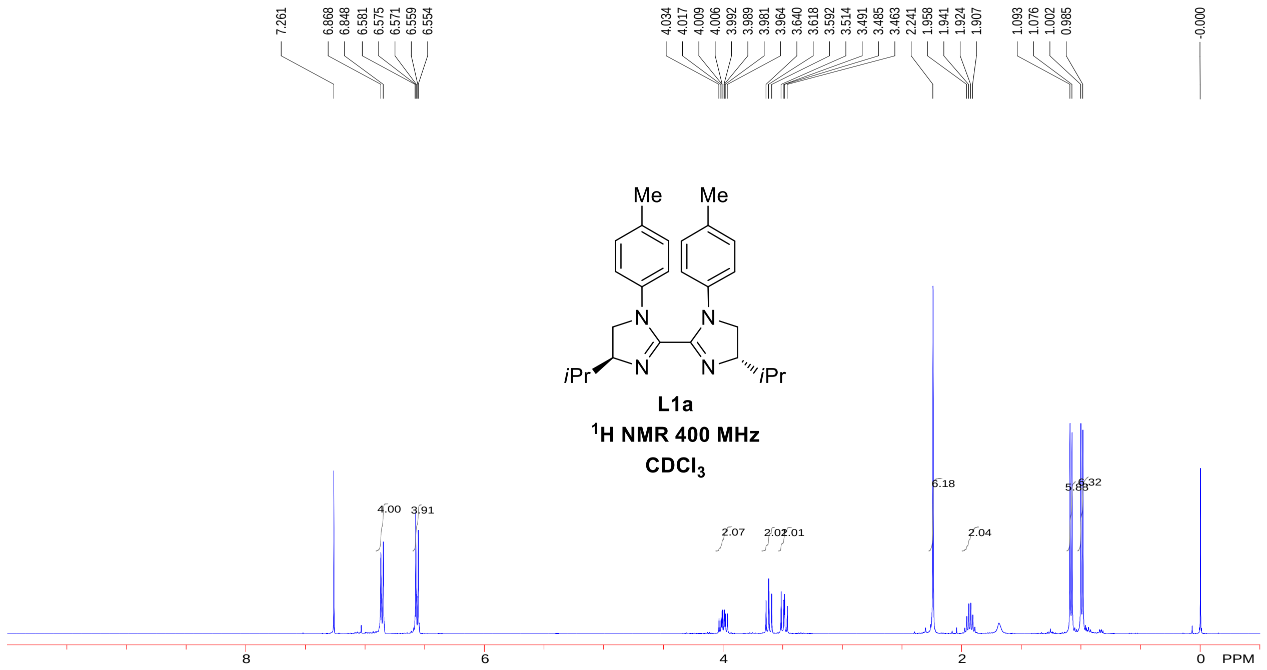

Supplementary Figure 1. ^{1}H NMR spectrum for L1a

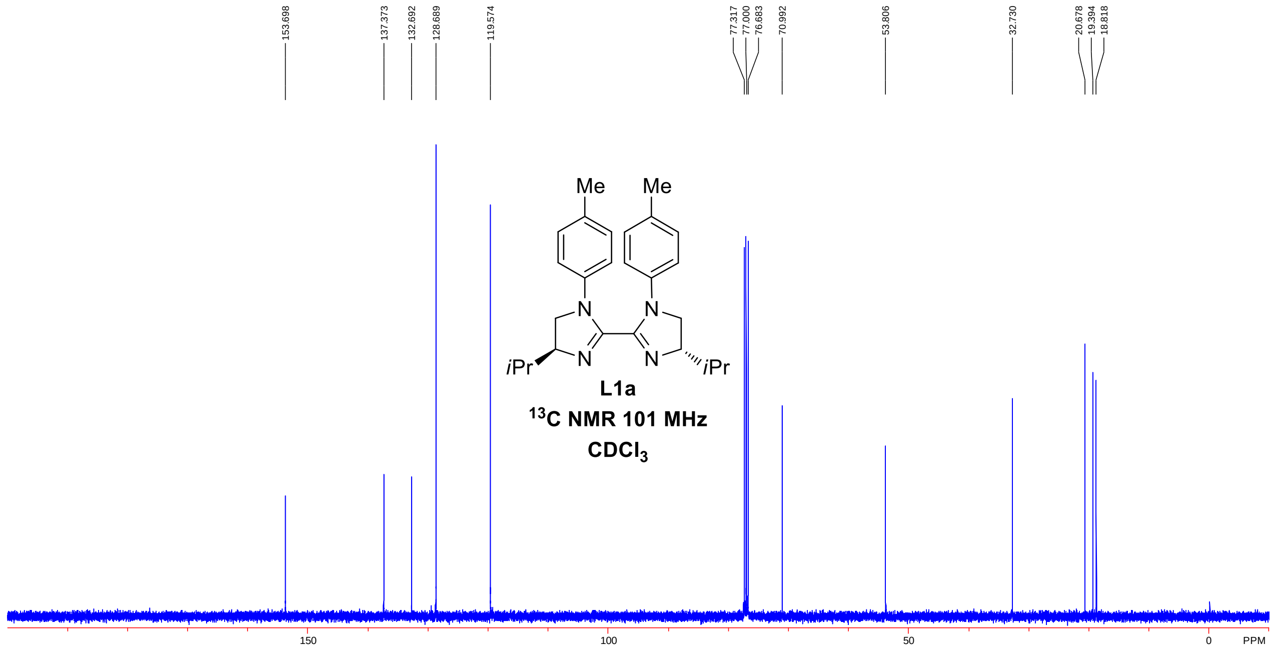

Supplementary Figure 2. ^{13}C NMR spectrum for L1a

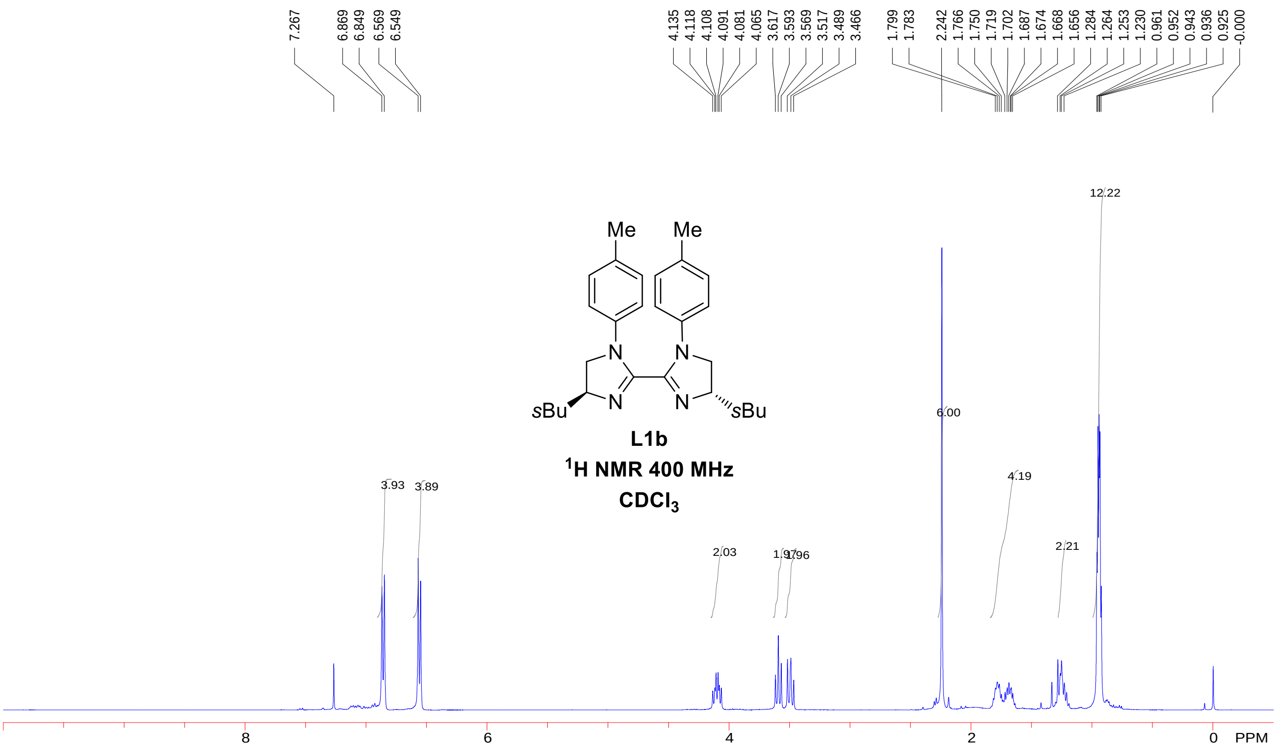

Supplementary Figure 3. ^{1}H NMR spectrum for L1b

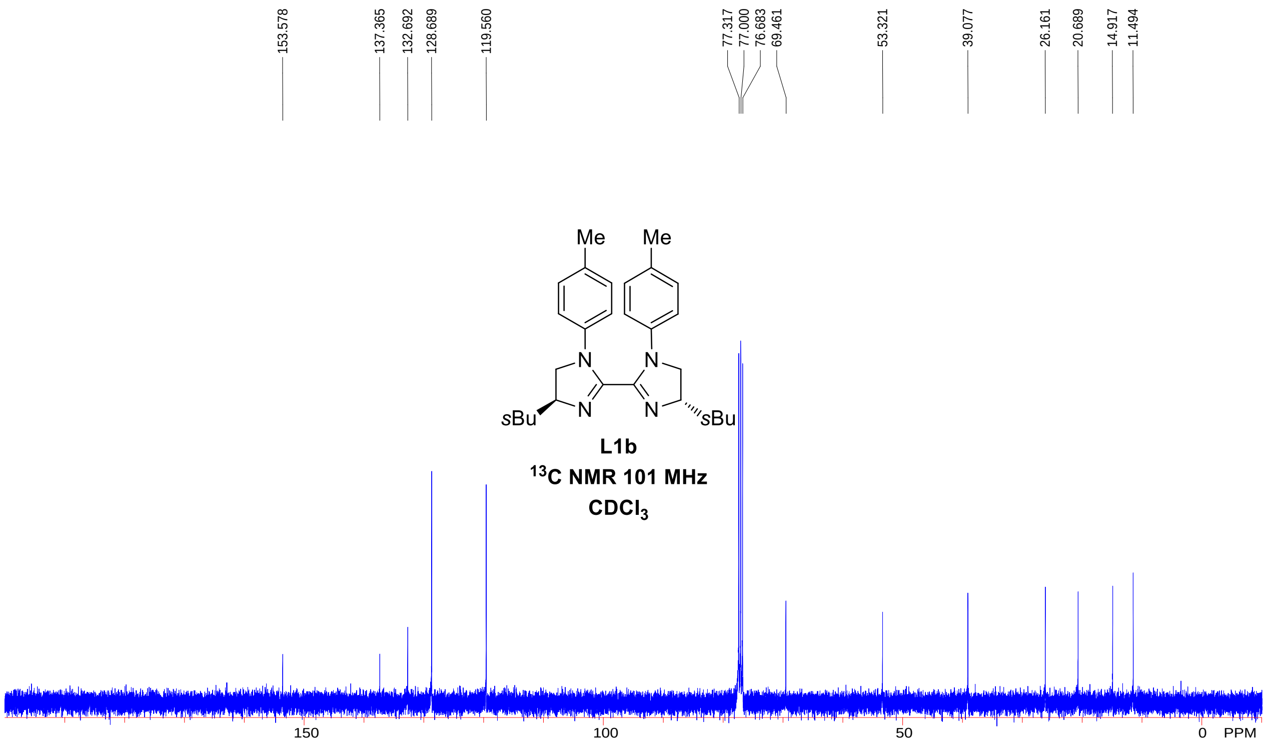

Supplementary Figure 4. ^{13}C NMR spectrum for L1b

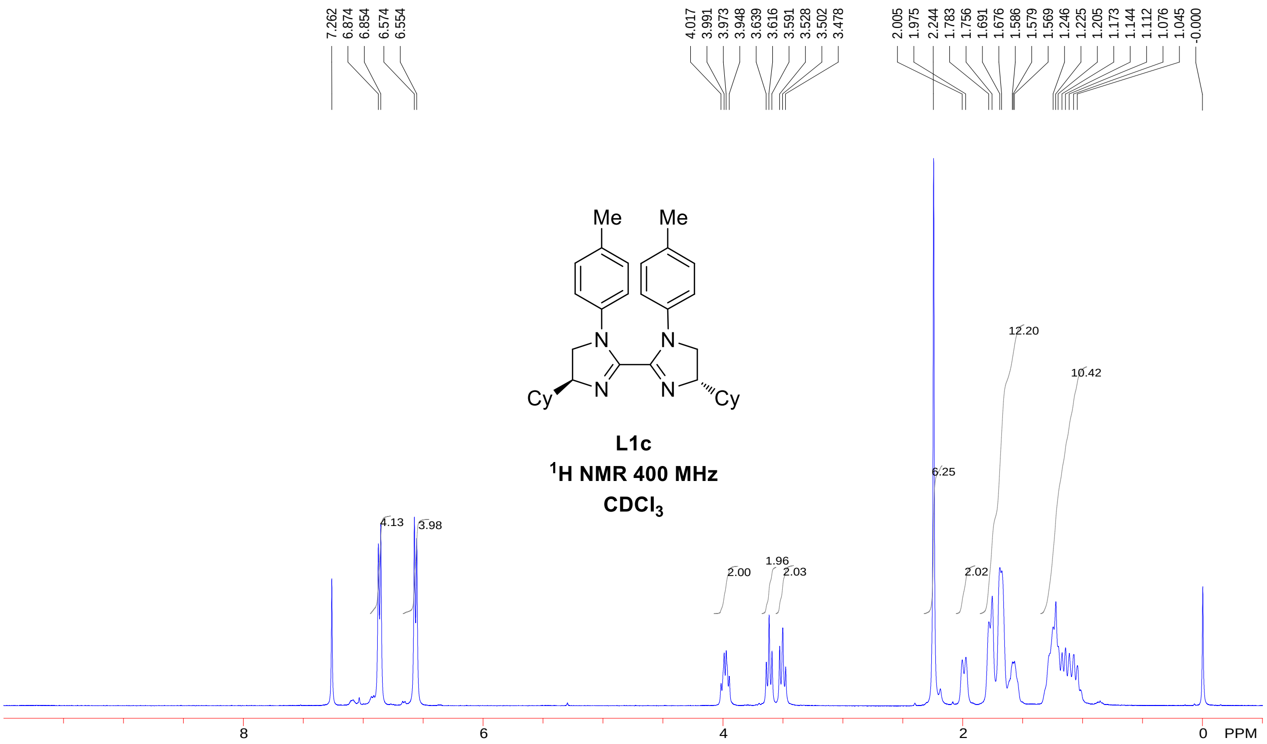

Supplementary Figure 5. ^{1}H NMR spectrum for L1c

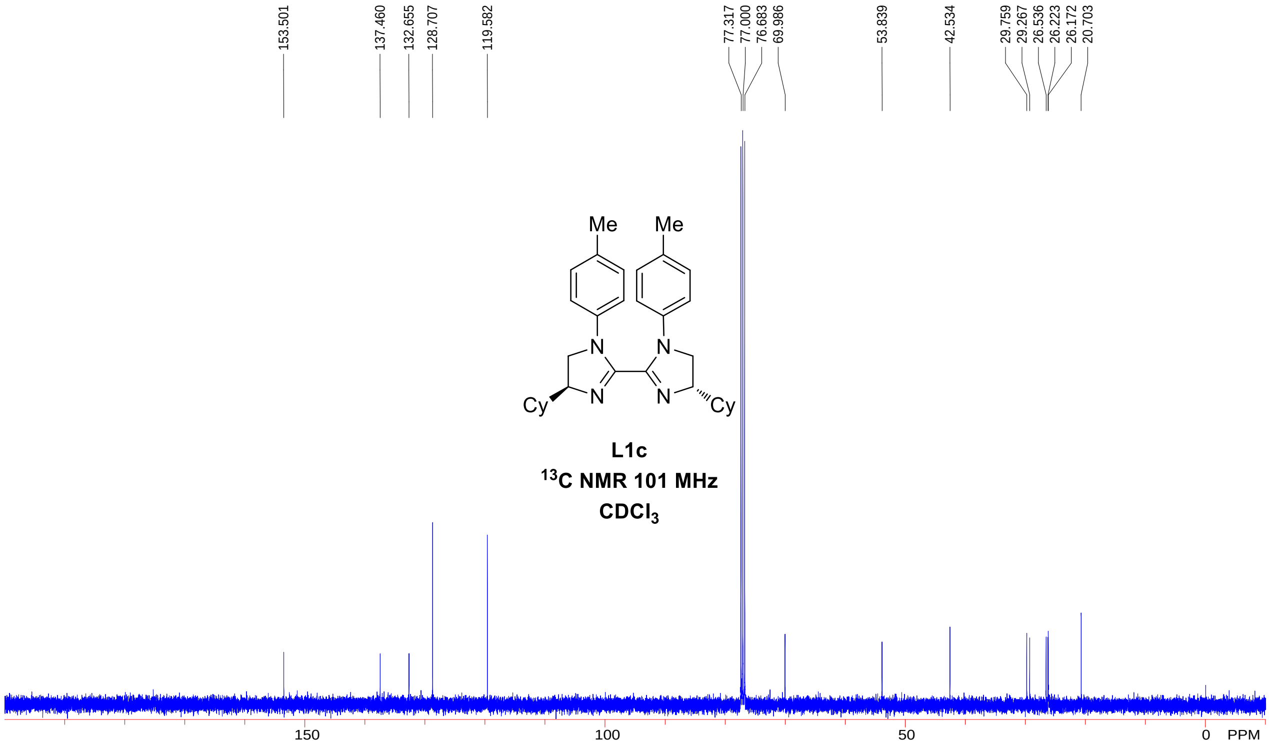

Supplementary Figure 6. ^{13}C NMR spectrum for L1c

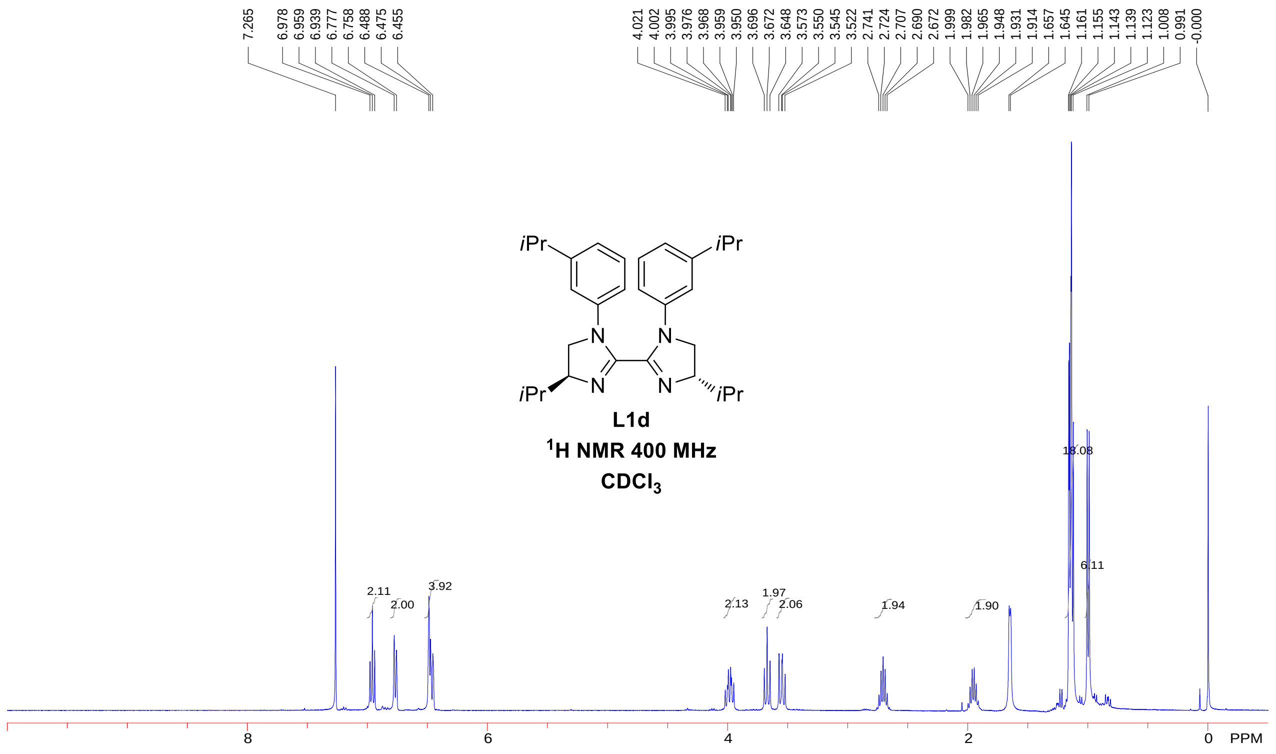

Supplementary Figure 7. ^{1}H NMR spectrum for L1d

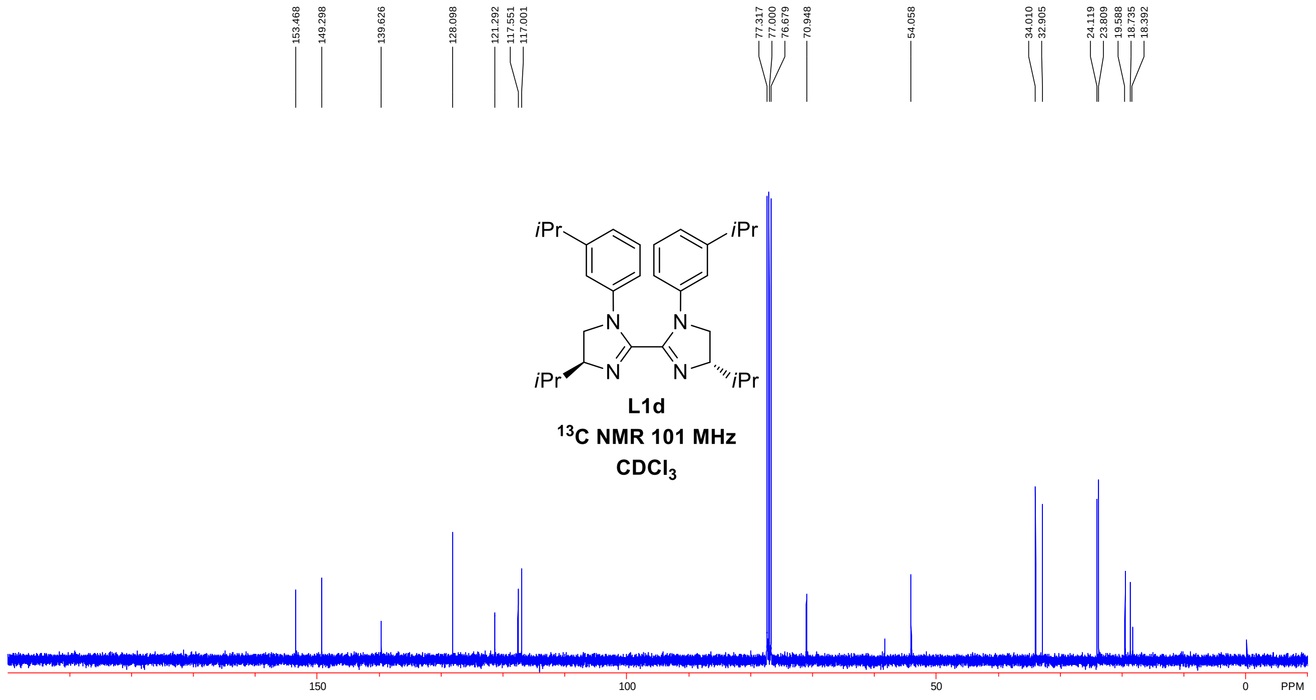

Supplementary Figure 8. ^{13}C NMR spectrum for L1d

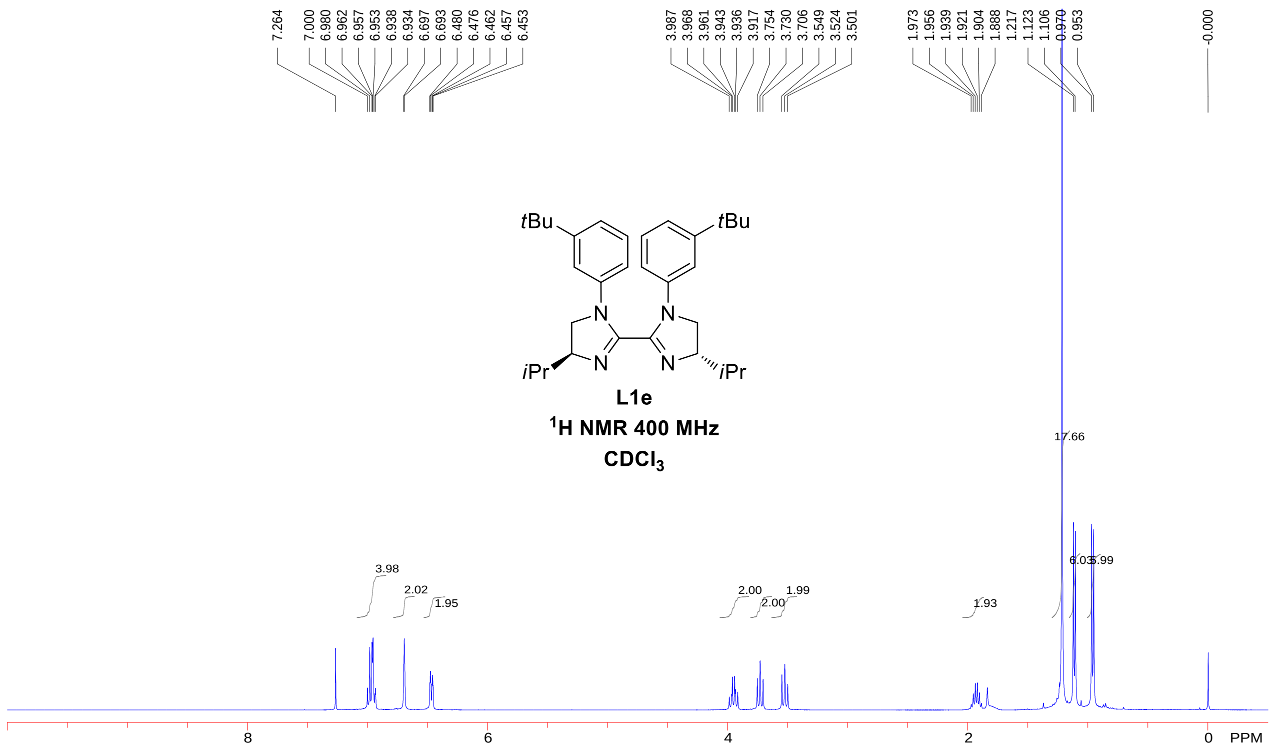

Supplementary Figure 9. ^{1}H NMR spectrum for L1e

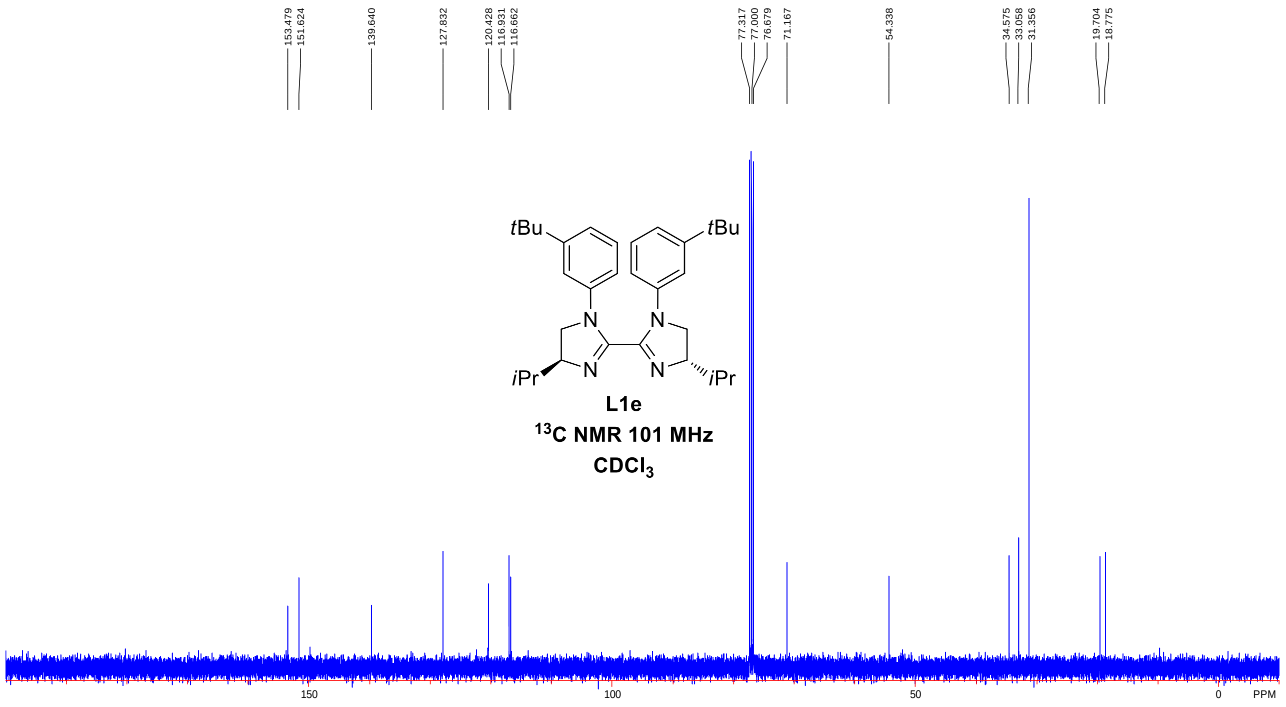

Supplementary Figure 10. ^{13}C NMR spectrum for L1e

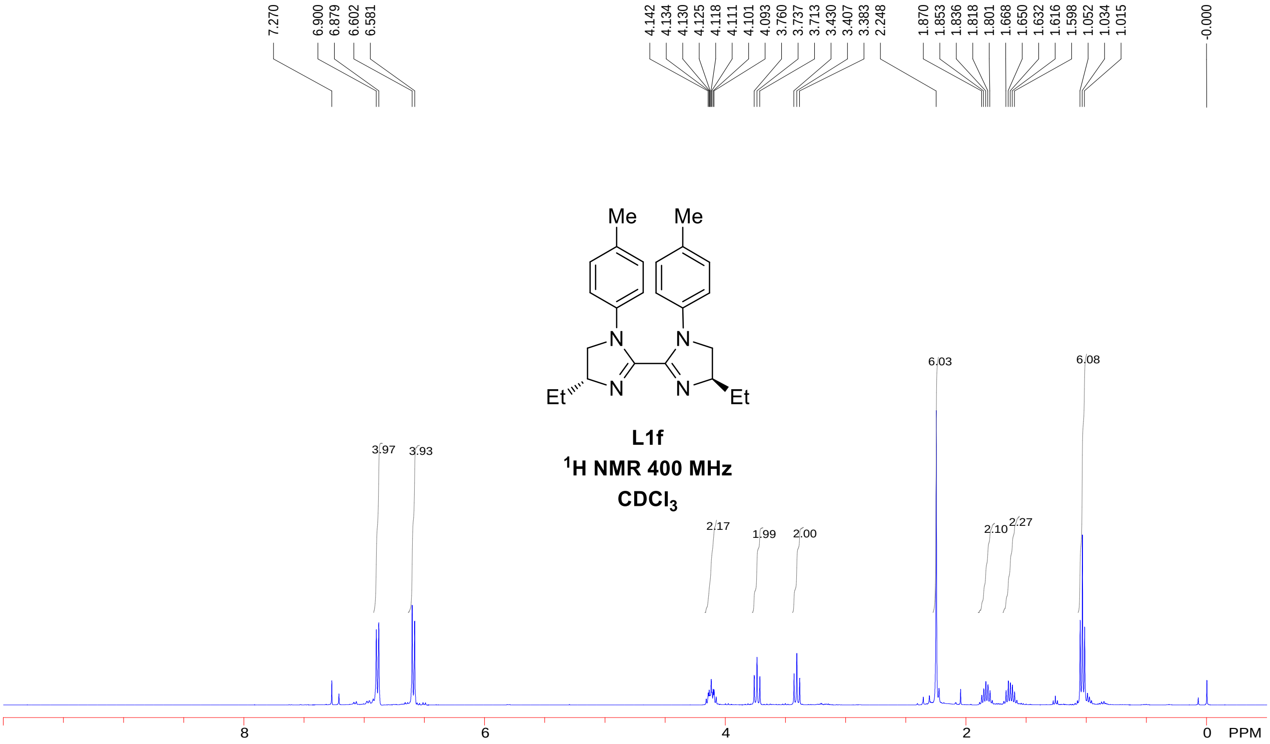

Supplementary Figure 11. ^{1}H NMR spectrum for L1f

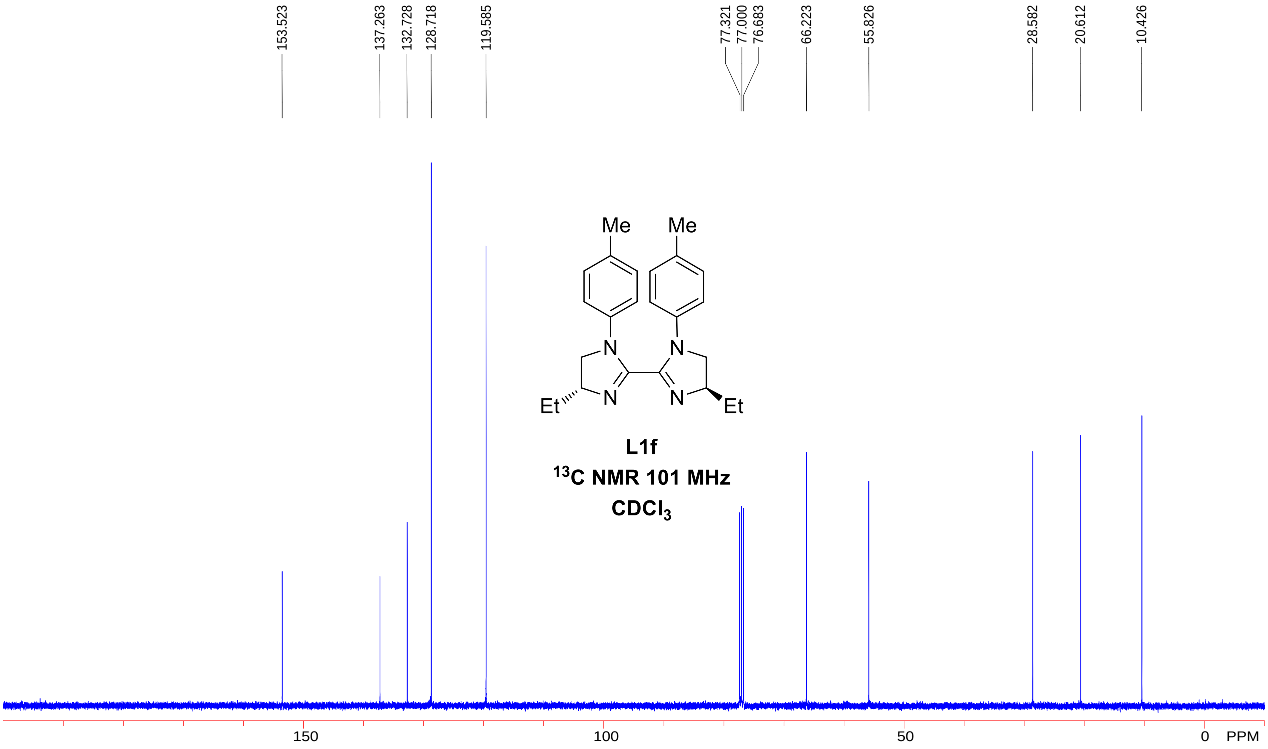

Supplementary Figure 12. ^{13}C NMR spectrum for L1f

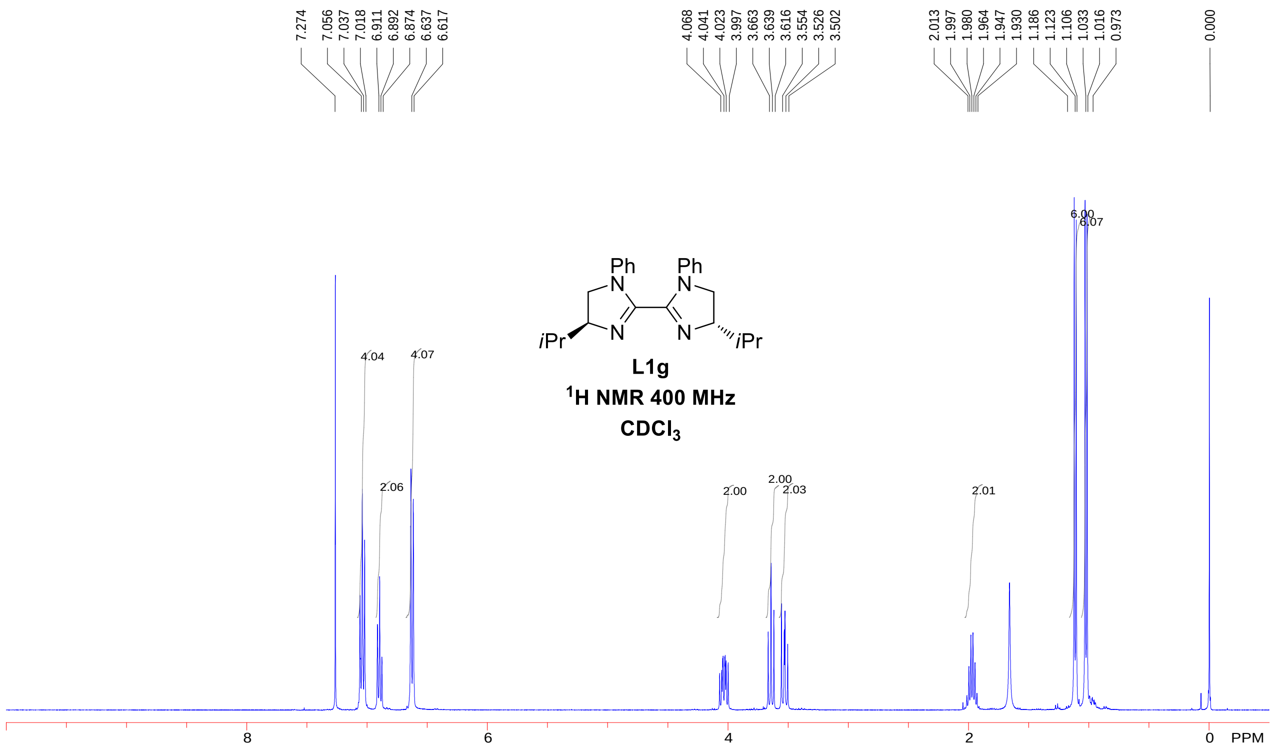

Supplementary Figure 13. ^{1}H NMR spectrum for L1g

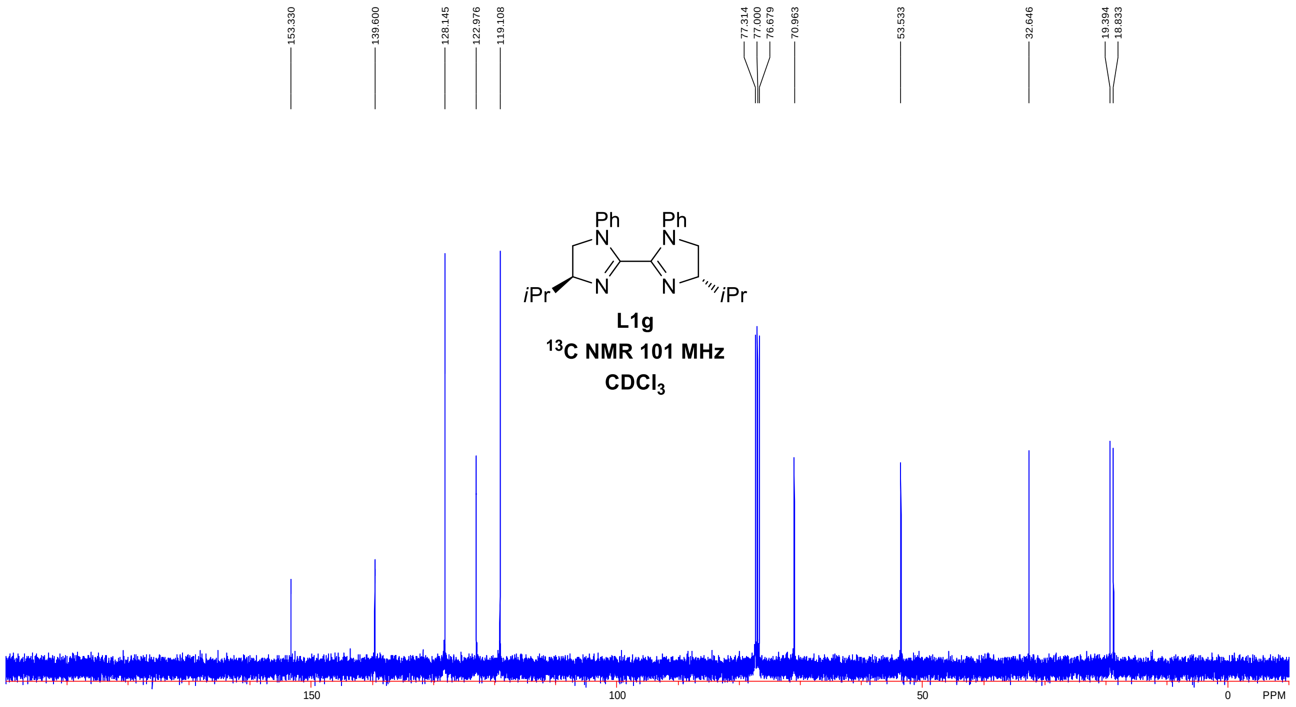

Supplementary Figure 14. ^{13}C NMR spectrum for L1g

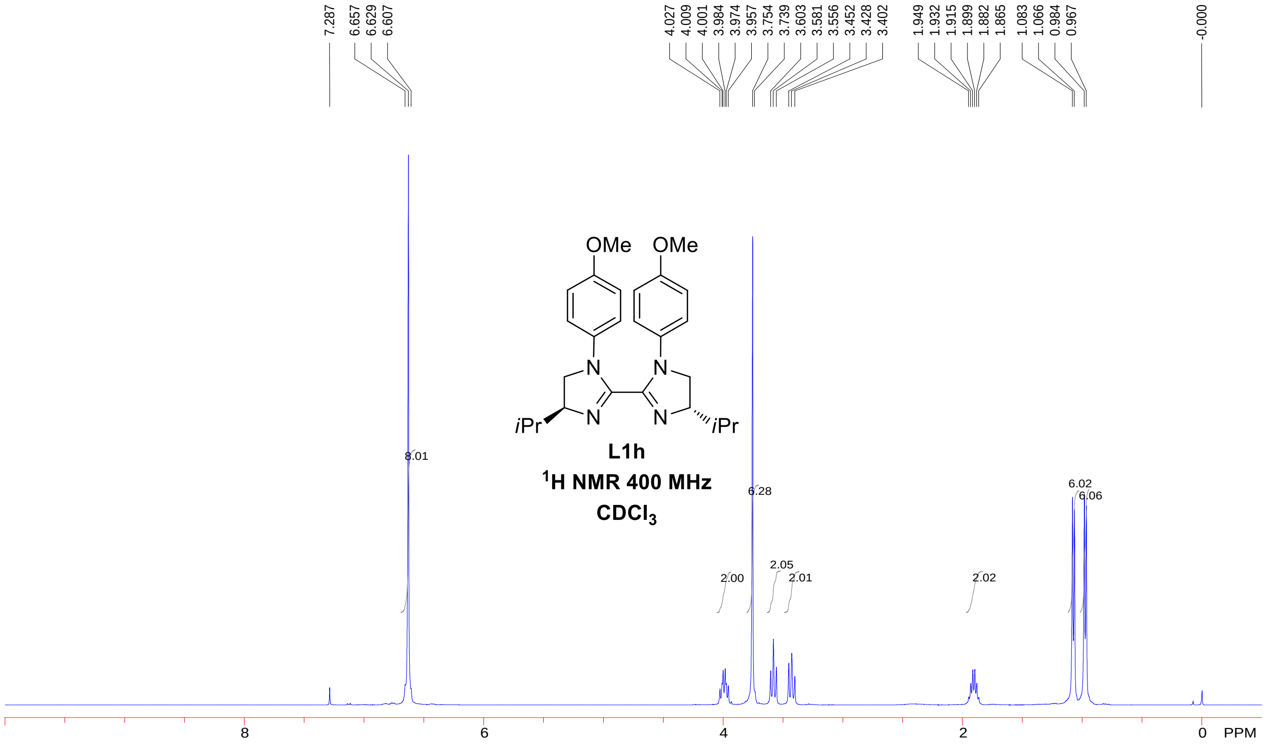

Supplementary Figure 15. ^{1}H NMR spectrum for L1h

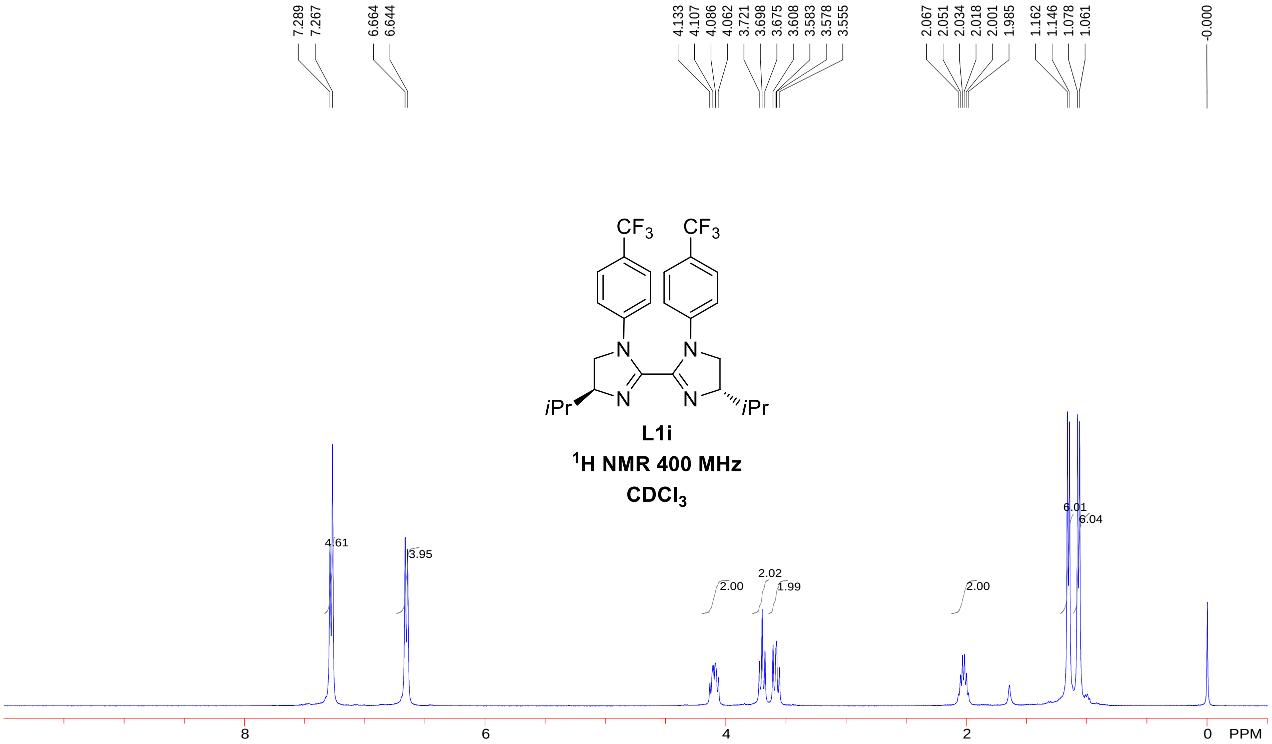

Supplementary Figure 16. ^{1}H NMR spectrum for L1i

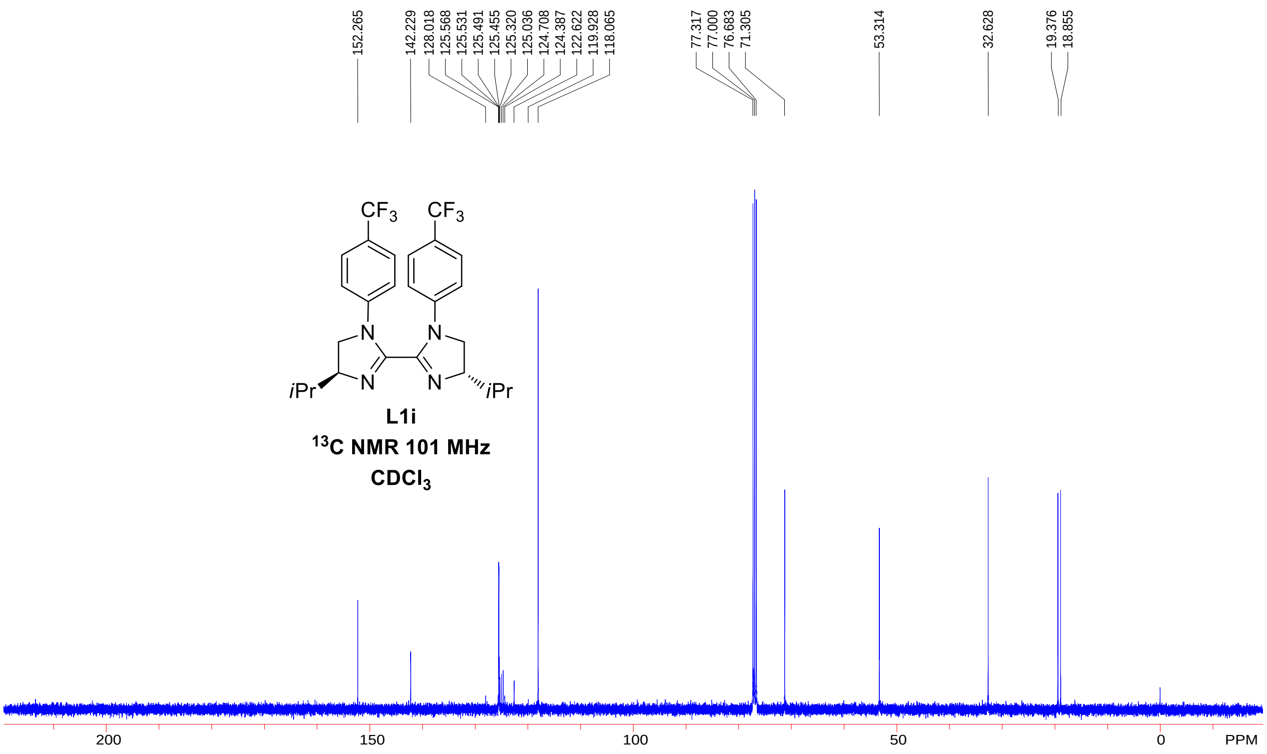

Supplementary Figure 17. ^{13}C NMR spectrum for L1i

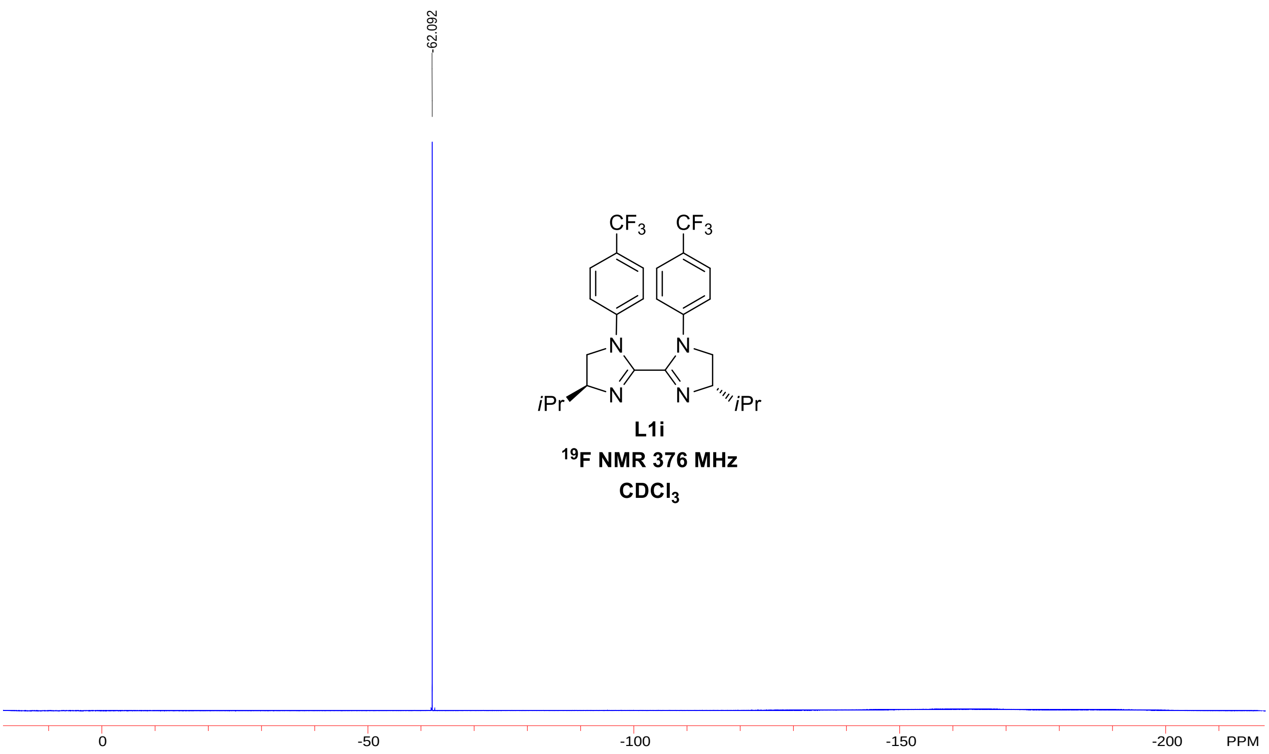

Supplementary Figure 18. ^{19}FNMR spectrum for L1i

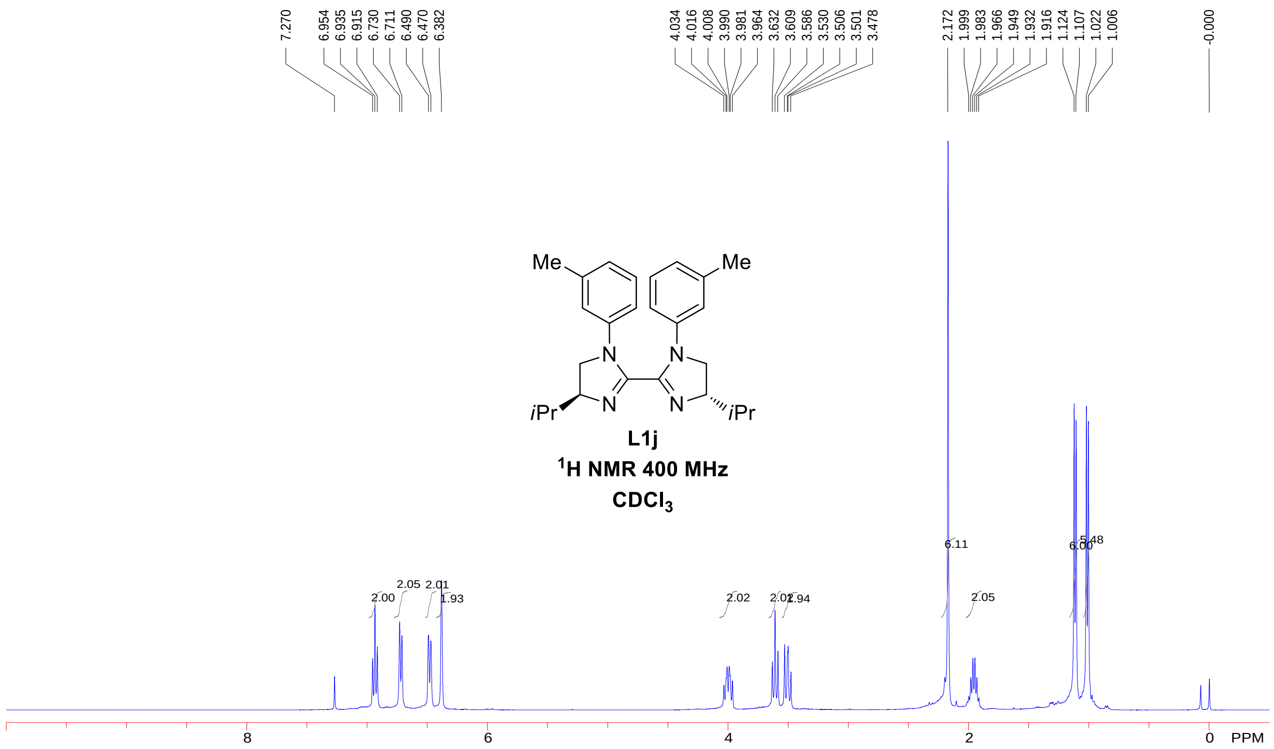

Supplementary Figure 19. ^{1}H NMR spectrum for L1j

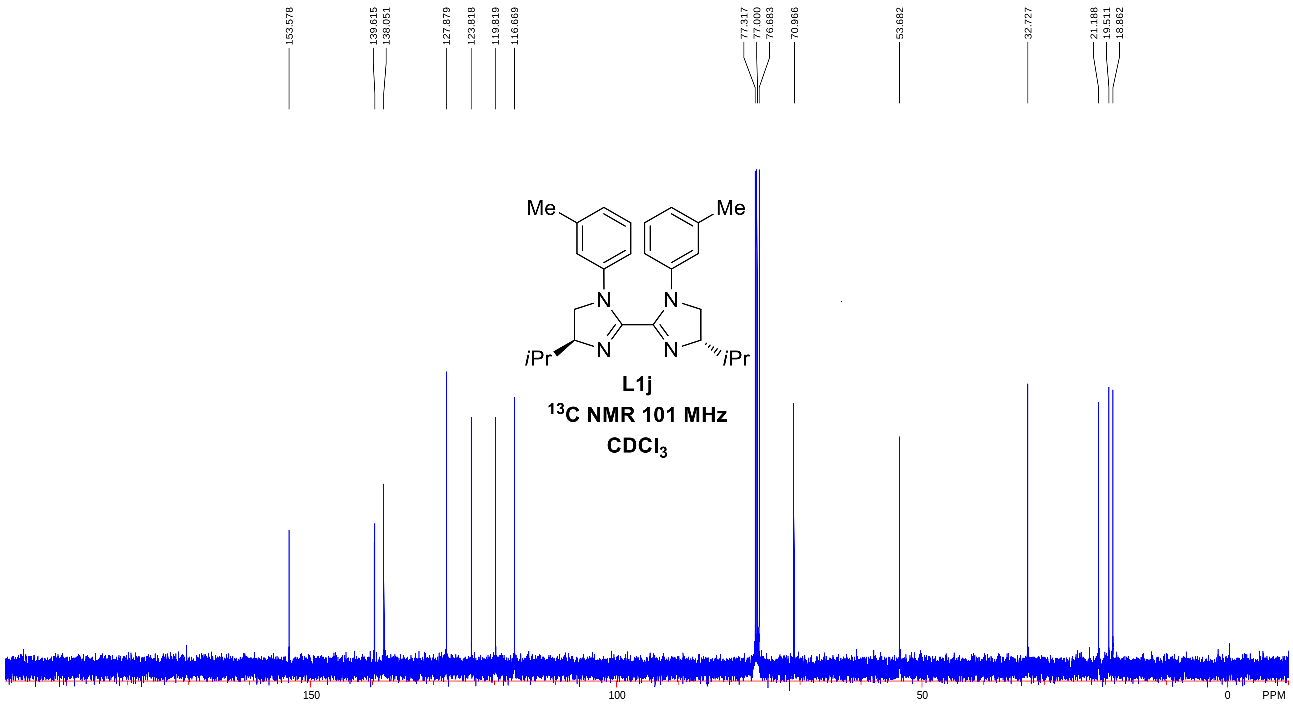

Supplementary Figure 20. ^{13}C NMR spectrum for L1j

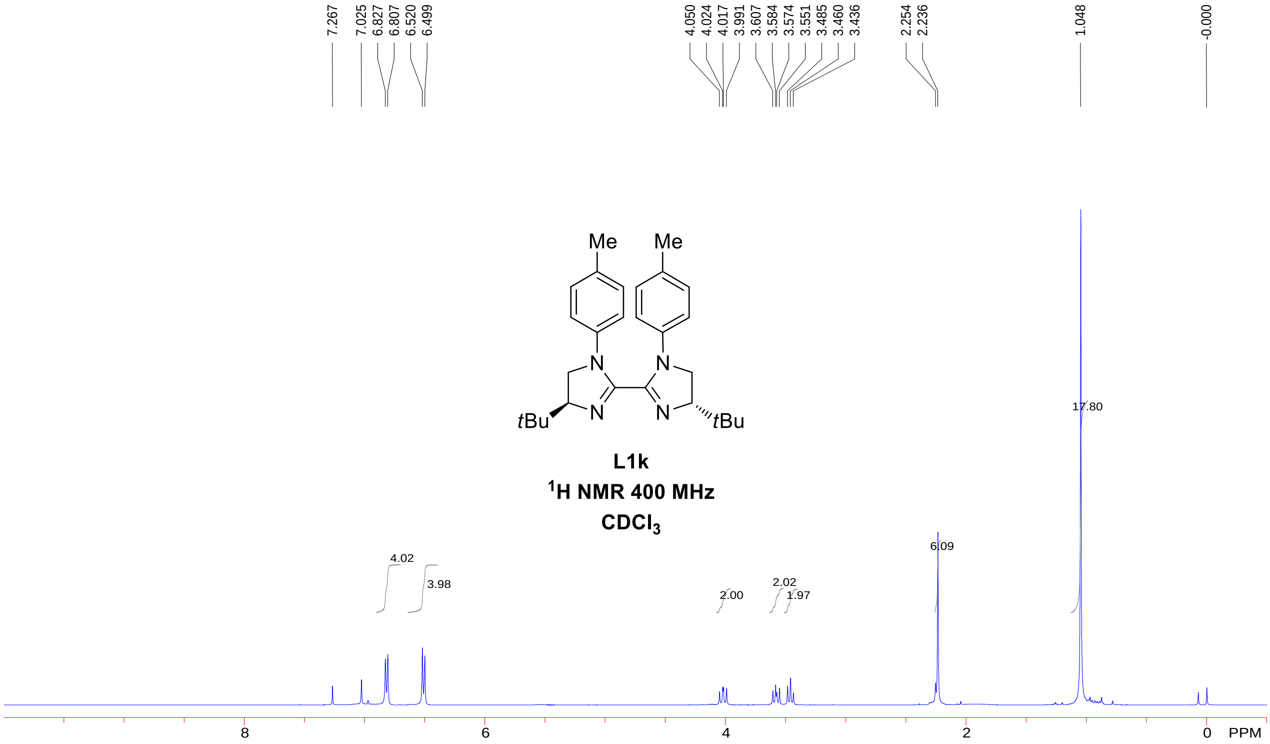

Supplementary Figure 21. ^{1}H NMR spectrum for L1k

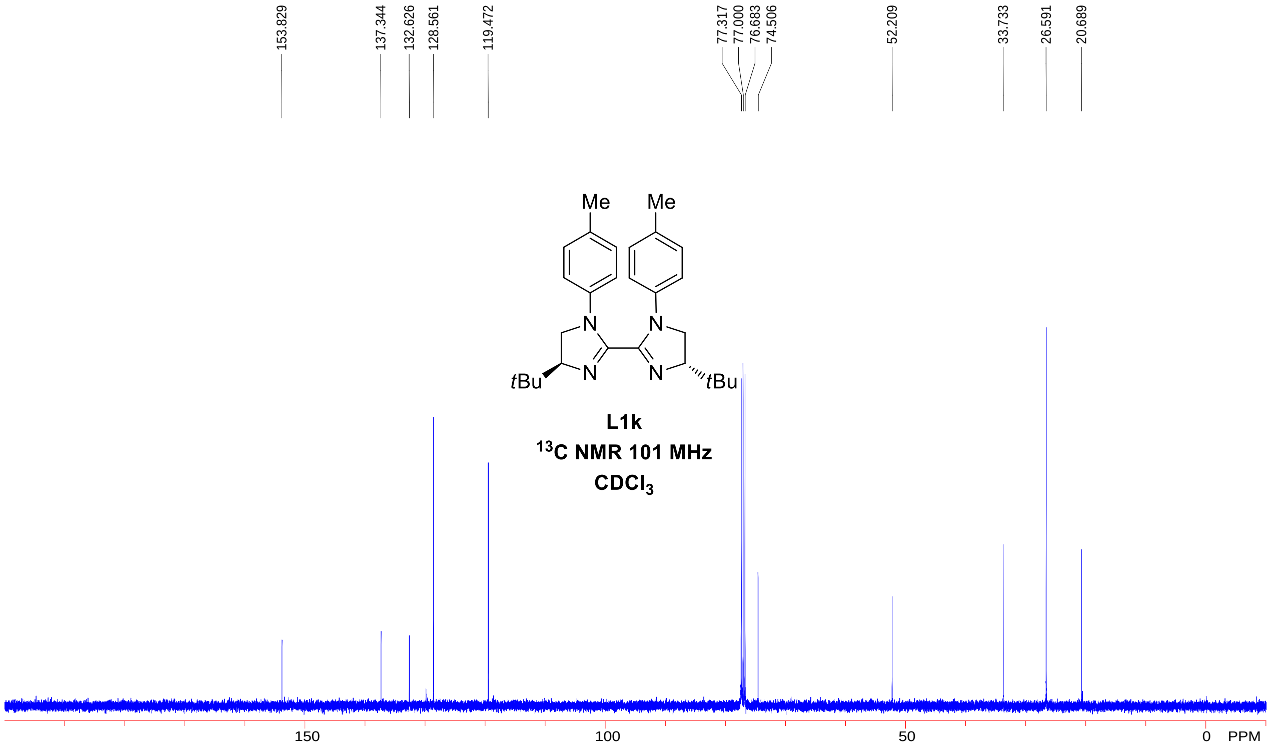

Supplementary Figure 22. ^{13}C NMR spectrum for L1k

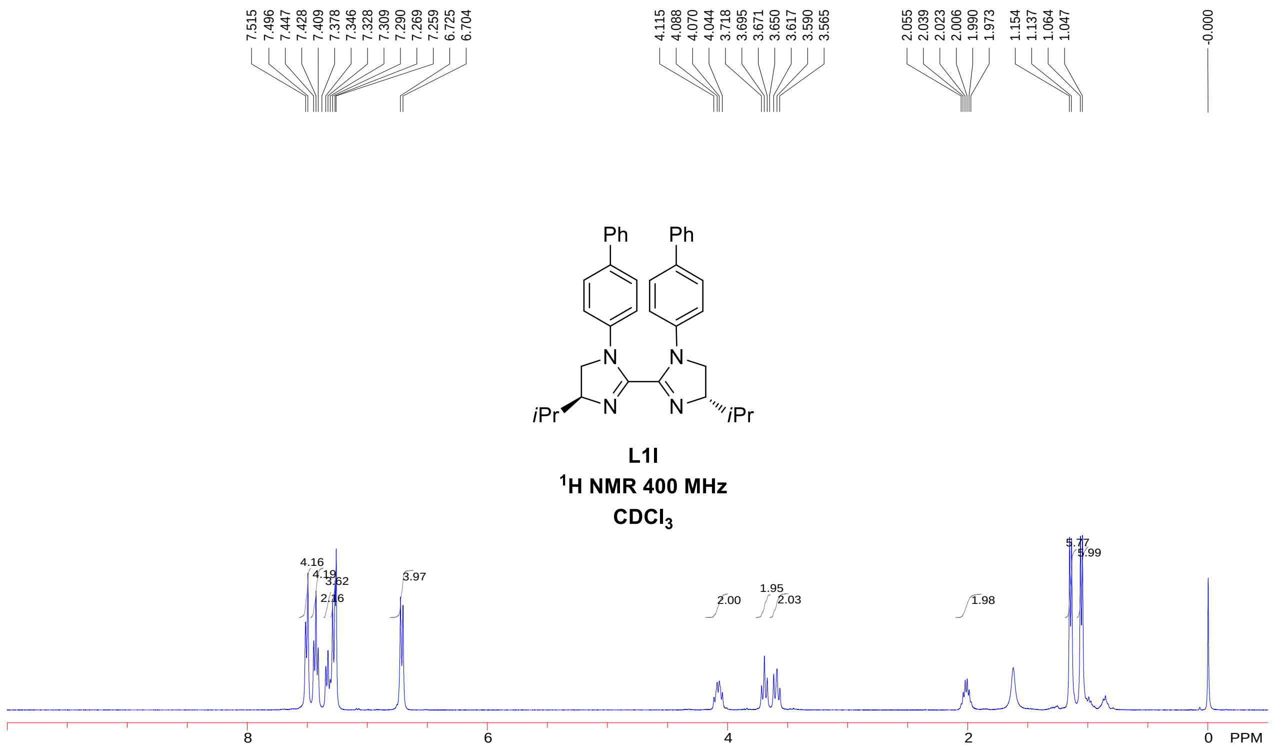

Supplementary Figure 23. ^{1}H NMR spectrum for L1l

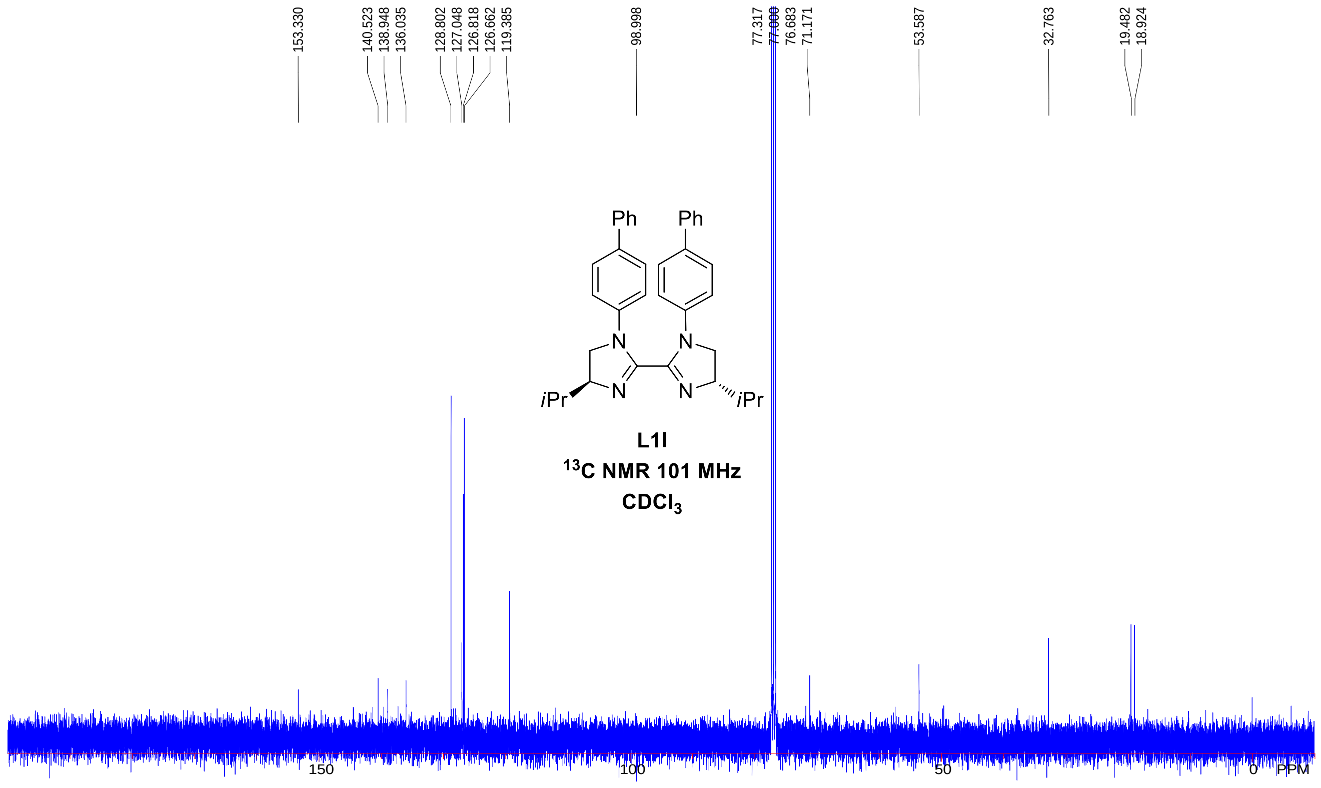

Supplementary Figure 24. ^{13}C NMR spectrum for L1l

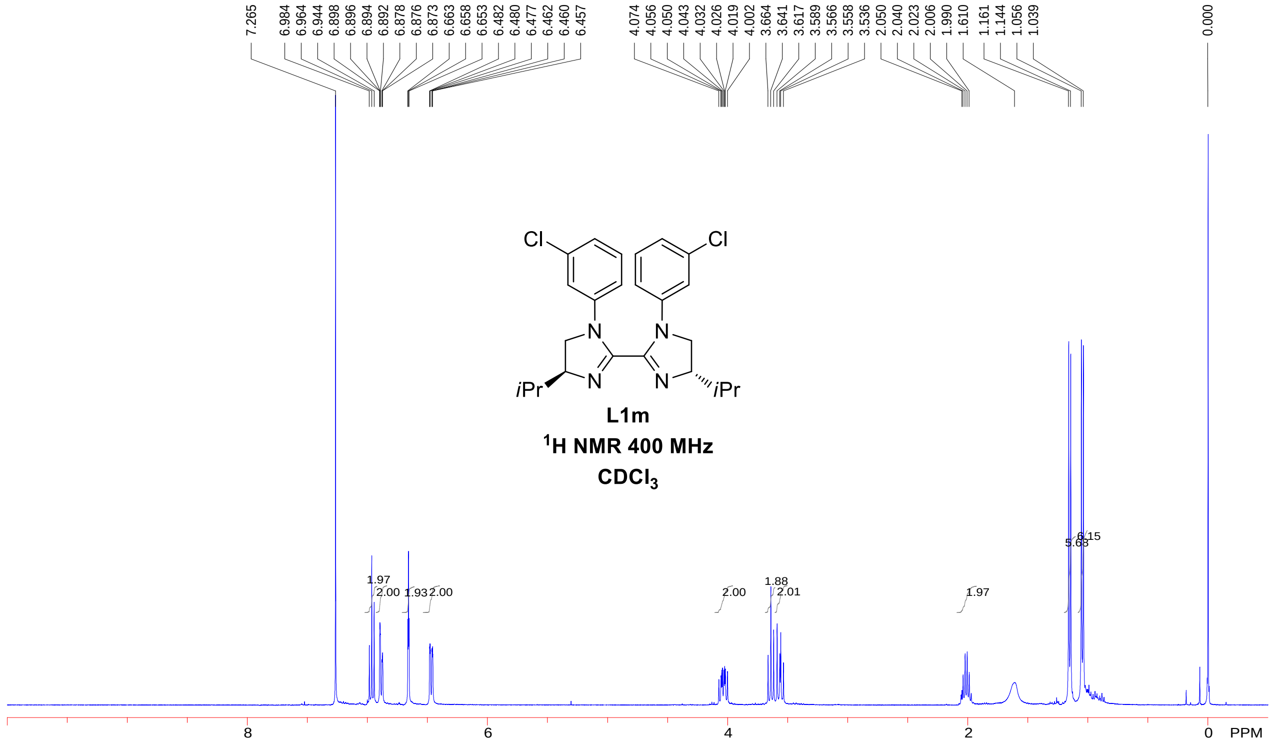

Supplementary Figure 25. ^{1}H NMR spectrum for L1m

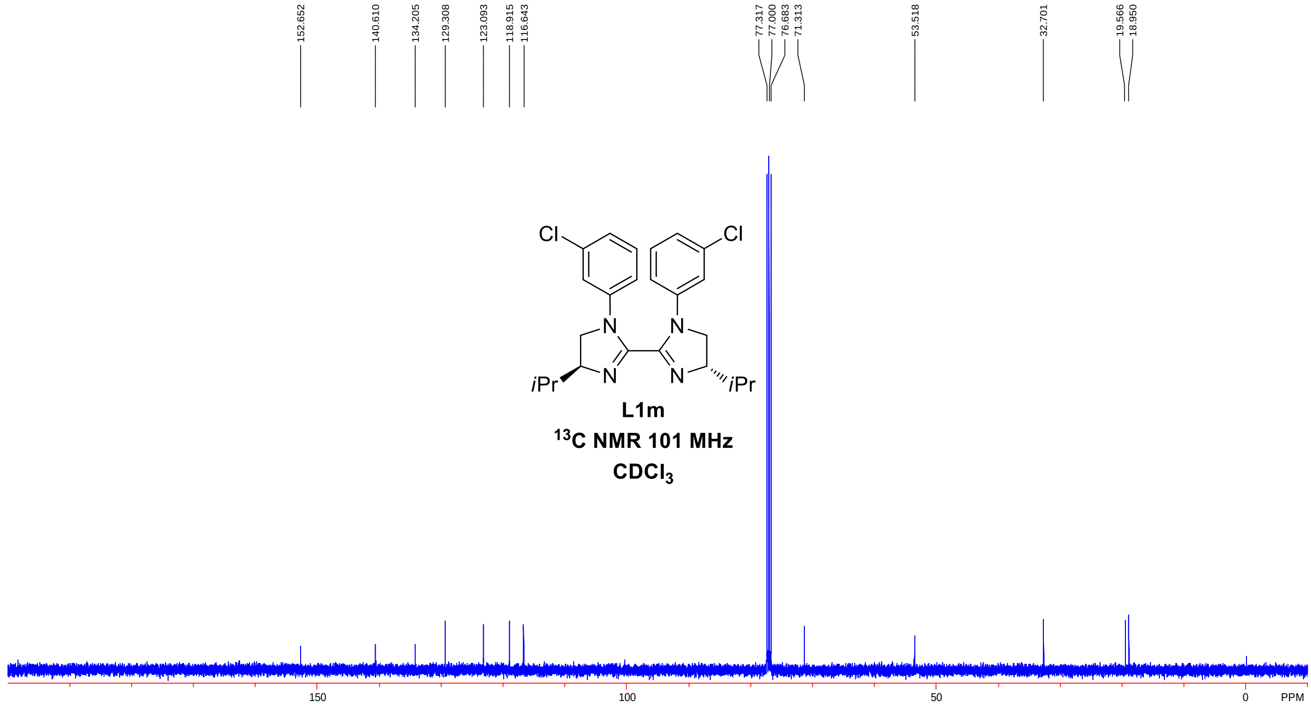

Supplementary Figure 26. ^{13}C NMR spectrum for L1m

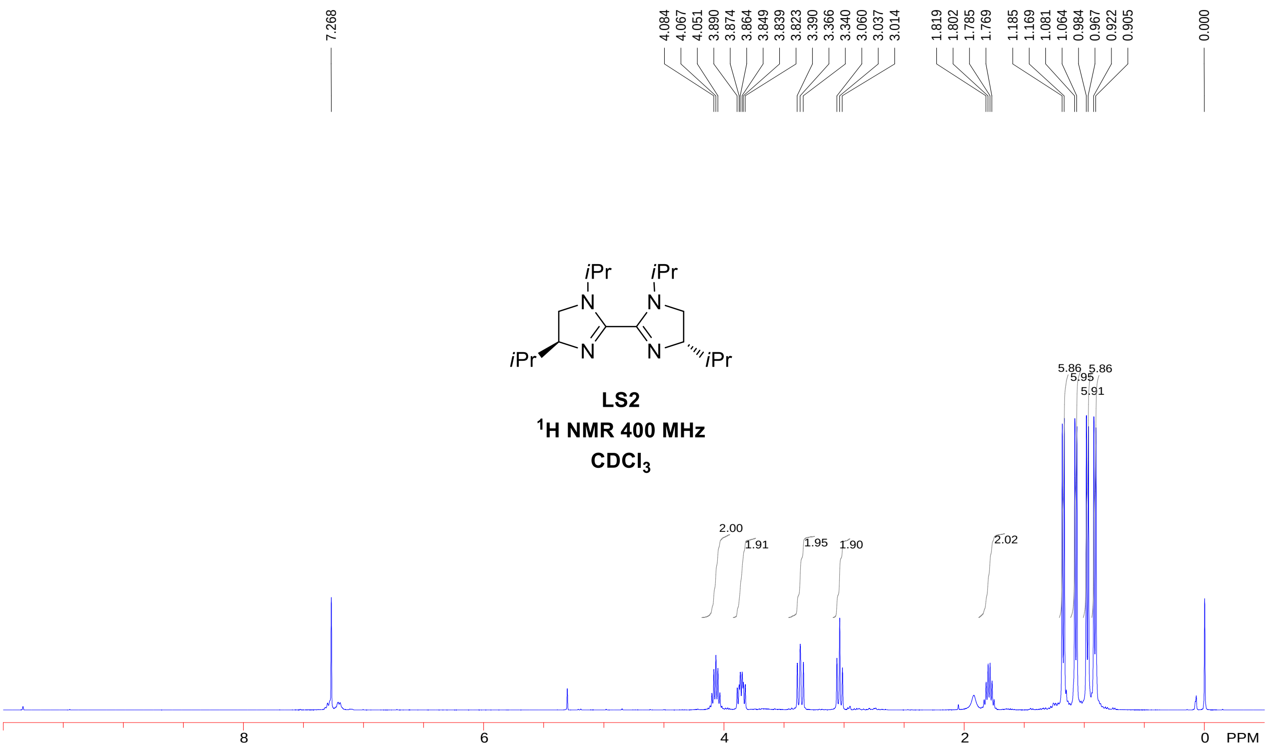

Supplementary Figure 27. ^{1}H NMR spectrum for LS2

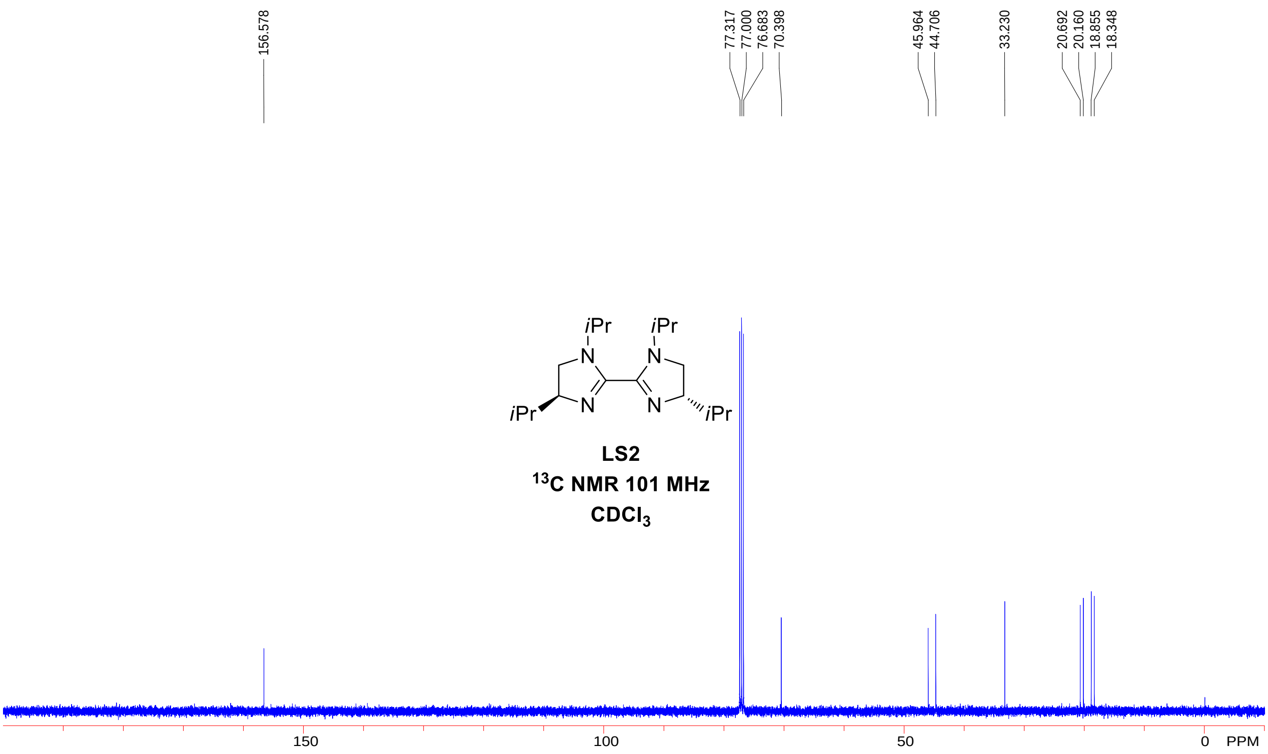

Supplementary Figure 28. ^{13}C NMR spectrum for LS2

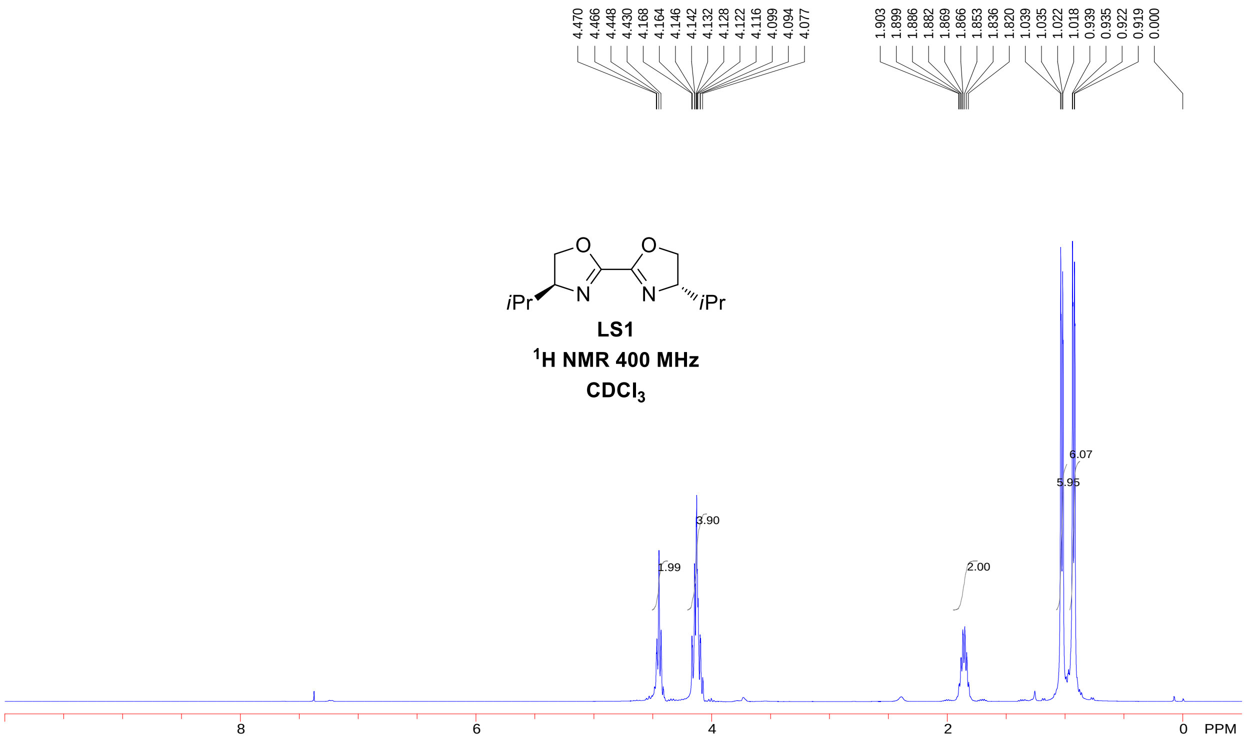

Supplementary Figure 29. ^{1}H NMR spectrum for LS1

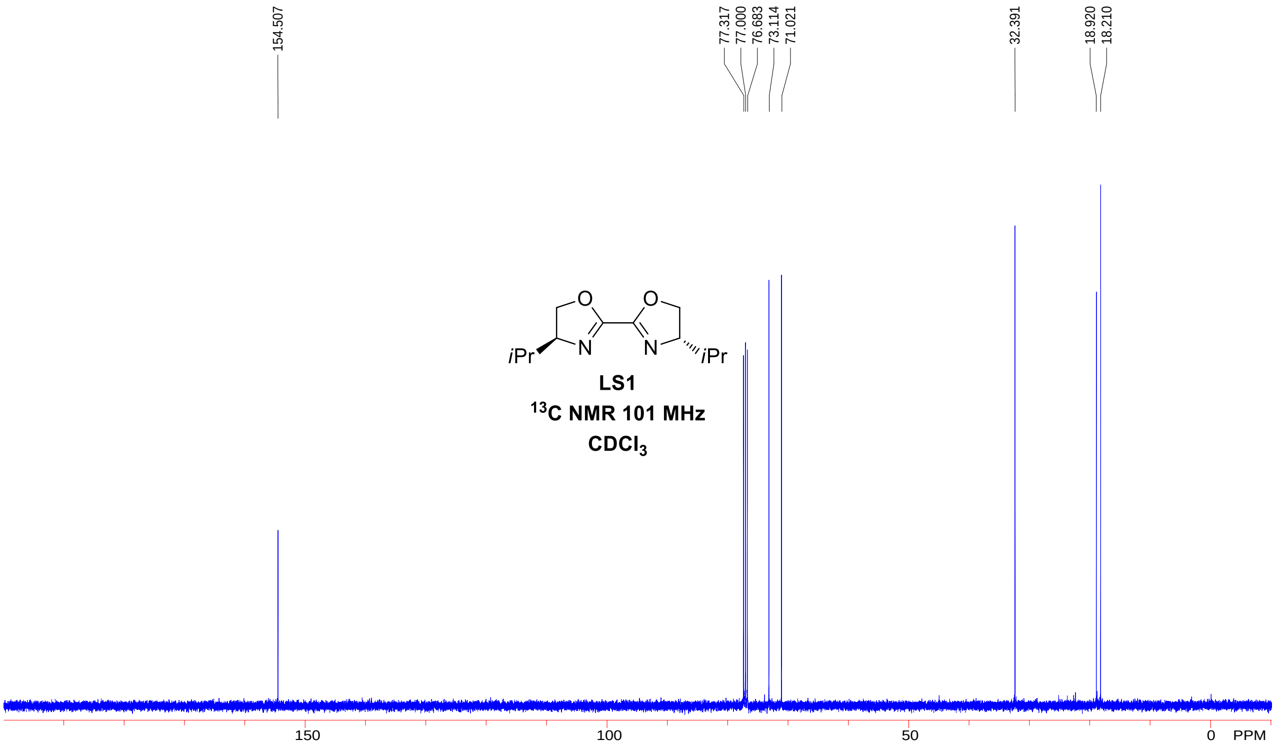

Supplementary Figure 30. ^{13}C NMR spectrum for LS1

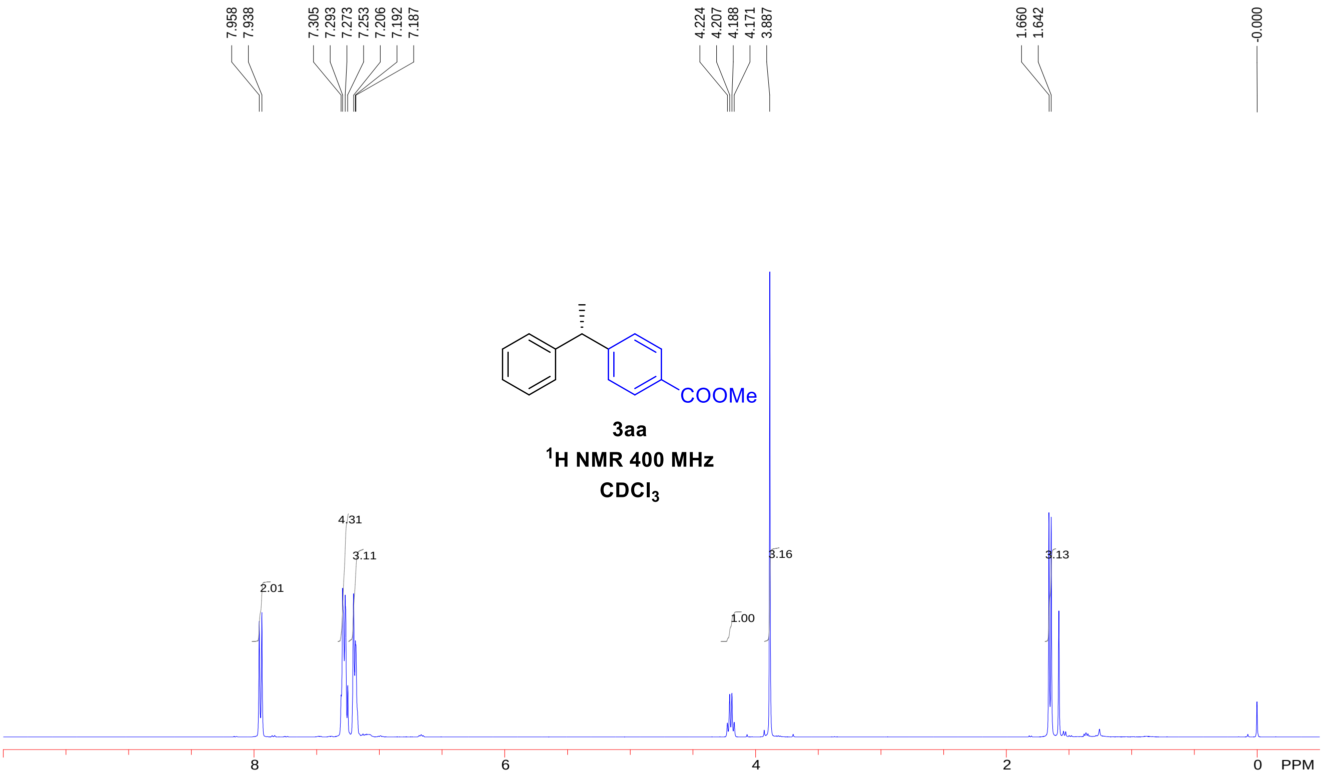

Supplementary Figure 31. ^{1}H NMR spectrum for **3aa**

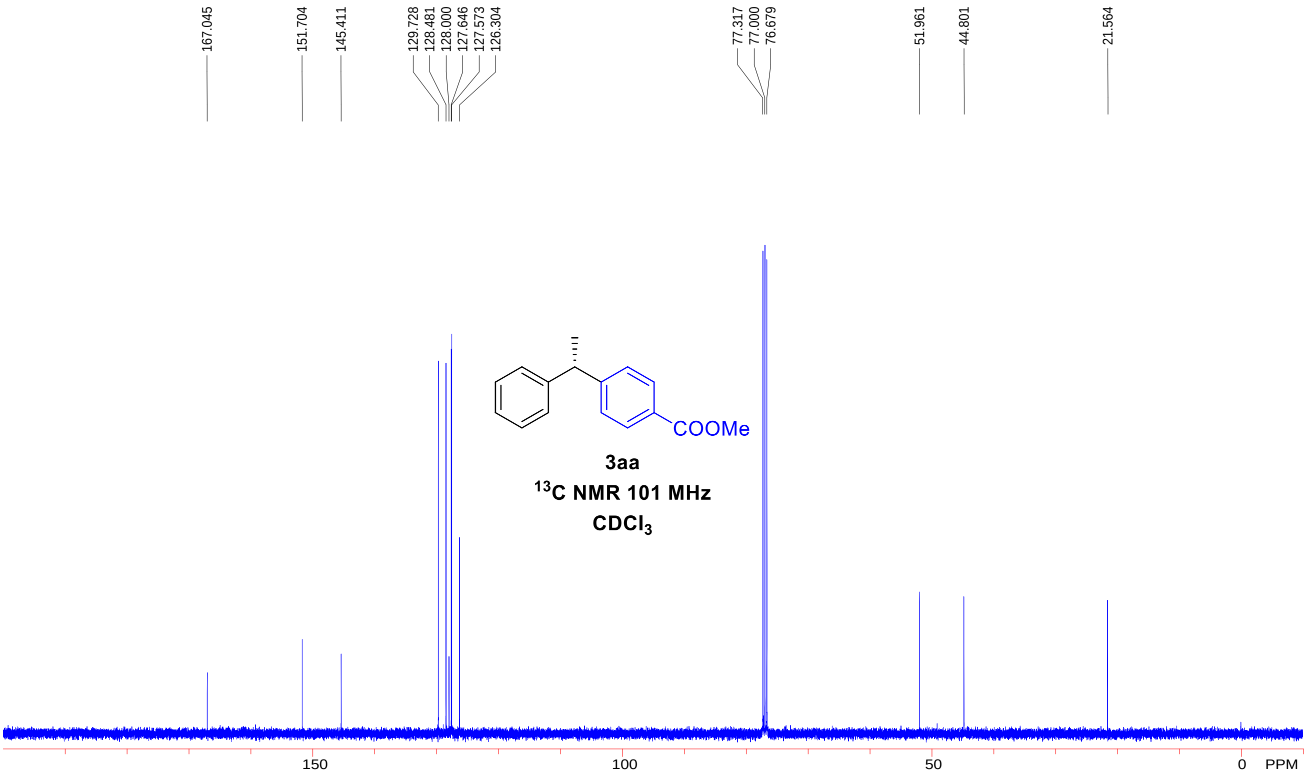

Supplementary Figure 32. ^{13}C NMR spectrum for **3aa**

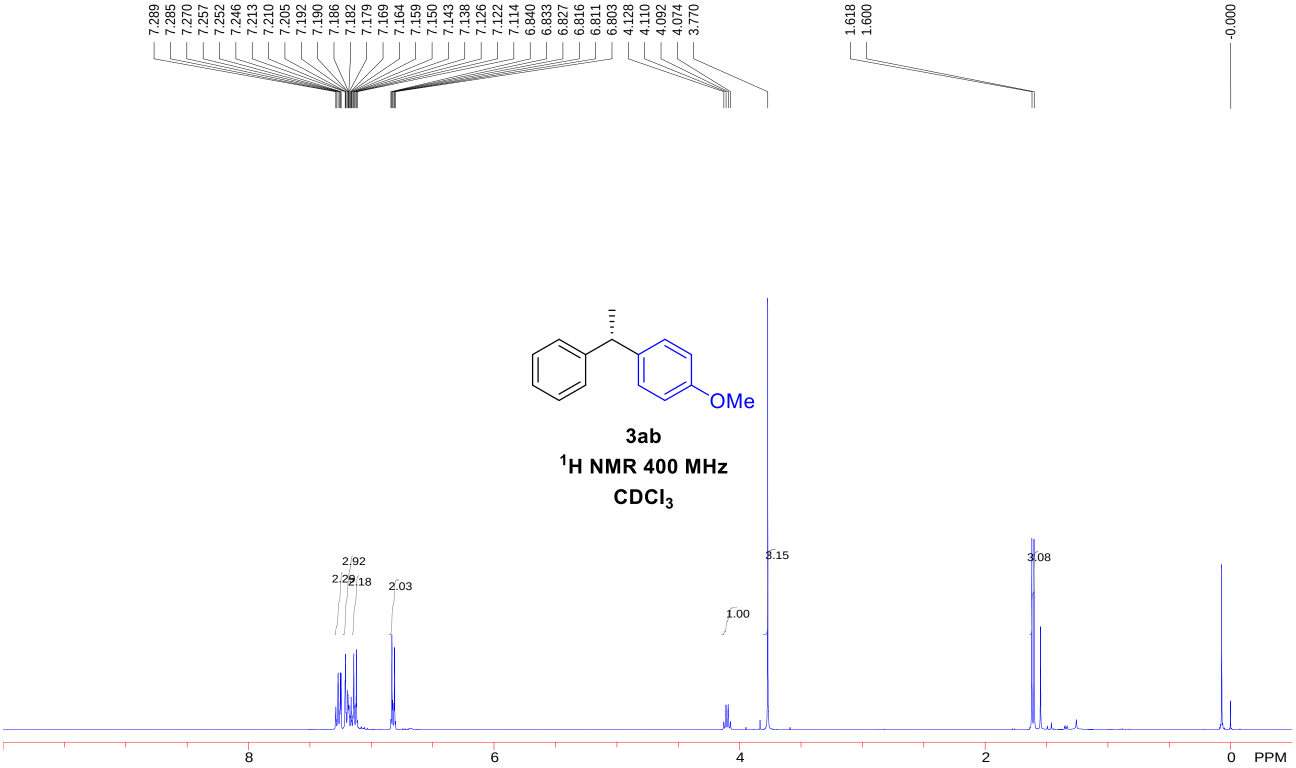

Supplementary Figure 33. ^{1}H NMR spectrum for **3ab**

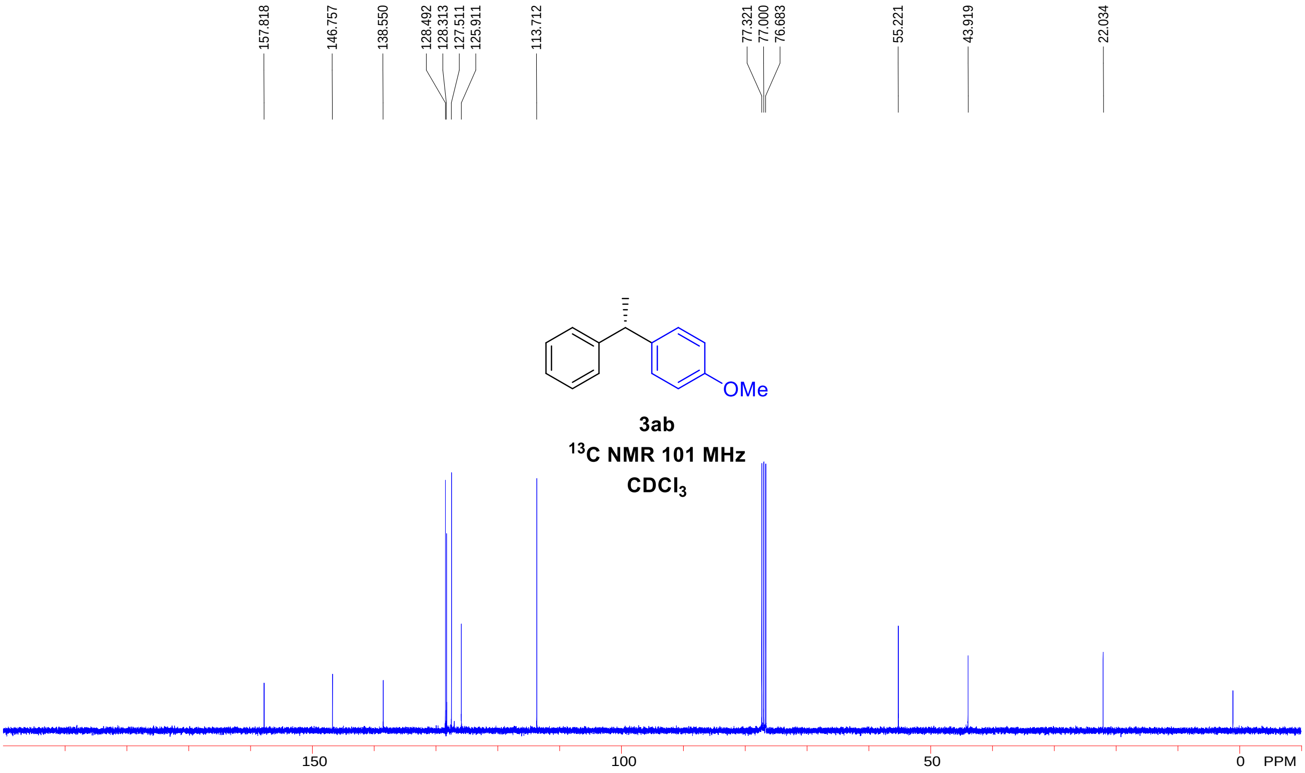

Supplementary Figure 34. ^{13}C NMR spectrum for **3ab**

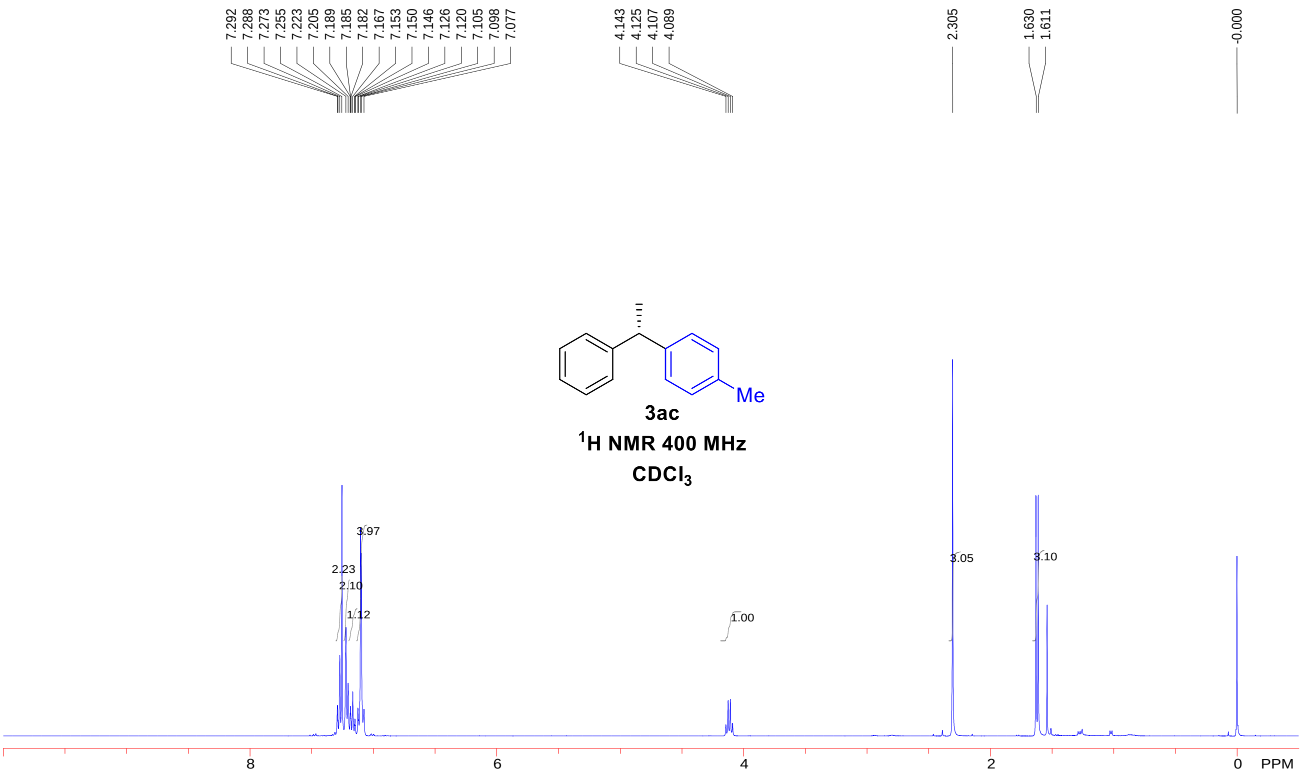

Supplementary Figure 35. ^{1}H NMR spectrum for **3ac**

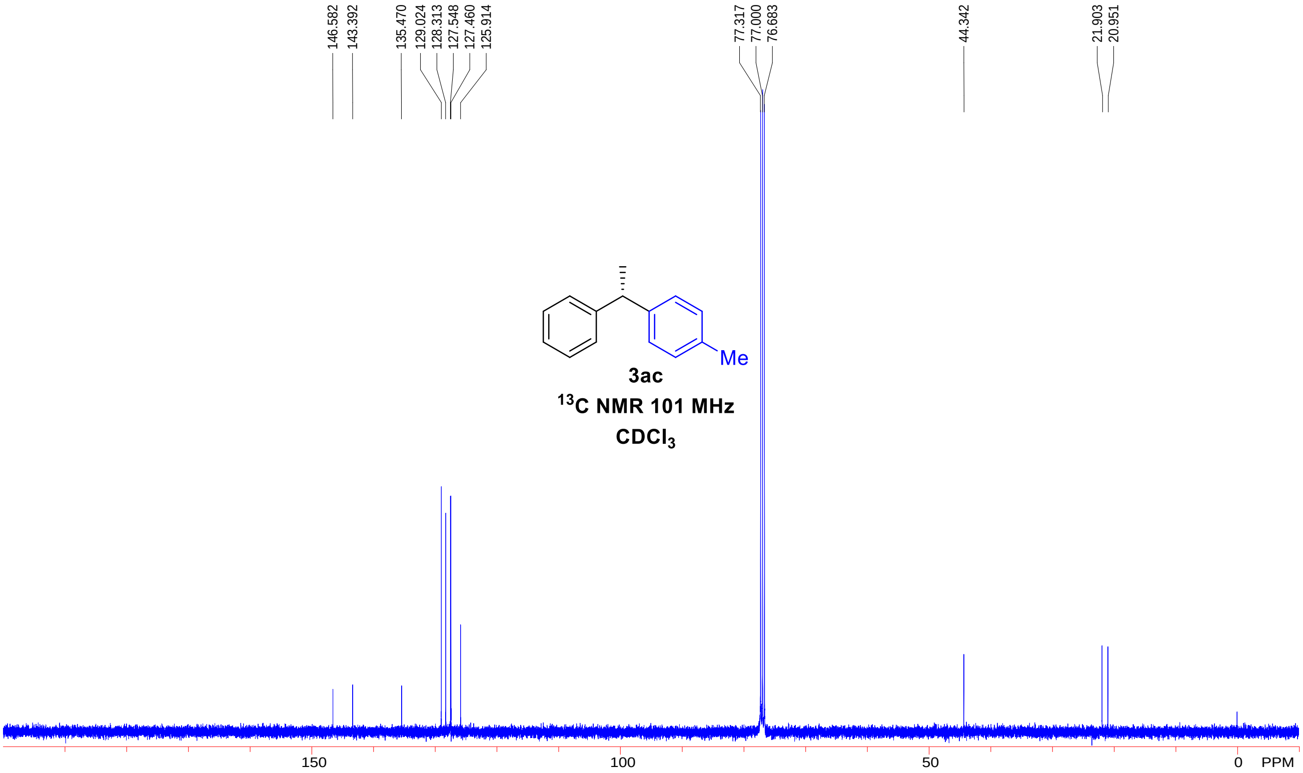

Supplementary Figure 36. ^{13}C NMR spectrum for **3ac**

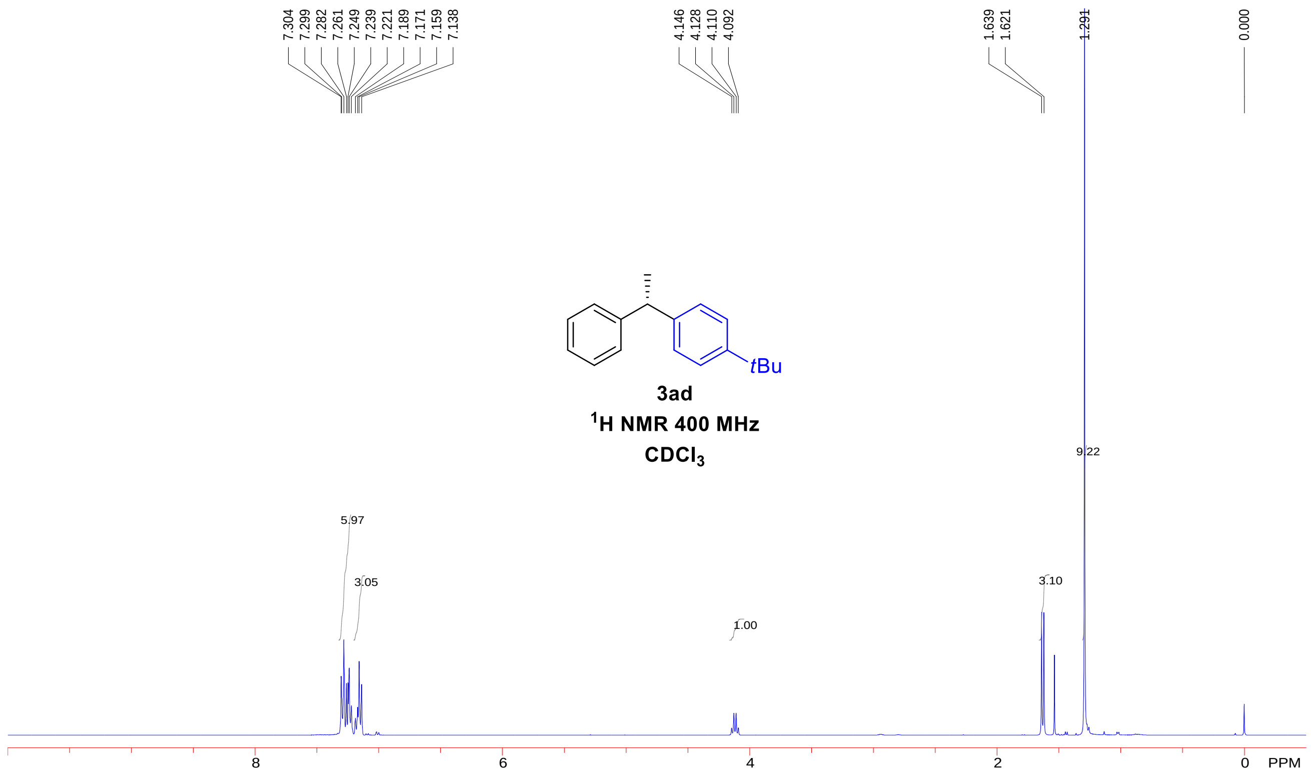

Supplementary Figure 37. ^{1}H NMR spectrum for **3ad**

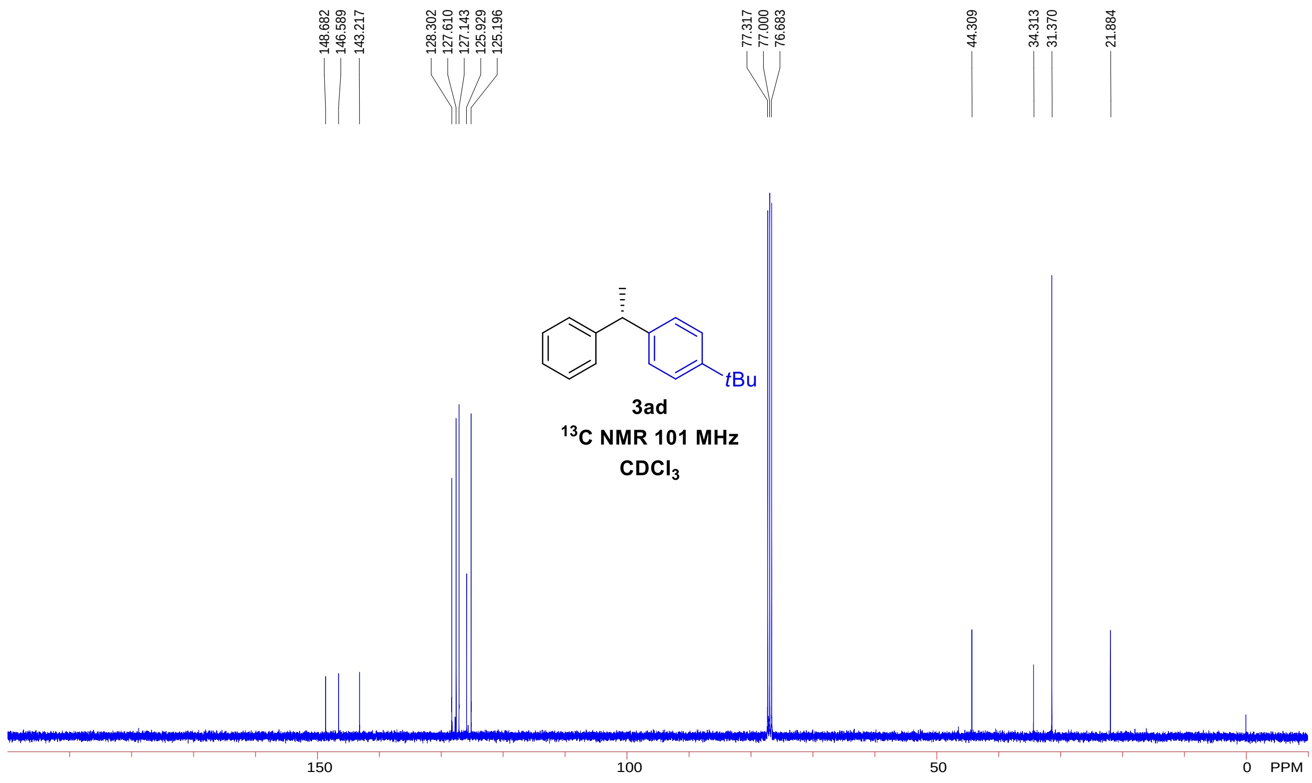

Supplementary Figure 38. ^{13}C NMR spectrum for **3ad**

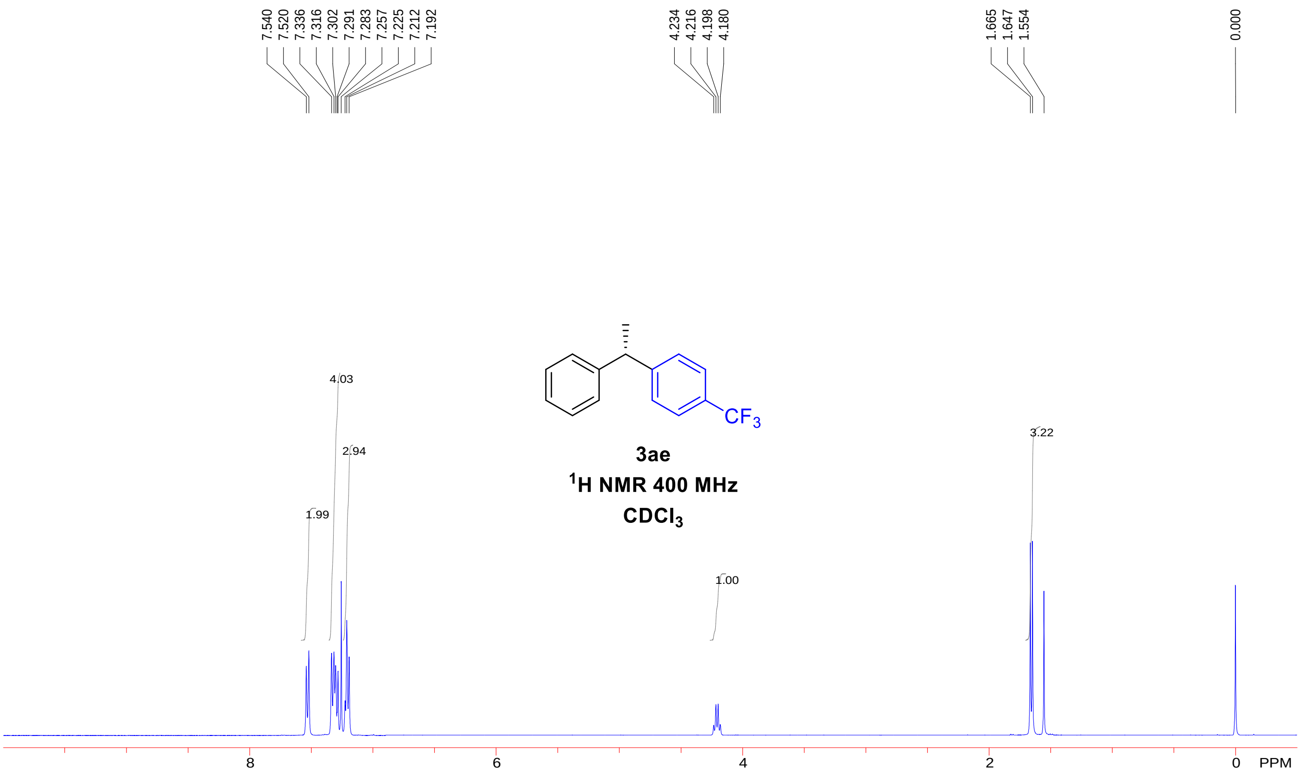

Supplementary Figure 39. ^{1}H NMR spectrum for **3ae**

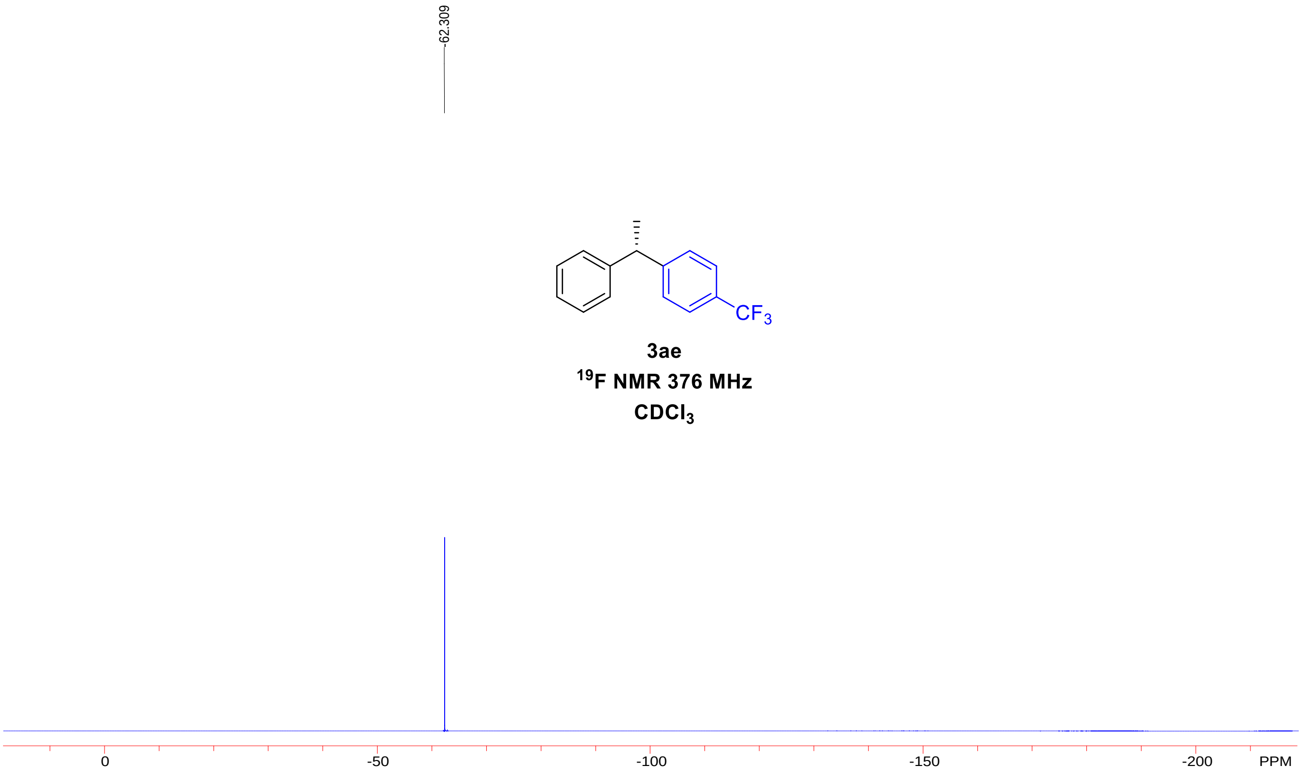

Supplementary Figure 40. ^{13}F NMR spectrum for **3ae**

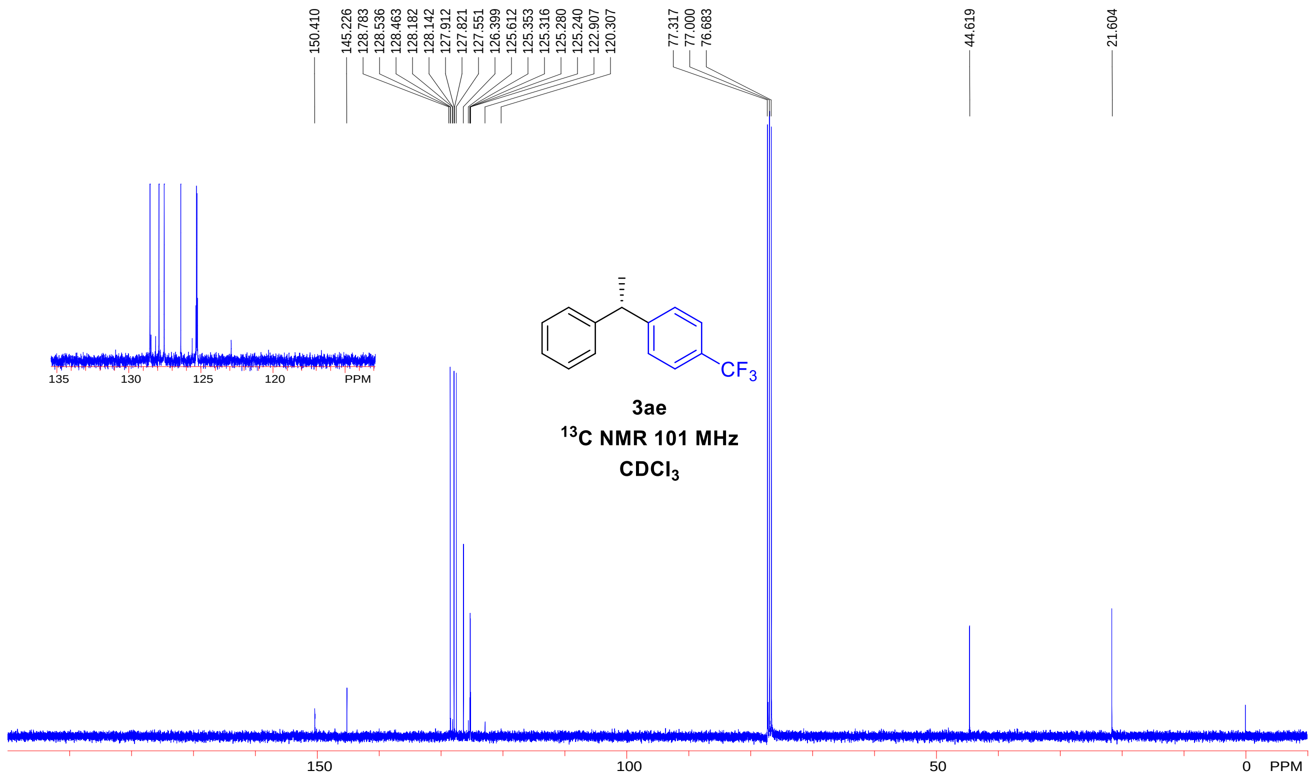

Supplementary Figure 41. ^{13}C NMR spectrum for **3ae**

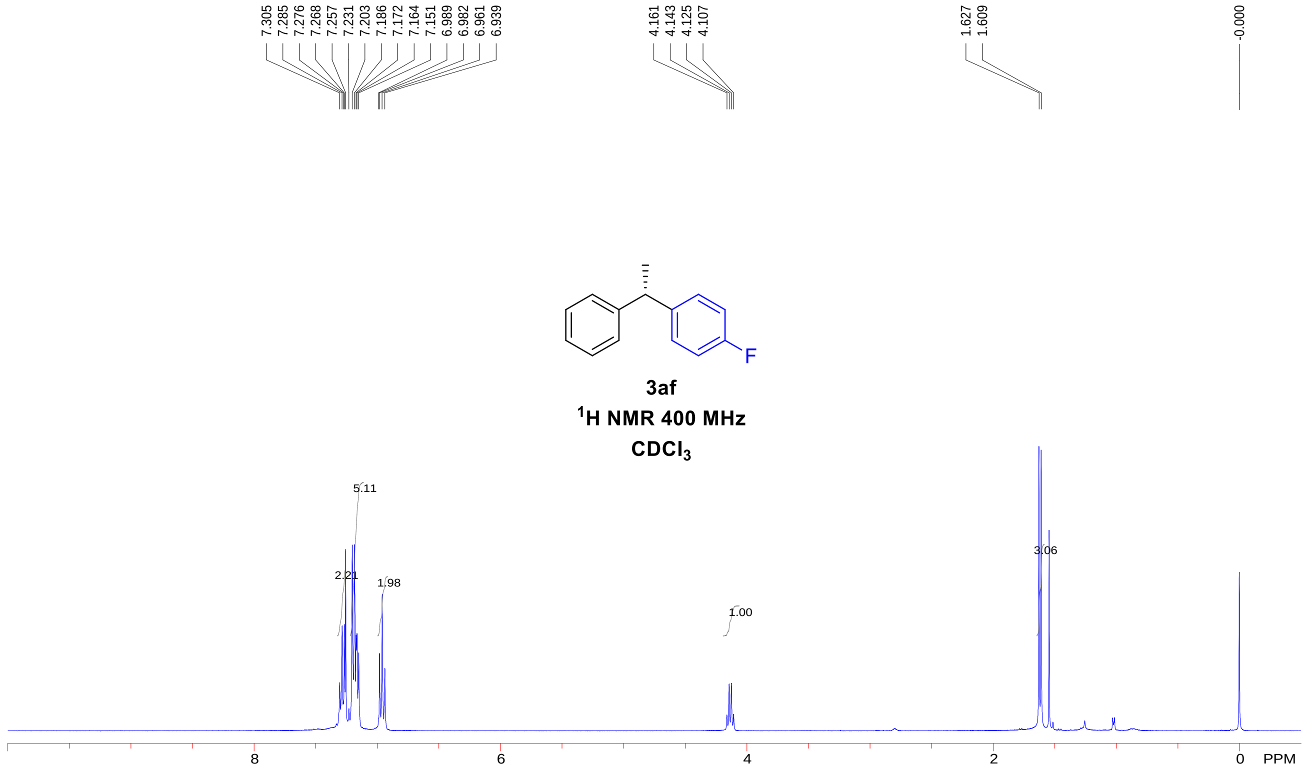

Supplementary Figure 42. ^{1}H NMR spectrum for **3af**

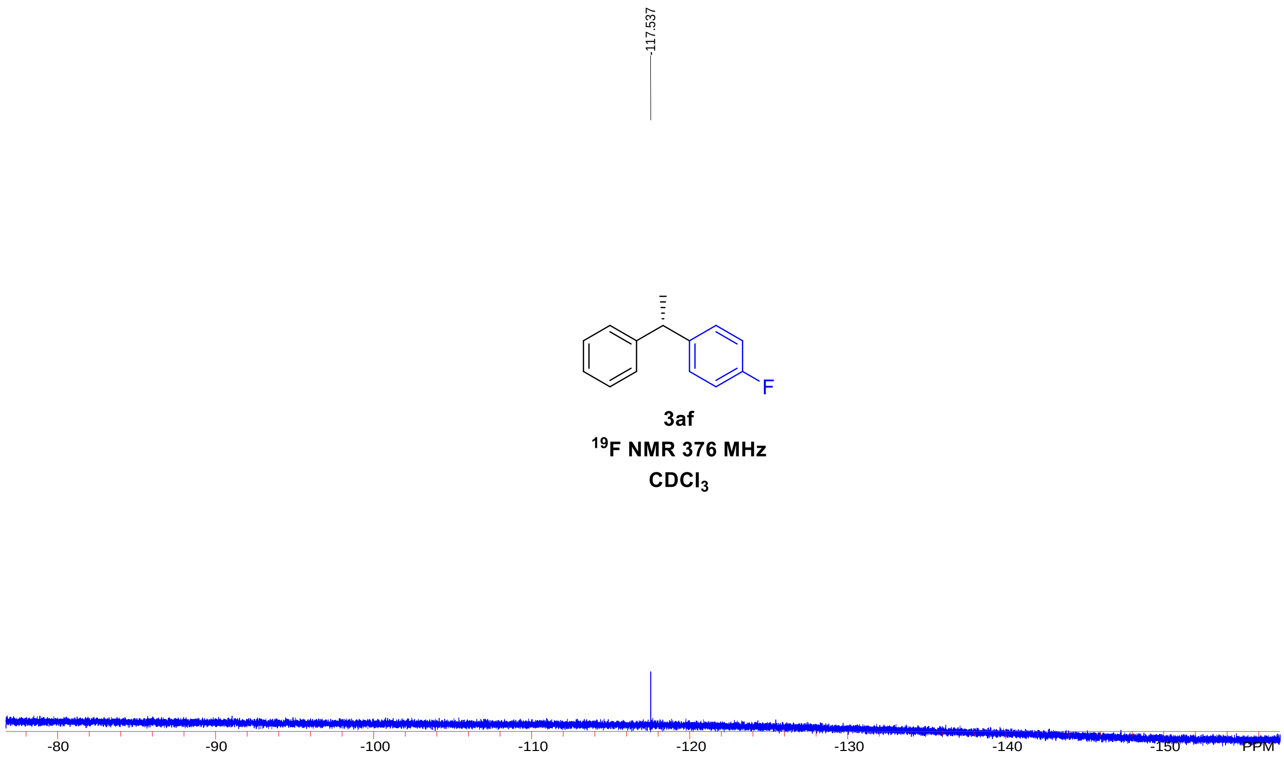

Supplementary Figure 43. ^{19}F NMR spectrum for **3af**

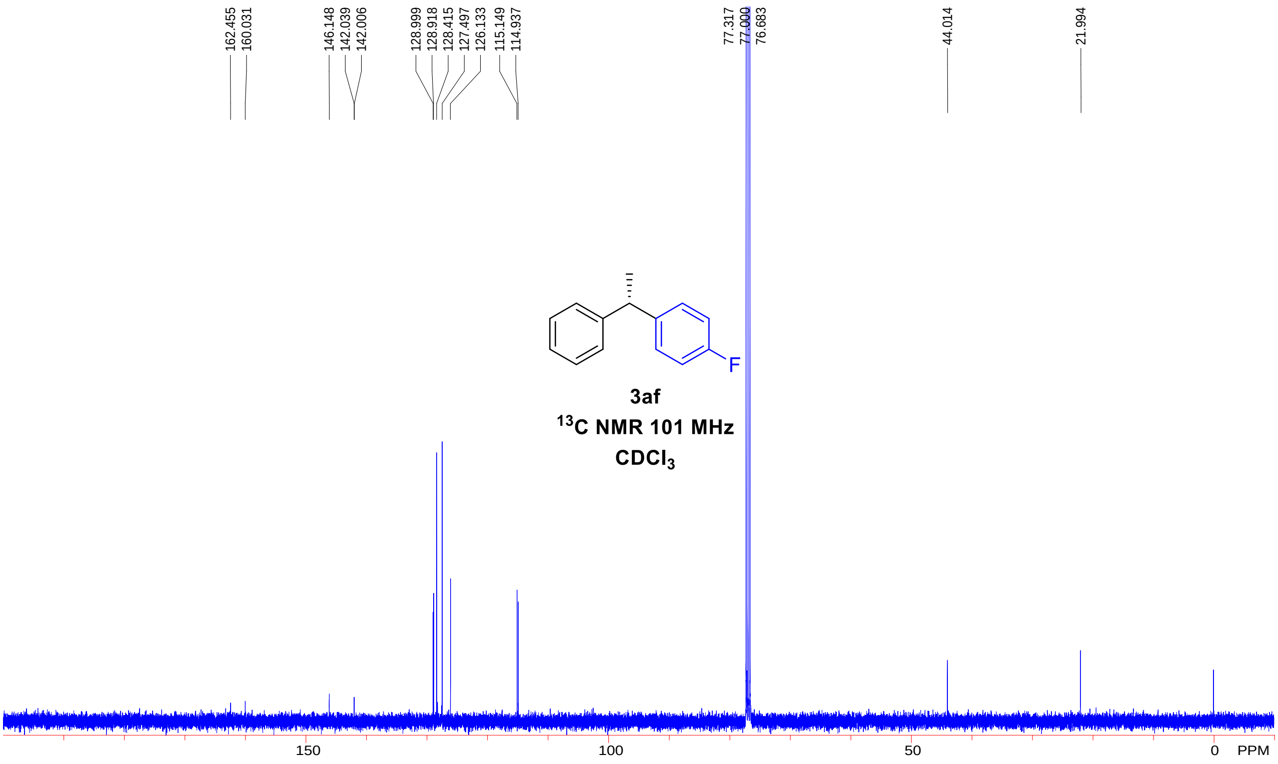

Supplementary Figure 44. ^{13}C NMR spectrum for **3af**

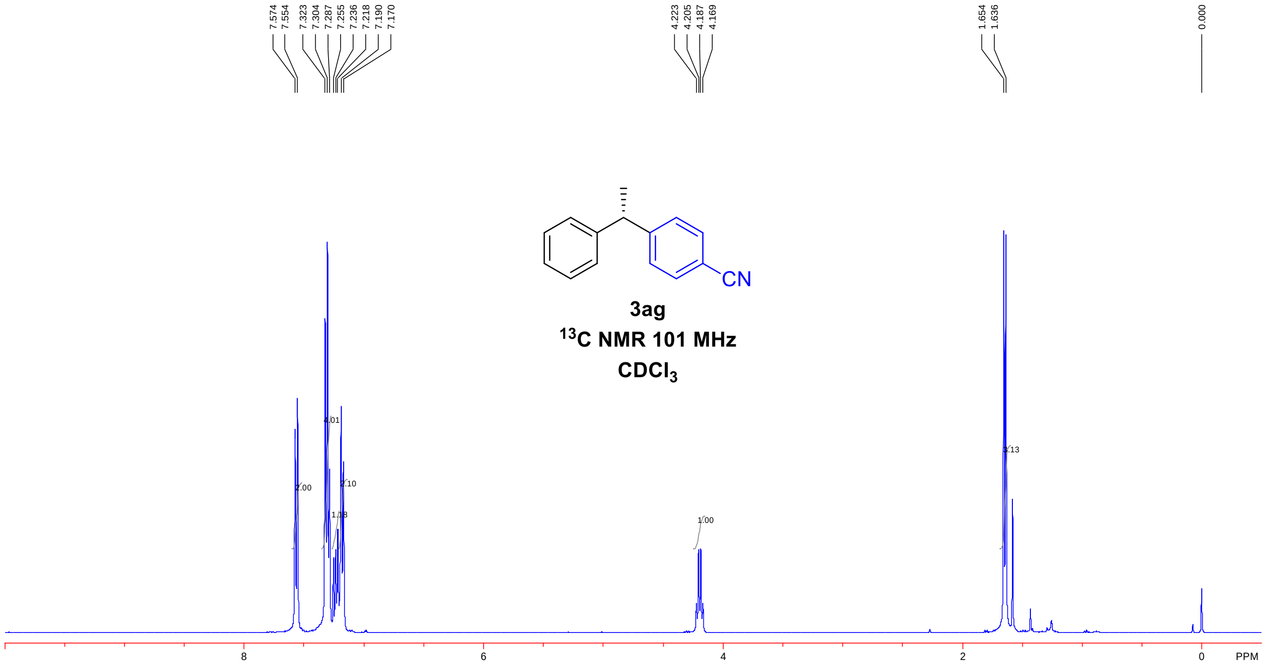

Supplementary Figure 45. ^{1}H NMR spectrum for **3ag**

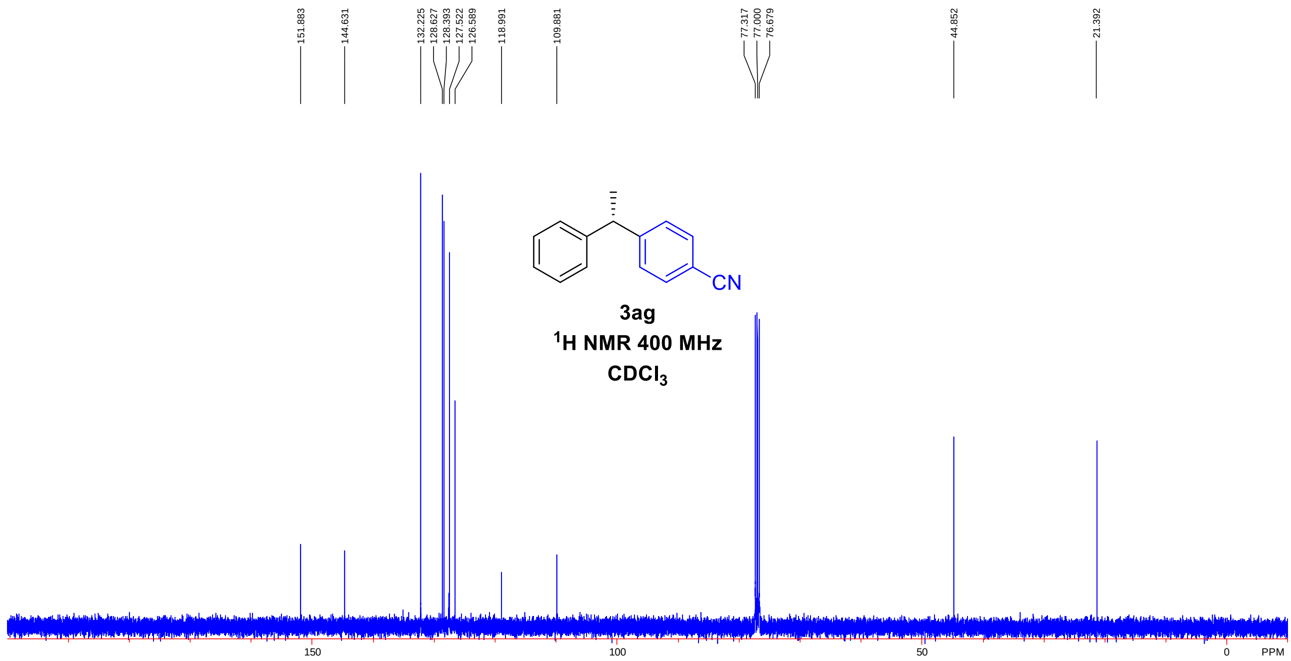

Supplementary Figure 46. ^{13}C NMR spectrum for **3ag**

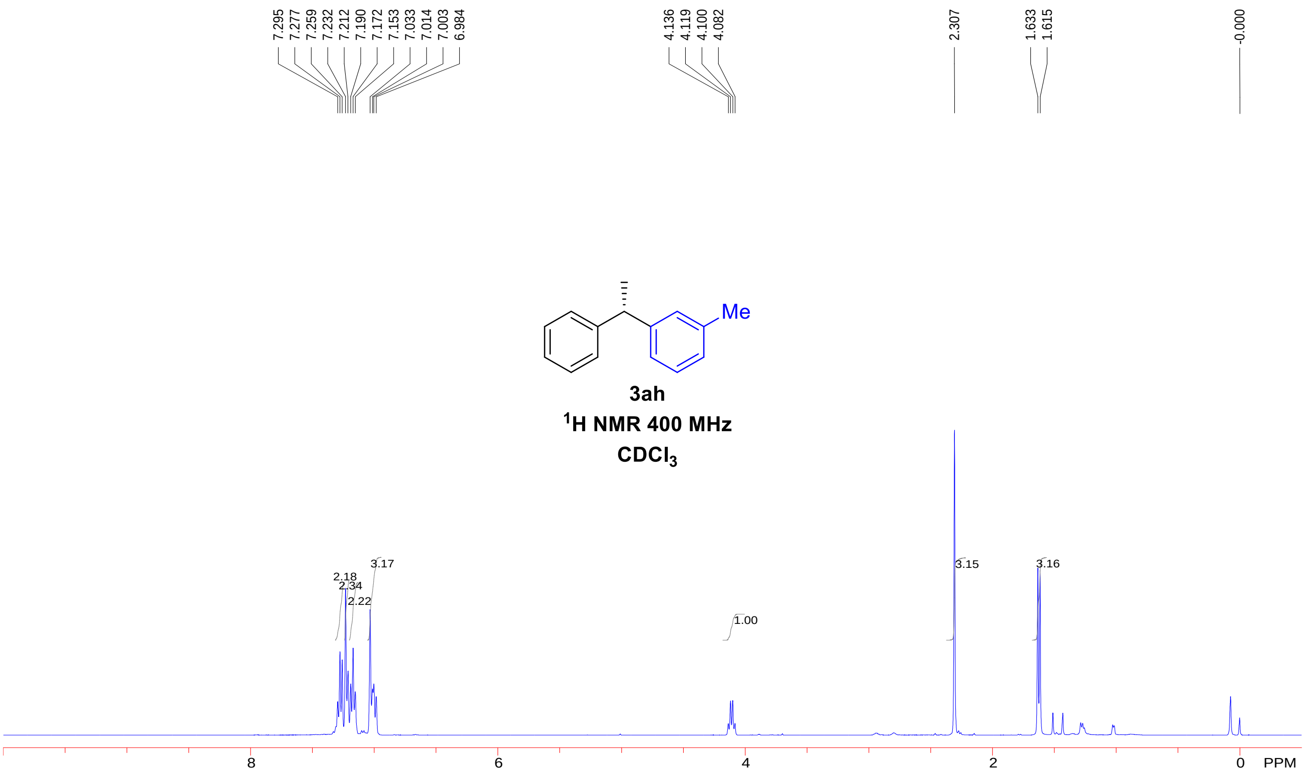

Supplementary Figure 47. ^{1}H NMR spectrum for **3ah**

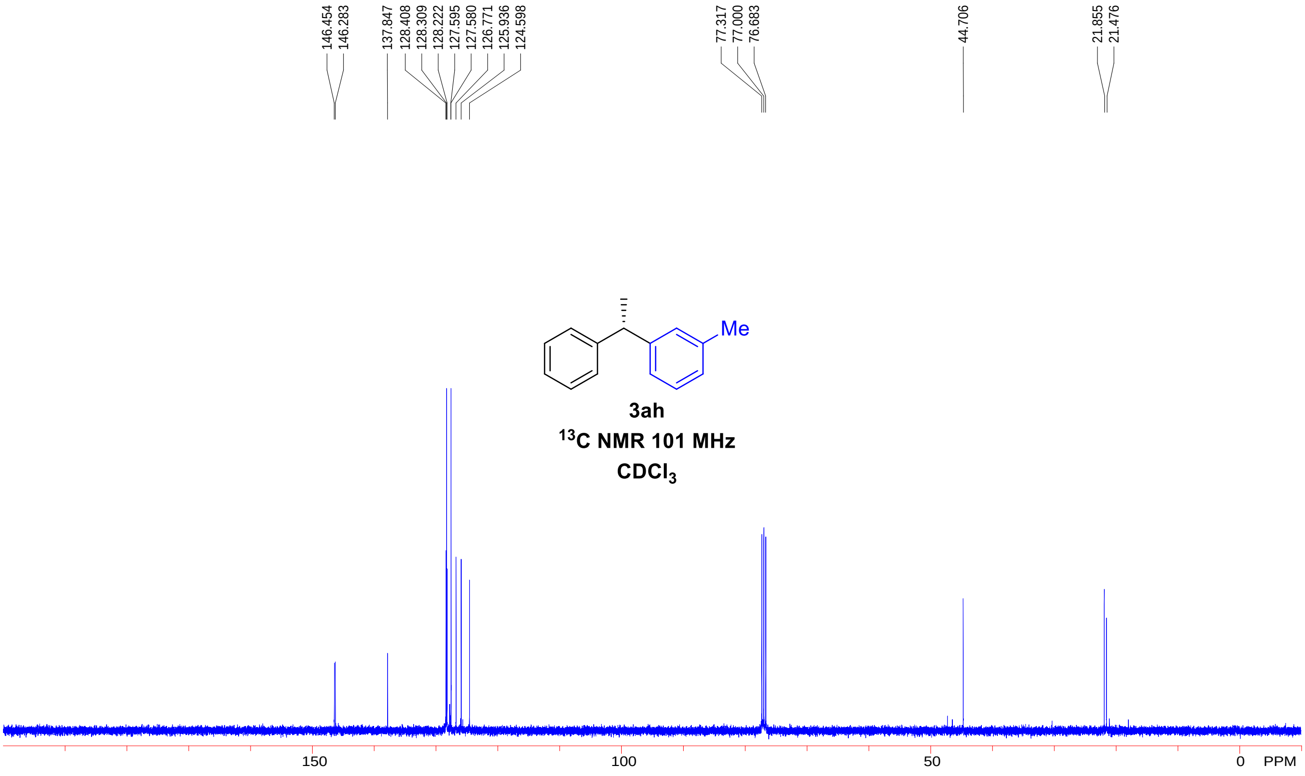

Supplementary Figure 48. ^{13}C NMR spectrum for **3ah**

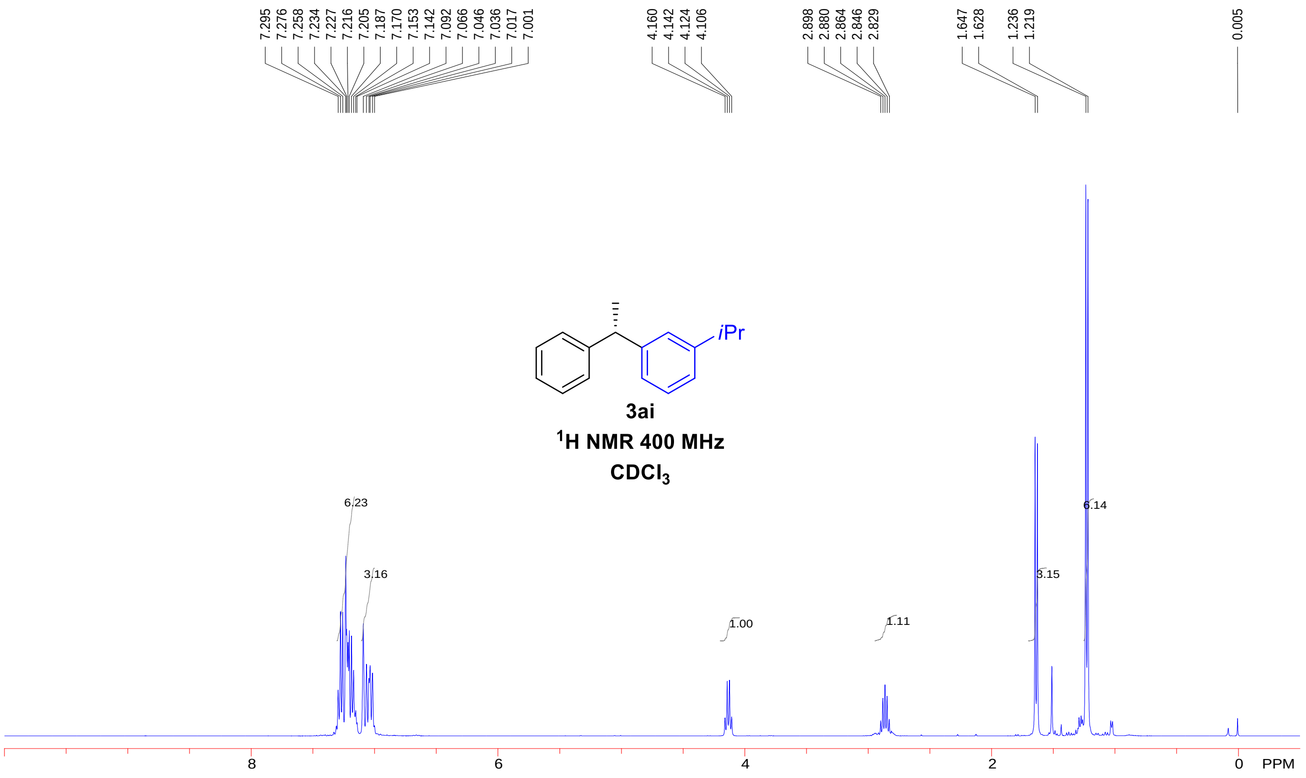

Supplementary Figure 49. ^{1}H NMR spectrum for **3ai**

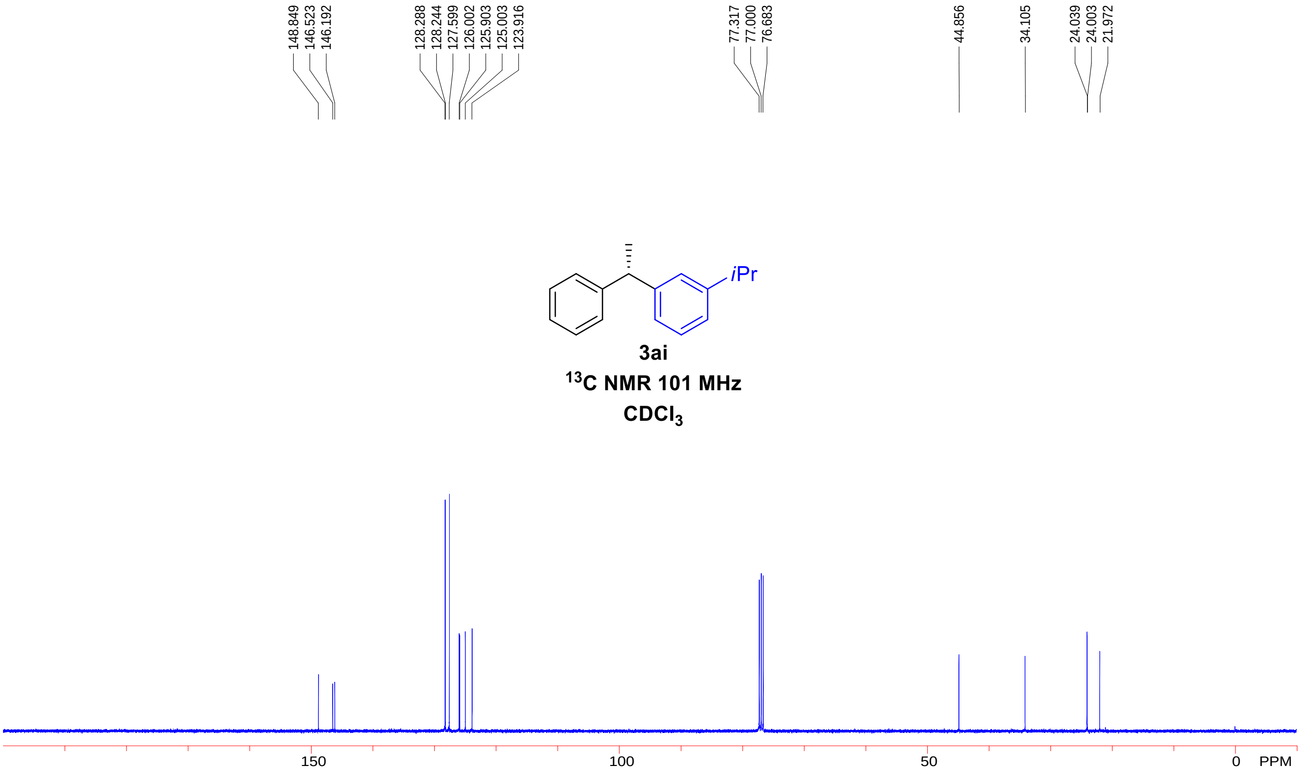

Supplementary Figure 50. ^{13}C NMR spectrum for **3ai**

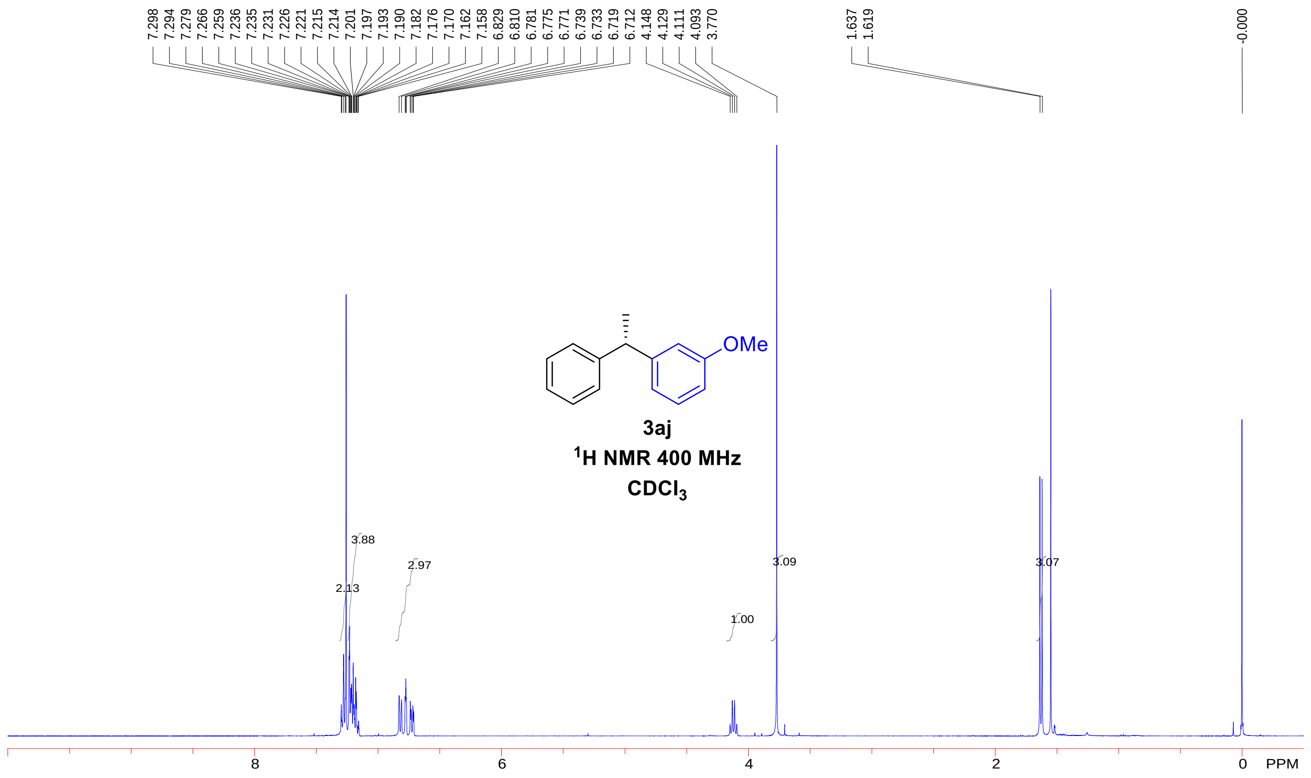

Supplementary Figure 51. ^{1}H NMR spectrum for **3aj**

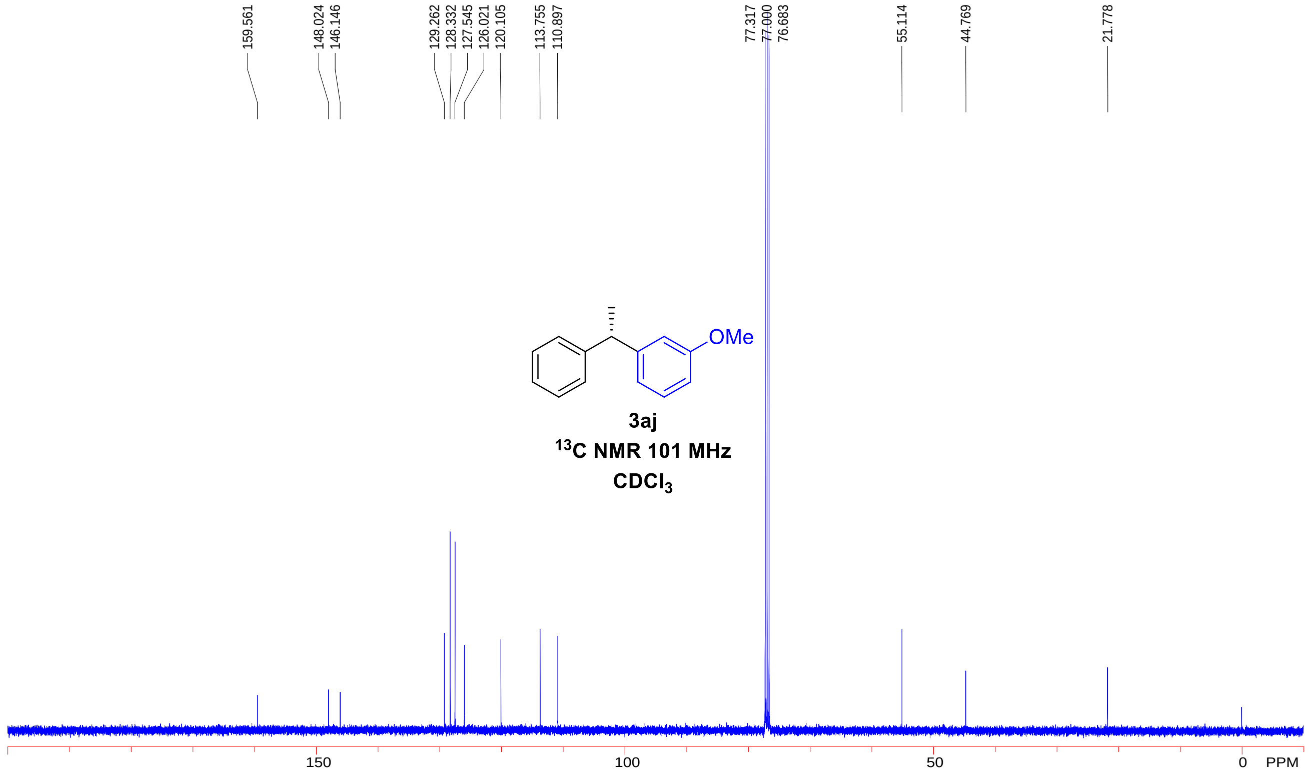

Supplementary Figure 52. ^{13}C NMR spectrum for **3aj**

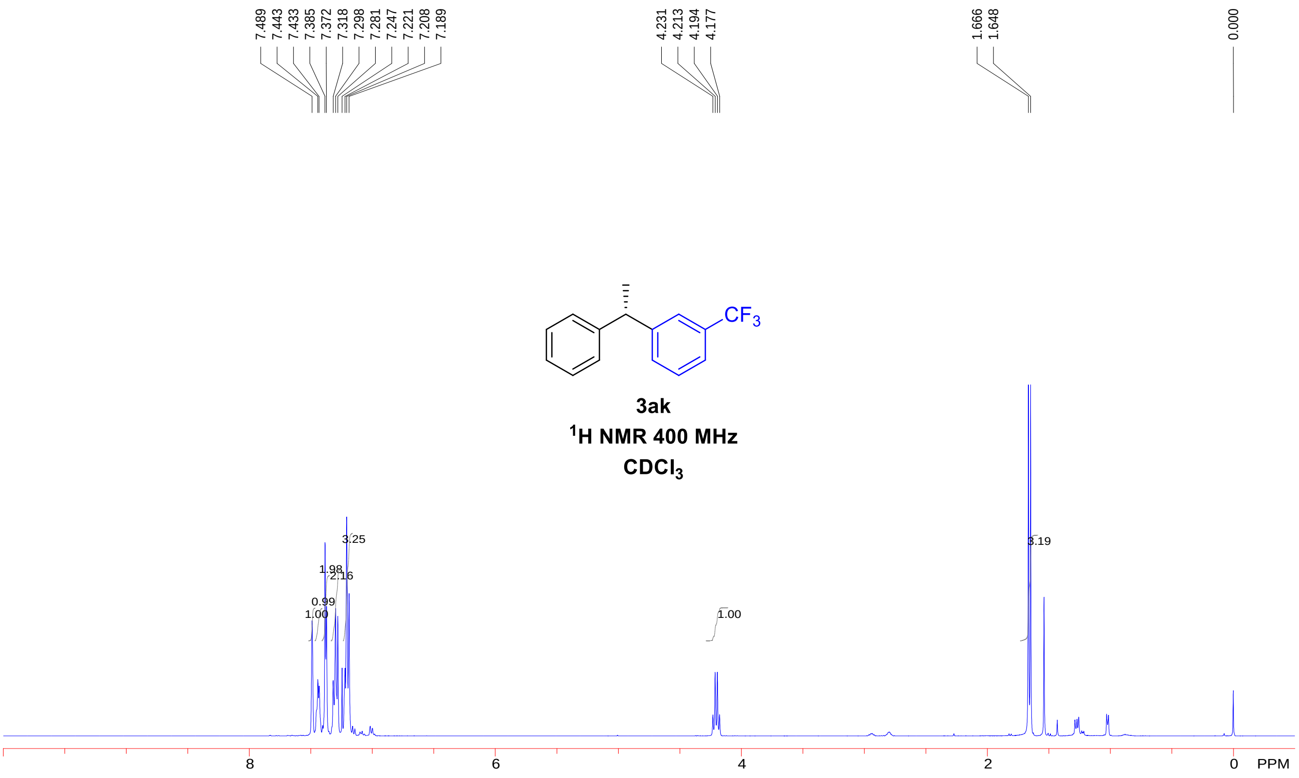

Supplementary Figure 53. ^{1}H NMR spectrum for **3ak**

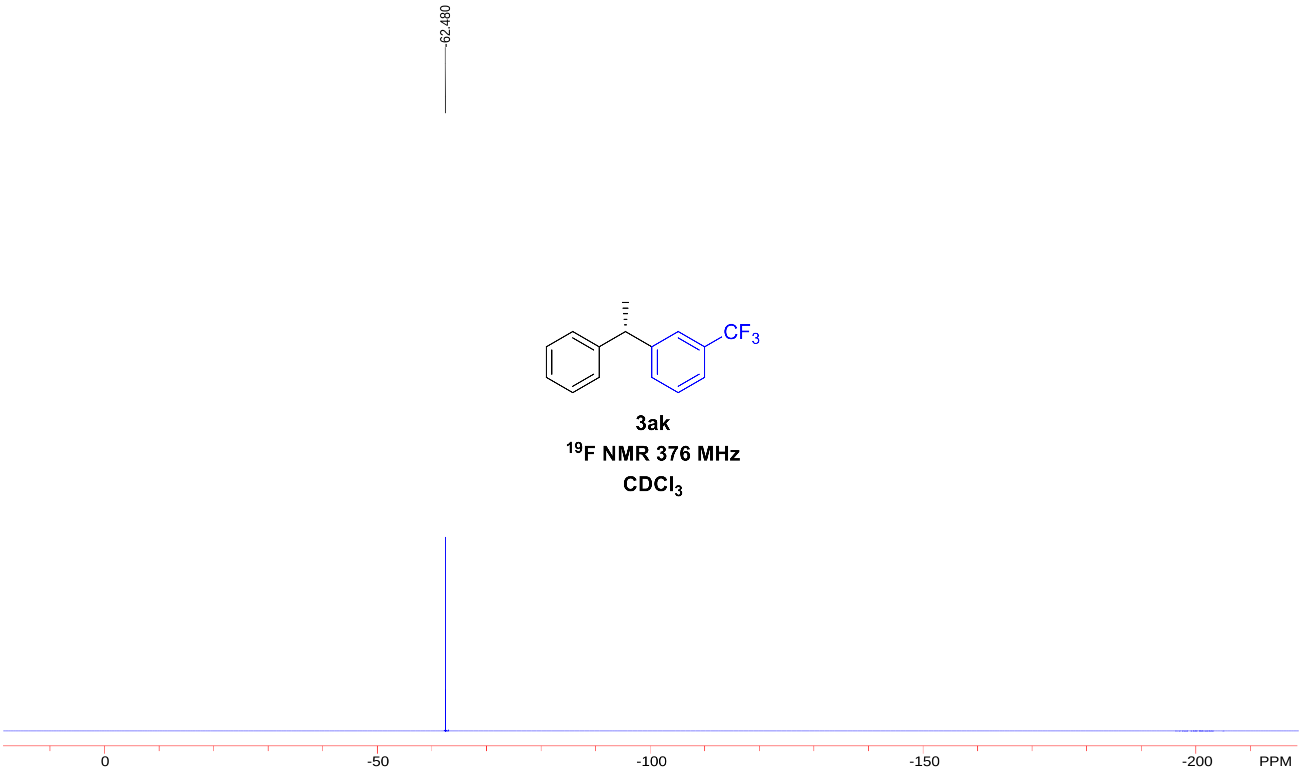

Supplementary Figure 54. ^{19}F NMR spectrum for **3ak**

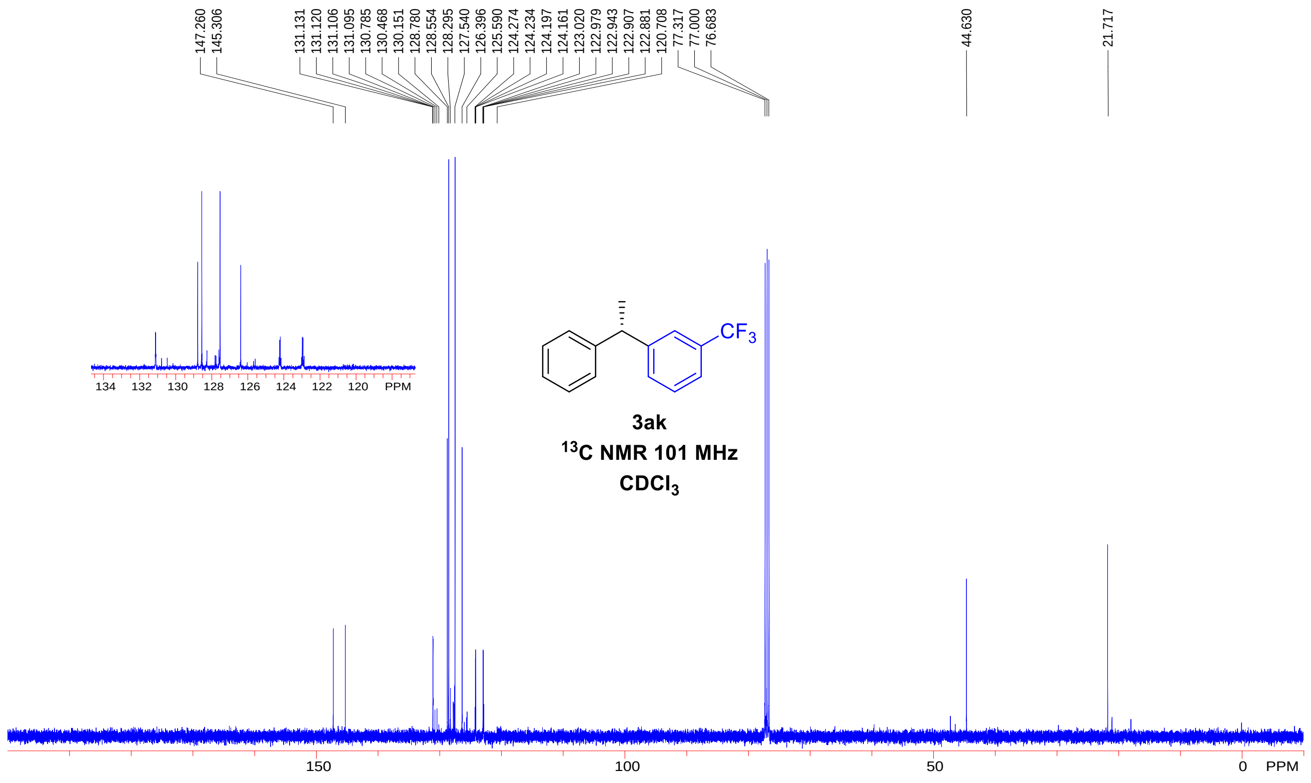

Supplementary Figure 55. ^{13}C NMR spectrum for **3ak**

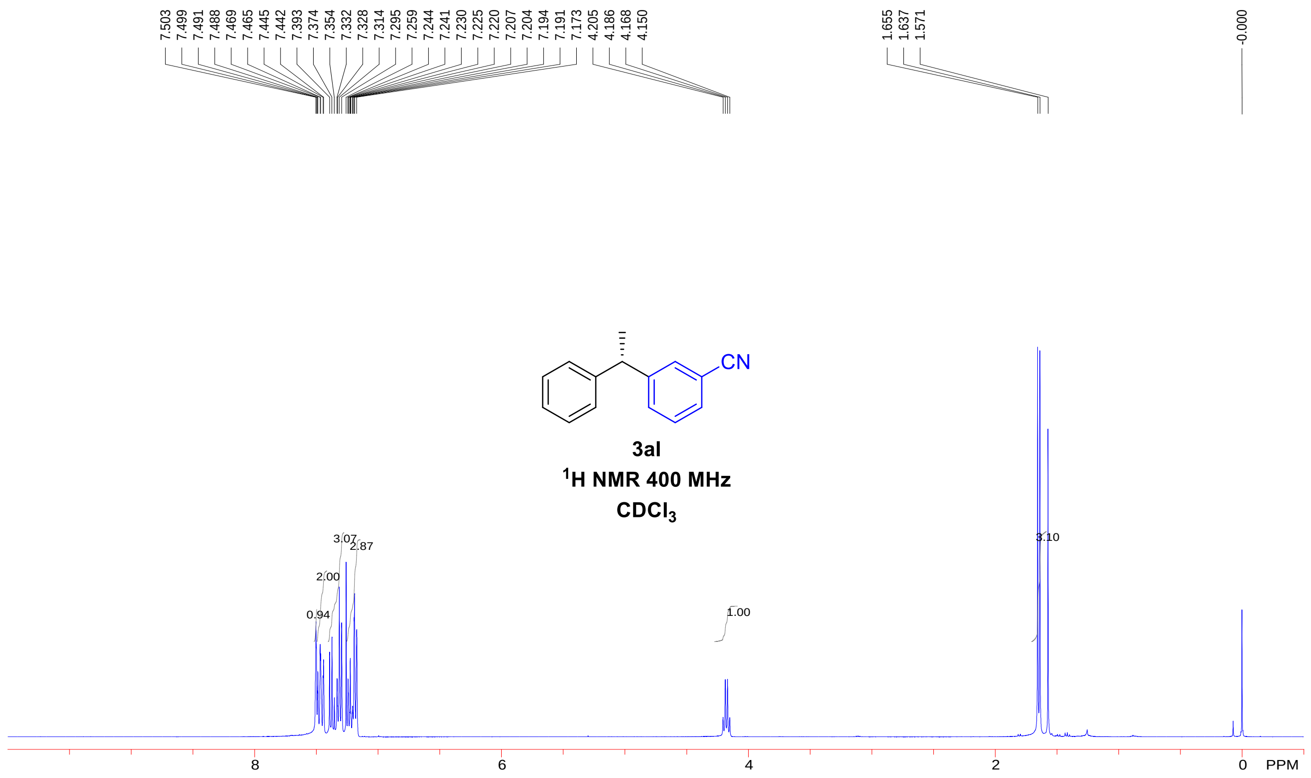

Supplementary Figure 56. ^{1}H NMR spectrum for **3al**

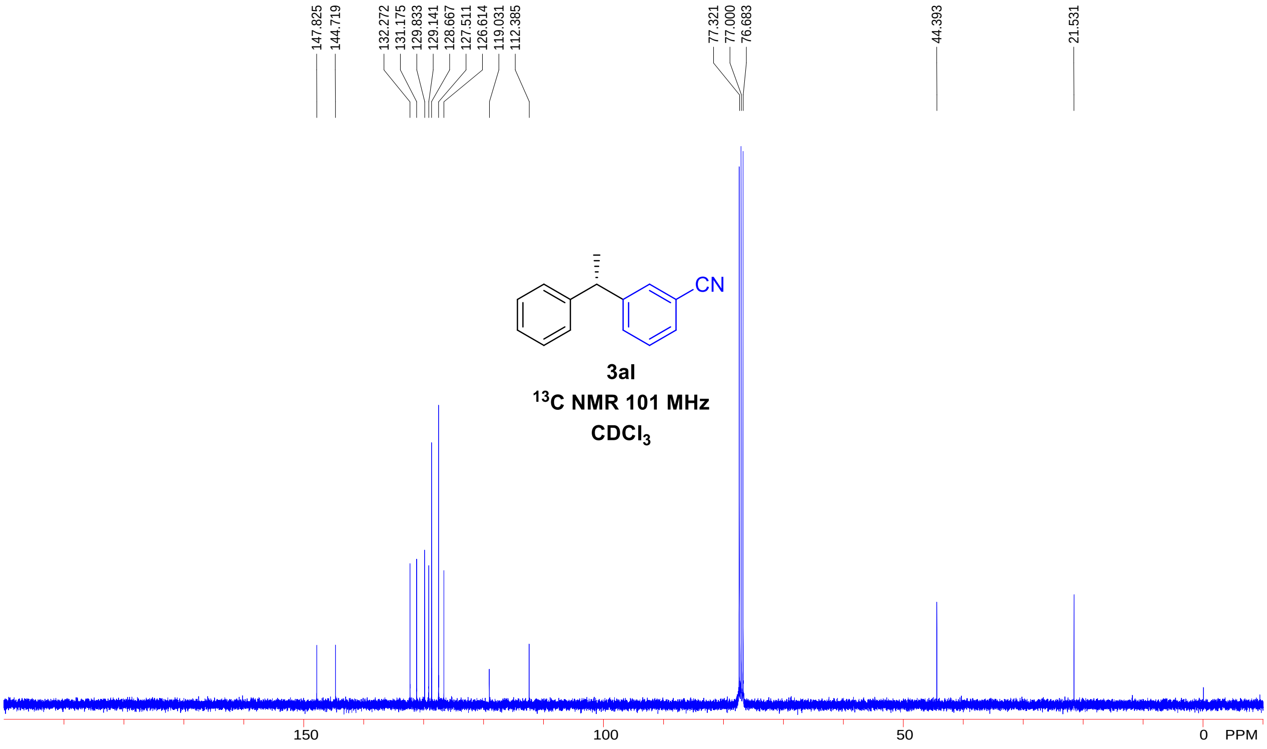

Supplementary Figure 57. ^{13}C NMR spectrum for **3al**

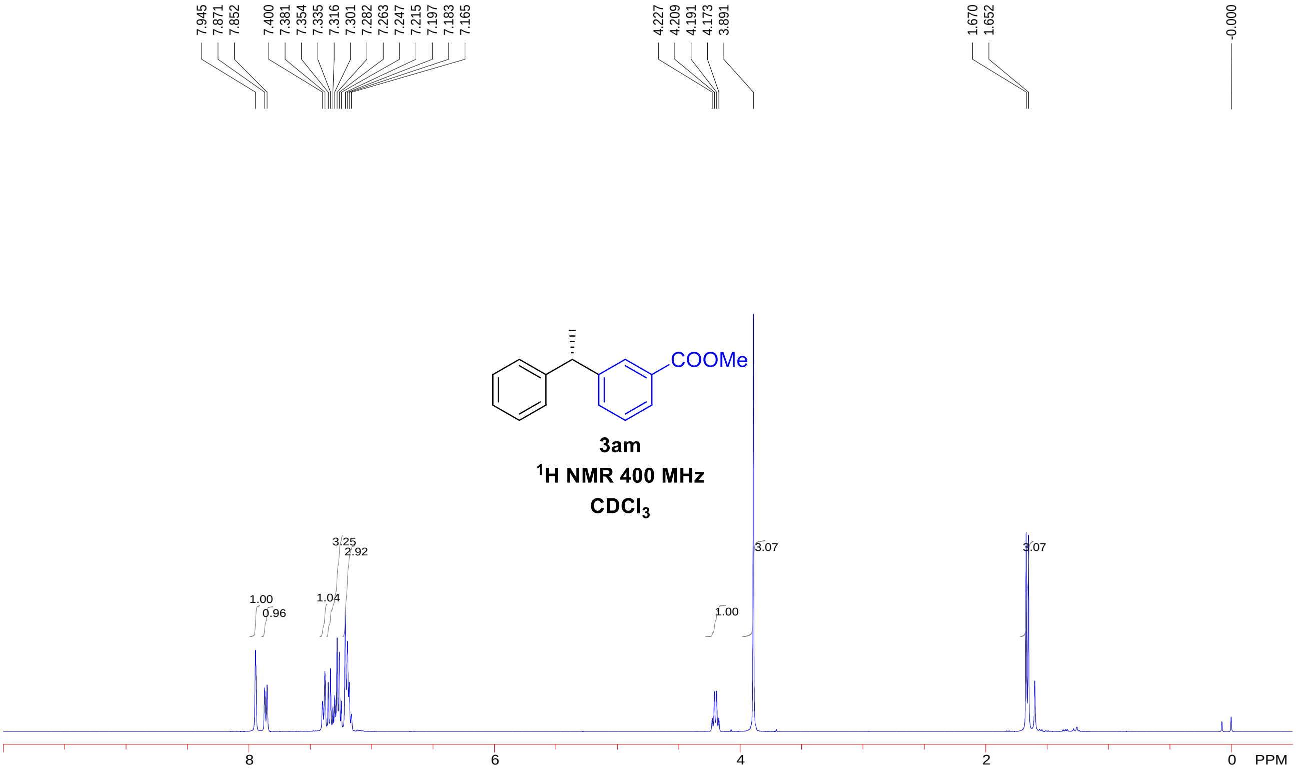

Supplementary Figure 58. ^{1}H NMR spectrum for **3am**

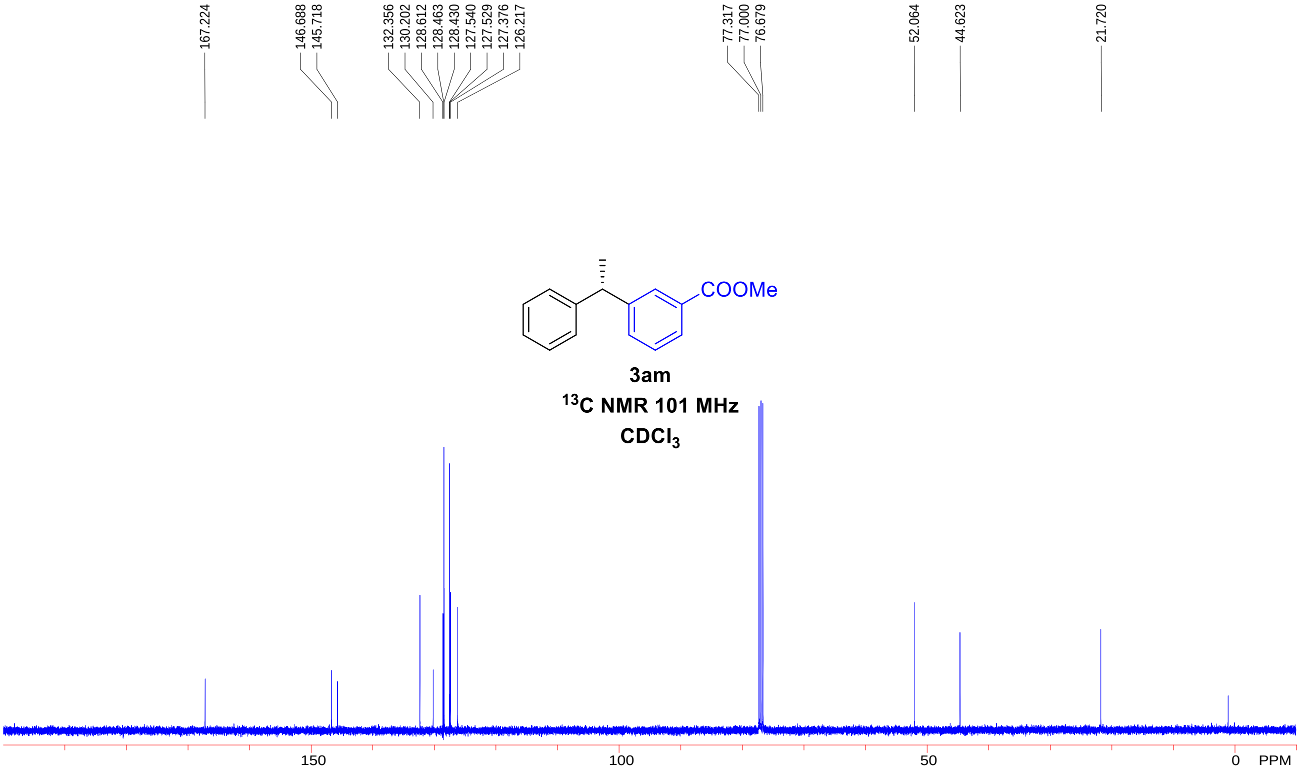

Supplementary Figure 59. ^{13}C NMR spectrum for **3am**

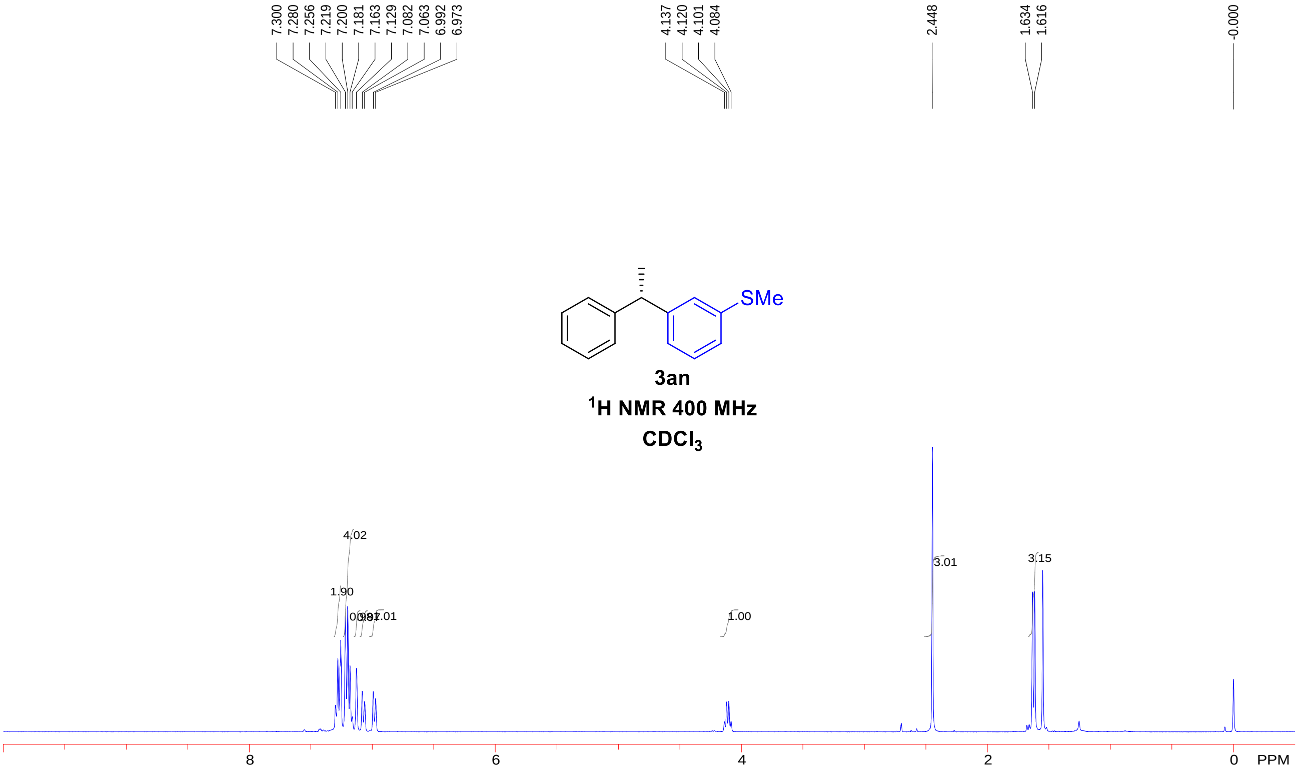

Supplementary Figure 60. ^{1}H NMR spectrum for **3an**

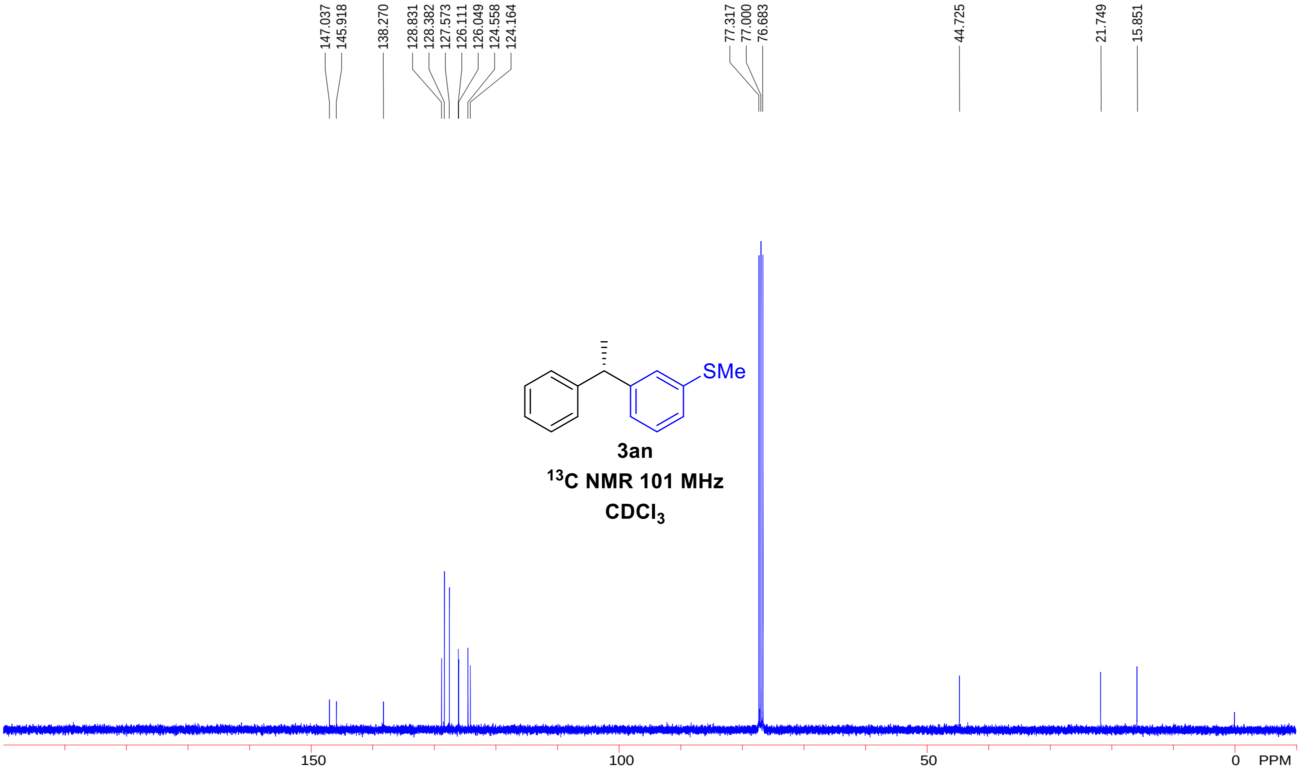

Supplementary Figure 61. ^{13}C NMR spectrum for **3an**

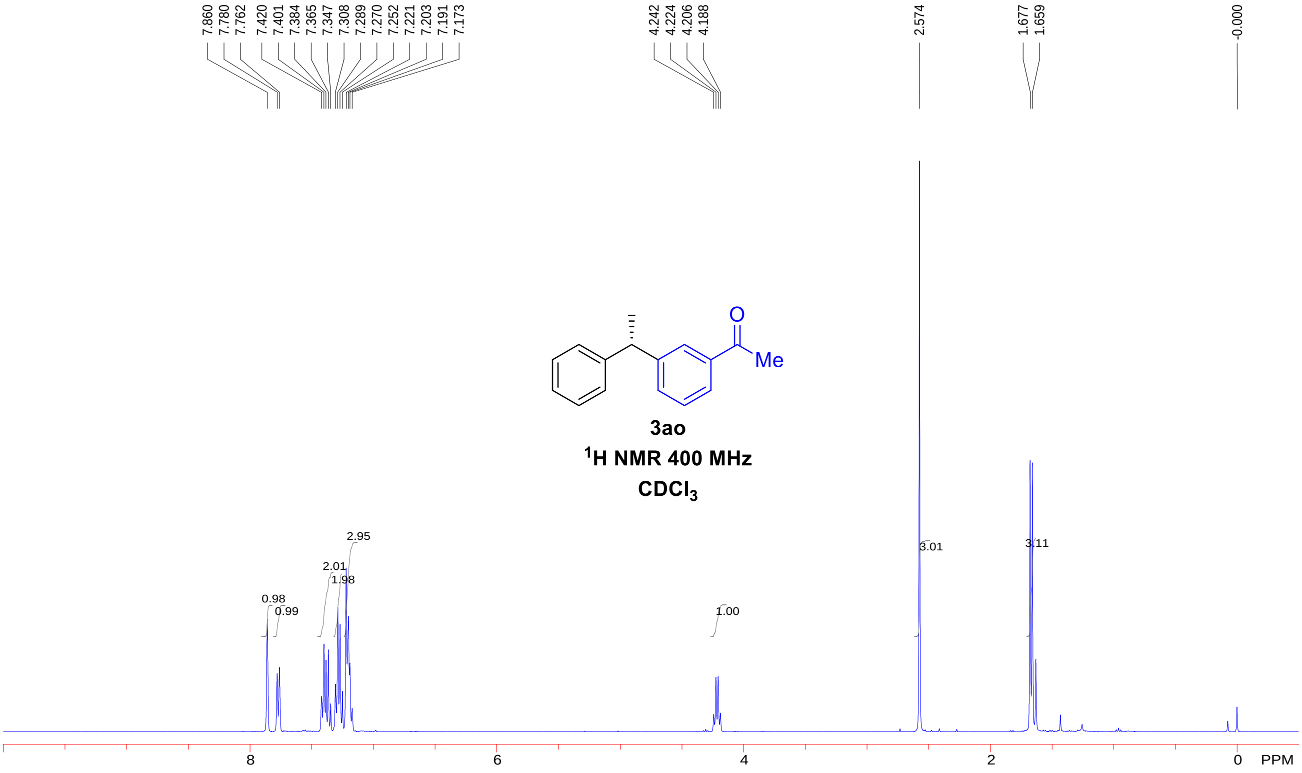

Supplementary Figure 62. ^{1}H NMR spectrum for **3ao**

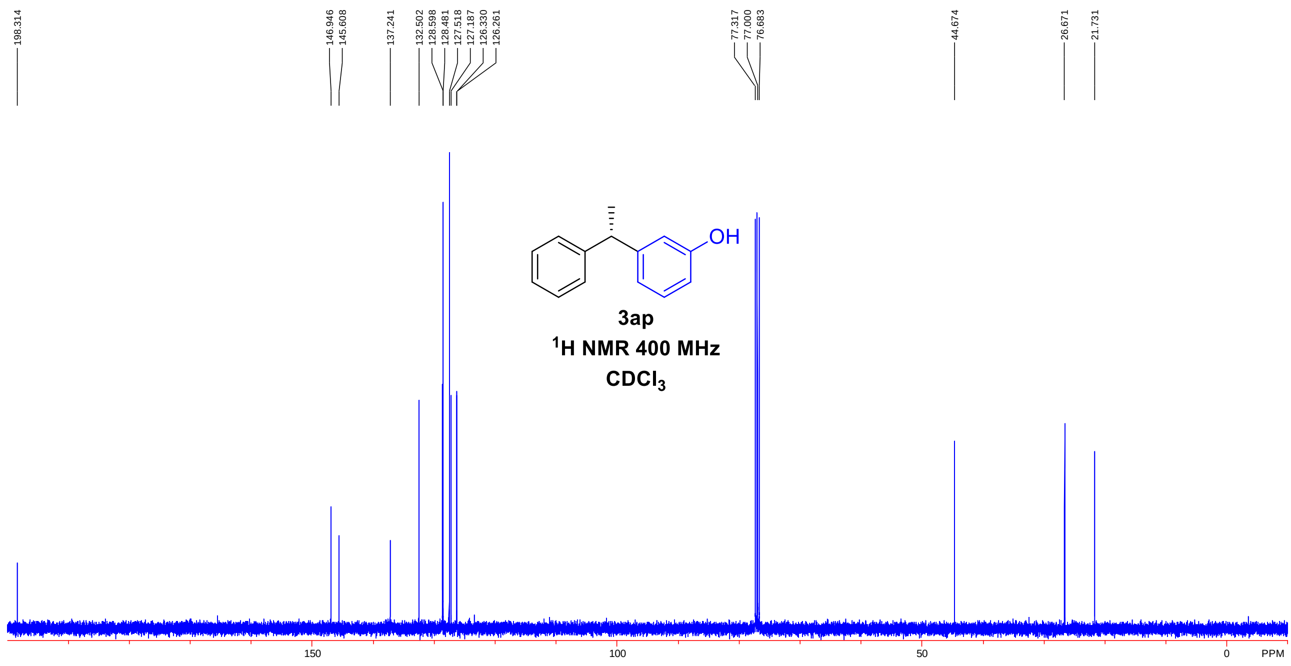

Supplementary Figure 63. ^{13}C NMR spectrum for **3ao**

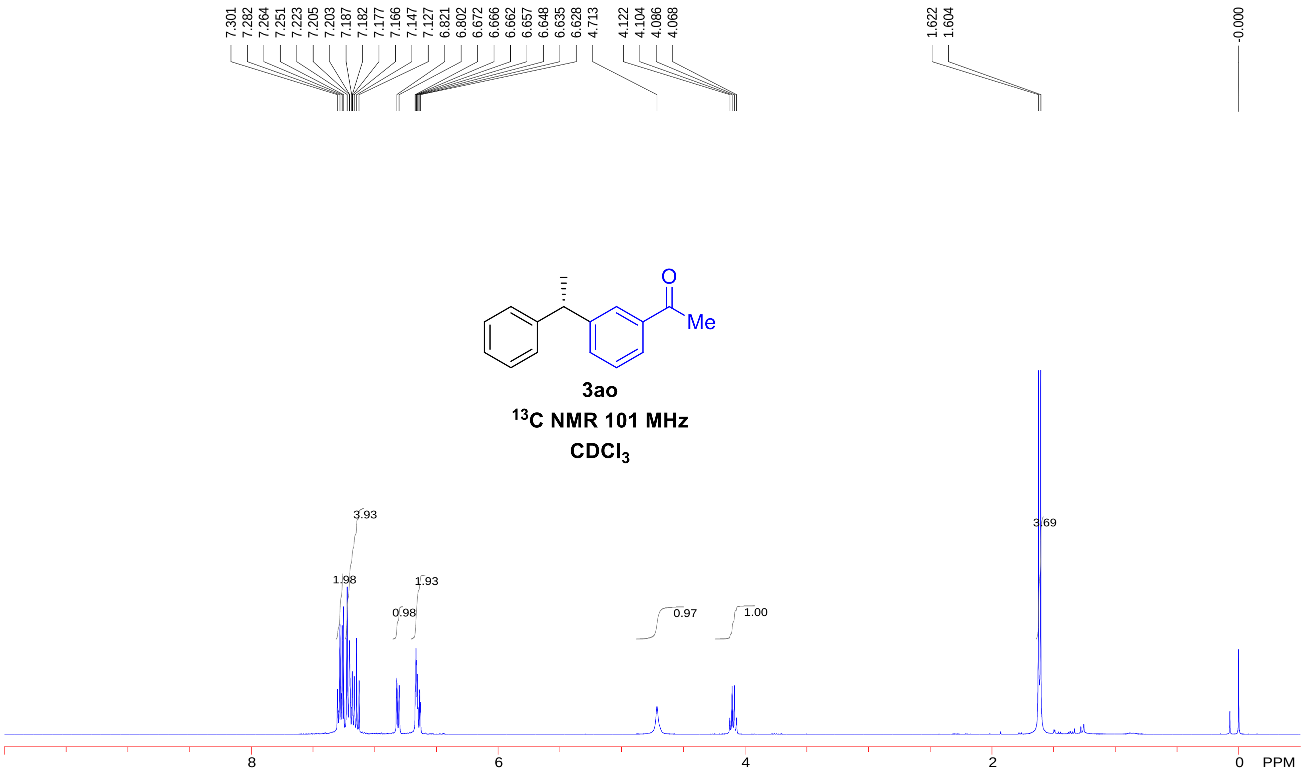

Supplementary Figure 64. ^{1}H NMR spectrum for **3ap**

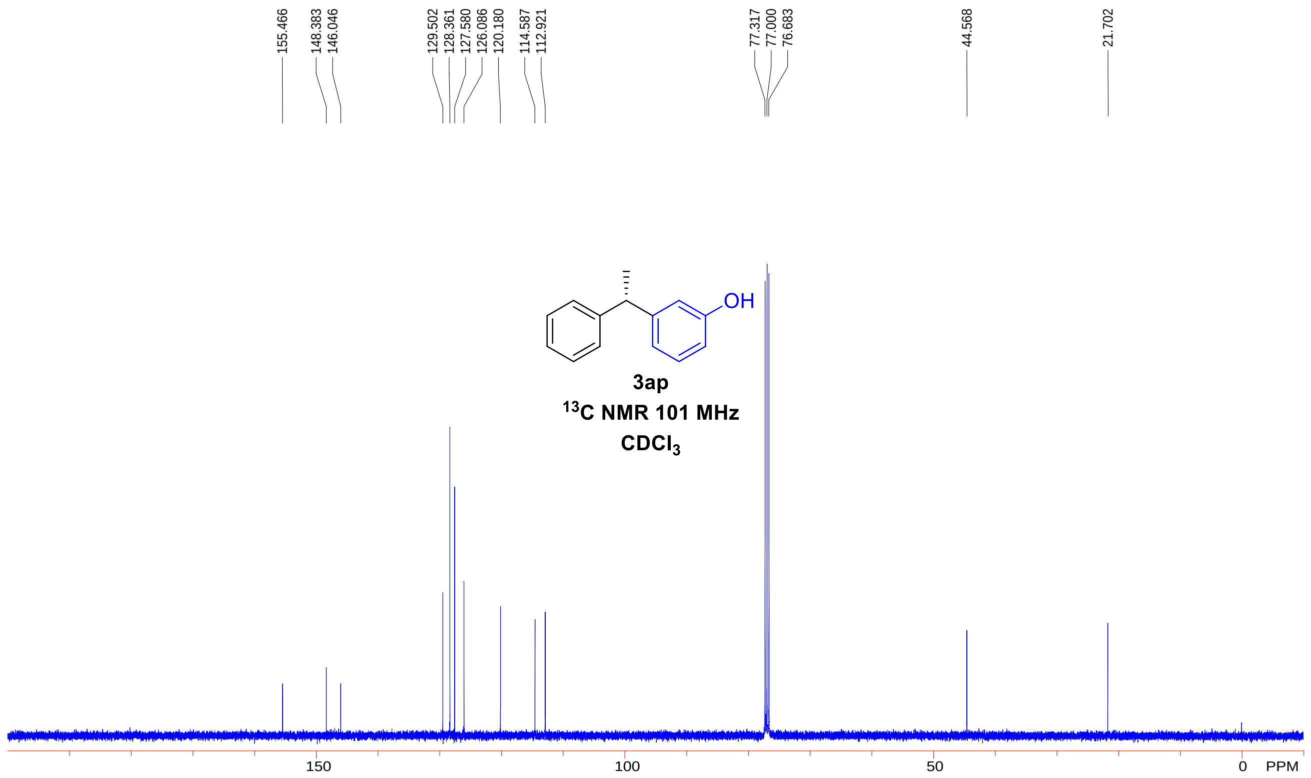

Supplementary Figure 65. ^{13}C NMR spectrum for **3ap**

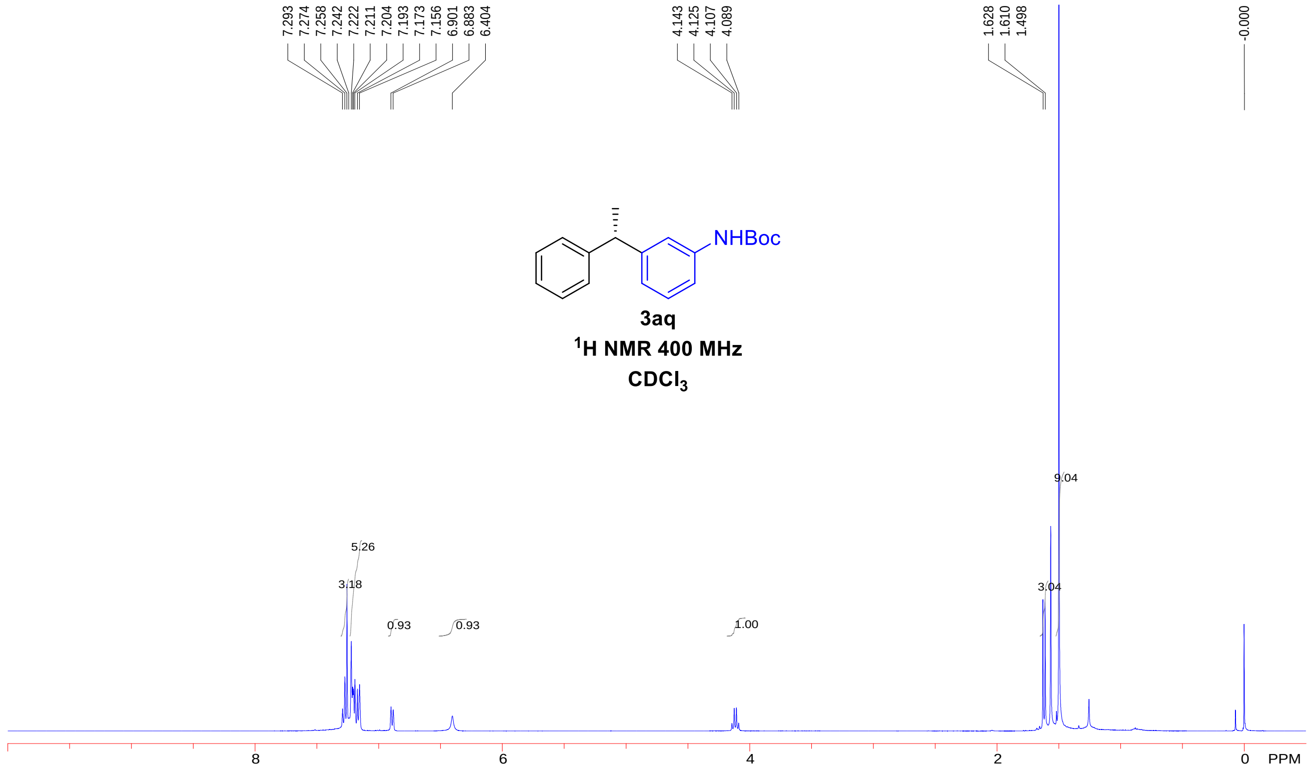

Supplementary Figure 66. ^{1}H NMR spectrum for **3aq**

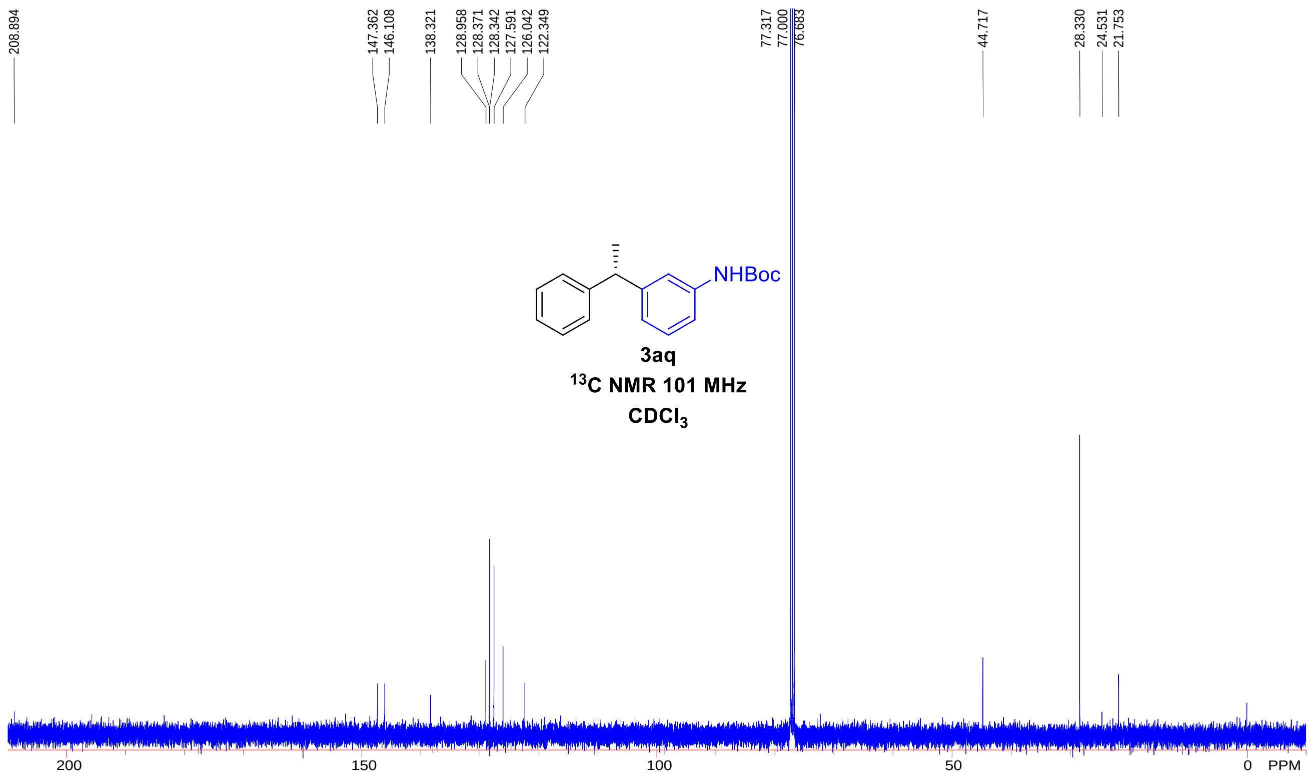

Supplementary Figure 67. ^{13}C NMR spectrum for **3aq**

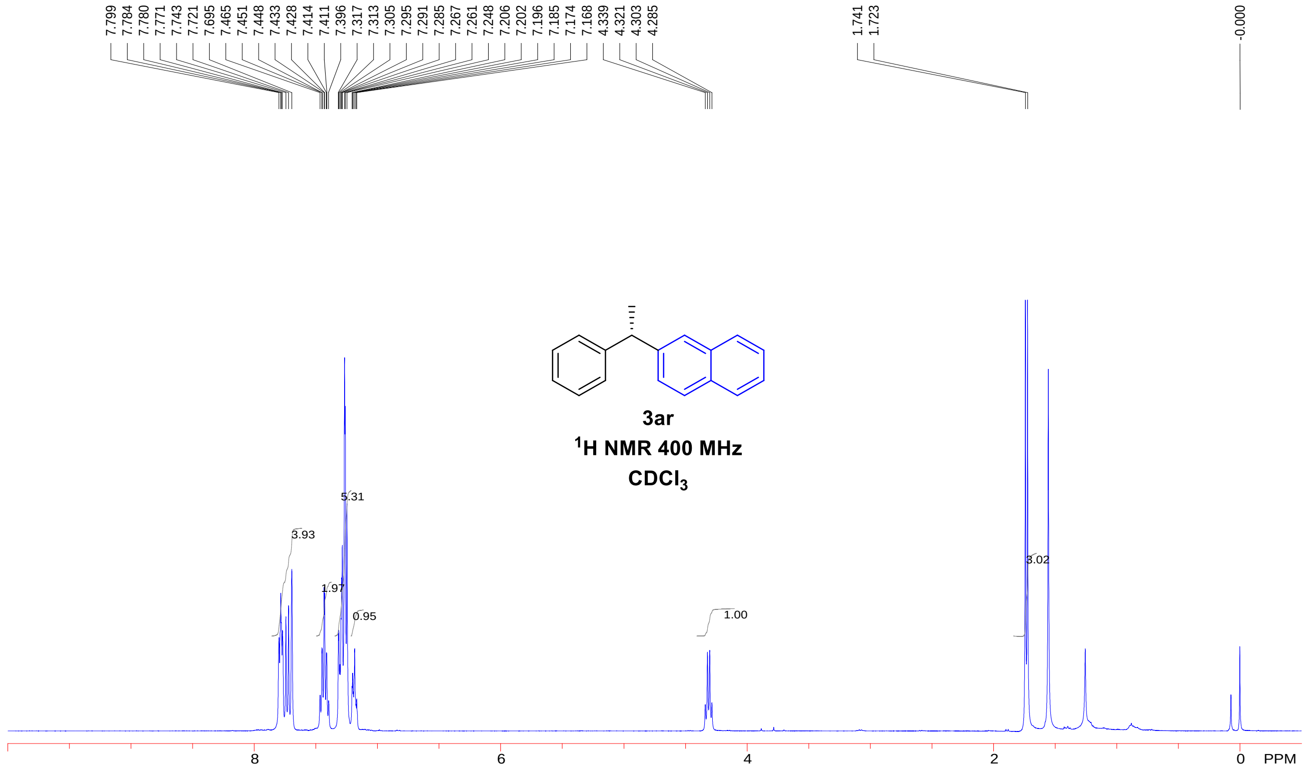

Supplementary Figure 68. ^{1}H NMR spectrum for **3ar**

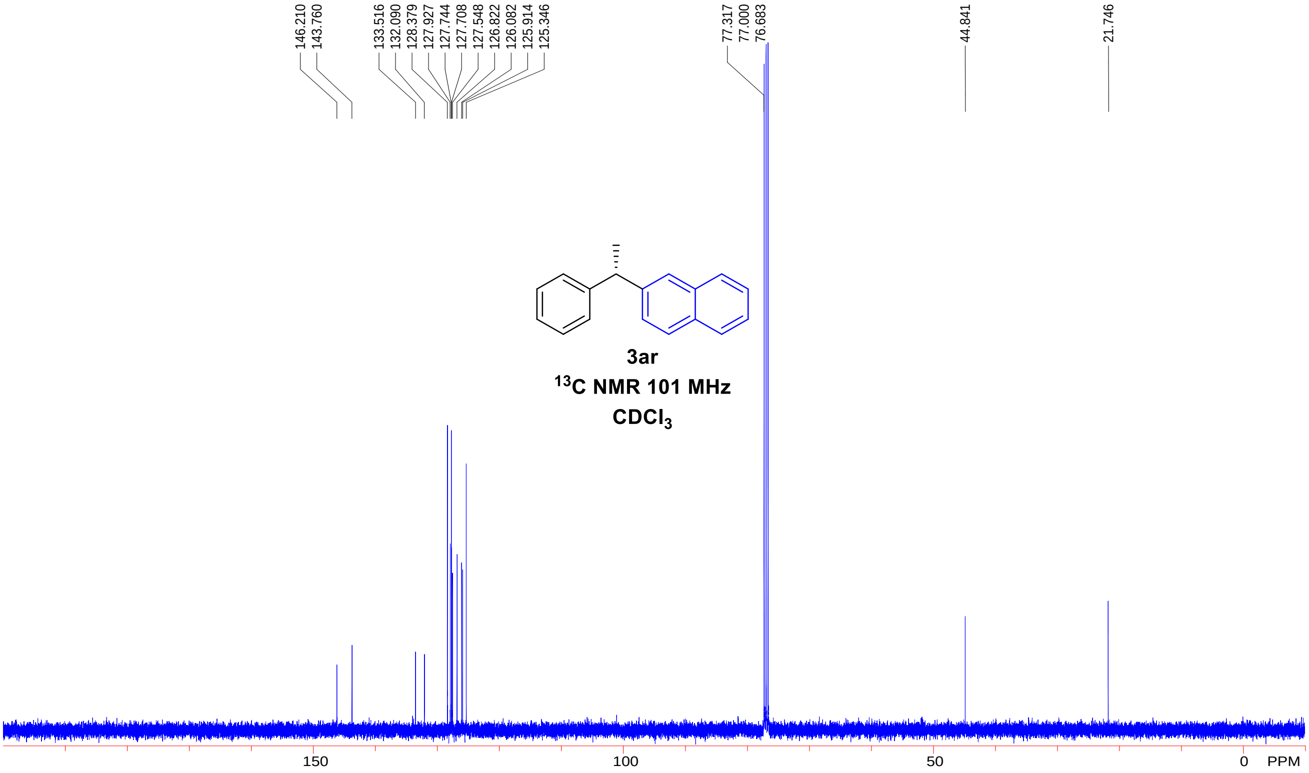

Supplementary Figure 69. ^{13}C NMR spectrum for **3ar**

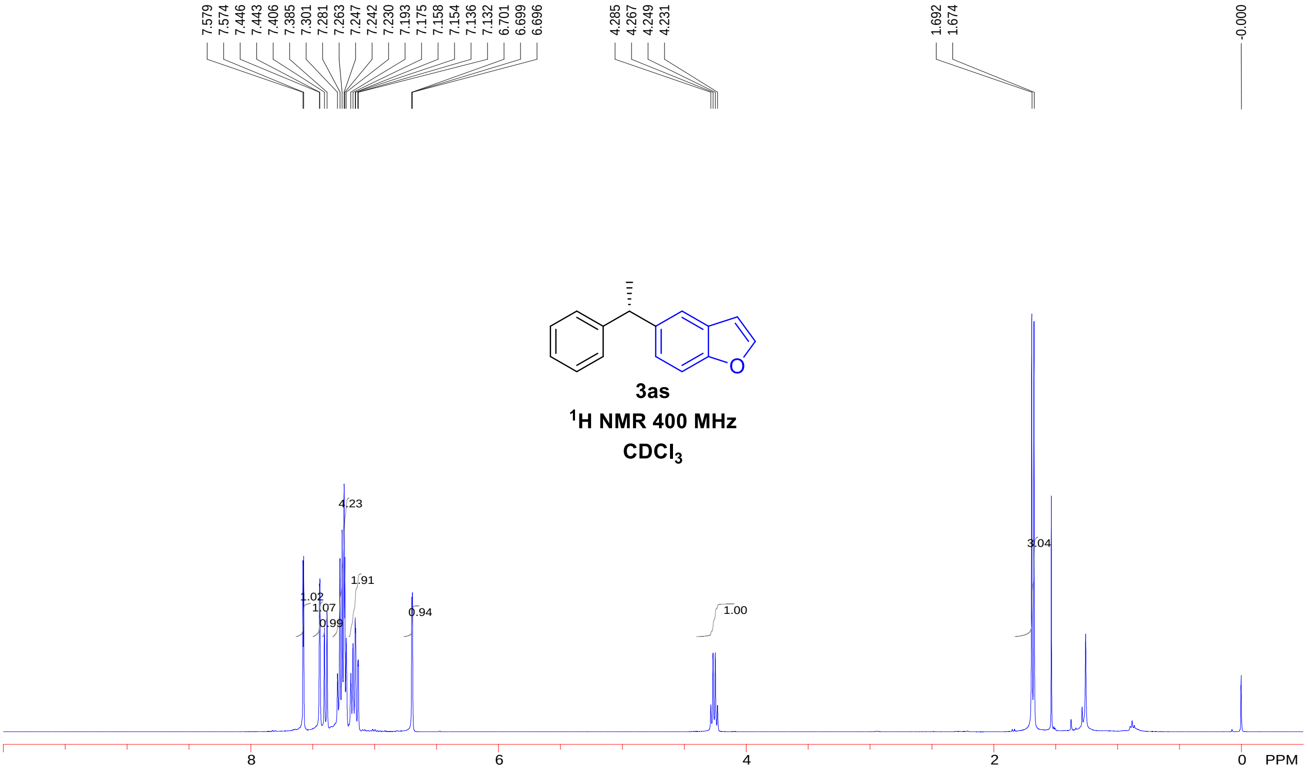

Supplementary Figure 70. ^{1}H NMR spectrum for **3as**

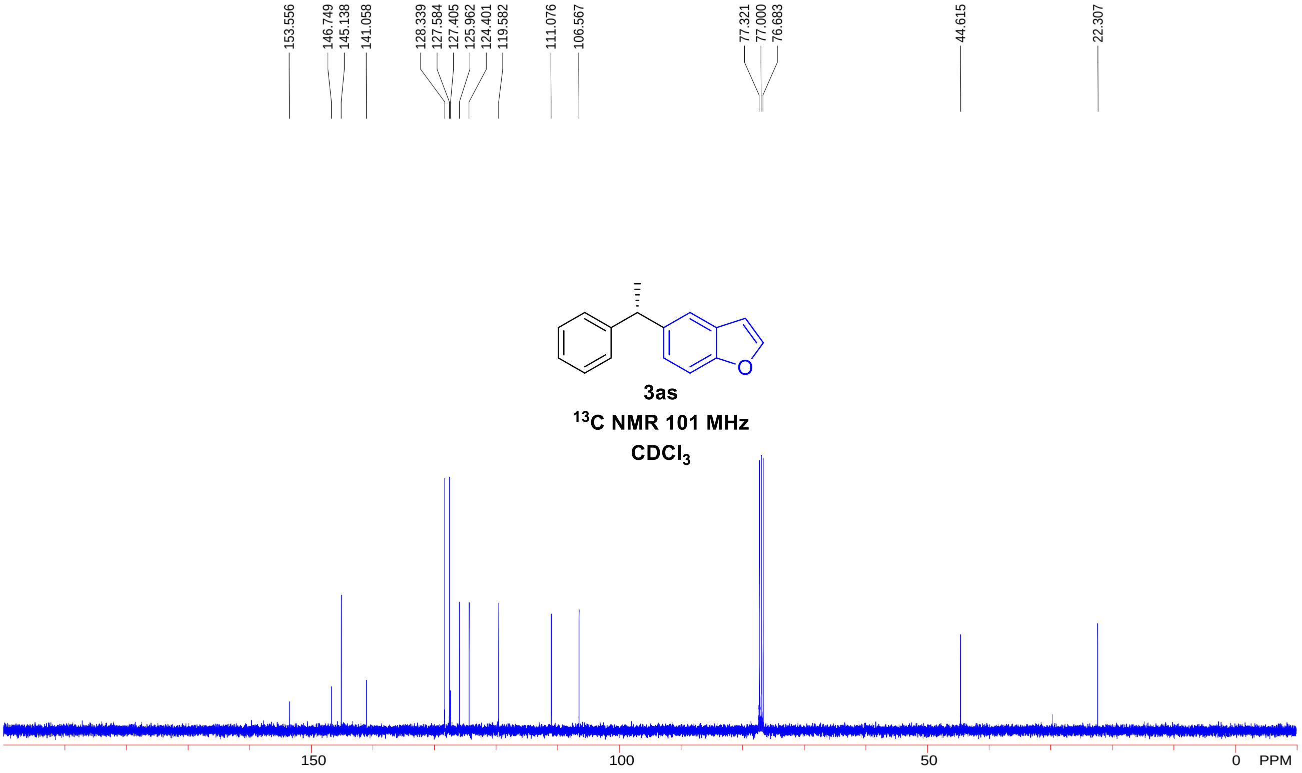

Supplementary Figure 71. ^{13}C NMR spectrum for **3as**

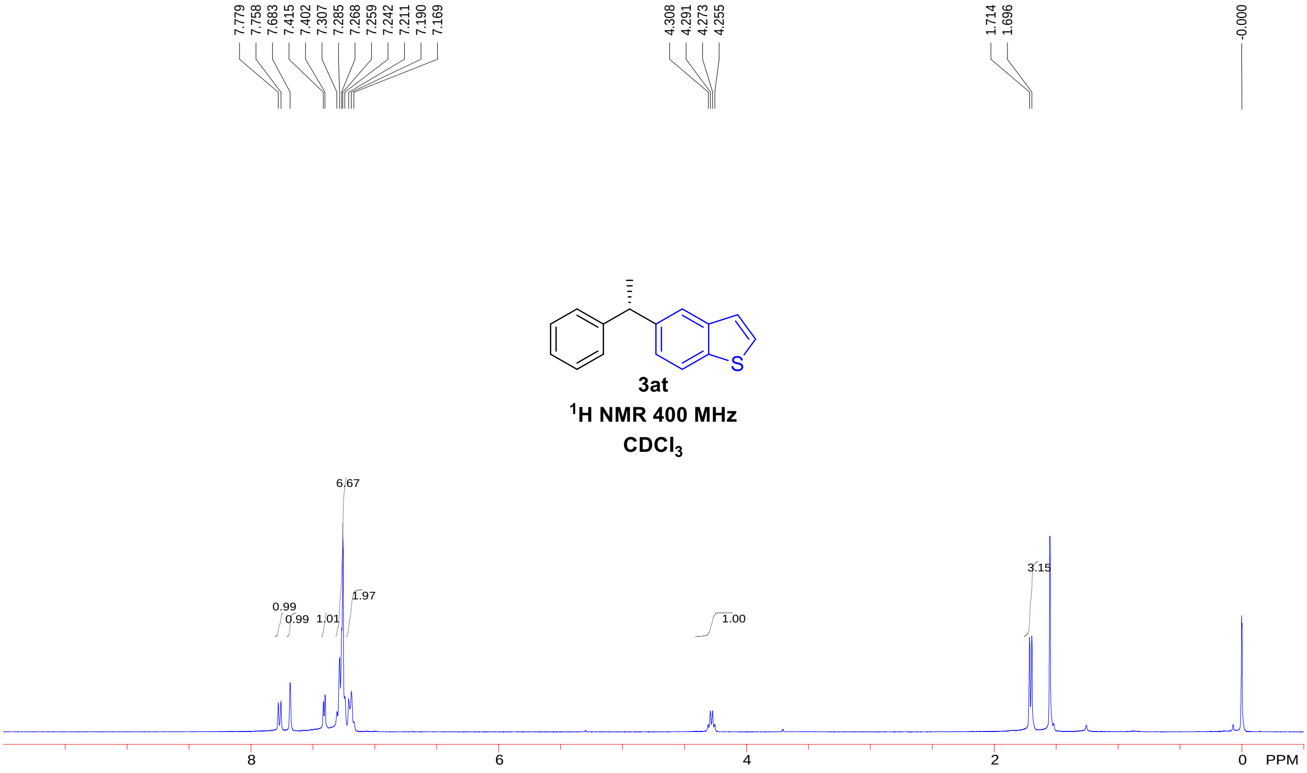

Supplementary Figure 72. ^{1}H NMR spectrum for **3at**

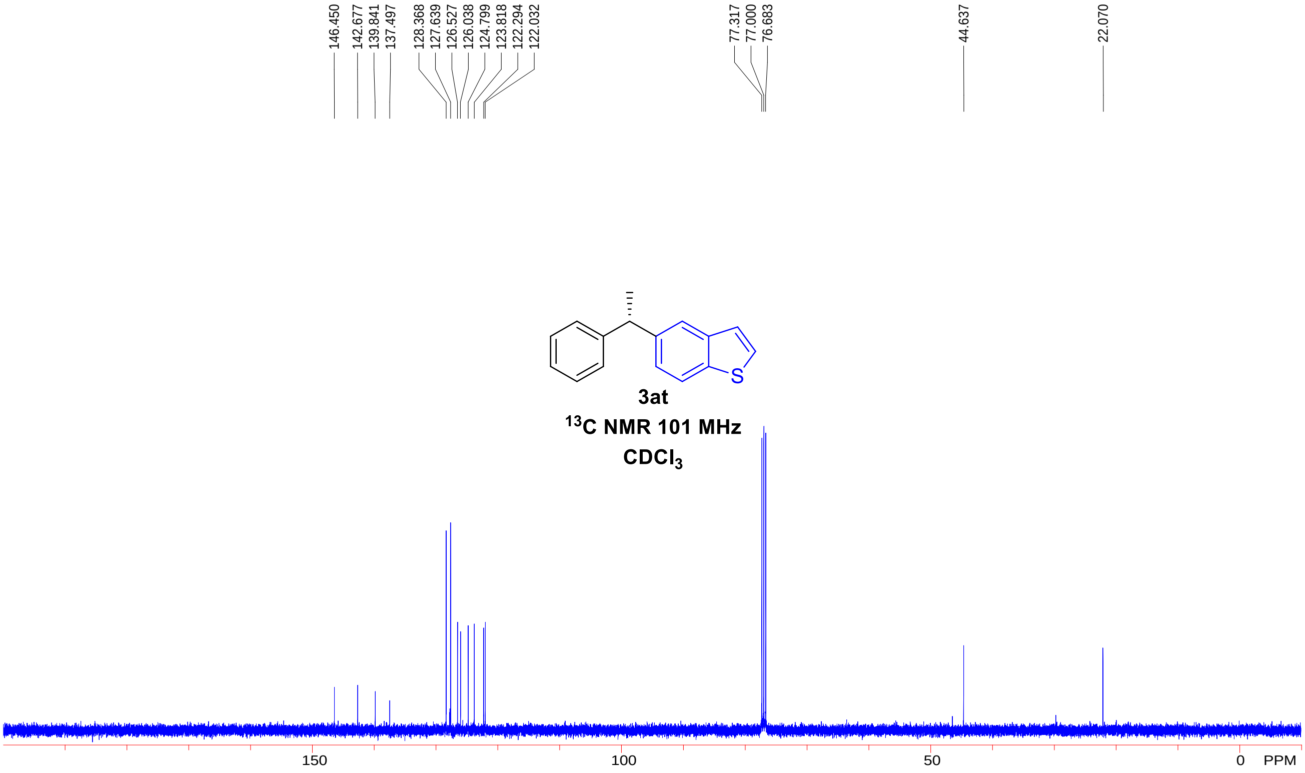

Supplementary Figure 73. ^{13}C NMR spectrum for **3at**

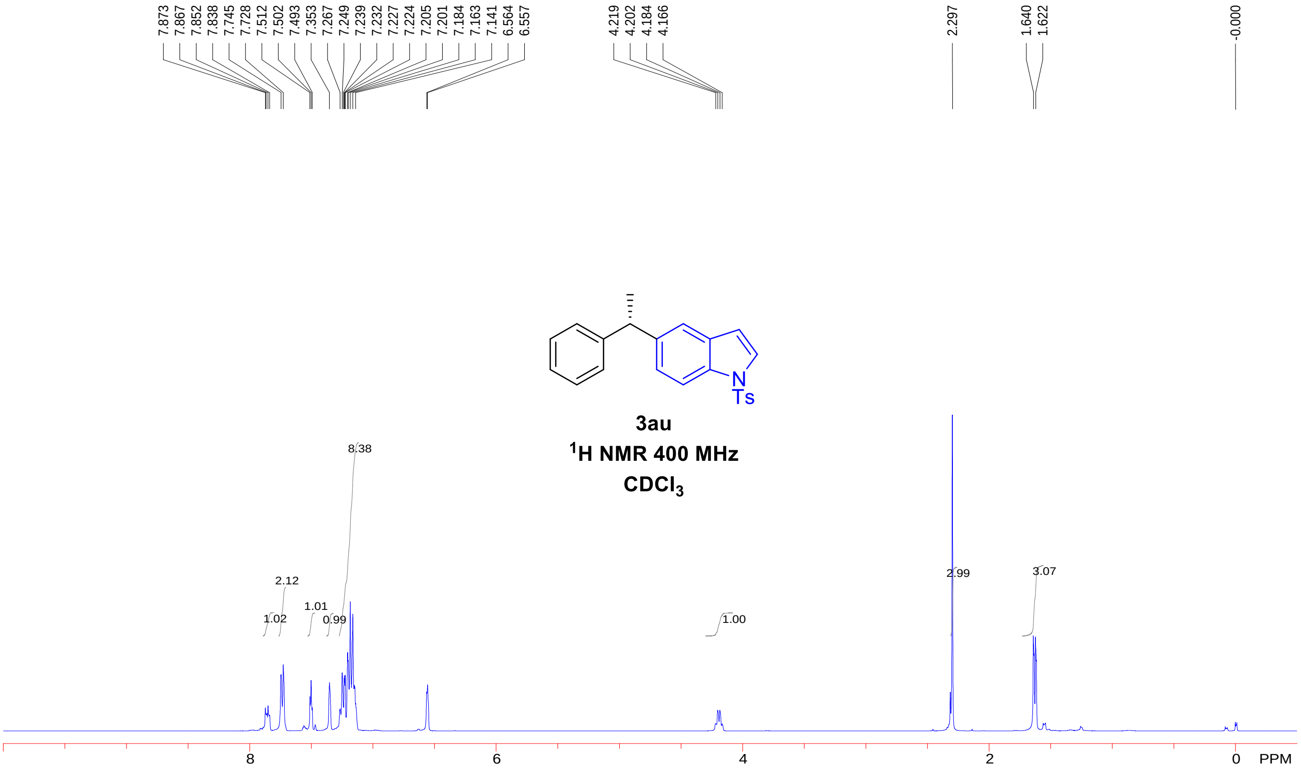

Supplementary Figure 74. ^{1}H NMR spectrum for **3au**

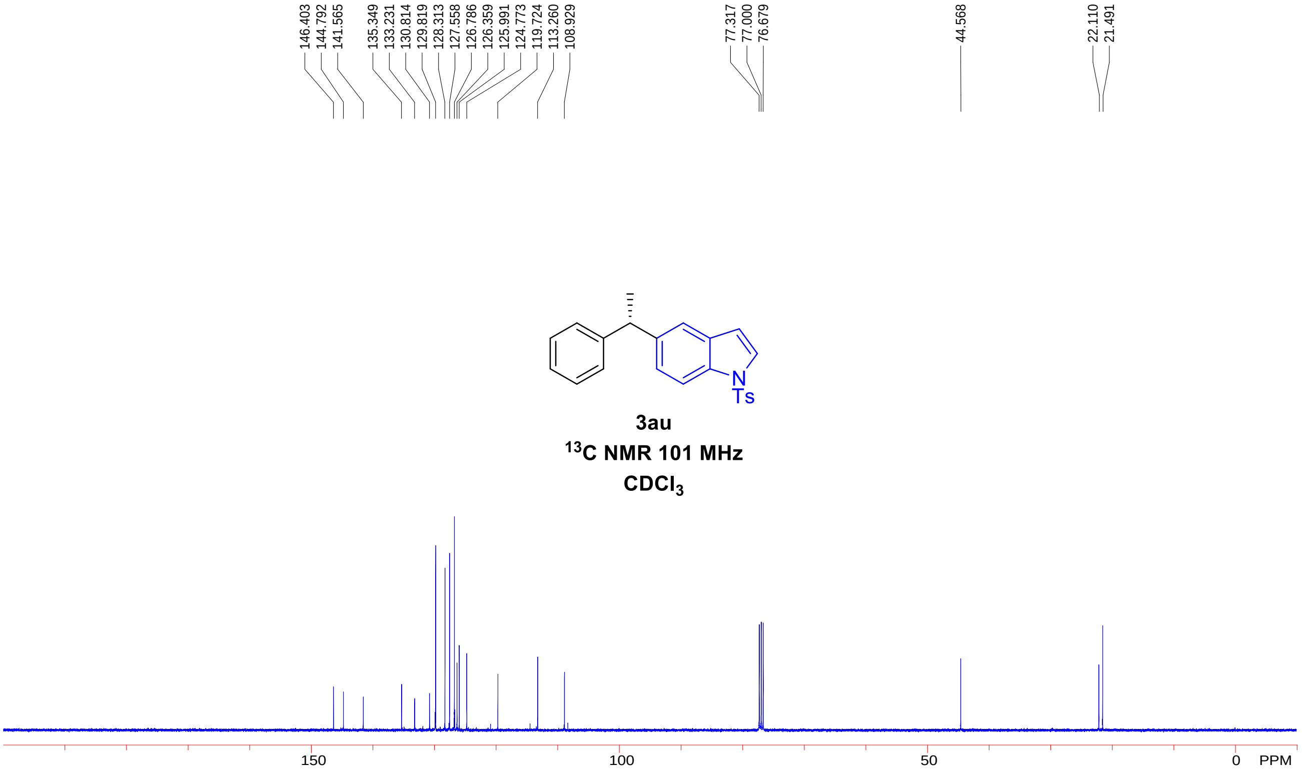

Supplementary Figure 75. ^{13}C NMR spectrum for **3au**

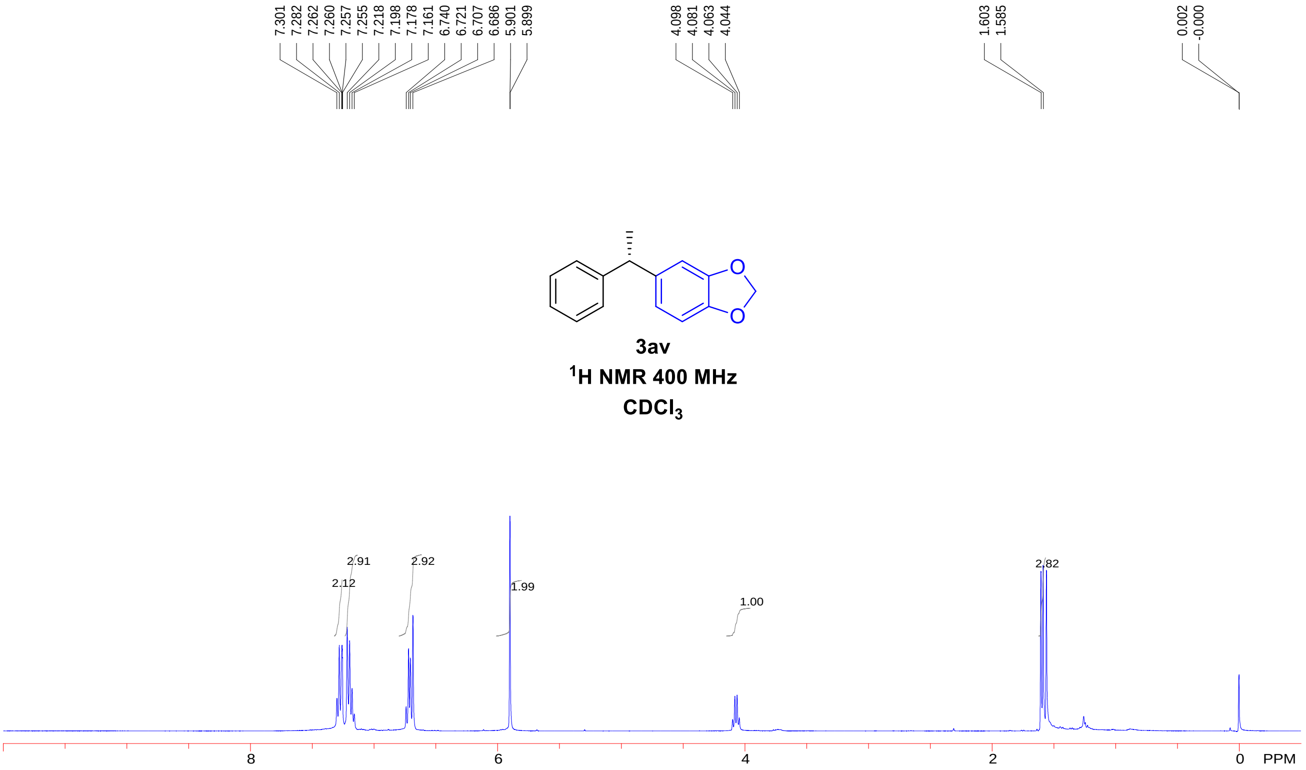

Supplementary Figure 76. ^{1}H NMR spectrum for **3av**

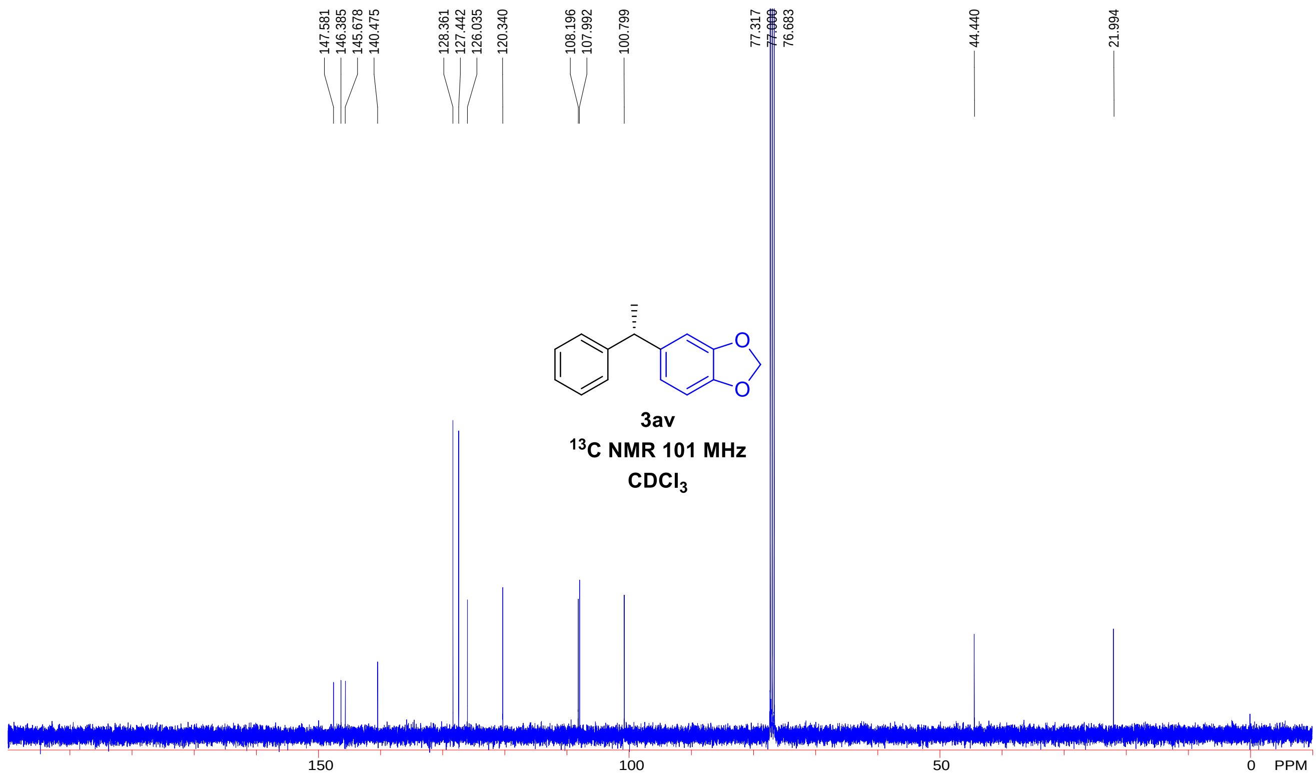

Supplementary Figure 77. ^{13}C NMR spectrum for **3av**

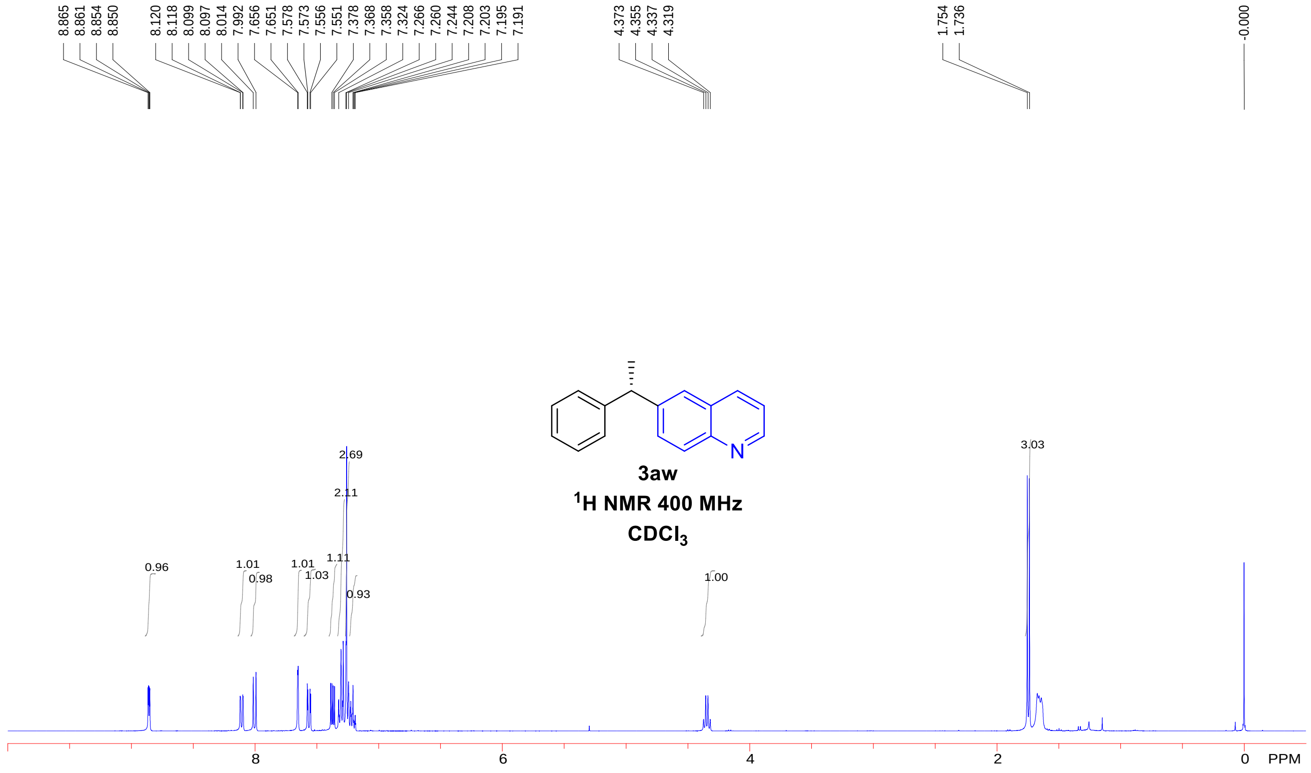

Supplementary Figure 78. ^{1}H NMR spectrum for **3aw**

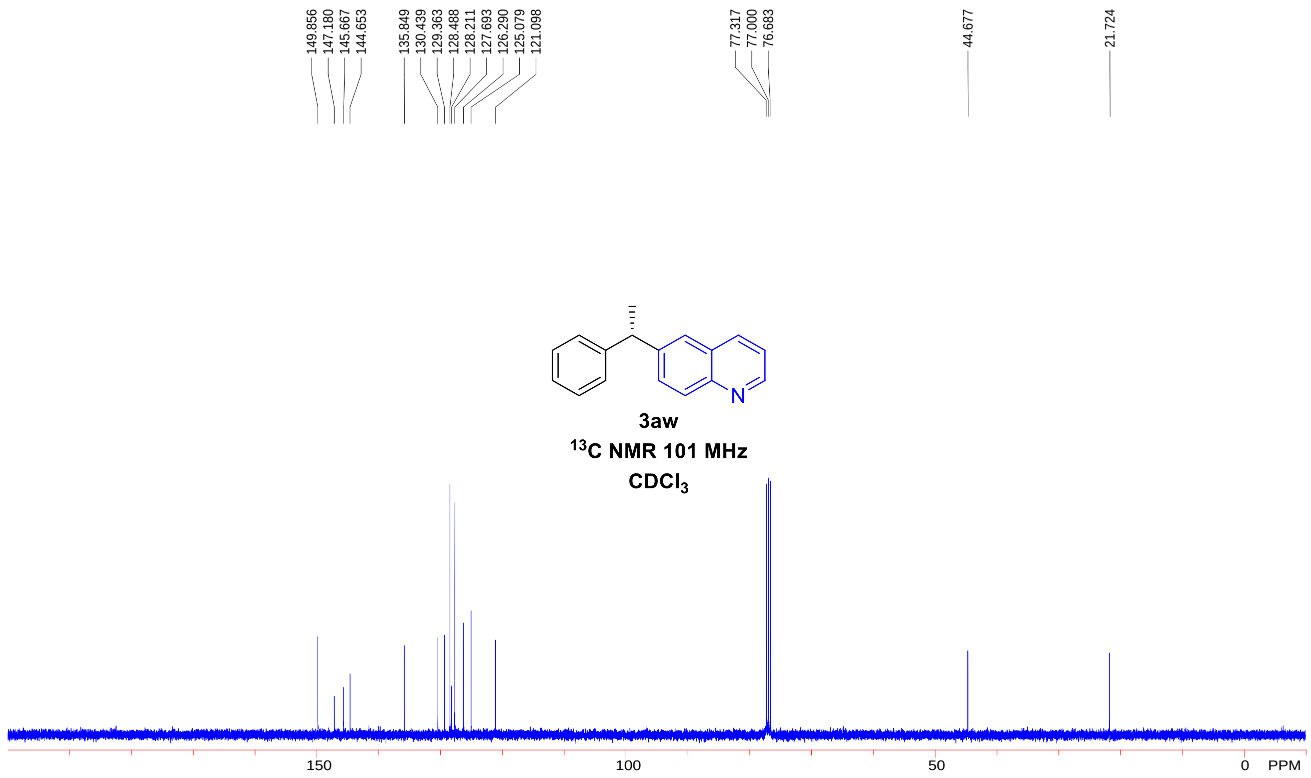

Supplementary Figure 79. ^{13}C NMR spectrum for **3aw**

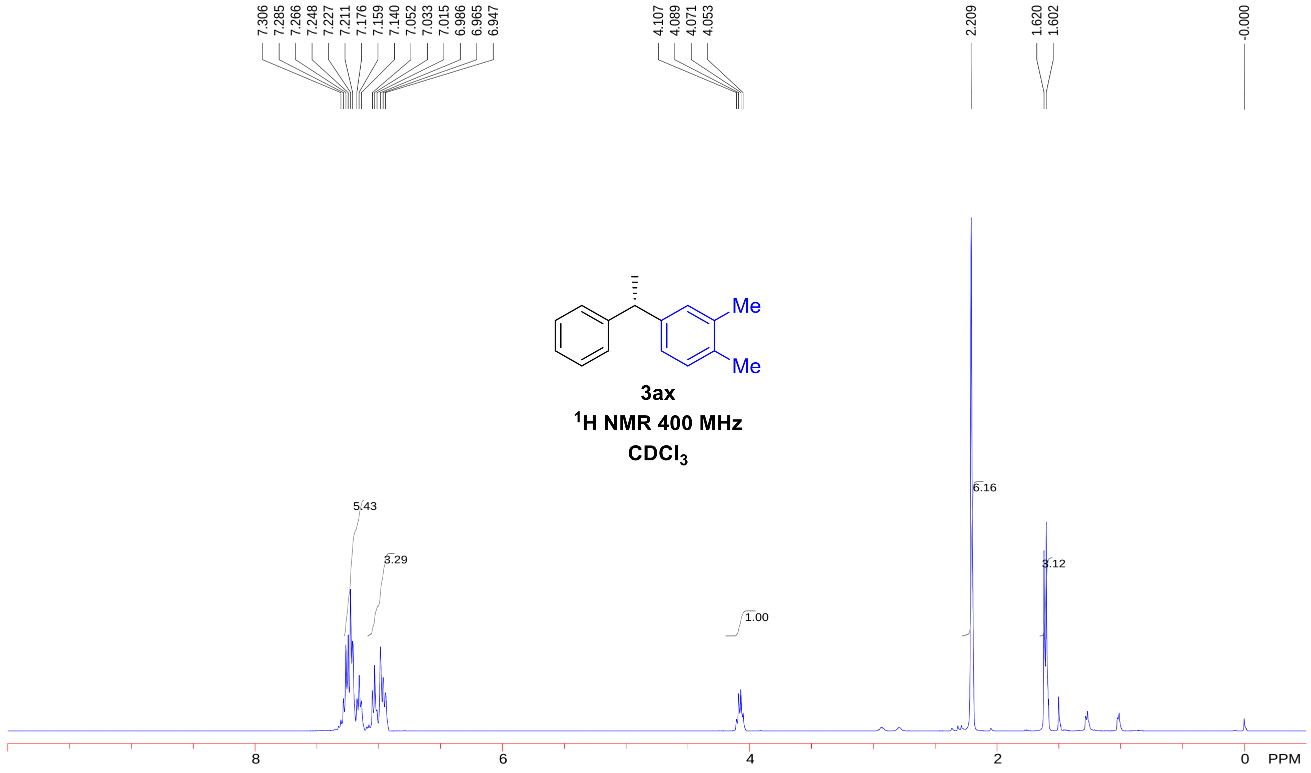

Supplementary Figure 80. ^{1}H NMR spectrum for **3ax**

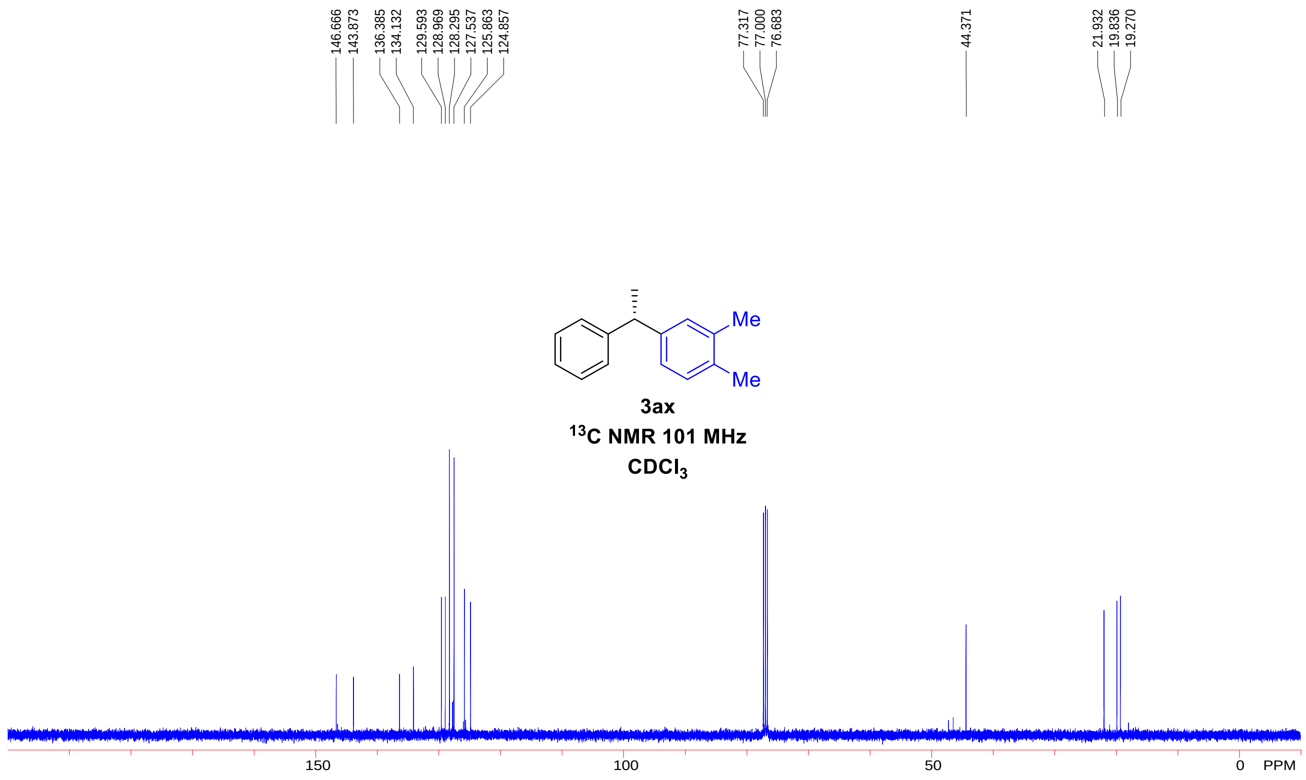

Supplementary Figure 81. ^{13}C NMR spectrum for **3ax**

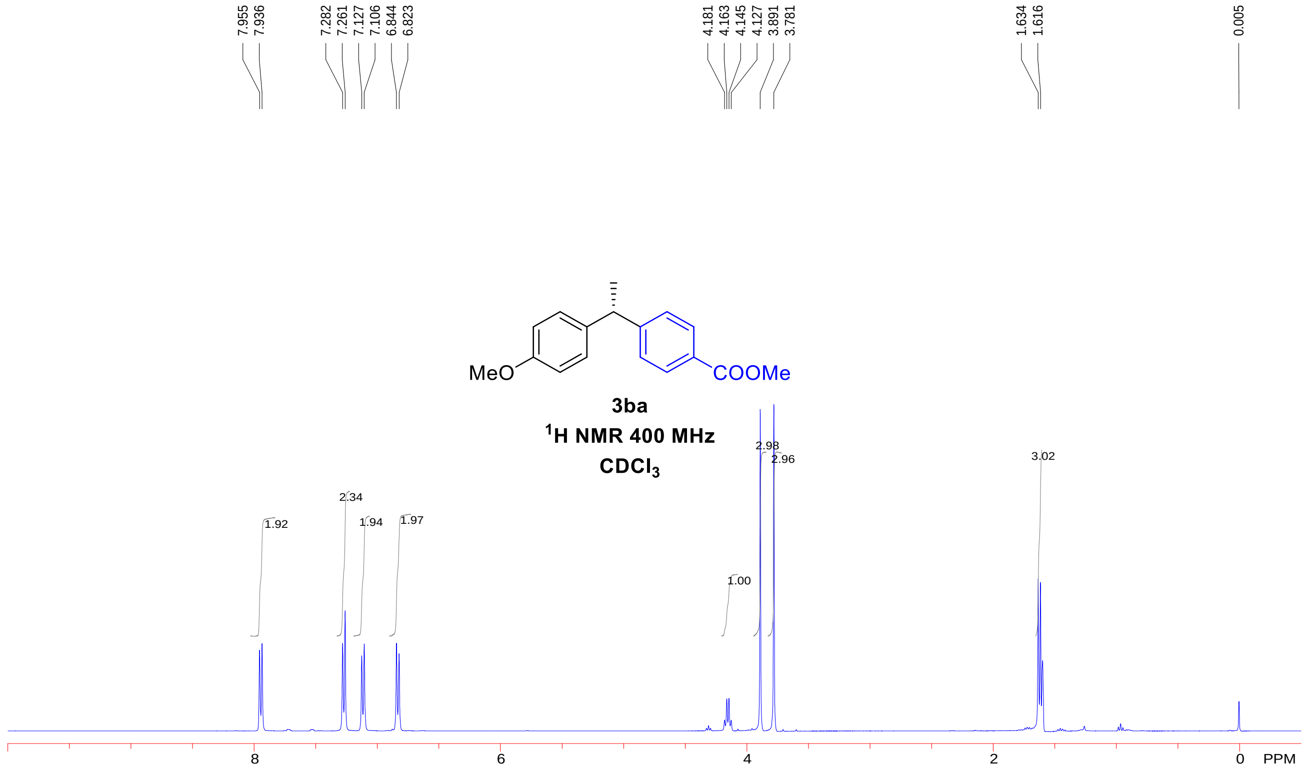

Supplementary Figure 82. ^{1}H NMR spectrum for **3ba**

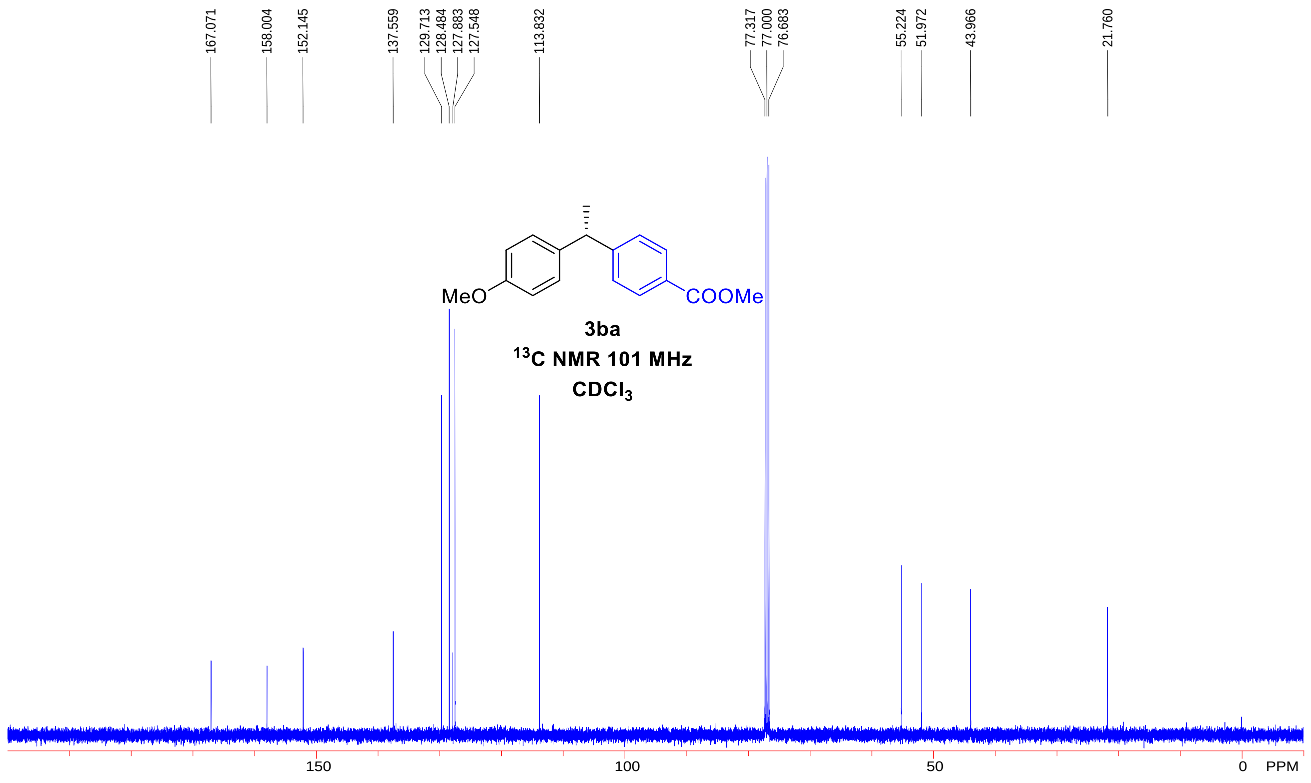

Supplementary Figure 83. ^{13}C NMR spectrum for **3ba**

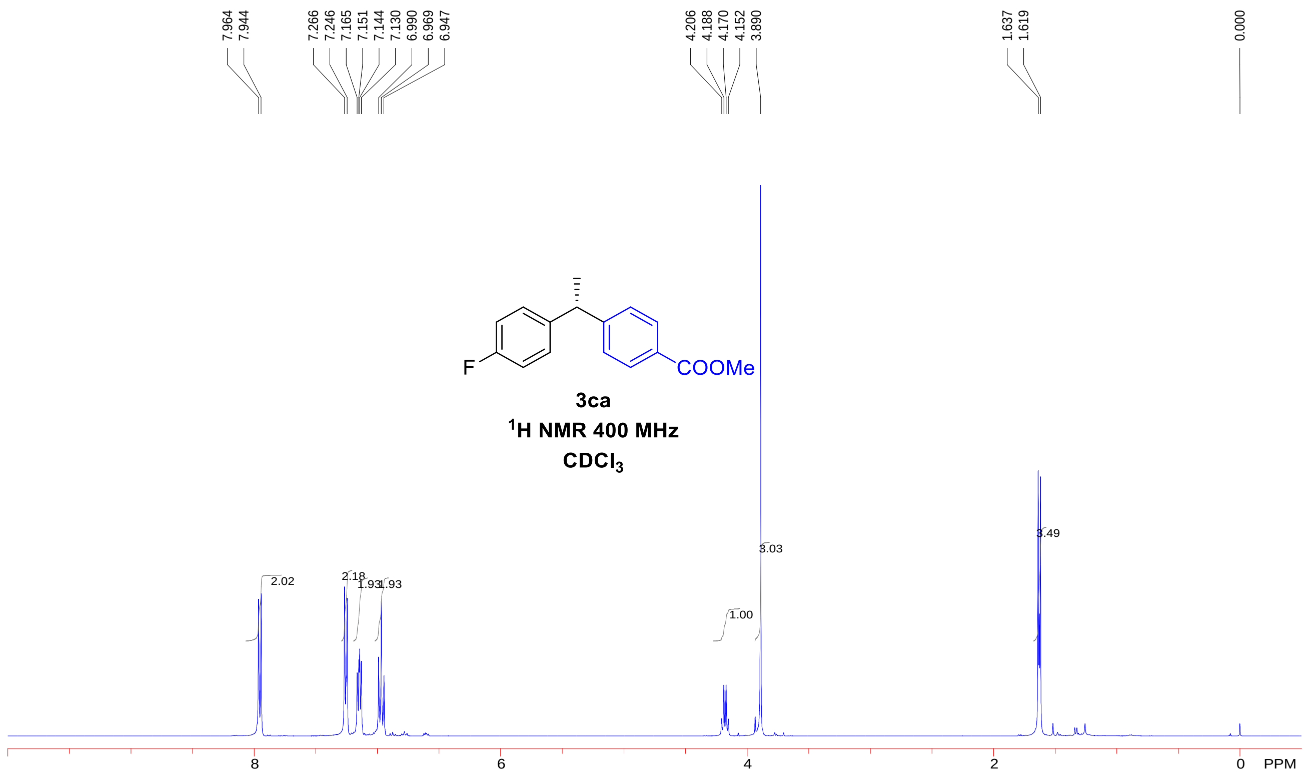

Supplementary Figure 84. ^{1}H NMR spectrum for **3ca**

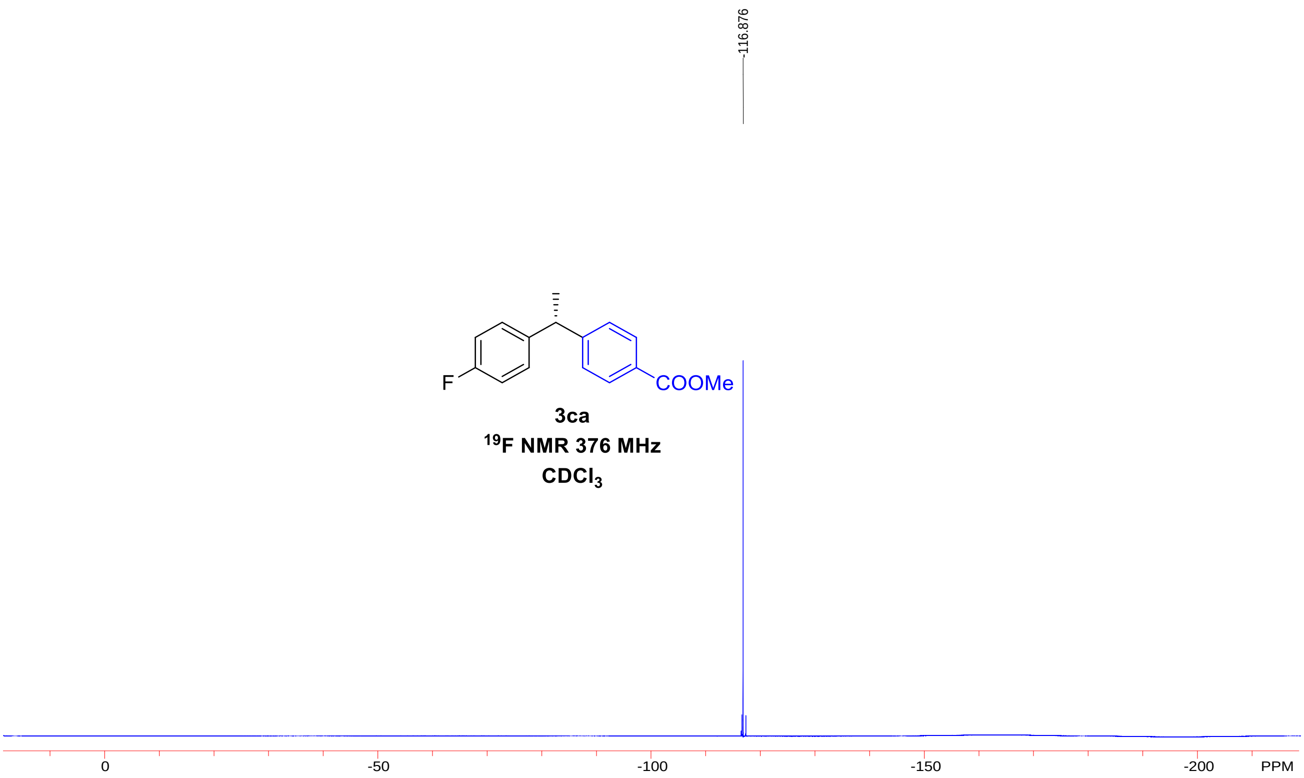

Supplementary Figure 85. ^{19}F NMR spectrum for **3ca**

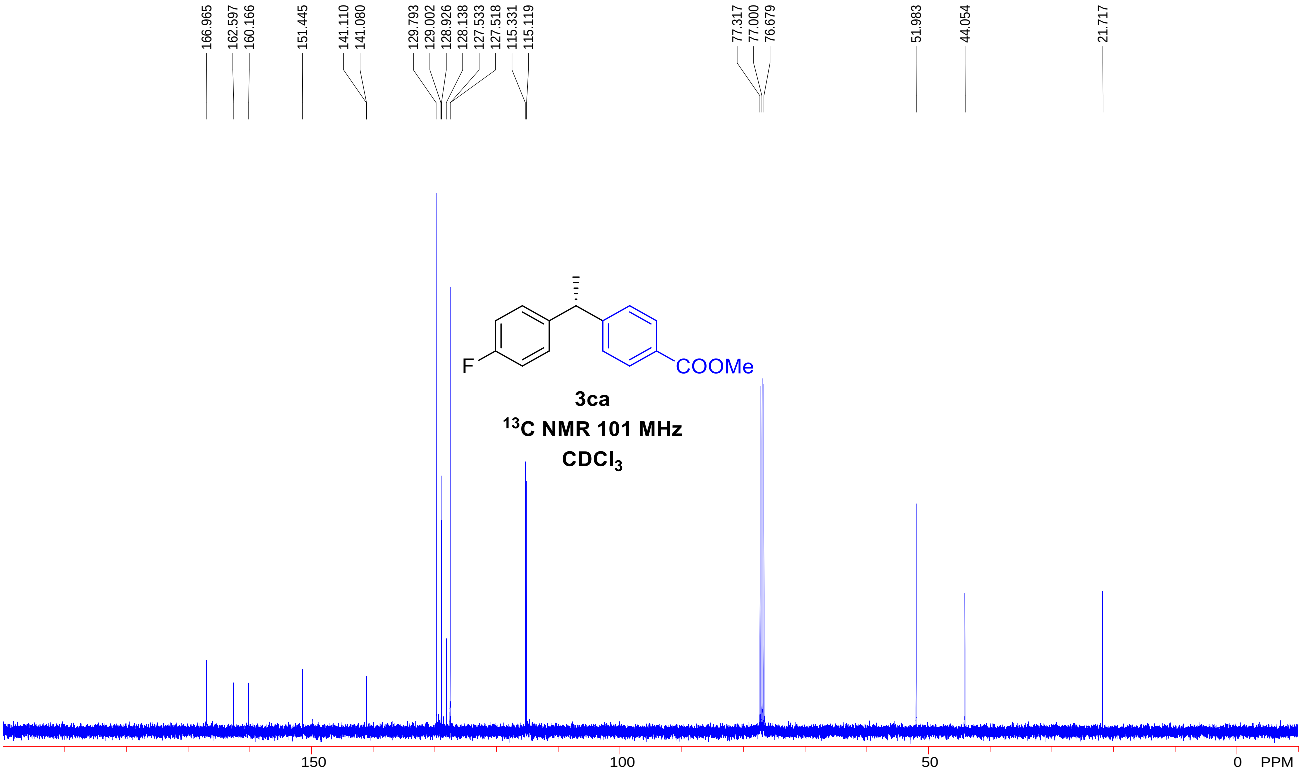

Supplementary Figure 86. ^{13}C NMR spectrum for **3ca**

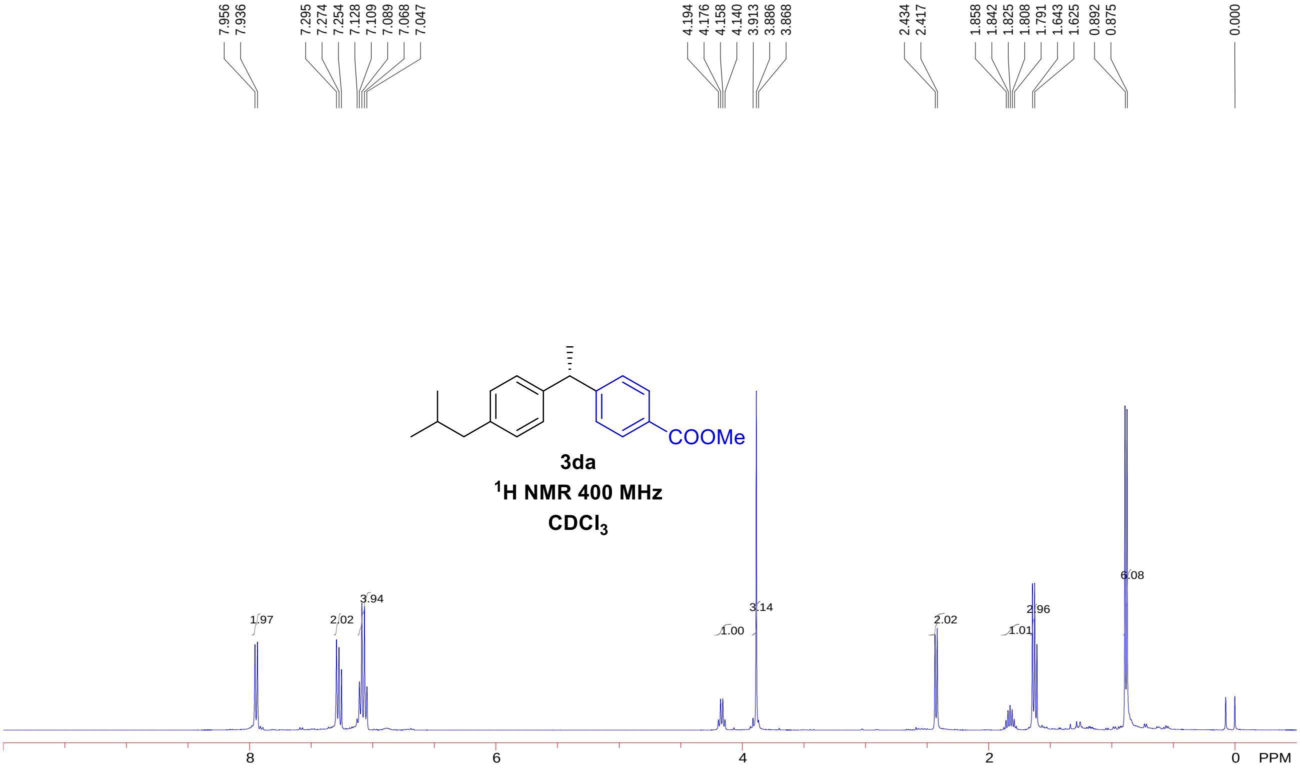

Supplementary Figure 87. ^{1}H NMR spectrum for **3da**

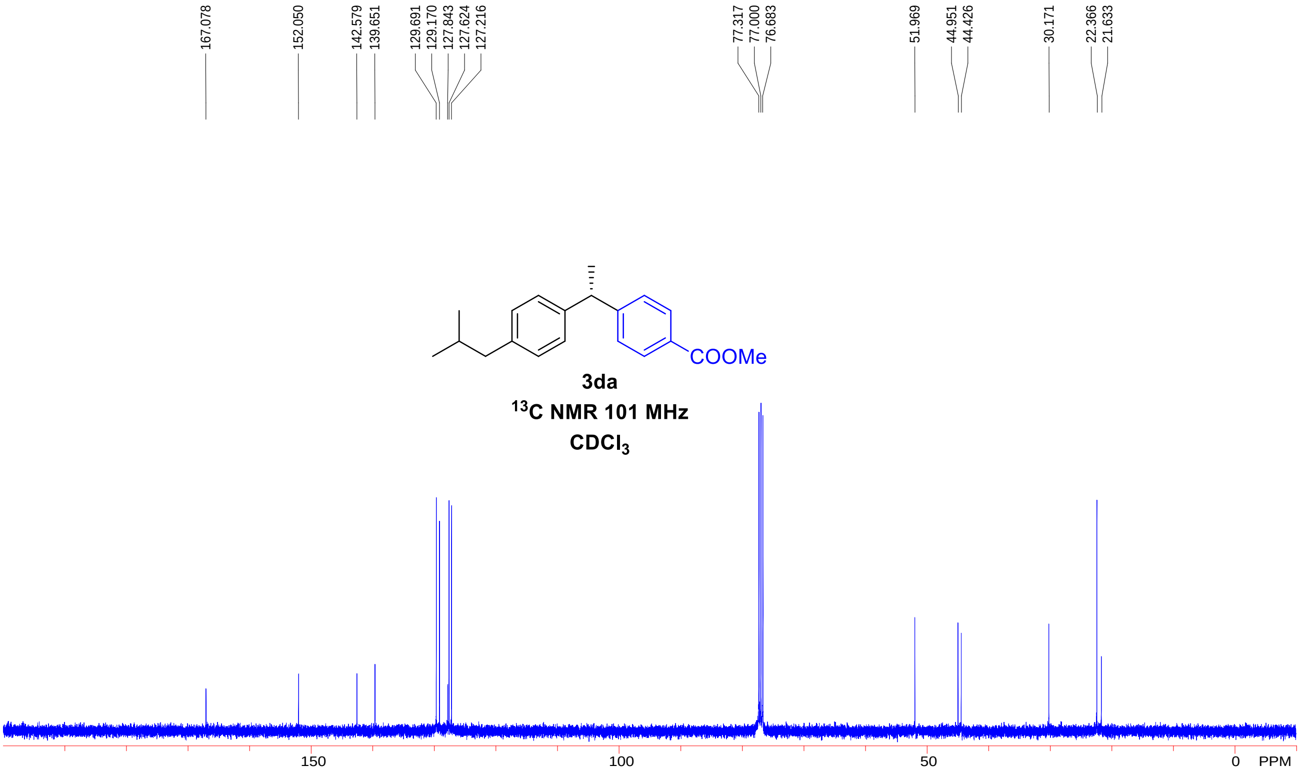

Supplementary Figure 88. ^{13}C NMR spectrum for **3da**

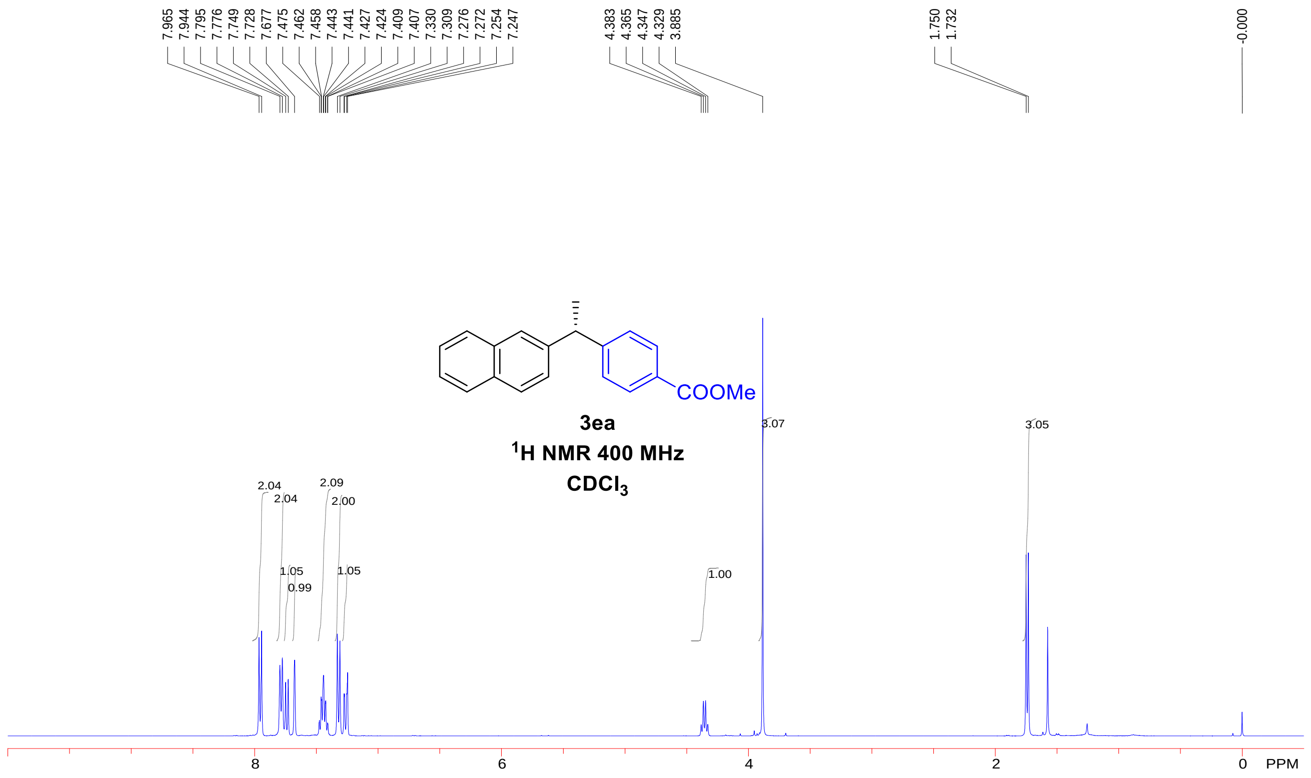

Supplementary Figure 89. ^{1}H NMR spectrum for **3ea**

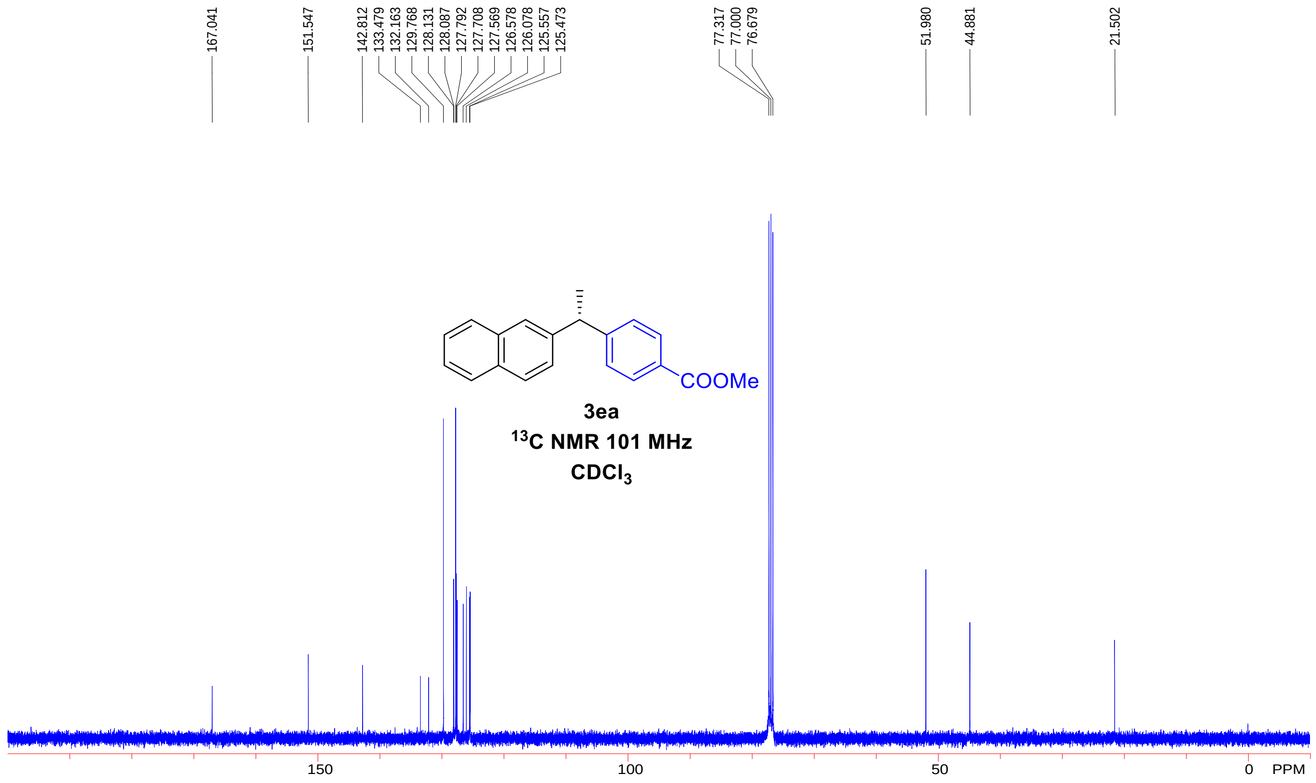

Supplementary Figure 90. ^{13}C NMR spectrum for **3ea**

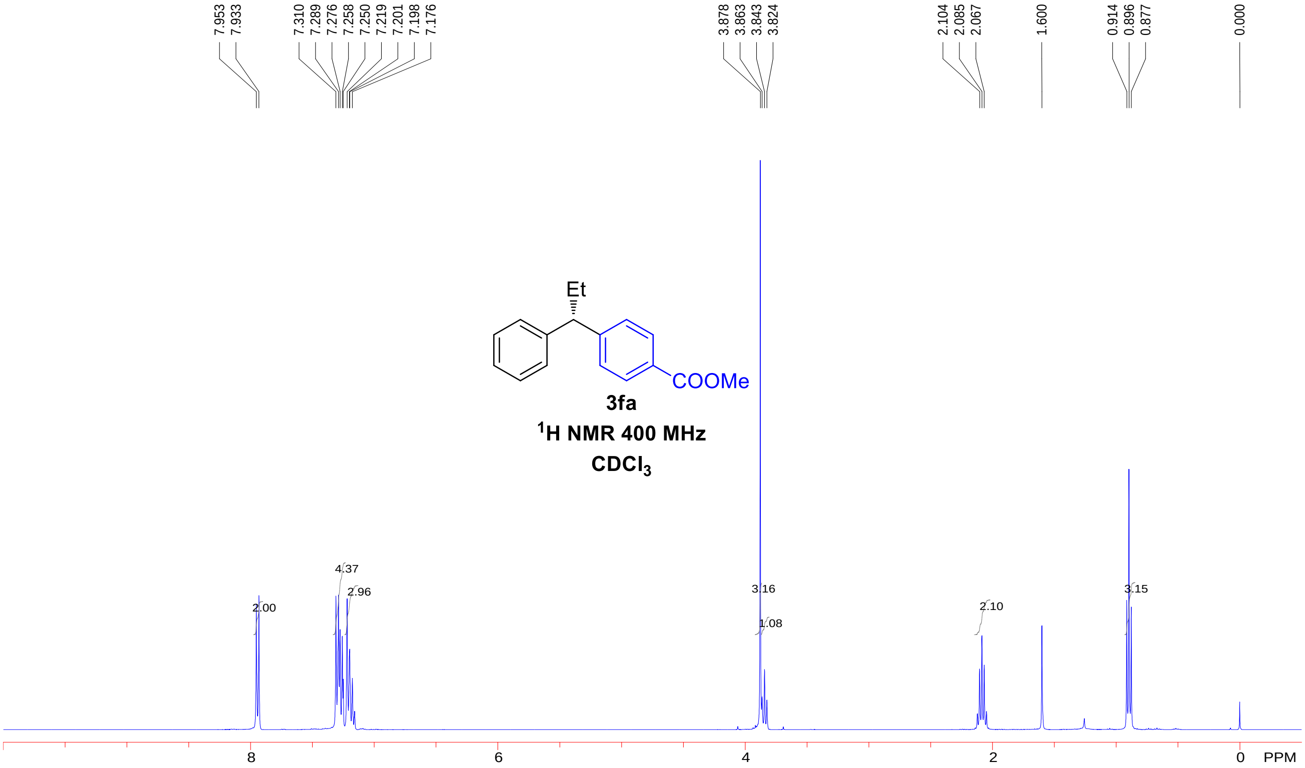

Supplementary Figure 91. ^{1}H NMR spectrum for **3f**

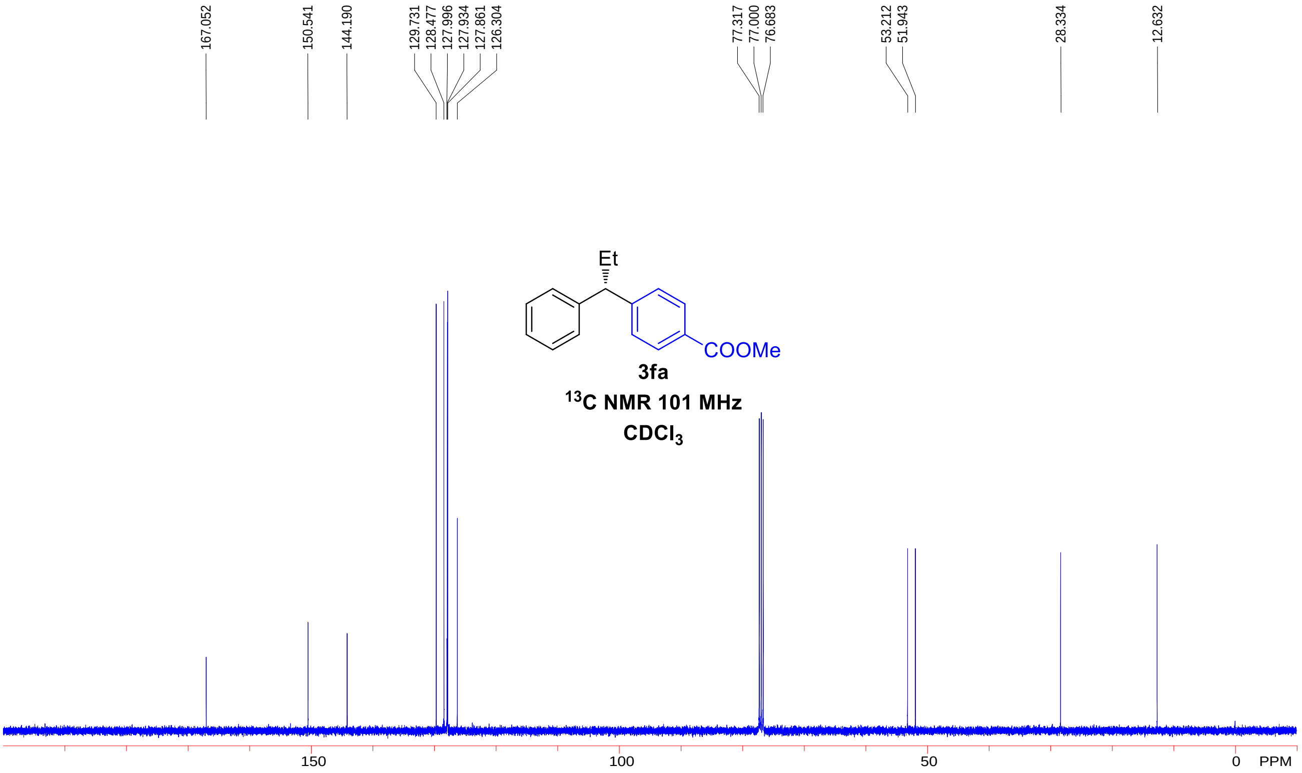

Supplementary Figure 92. ^{13}C NMR spectrum for **3fa**

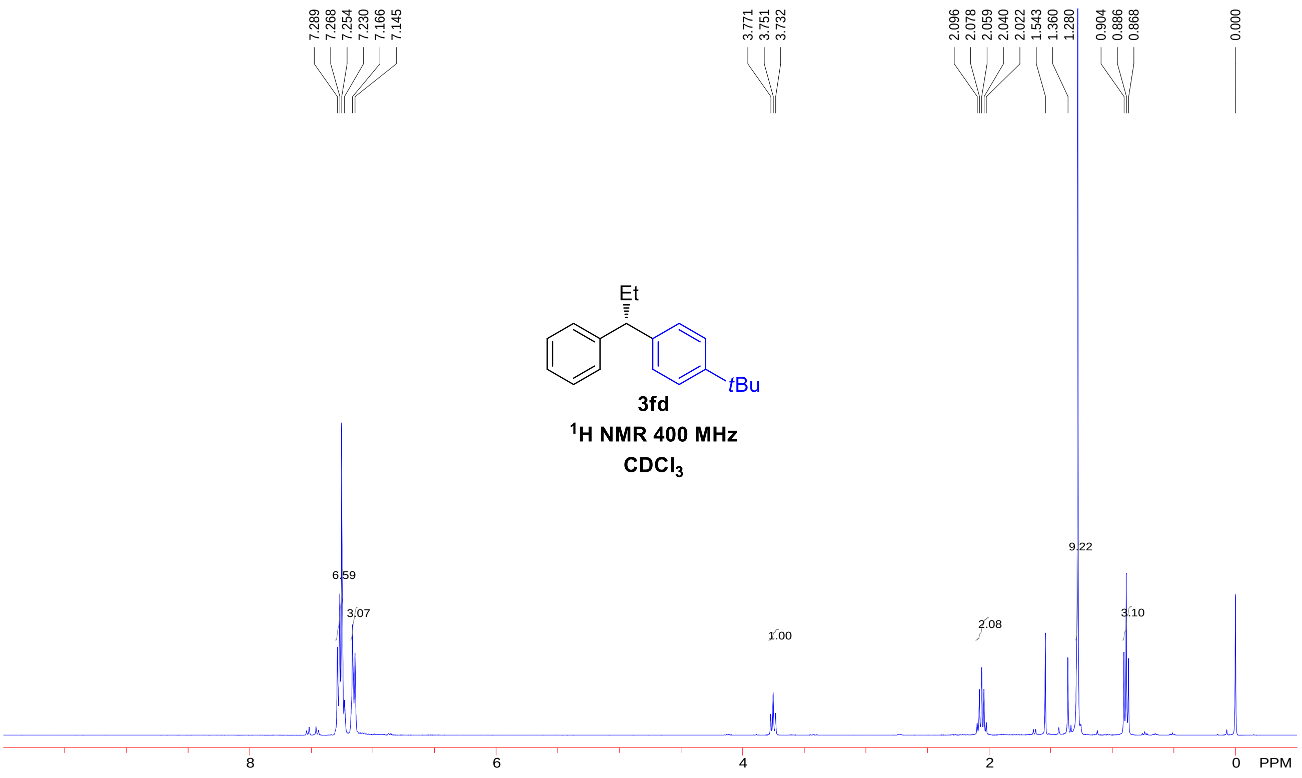

Supplementary Figure 93. ^{1}H NMR spectrum for **3fd**

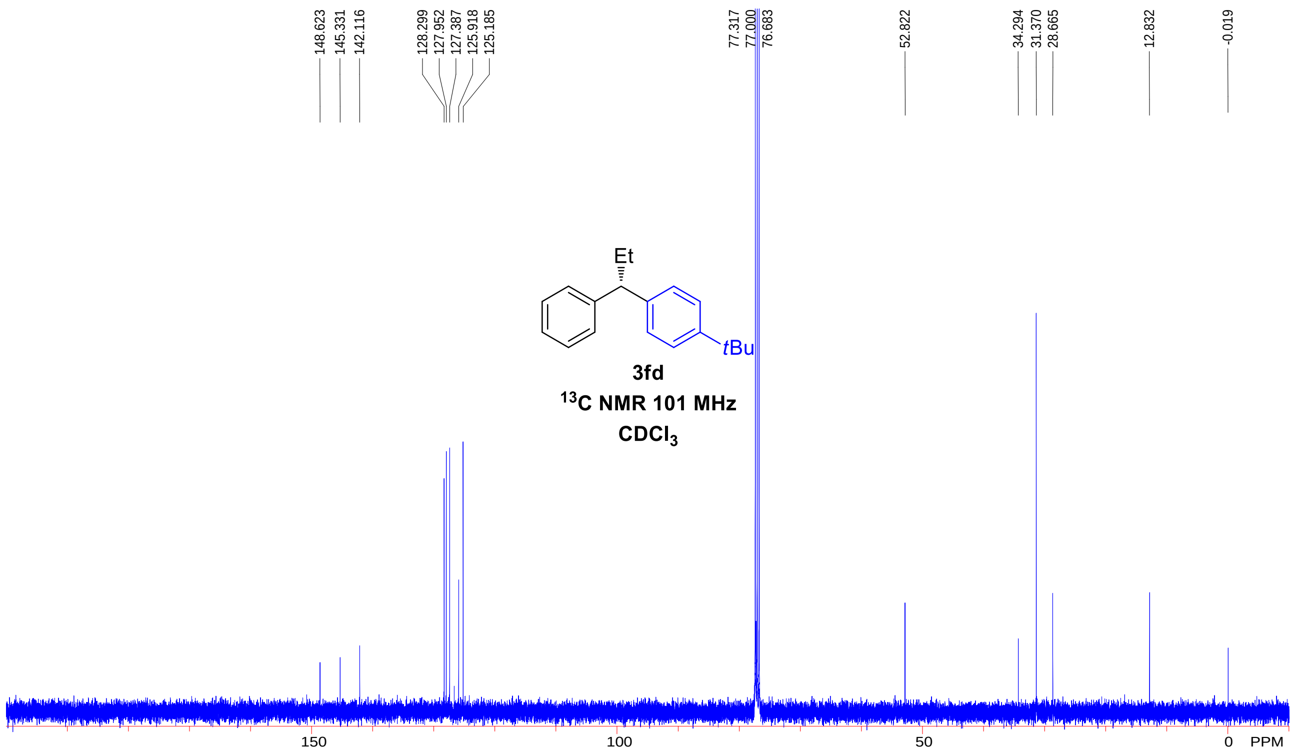

Supplementary Figure 94. ^{13}C NMR spectrum for **3fd**

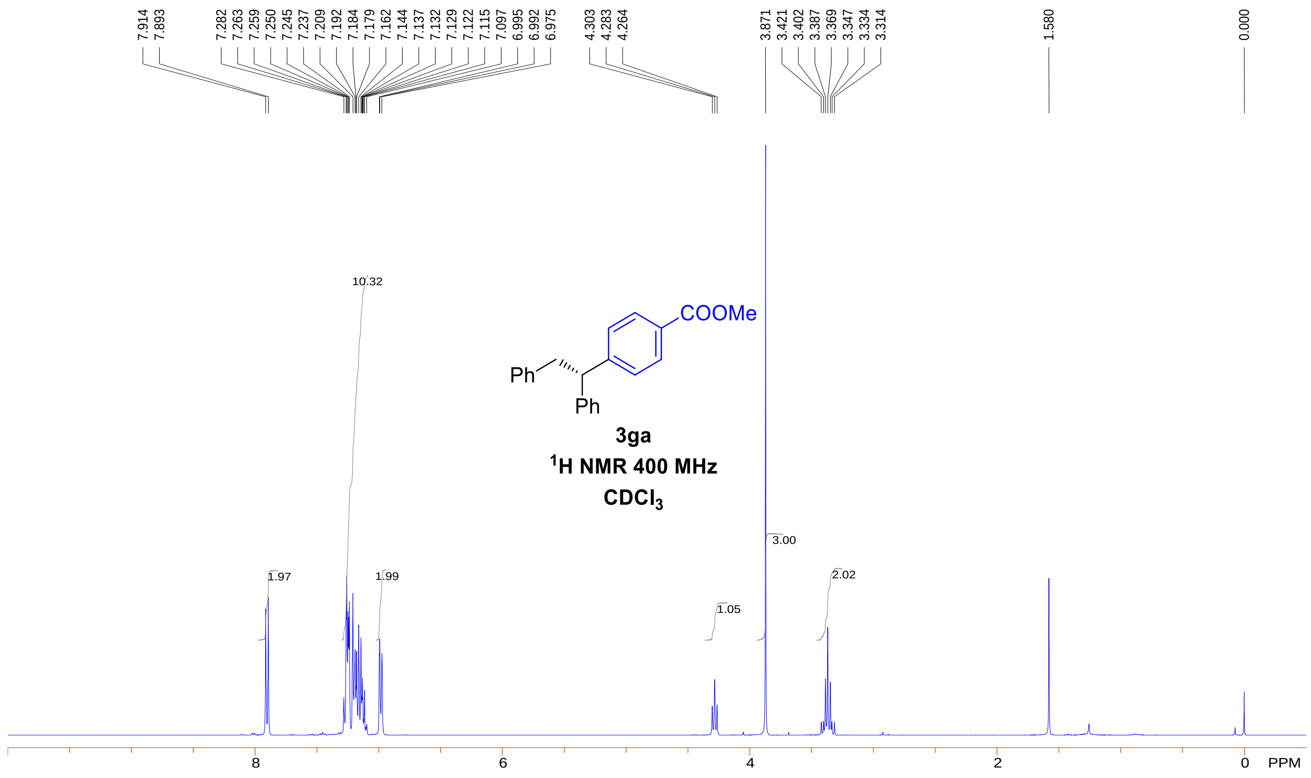

Supplementary Figure 95. ^{1}H NMR spectrum for **3ga**

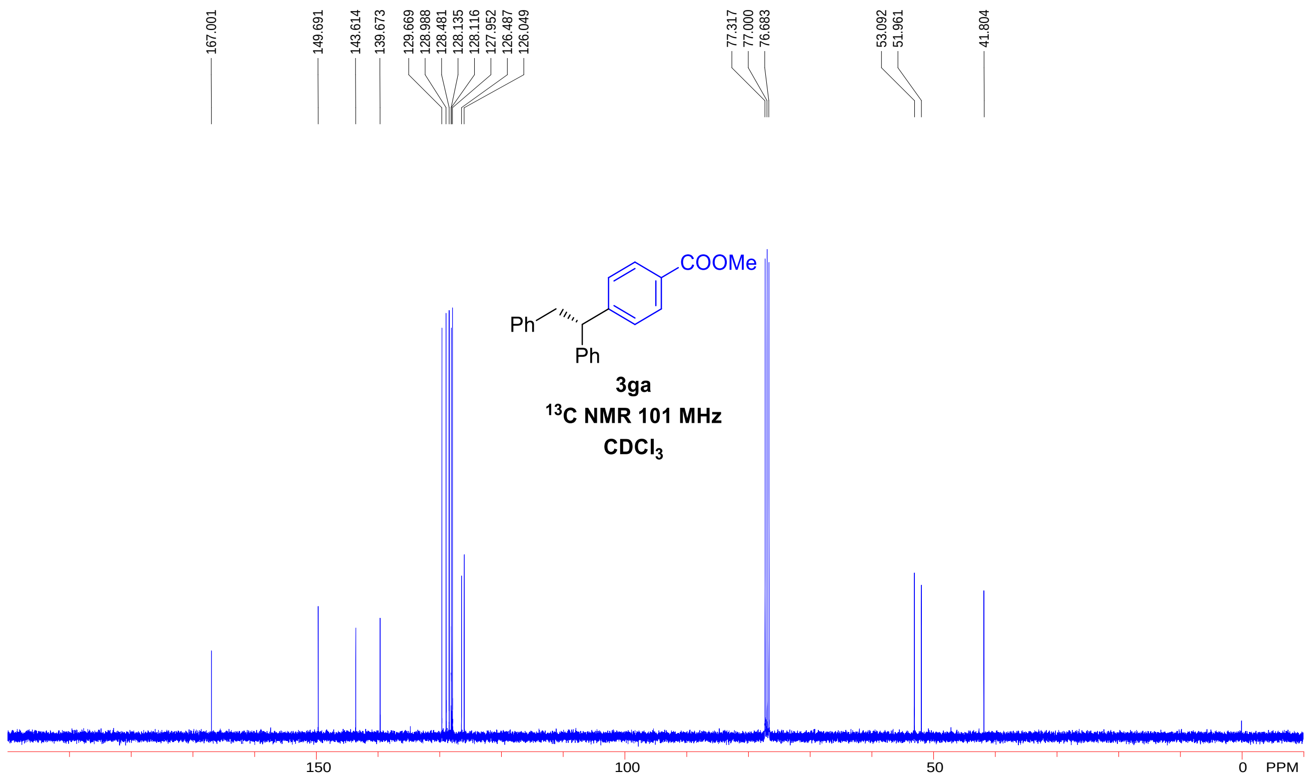

Supplementary Figure 96. ^{13}C NMR spectrum for **3ga**

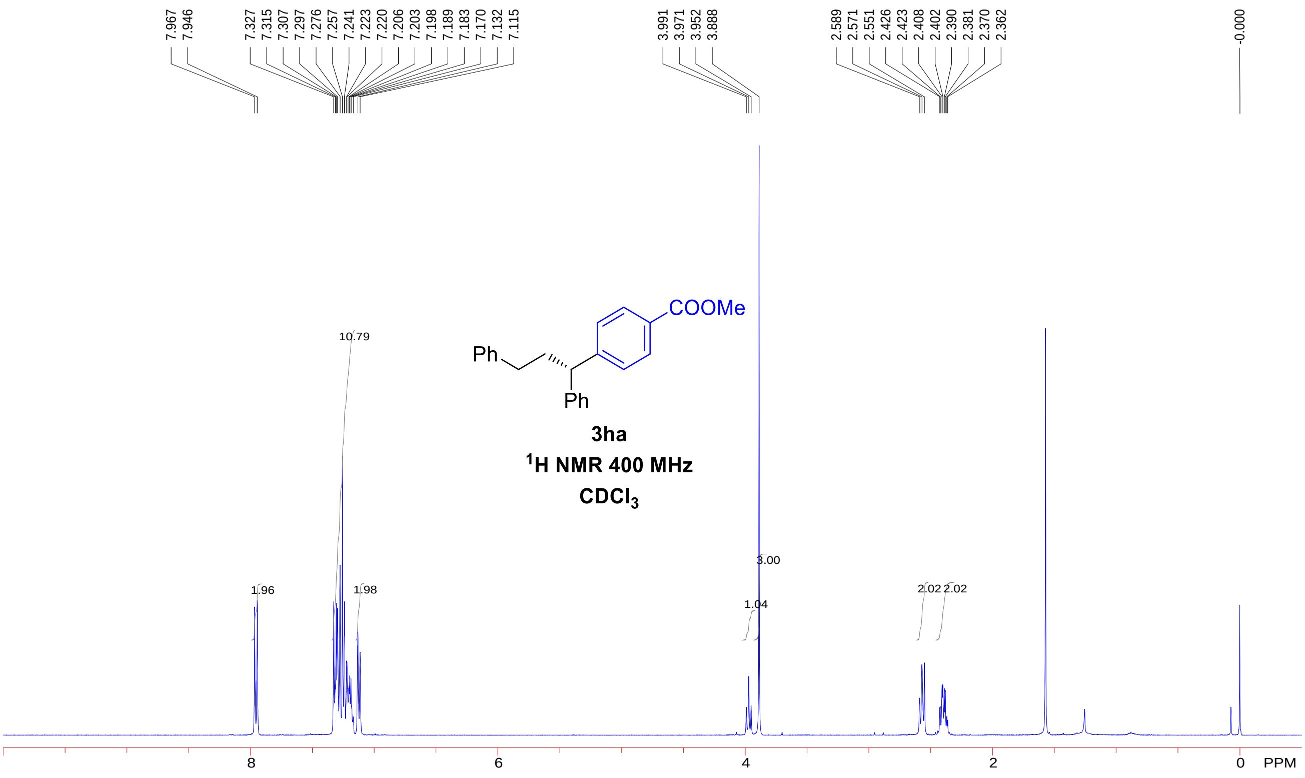

Supplementary Figure 97. ^{1}H NMR spectrum for **3ha**

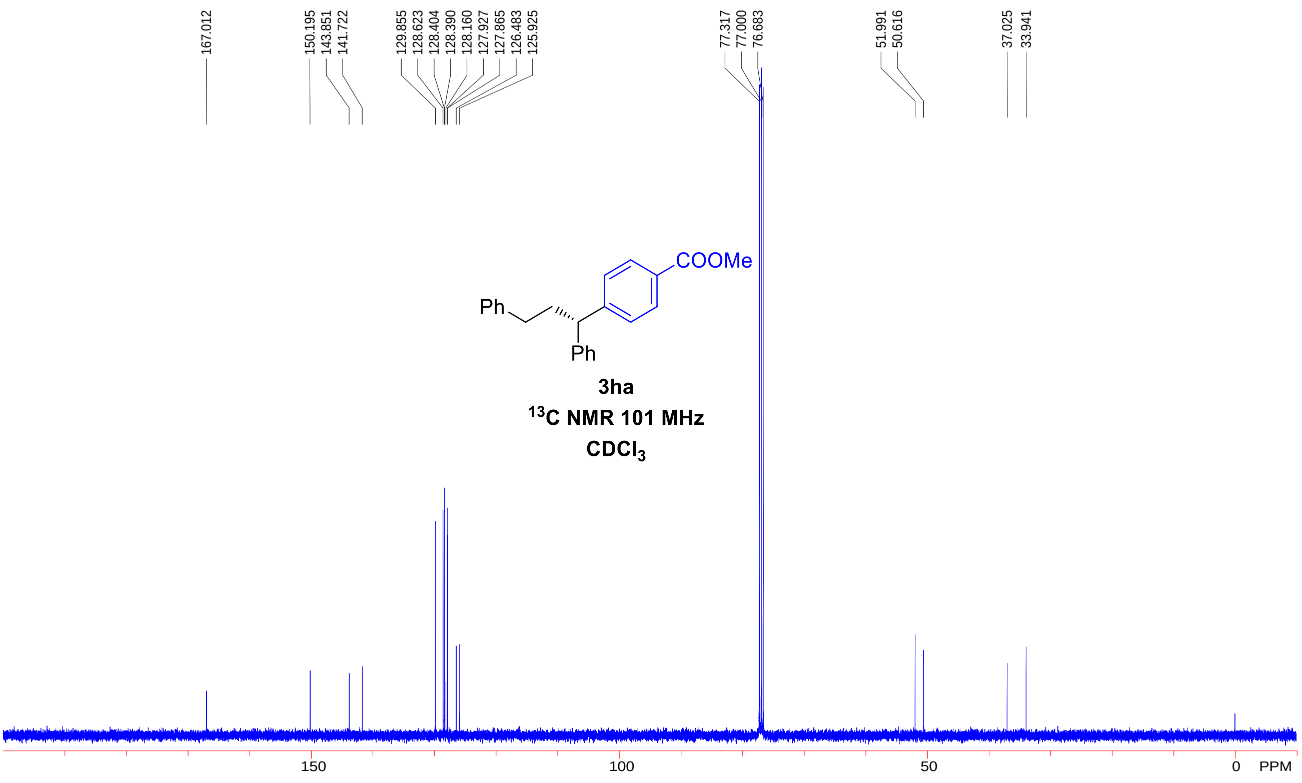

Supplementary Figure 98. ^{13}C NMR spectrum for **3ha**

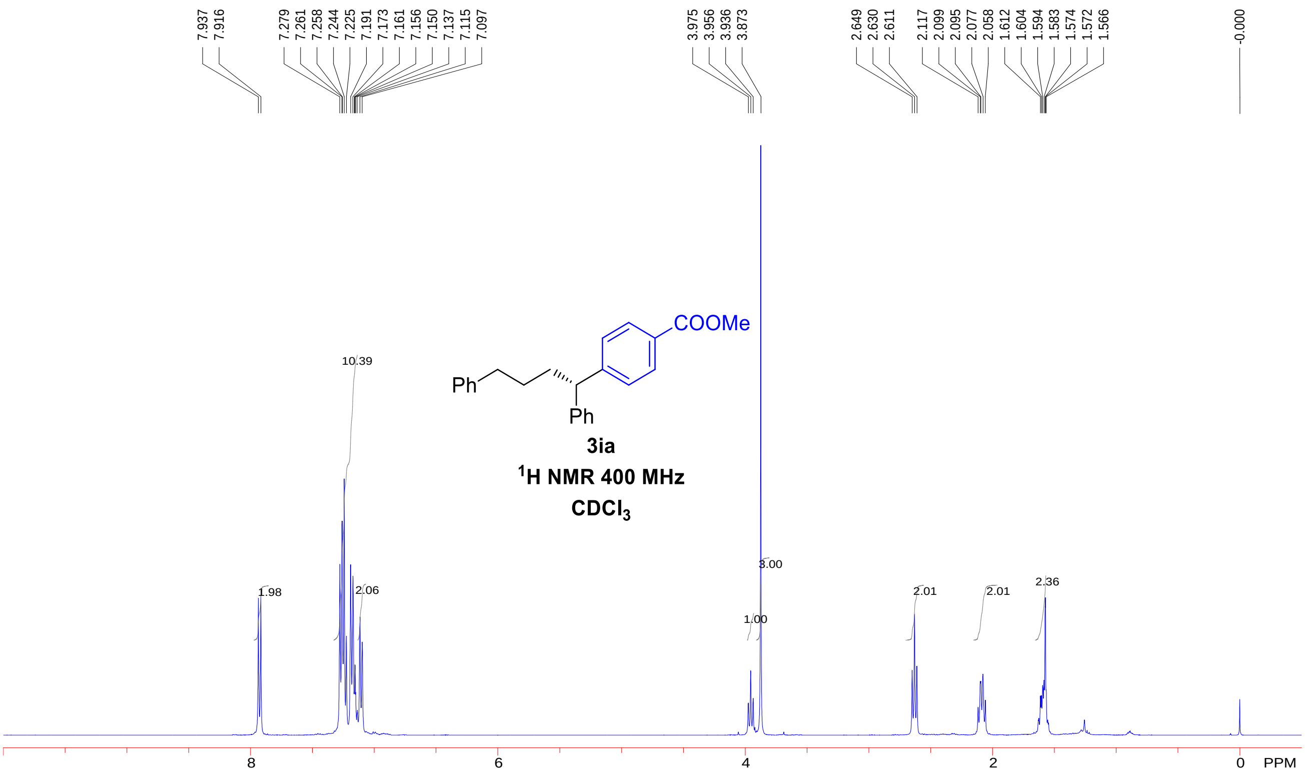

Supplementary Figure 99. ^{1}H NMR spectrum for **3ia**

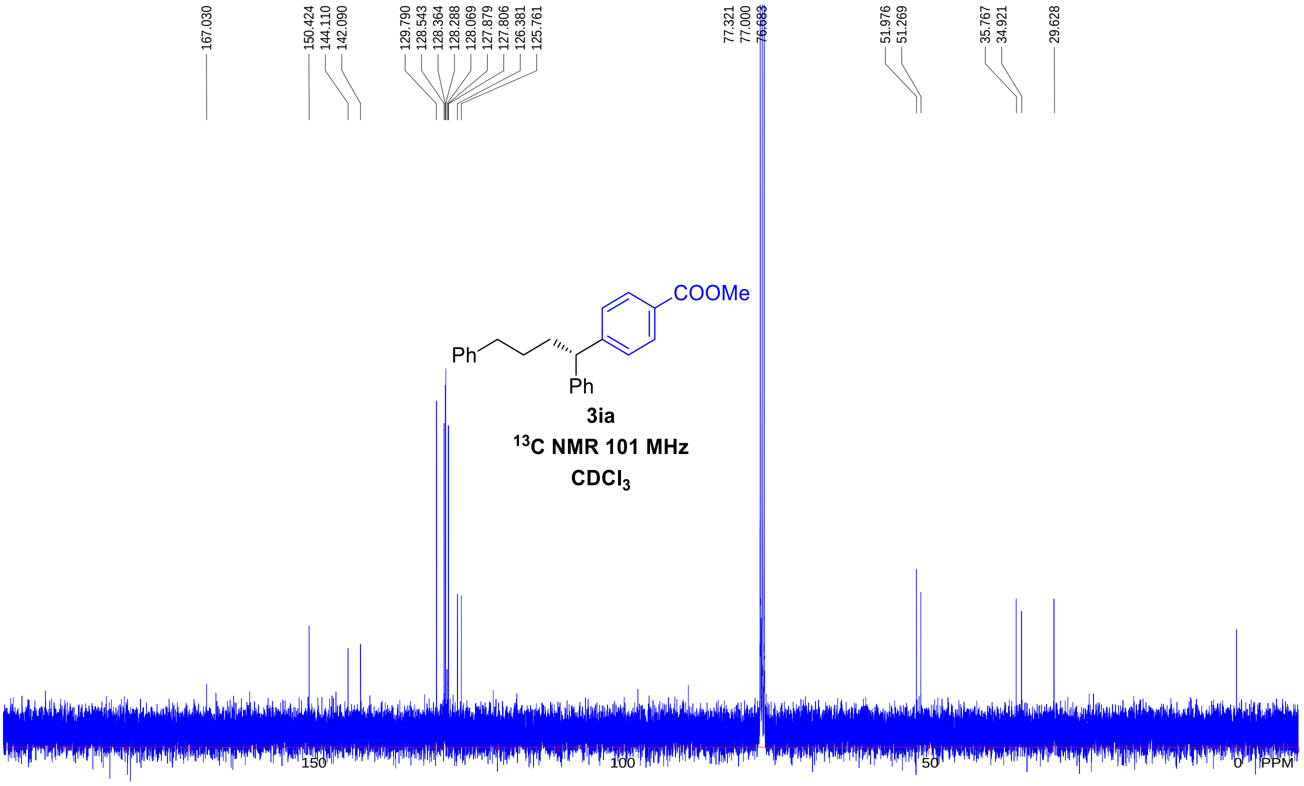

Supplementary Figure 100. ^{13}C NMR spectrum for **3ia**

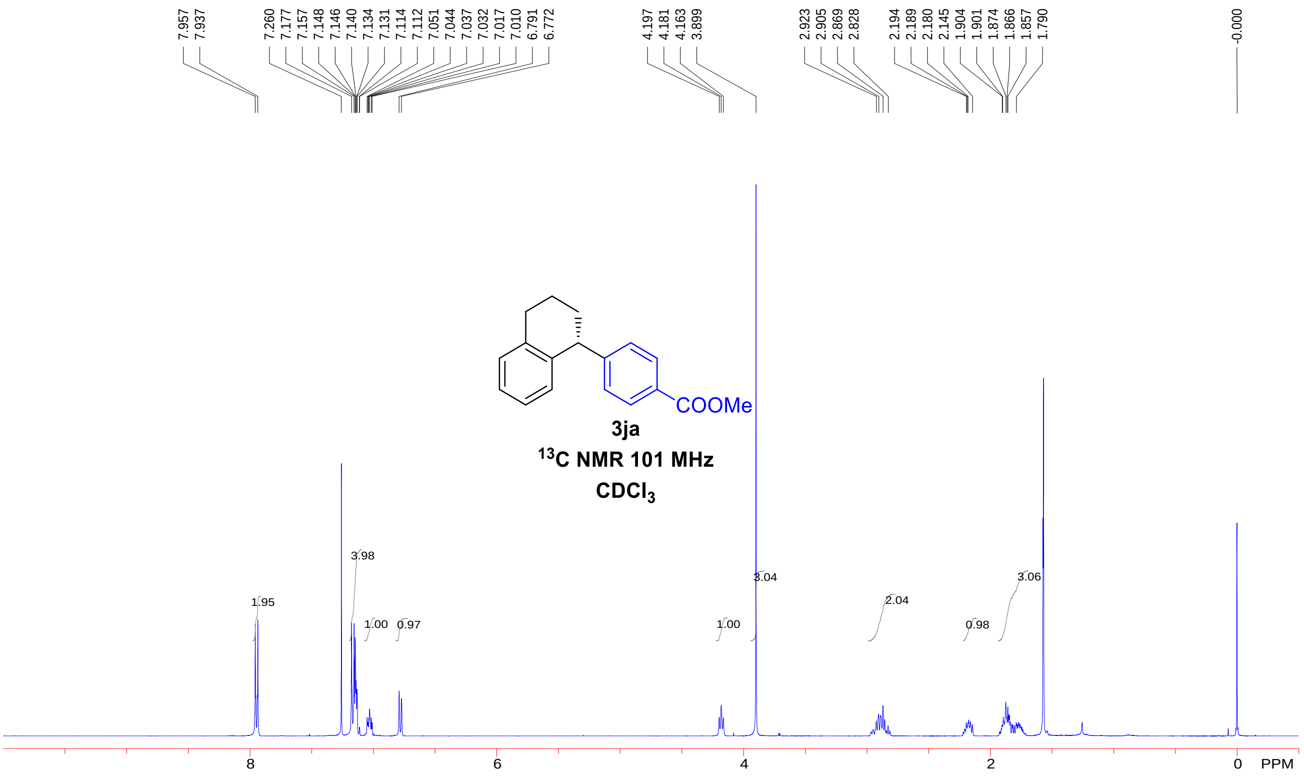

Supplementary Figure 101. ^{1}H NMR spectrum for **3ja**

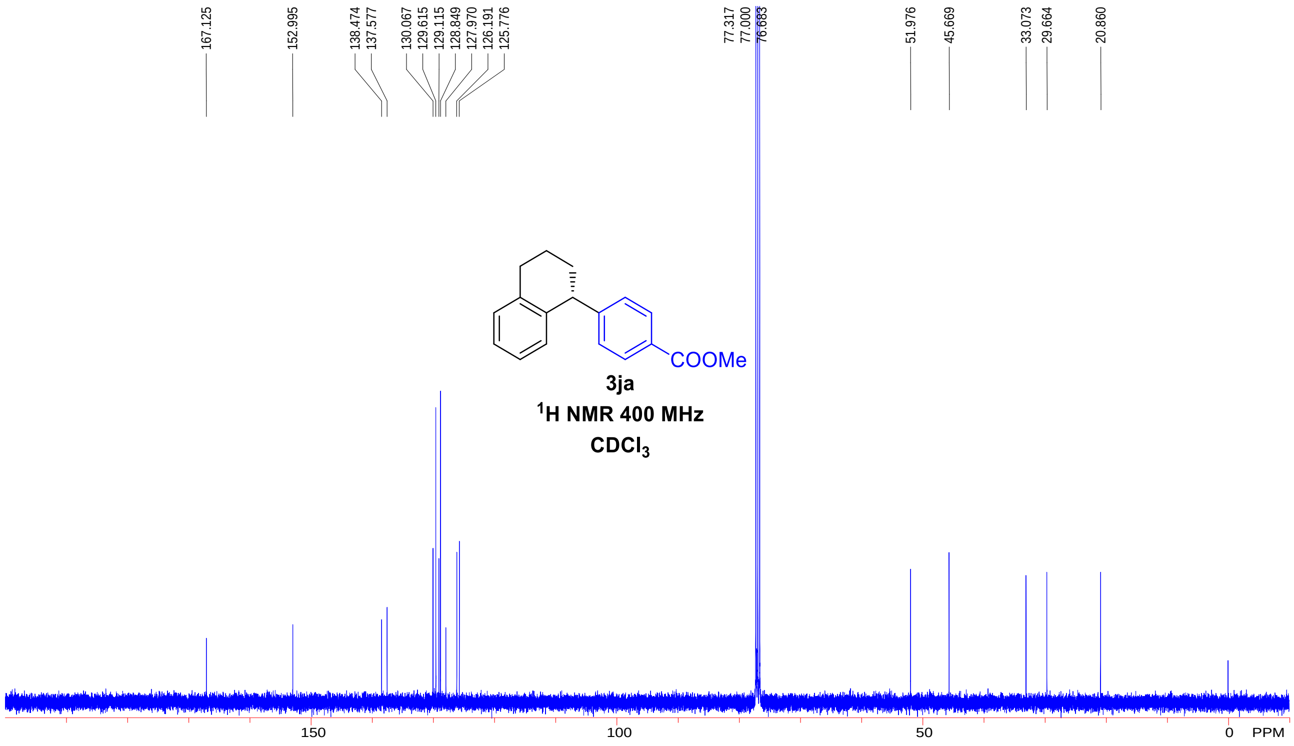

Supplementary Figure 102. ^{13}C NMR spectrum for **3ja**

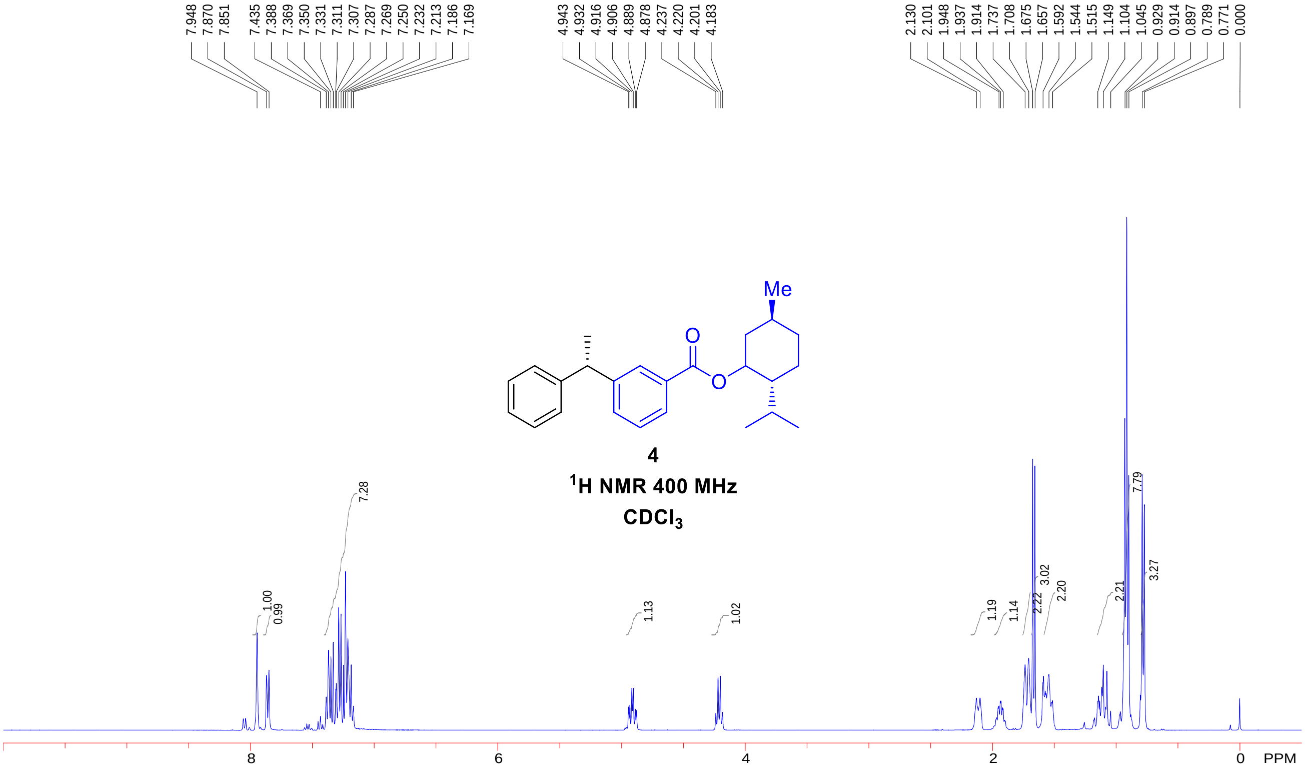

Supplementary Figure 103. ^{1}H NMR spectrum for **4**

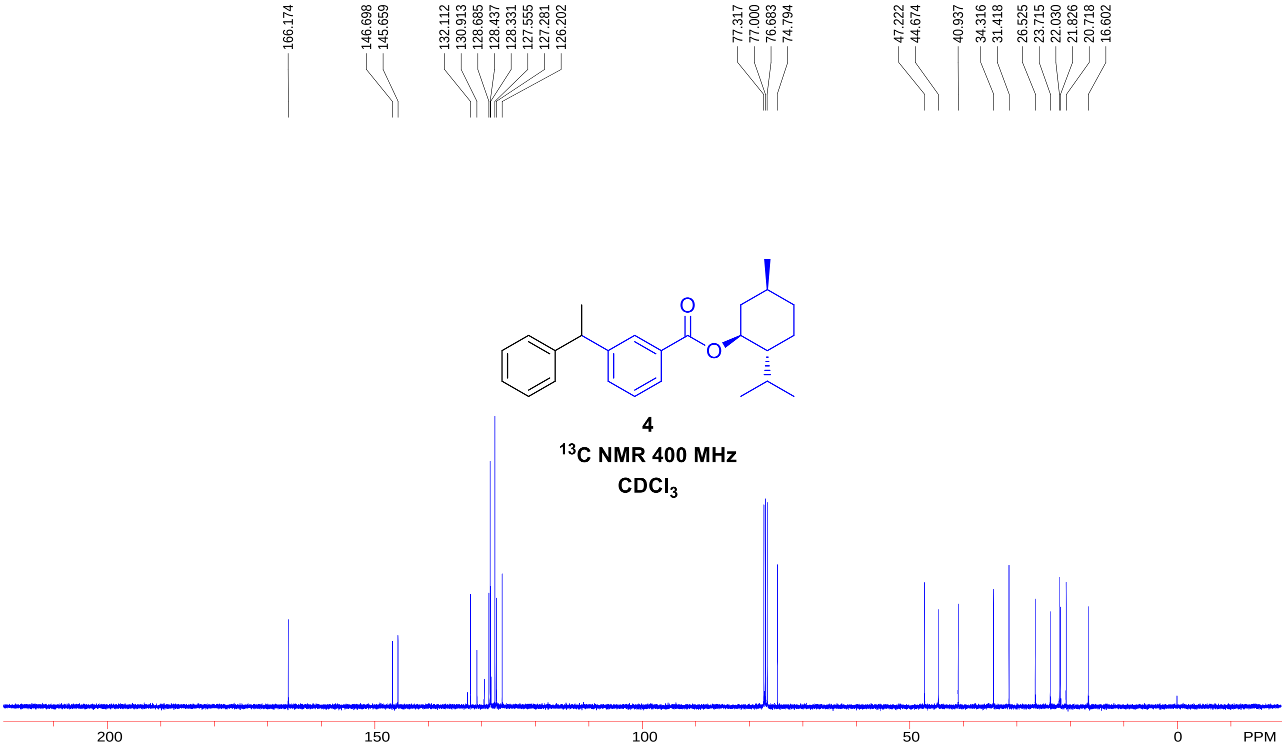

Supplementary Figure 104. ^{13}C NMR spectrum for **4**

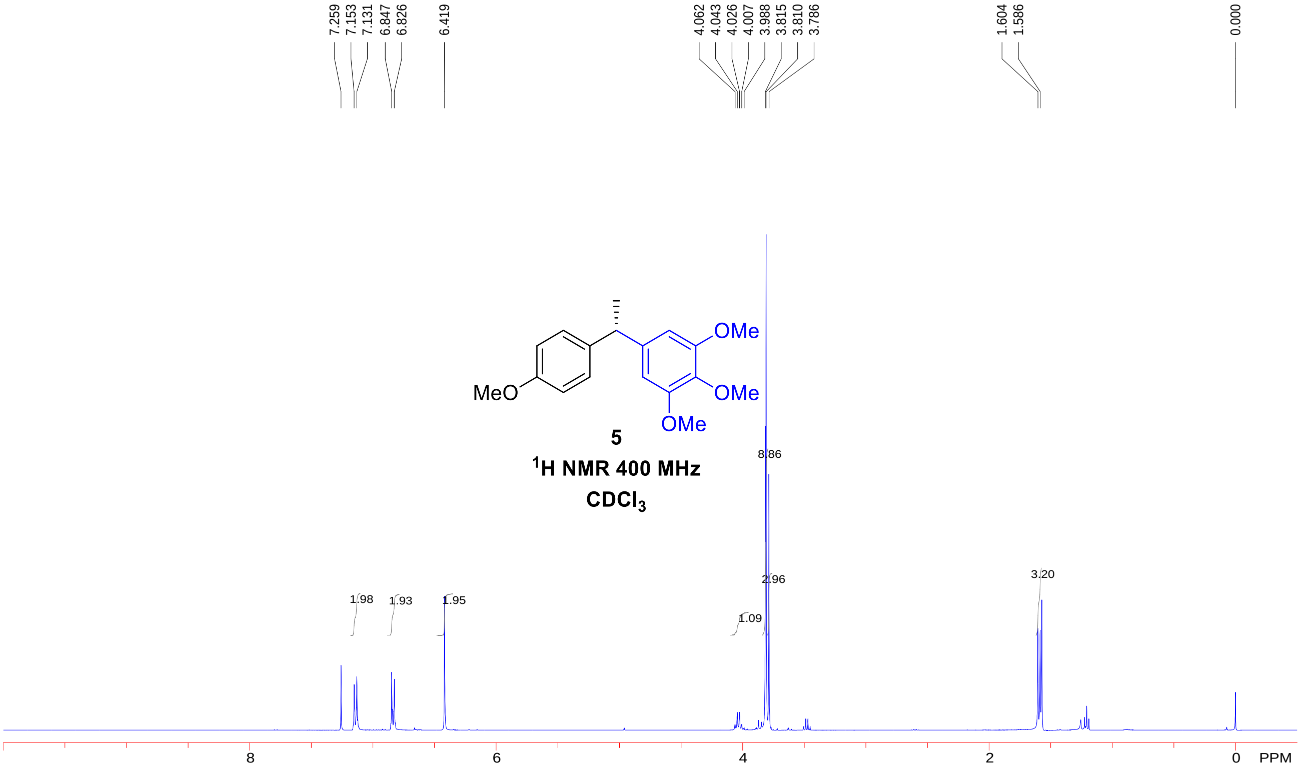

Supplementary Figure 105. ^{1}H NMR spectrum for **5**

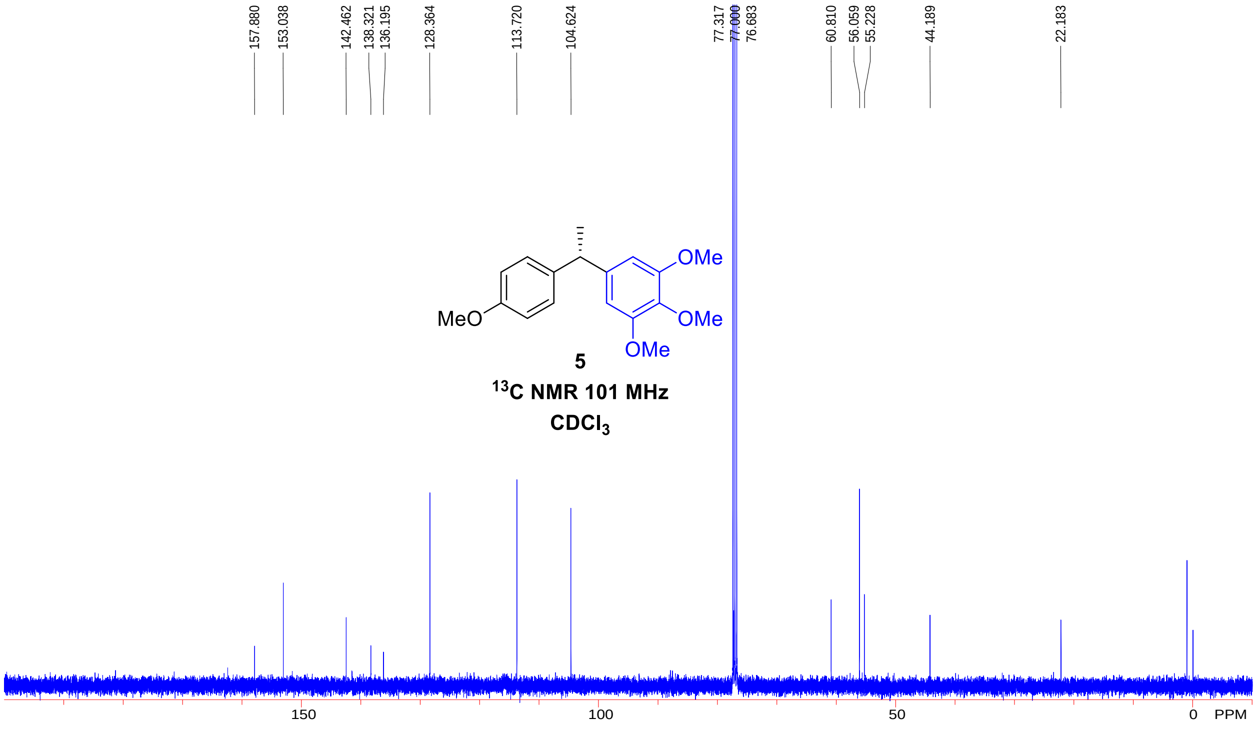

Supplementary Figure 106. ^{13}C NMR spectrum for **5**

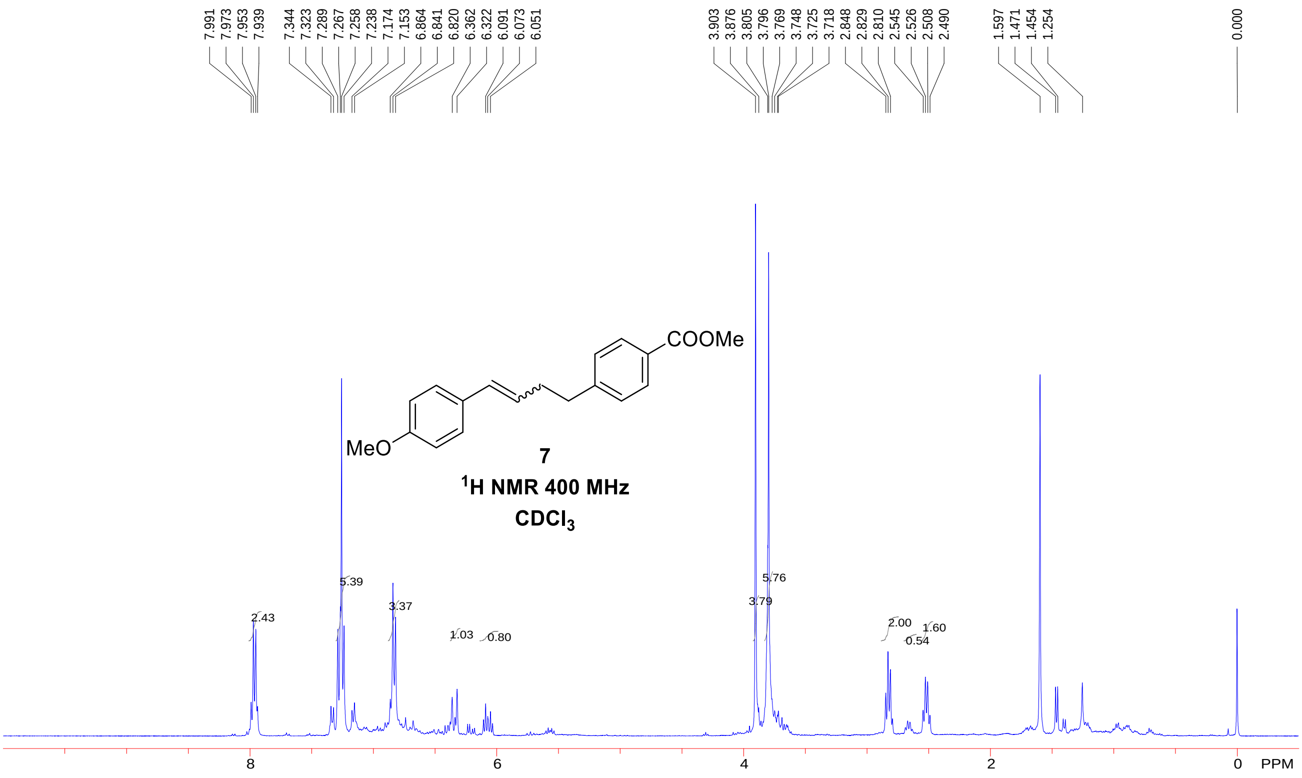

Supplementary Figure 107. ^{1}H NMR spectrum for **7**

All the Chinese characters in HPLC spectra are replaced by English translations

HPLC Condition： OD-H*2, n-hexane/iPrOH = 99.8/0.2, 1.0 ml/min, 220 nm

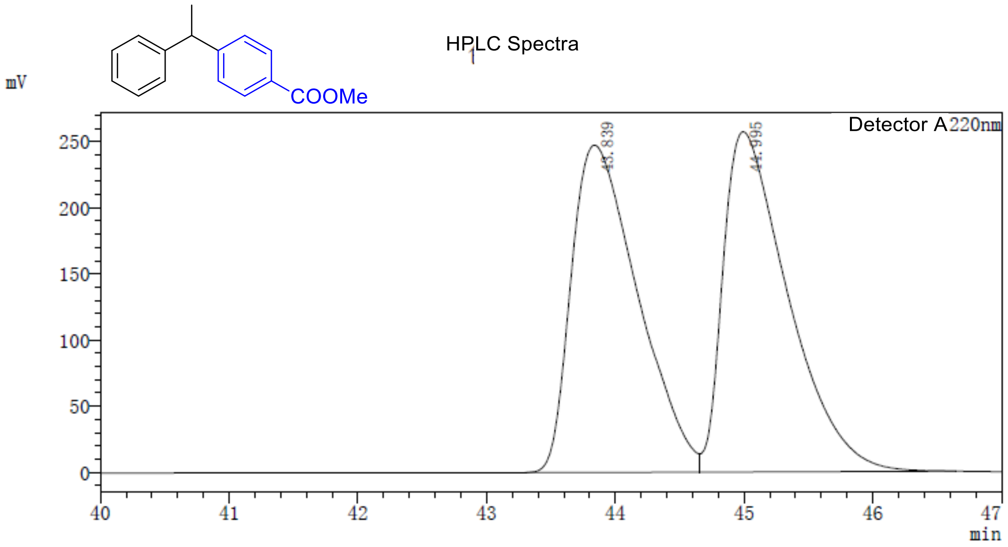

Area Percent Report

Detector A 220nm

Number	Remaining Time	Area	Height	Note	Area %
1	43.839	8895809	247509		49.610
2	44.995	9035627	257217	V M	50.390
Total		17931436	504726		100.000

HPLC Condition： 2*OD-H , n-hexane/iPrOH = 99.8/0.2, 1.0 mL/min, 220 nm

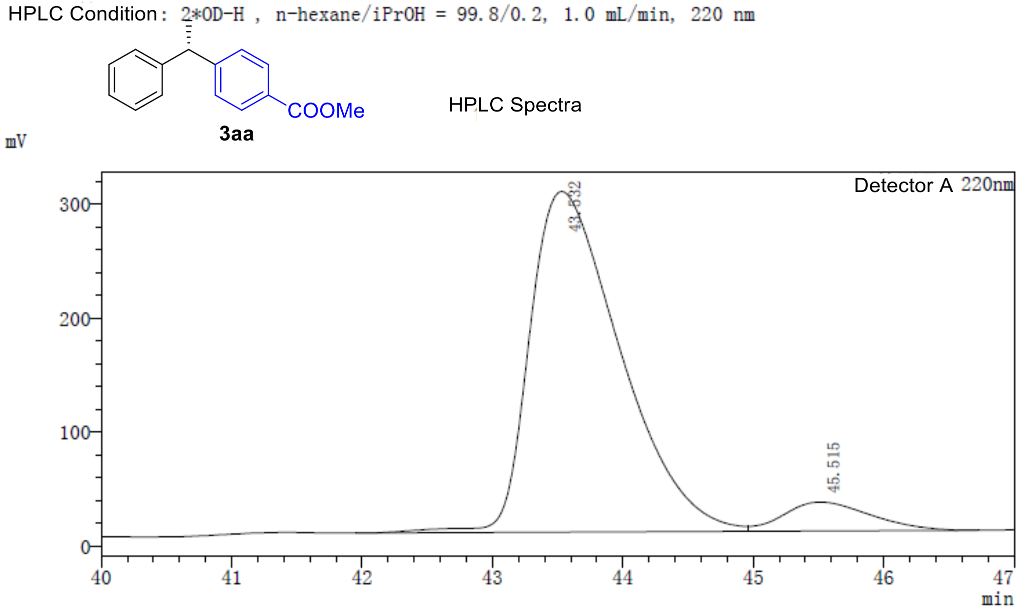

Area Percent Report

Detector A 220nm

Number	Remaining Time	Area	Height	Note	Area %
1	43.532	14453215	298835		92.438
2	45.515	1182381	25464	V	7.562
Total		15635596	324299		100.000

Supplementary Figure 108. HPLC spectra for **3aa**

HPLC Condition : OD-H*2, n-hexane:iPrOH = 99:1, 1.0 ml/min, 220 nm

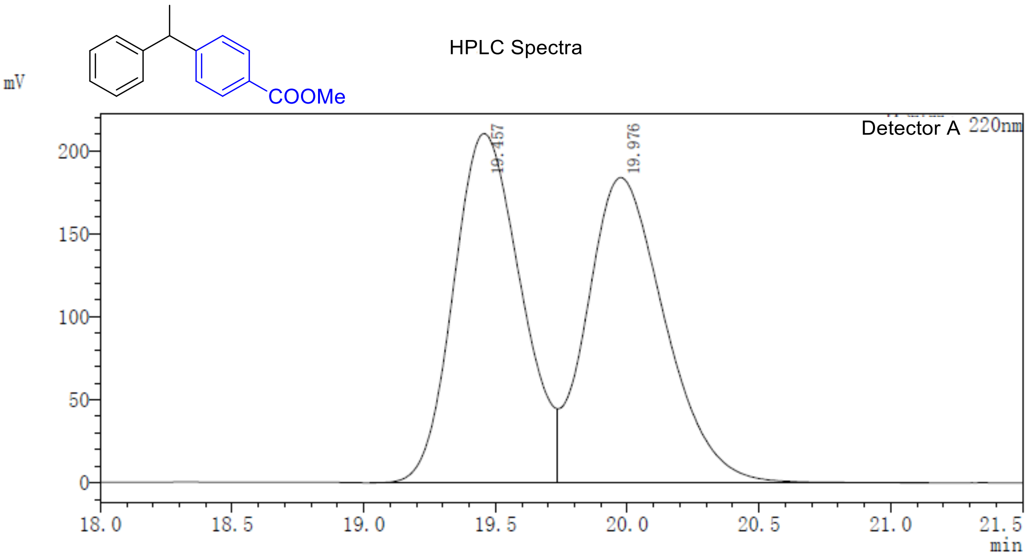

Area Percent Report

Detector A 220nm

Number	Remaining Time	Area	Height	Note	Area %
1	19.457	3743737	210400		49.629
2	19.976	3799785	183906	V	50.371
Total		7543523	394306		100.000

HPLC Condition : OD-H*2, n-Hexane/iPrOH = 99/1, 1.0 mL/min, 220 nm

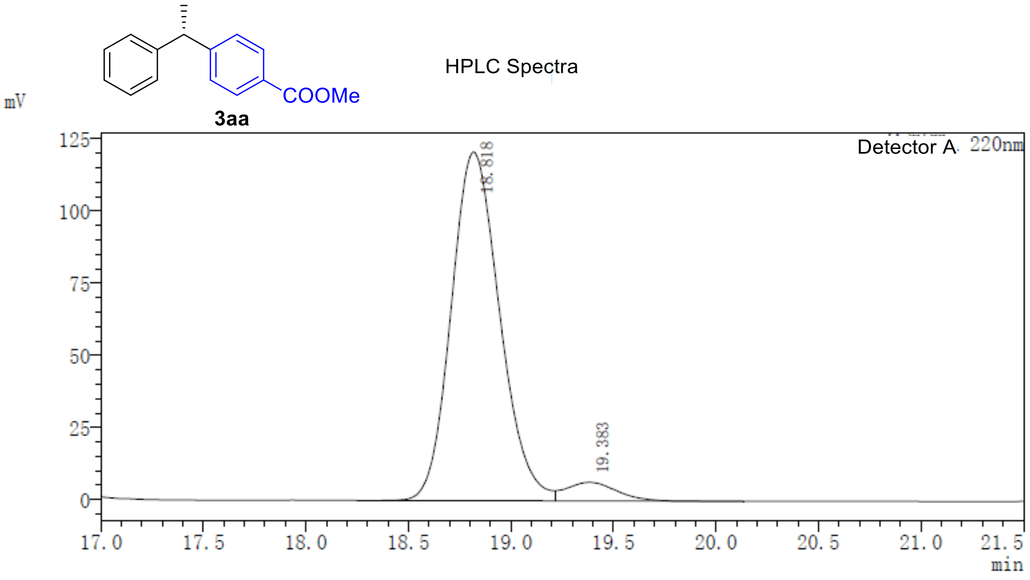

Area Percent Report

Detector A 220nm

Number	Remaining Time	Area	Height	Note	Area %
1	18.818	1987956	120817		94.695
2	19.383	111372	6470	V	5.305
Total		2099328	127287		100.000

Supplementary Figure 109. HPLC spectra for **3aa**

HPLC Condition ： AS-H*2, n-hexane/iPrOH = 99.9/0.1, 1.0 ml/min, 220 nm

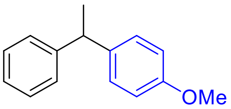

HPLC Spectra

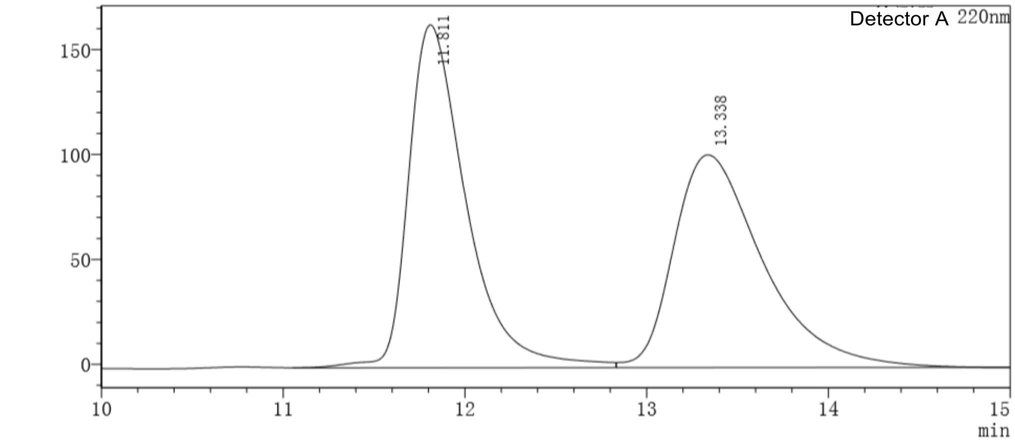

Area Percent Report

Detector A 220nm

Number	Remaining Time	Area	Height	Note	Area %
1	11.811	3607564	163376		50.932
2	13.338	3475491	101369	V	49.068
Total		7083054	264745		100.000

HPLC Condition： 2*AS-H , n-hexane/iPrOH = 99.9/0.1, 1.0 mL/min, 220 nm

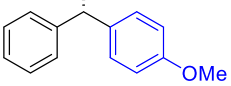

3ab

HPLC Spectra

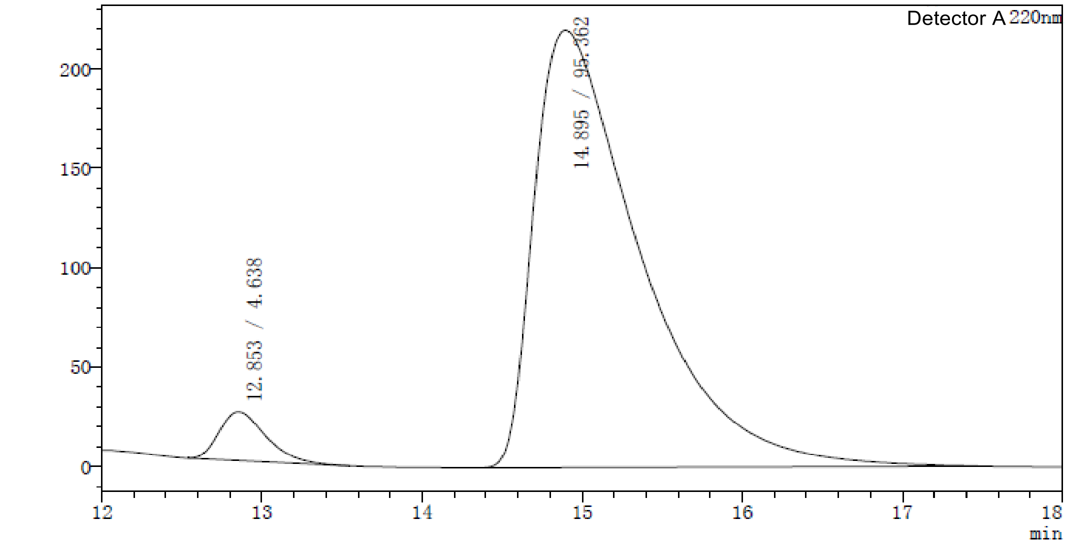

Area Percent Report

Detector A 220nm

Number	Remaining Time	Area	Height	Note	Area %
1	12.853	494115	24422		4.638
2	14.895	10158935	219916	M	95.362
Total		10653050	244338		100.000

Supplementary Figure 110. HPLC spectra for **3ab**

HPLC Condition : OD-H, n-hexane:iPrOH = 99.9/0.1, 1.0 ml/min, 220 nm

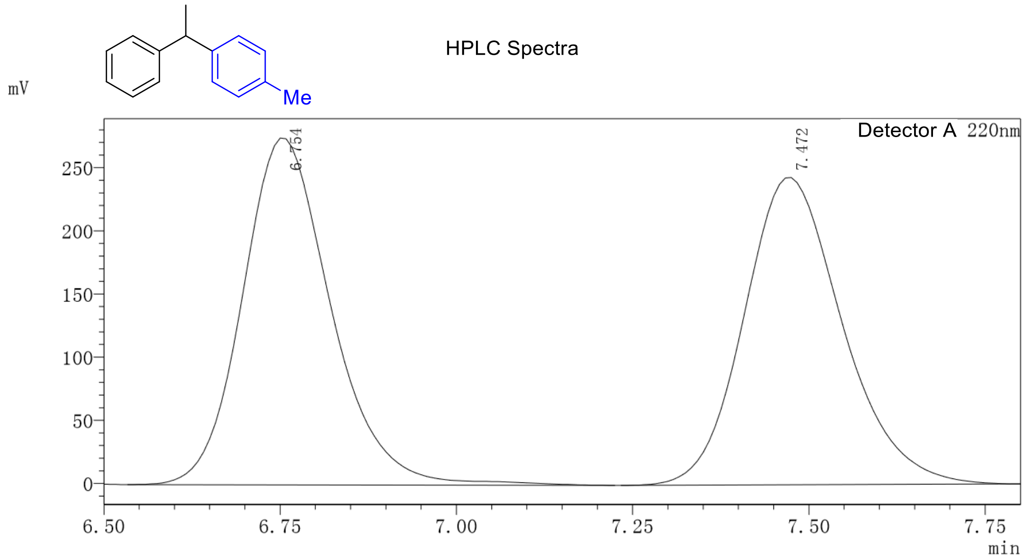

Area Percent Report

Detector A 220nm

Number	Remaining Time	Area	Height	Note	Area %
1	6.754	2394408	274464		50.406
2	7.472	2355801	243332		49.594
Total		4750208	517796		100.000

HPLC Condition : OD-H, n-hexane/iPrOH = 99.9/0.1, 1.0 ml/min, 220 nm

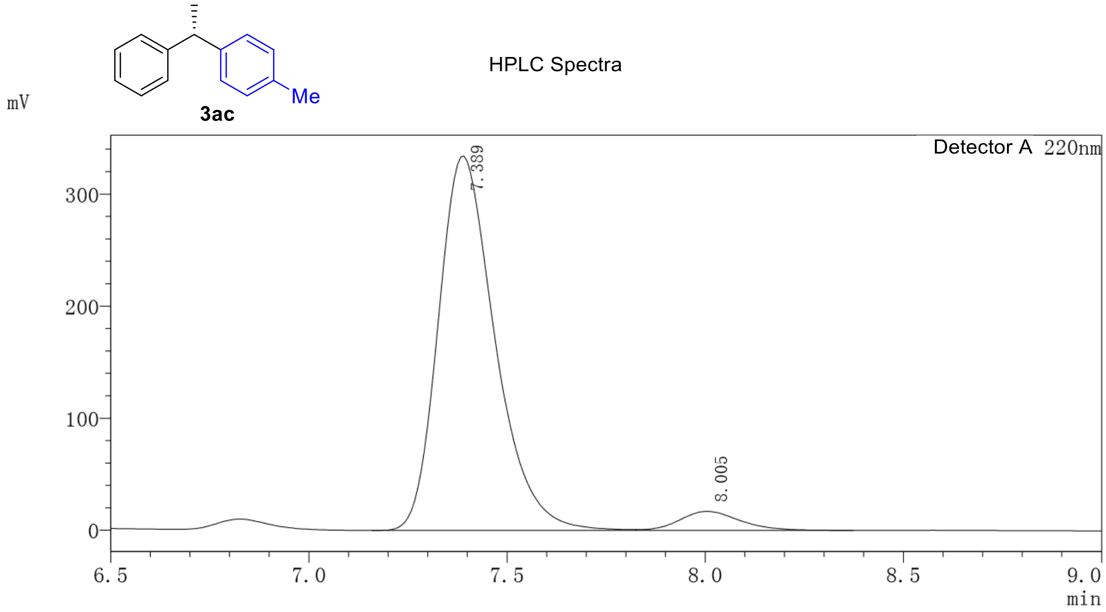

Area Percent Report

Detector A 220nm

Number	Remaining Time	Area	Height	Note	Area %
1	7.389	3318905	334065		94.928
2	8.005	177327	16950	V	5.072
Total		3496232	351015		100.000

Supplementary Figure 111. HPLC spectra for **3ac**

HPLC Condition : OD-H, n-hexane:iPrOH = 100/0, 0.5 ml/min, 220 nm

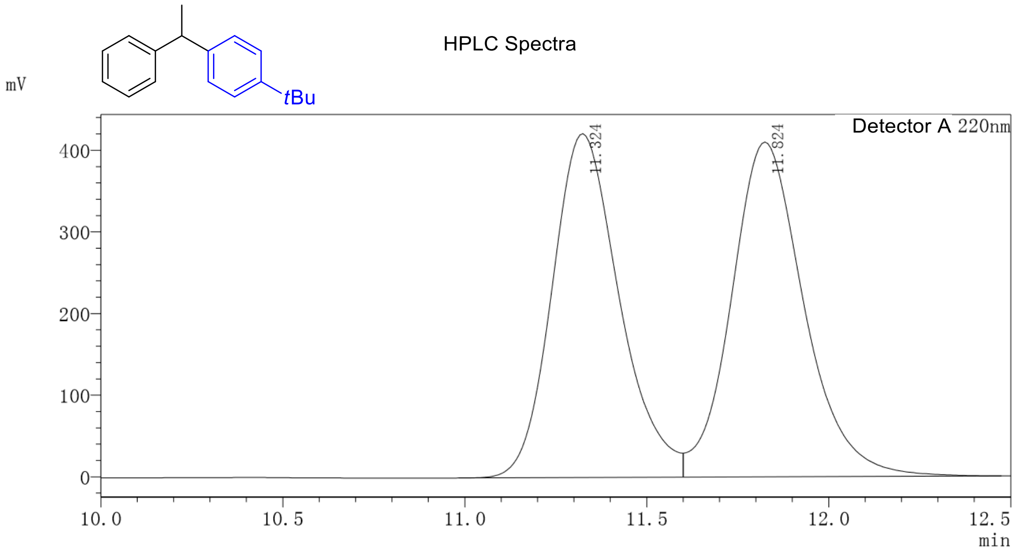

Area Percent Report

Detector A 220nm

Number	Remaining Time	Area	Height	Note	Area %
1	11.324	5420759	421188		48.795
2	11.824	5688431	410130	V	51.205
Total		11109189	831318		100.000

HPLC Condition : OD-H, n-hexane:iPrOH = 100/0, 0.5 ml/min, 220 nm

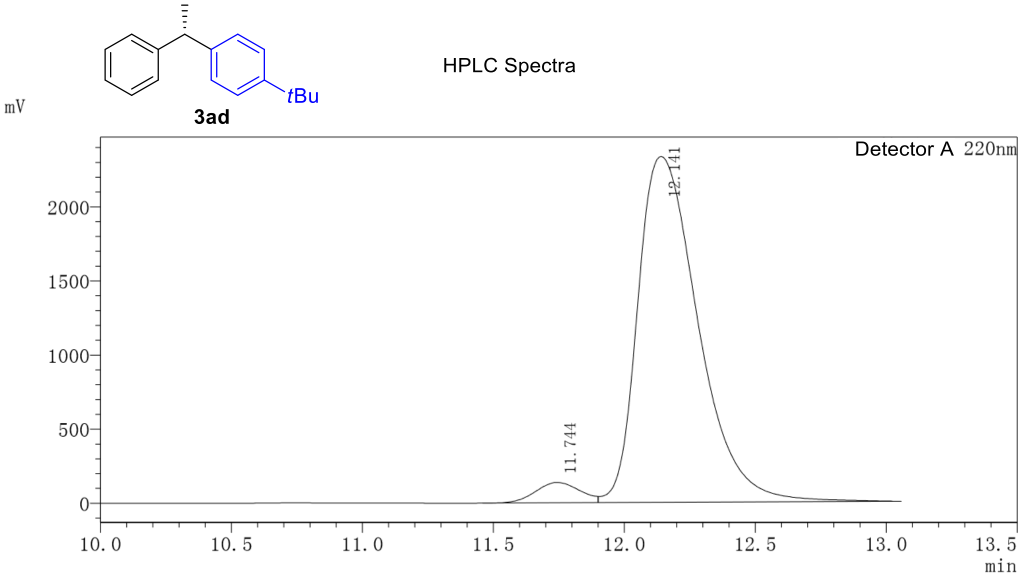

Area Percent Report

Detector A 220nm

Number	Remaining Time	Area	Height	Note	Area %
1	11.744	1641340	137627		4.210
2	12.141	37346616	2333531	V	95.790
Total		38987956	2471157		100.000

Supplementary Figure 112. HPLC spectra for **3ad**

HPLC Condition ： OJ-H, n-hexane/iPrOH = 100/0, 1.0 ml/min, 220 nm

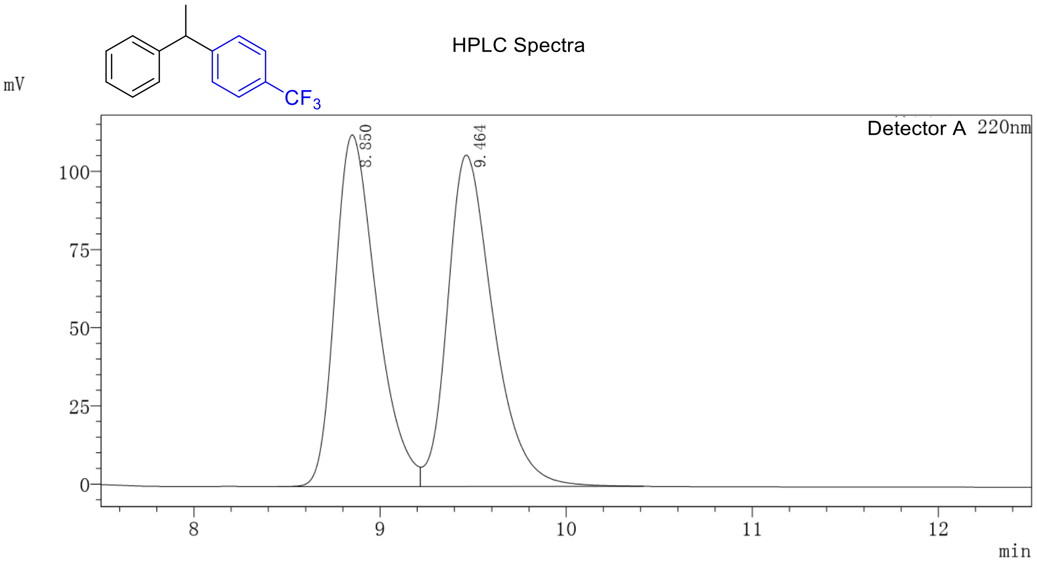

Area Percent Report

Detector A 220nm

Number	Remaining Time	Area	Height	Note	Area %
1	8.850	1752539	112472		49.328
2	9.464	1800259	105969	V M	50.672
Total		3552798	218441		100.000

HPLC Condition ： OJ-H, n-hexane:iPrOH = 100/0, 1.0 ml/min, 220 nm

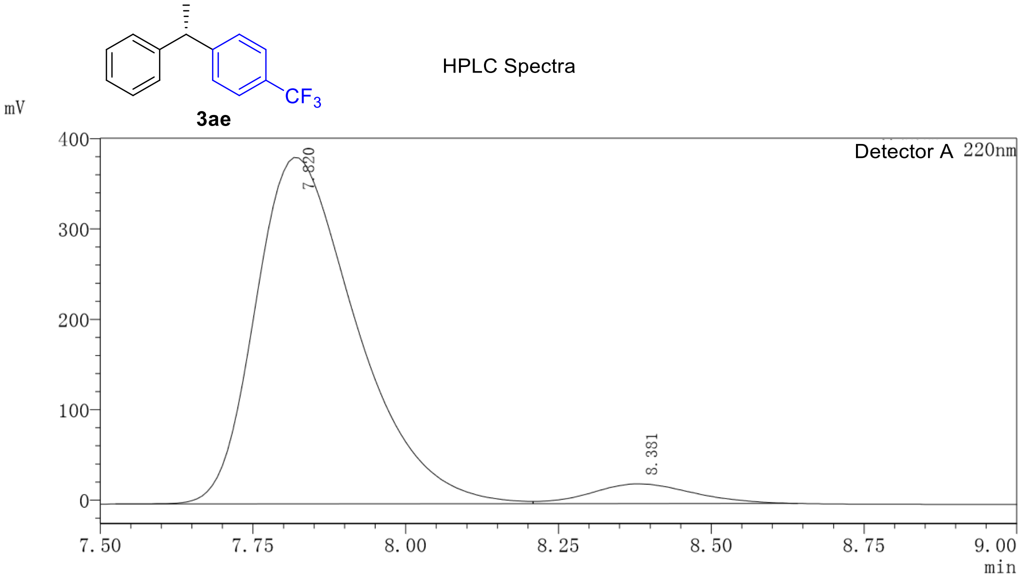

Area Percent Report

Detector A 220nm

Number	Remaining Time	Area	Height	Note	Area %
1	7.820	4414870	383264	M	94.579
2	8.381	253035	21867	V M	5.421
Total		4667905	405131		100.000

Supplementary Figure 113. HPLC spectra for **3ae**

HPLC Condition: OJ-H, n-hexane/iPrOH = 100/0, 1.0 mL/min, 220 nm

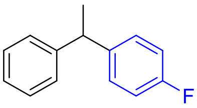

HPLC Spectra

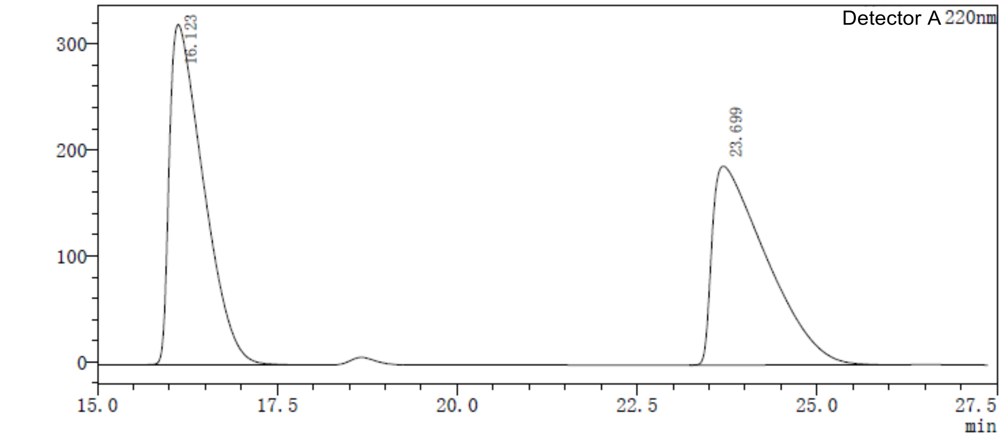

Area Percent Report

Detector A 220nm

Number	Remaining Time	Area	Height	Note	Area %
1	16.123	10536353	320953		51.652
2	23.699	9862351	187645		48.348
Total		20398704	508598		100.000

HPLC Condition
描述 : OJ-H, n-hexane/iPrOH = 100/0, 1.0 mL/min, 220 nm

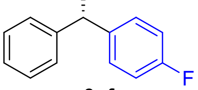

3af

HPLC Spectra
色谱图

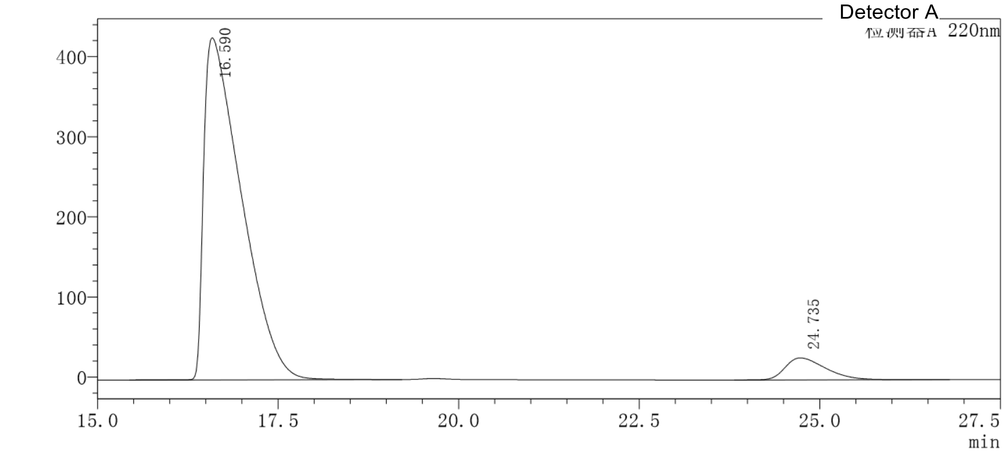

Area Percent Report
峰表

Detector

Number	Remaining Time	Area	Height	Note	Area %
峰号	保留时间	面积	高度	标记	面积%
1	16.590	15494156	426939		93.495
Total	24.735	1077951	27468		6.505
总计		16572107	454407		100.000

Supplementary Figure 114. HPLC spectra for **3af**

HPLC Condition ： OJ-H ， n-hexane/iPrOH = 98/2， 1.0 mL/min， 220 nm

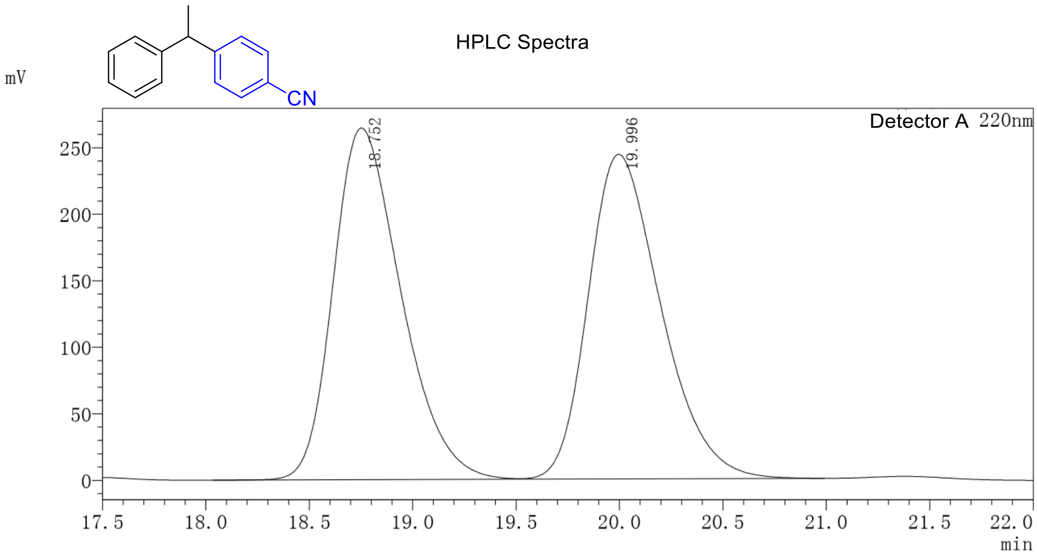

Area Percent Report

Detector :A 220nm

Number	Remaining Time	Area	Height	Note	Area %
1	18.752	6041212	264352		50.259
2	19.996	5978884	243979	V	49.741
Total		12020096	508331		100.000

HPLC Condition ： OJ-H ， n-hexane/iPrOH = 98/2， 1.0 mL/min， 220 nm

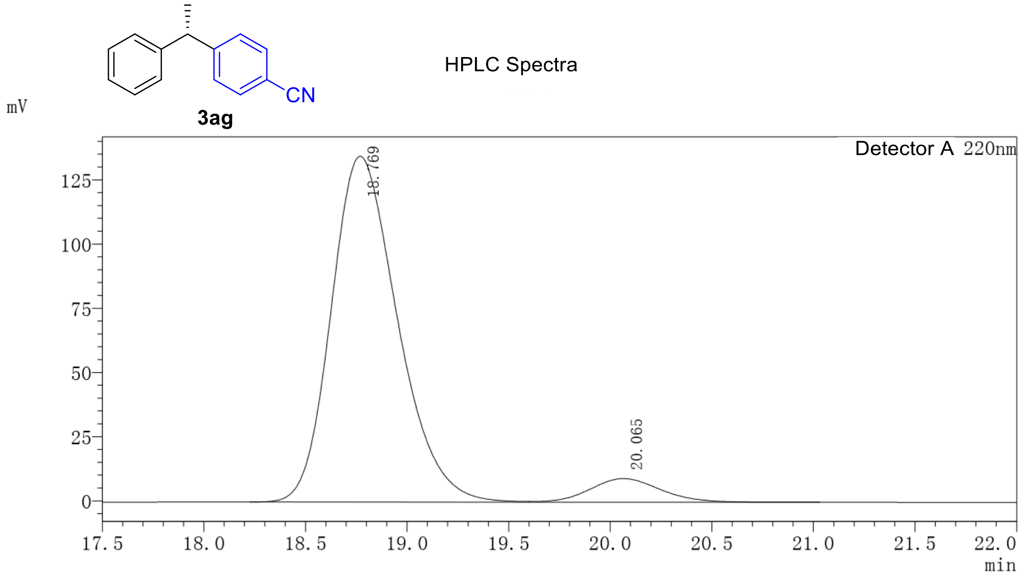

Area Percent Report

Detector :A 220nm

Number	Remaining Time	Area	Height	Note	Area %
1	18.769	3052744	134685		93.225
2	20.065	221855	9178	V	6.775
Total		3274599	143864		100.000

Supplementary Figure 115. HPLC spectra for **3ag**

HPLC Condition : OJ-H, n-hexane:iPrOH = 100/0, 1.0 ml/min, 220 nm

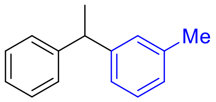

HPLC Spectra

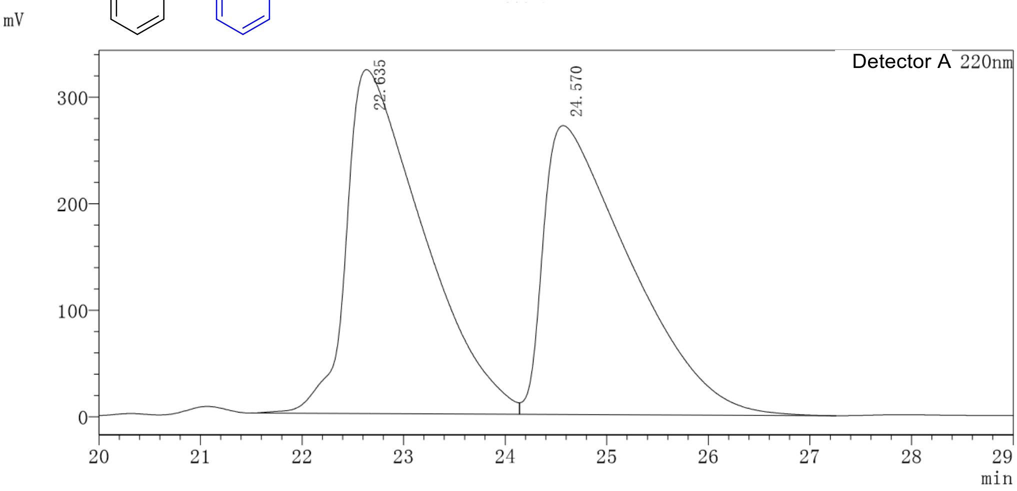

Area Percent Report

Detector A 220nm

Number	Remaining Time	Area	Height	Note	Area %
1	22.635	17039447	322875		51.347
2	24.570	16145708	271153	V	48.653
Total		33185155	594028		100.000

HPLC Condition : OJ-H, n-hexane:iPrOH = 100/0, 1.0 ml/min, 220 nm

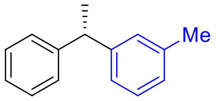

3ah

HPLC Spectra

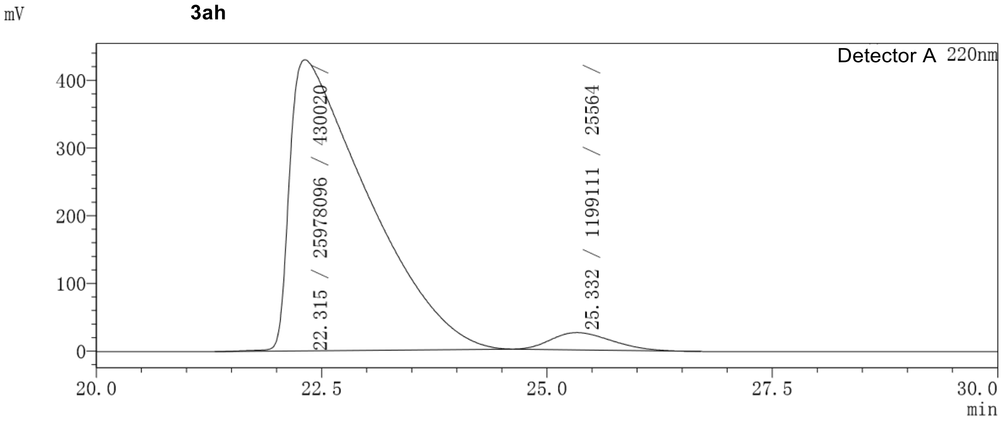

Area Percent Report

Detector A 220nm

Number	Remaining Time	Area	Height	Note	Area %
1	22.315	25978096	430020		95.588
2	25.332	1199111	25564	M	4.412
Total		27177207	455585		100.000

Supplementary Figure 116. HPLC spectra for **3ah**

HPLC Condition : OJ-H, n-hexane:iPrOH = 100/0, 1.0 ml/min, 220 nm

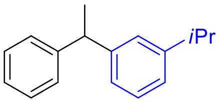

HPLC Spectra

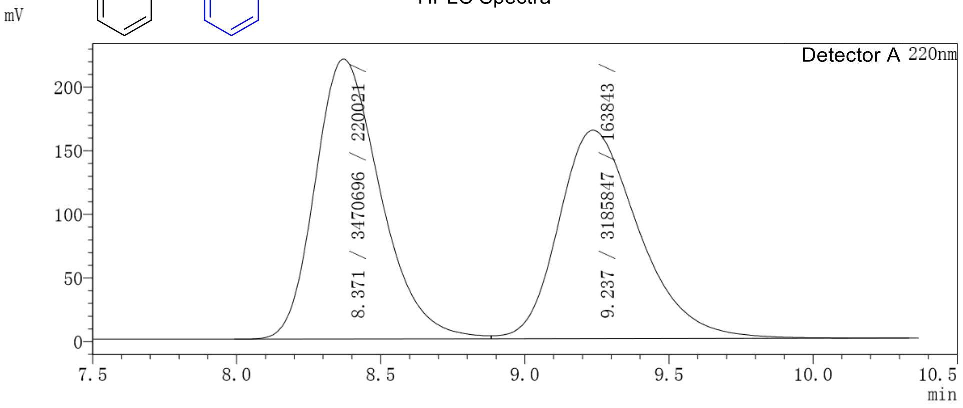

Area Percent Report

Detector A 220nm

Number	Remaining Time	Area	Height	Note	Area %
1	8.371	3470696	220021		52.140
2	9.237	3185847	163843	V	47.860
Total		6656543	383864		100.000

HPLC Condition : Oj-H, n-hexane:iPrOH = 100/0, 1.00 ml/min, 220 nm

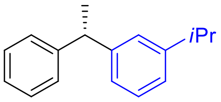

3ai

HPLC Spectra

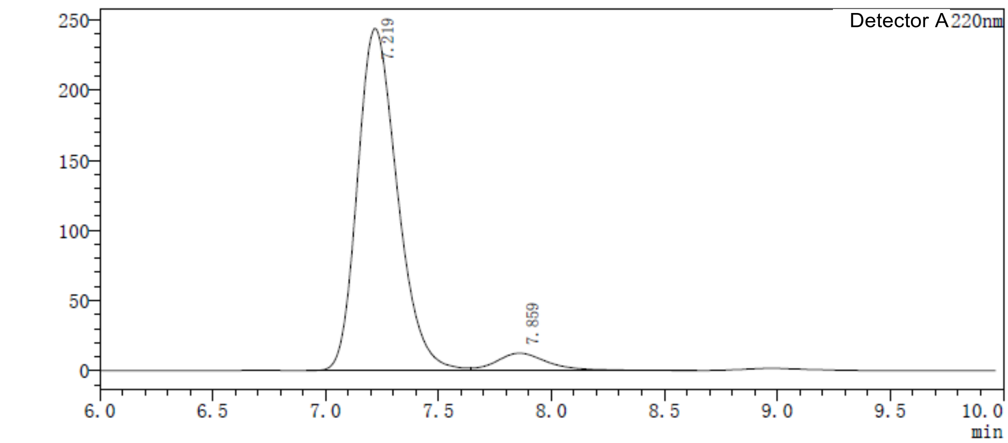

Area Percent Report

Detector A 220nm

Number	Remaining Time	Area	Height	Note	Area %
1	7.219	2984925	243752		93.914
2	7.859	193426	12342	V	6.086
Total		3178351	256094		100.000

Supplementary Figure 117. HPLC spectra for **3ai**

HPLC Condition : 2*OD-H , n-hexane/iPrOH = 98/2, 1.0 mL/min, 220 nm

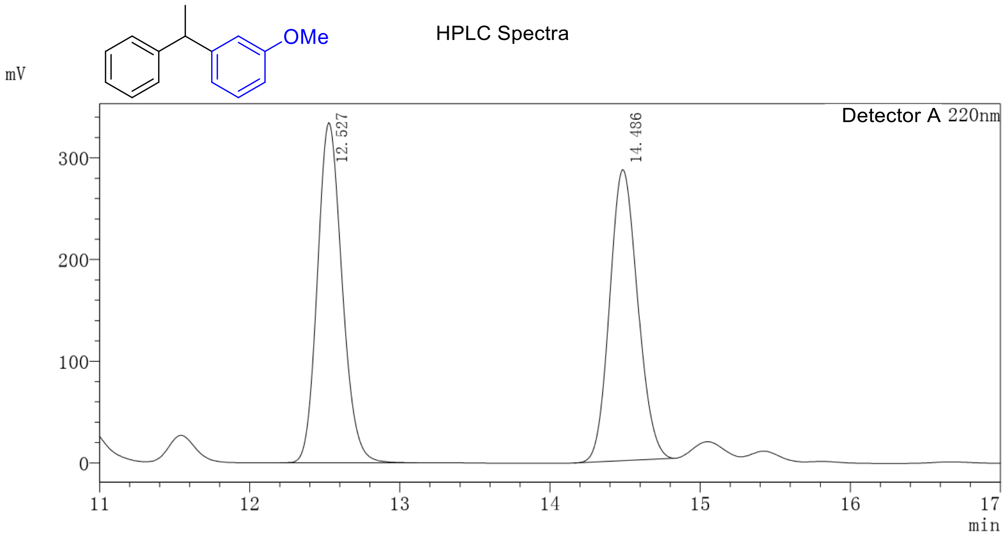

Area Percent Report

Detector A 220nm

Number	Remaining Time	Area	Height	Note	Area %
1	12.527	3824349	334389		50.711
2	14.486	3717173	286265		49.289
Total		7541522	620654		100.000

HPLC Condition : 2*OD-H , n-hexane/iPrOH = 98/2, 1.0 mL/min, 220 nm

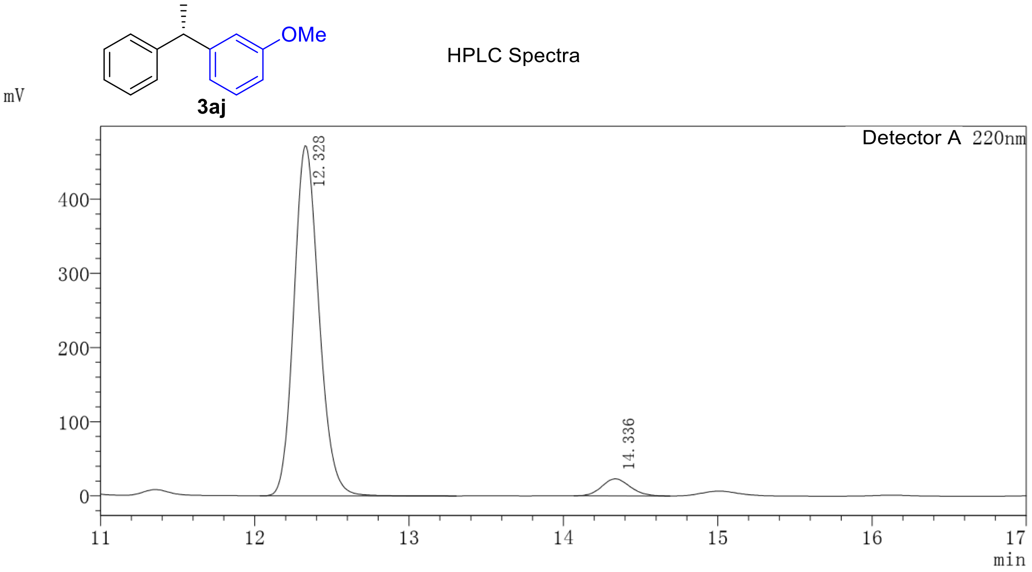

Area Percent Report

Detector A 220nm

Number	Remaining Time	Area	Height	Note	Area %
1	12.328	5319696	471890		94.834
2	14.336	289762	22825		5.166
Total		5609458	494715		100.000

Supplementary Figure 118. HPLC spectra for **3aj**

HPLC Condition ： OJ-H, n-hexane/iPrOH =100/0, 1.0 ml/min, 220 nm

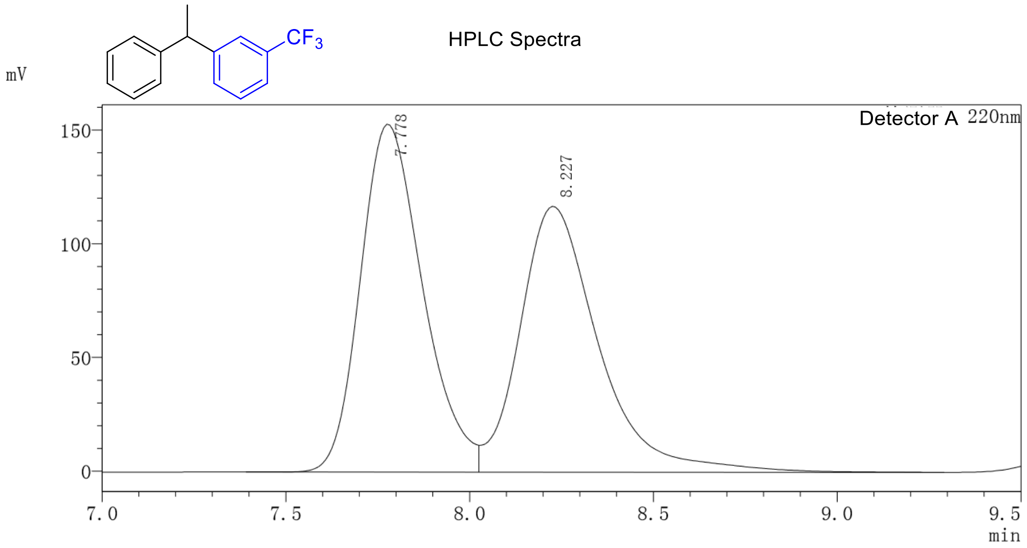

Area Percent Report

Detector A 220nm

Number	Remaining Time	Area	Height	Note	Area %
1	7.778	1810130	152884		51.053
2	8.227	1735427	116838	V	48.947
Total		3545557	269722		100.000

HPLC Condition ： Oj-H, n-hexane:iPrOH = 100/0, 1.00 ml/min, 220 nm

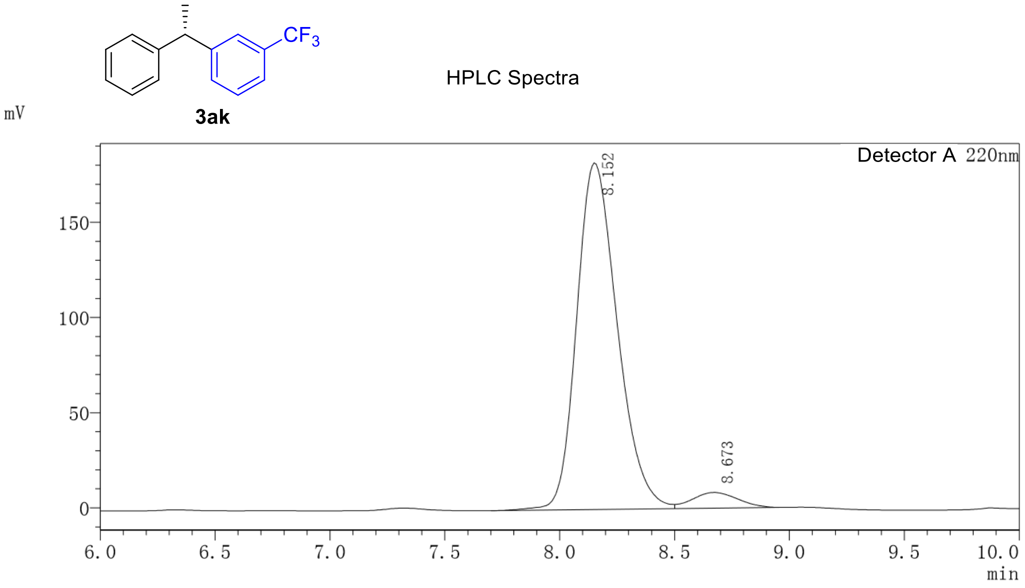

Area Percent Report

Detector A 220nm

Number	Remaining Time	Area	Height	Note	Area %
1	8.152	2243386	181983		95.202
2	8.673	113070	8246	V	4.798
Total		2356457	190229		100.000

Supplementary Figure 119. HPLC spectra for **3ak**

HPLC Condition ： OJ-H, n-hexane:iPrOH = 85/15, 1.0 ml/min, 220 nm

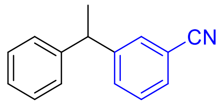

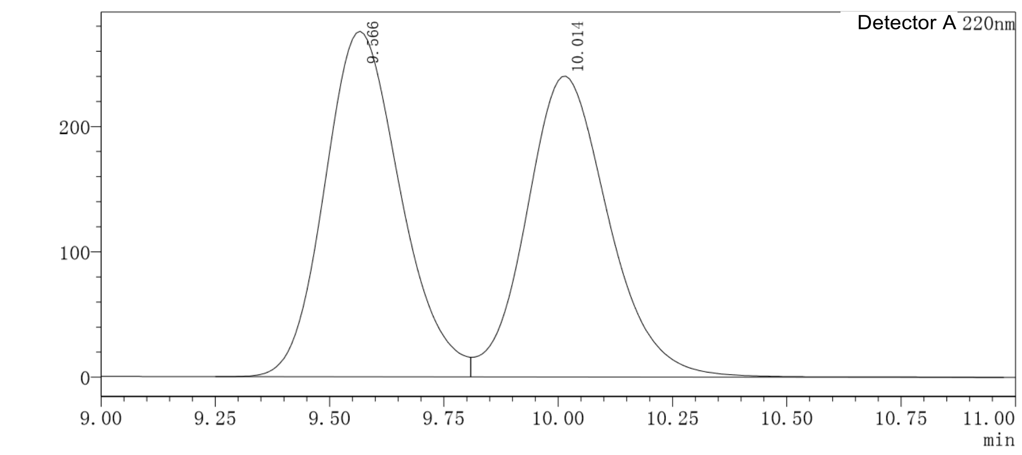

Area Percent Report

Detector A 220nm

Number	Remaining Time	Area	Height	Note	Area %
1	9.566	3182760	275530		51.471
2	10.014	3000820	239859	SV	48.529
Total		6183580	515389		100.000

HPLC Condition ： OJ-H, n-hexane:iPrOH = 85/15, 1.0 ml/min, 220 nm

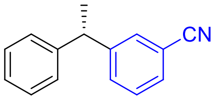

3al

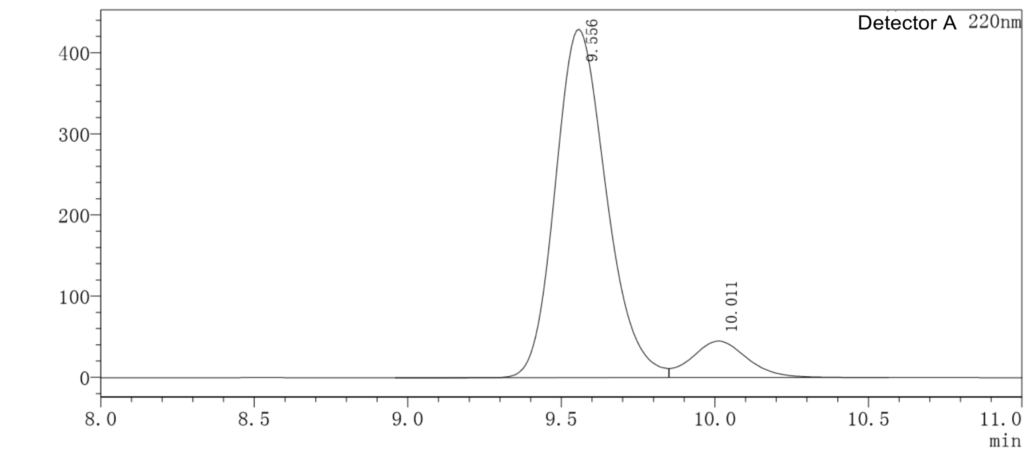

Area Percent Report

Detector A 220nm

Number	Remaining Time	Area	Height	Note	Area %
1	9.556	4975085	429310		89.802
2	10.011	564978	45015	V	10.198
Total		5540064	474325		100.000

Supplementary Figure 120. HPLC spectra for **3al**

HPLC Condition : OJ-H , n-hexane/iPrOH = 99/1, 1.0 mL/min, 220 nm

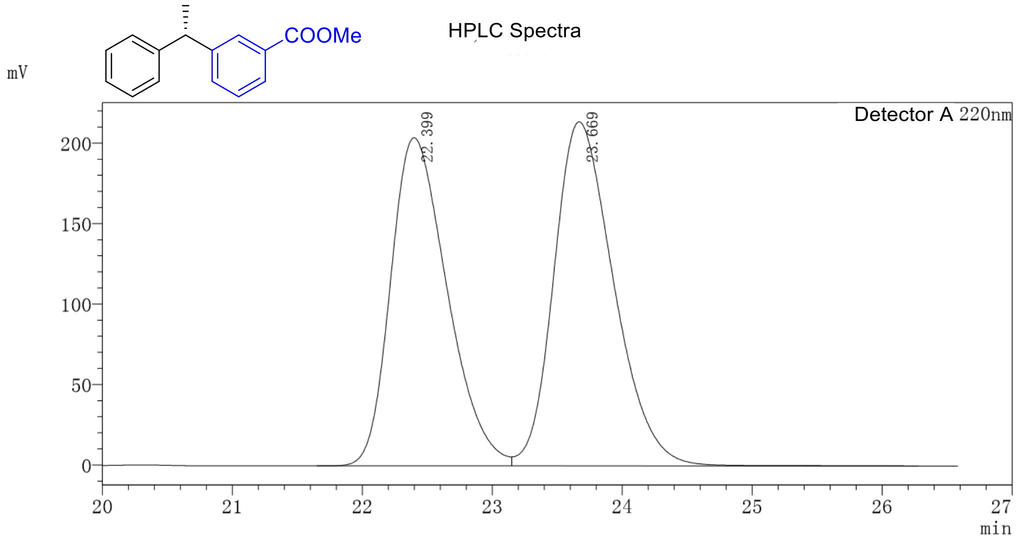

Area Percent Report

Detector A 220nm

Number	Remaining Time	Area	Height	Note	Area %
1	22.399	6162784	203882		47.384
2	23.669	6843192	213776	V	52.616
Total		13005976	417658		100.000

HPLC Condition : OJ-H, n-hexane:iPrOH = 99/1, 1.0 ml/min, 220 nm

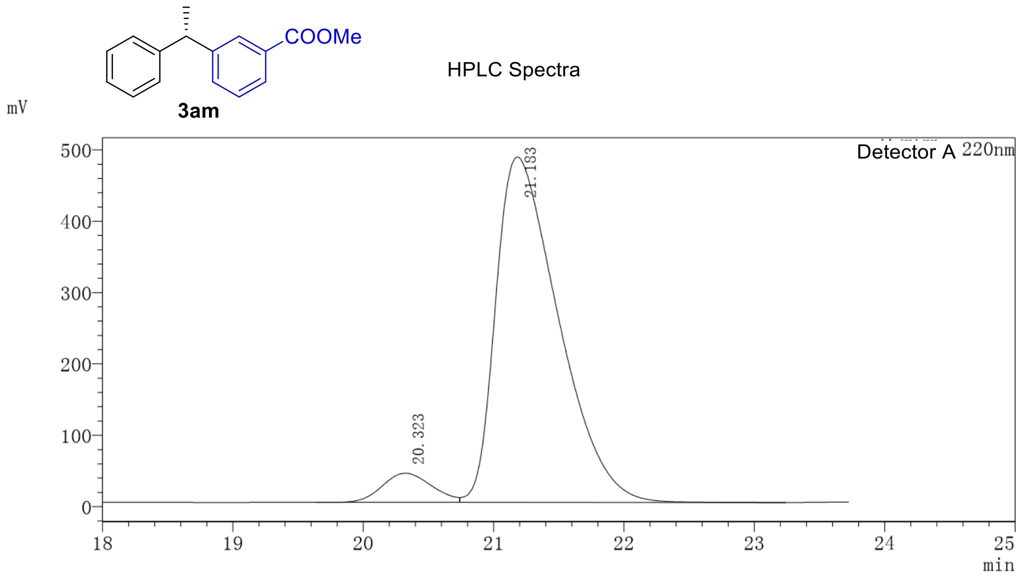

Area Percent Report

Detector A 220nm

Number	Remaining Time	Area	Height	Note	Area %
1	20.323	1087745	41063		6.291
2	21.183	16201438	484217	V	93.709
Total		17289183	525281		100.000

Supplementary Figure 121. HPLC spectra for **3am**

HPLC Condition ： OJ-H ， n-hexane/iPrOH = 99/1， 1.0 mL/min， 220 nm

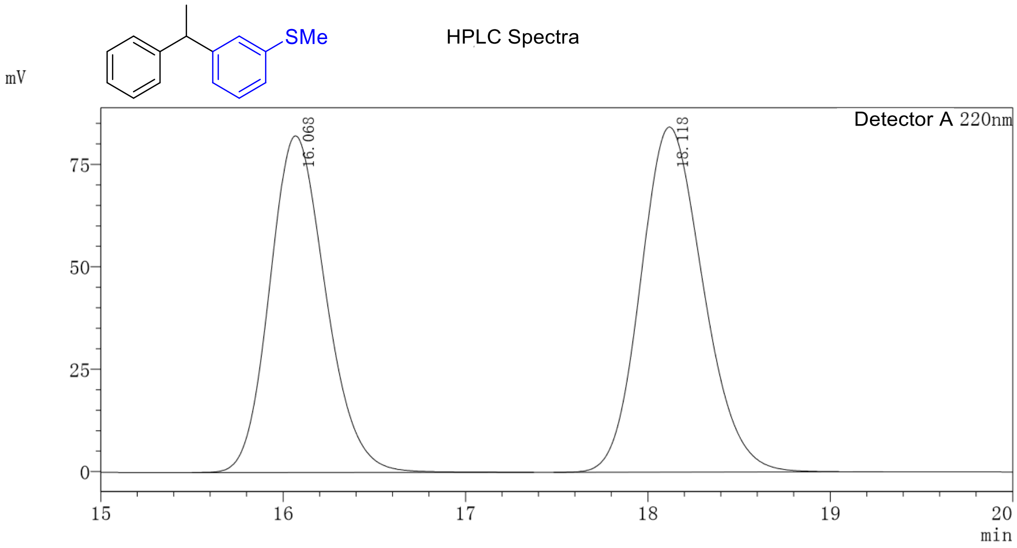

Area Percent Report

Detector A 220nm

Number	Remaining Time	Area	Height	Note	Area %
1	16.068	1752284	82166		46.574
2	18.118	2010067	84235		53.426
Total		3762351	166401		100.000

HPLC Condition ： OJ-H ， n-hexane/iPrOH = 99/1， 1.0 mL/min， 220 nm

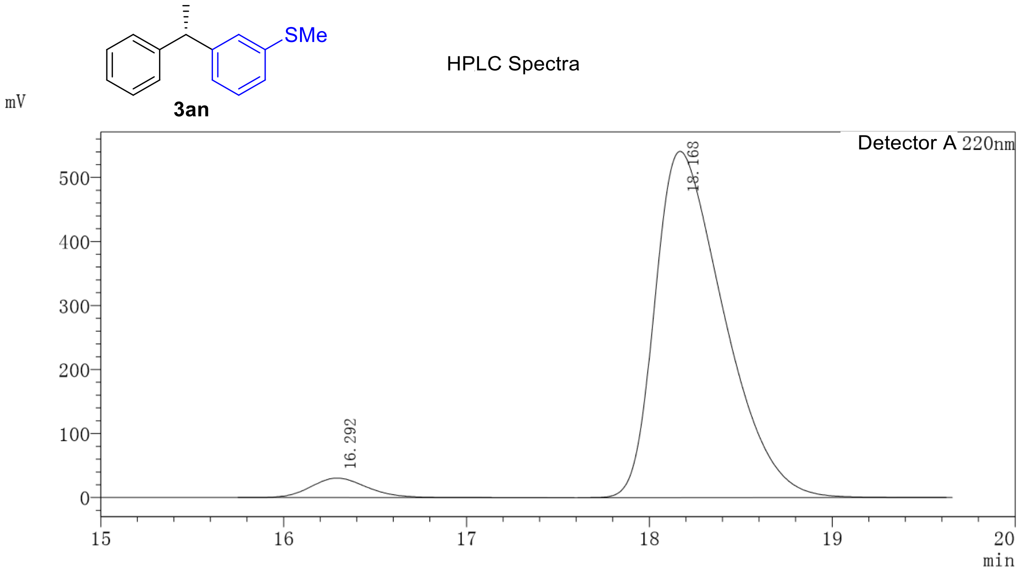

Area Percent Report

Detector A 220nm

Number	Remaining Time	Area	Height	Note	Area %
1	16.292	653984	30451		4.418
2	18.168	14148977	540928		95.582
Total		14802961	571379		100.000

Supplementary Figure 122. HPLC spectra for **3an**

HPLC Condition ： OD-H*2, n-hexane:iPrOH = 98/2, 1.0 ml/min, 220 nm

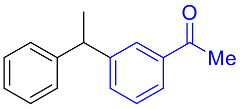

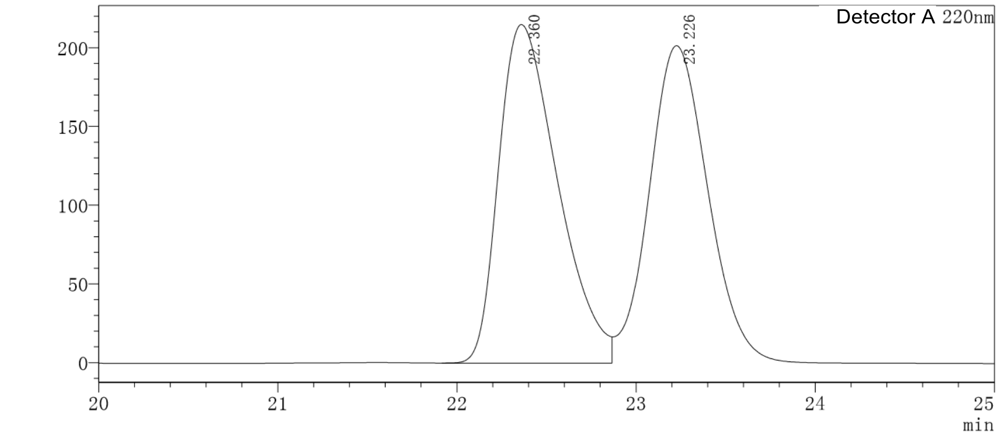

Area Percent Report

Detector A 220nm

Number	Remaining Time	Area	Height	Note	Area %
1	22.360	4918531	215090		51.600
2	23.226	4613476	201778	V	48.400
Total		9532008	416868		100.000

HPLC Condition ： OD-H*2, n-hexane:iPrOH = 98/2, 1.0 ml/min, 220 nm

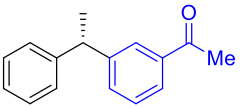

3ao

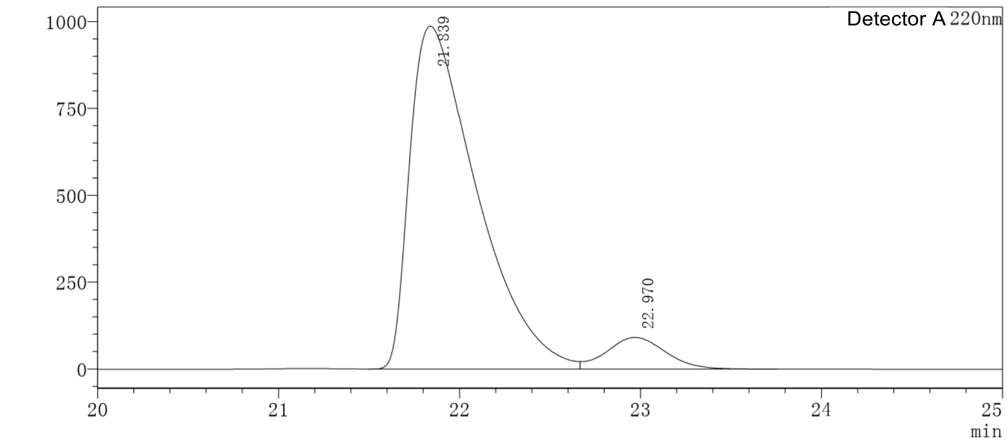

Area Percent Report

Detector A 220nm

Number	Remaining Time	Area	Height	Note	Area %
1	21.839	25501228	986852		92.552
2	22.970	2052121	91024	V M	7.448
Total		27553349	1077875		100.000

Supplementary Figure 123. HPLC spectra for **3ao**

HPLC Condition : OJ-H, n-hexane:iPrOH = 80/20, 1.0 ml/min, 220 nm

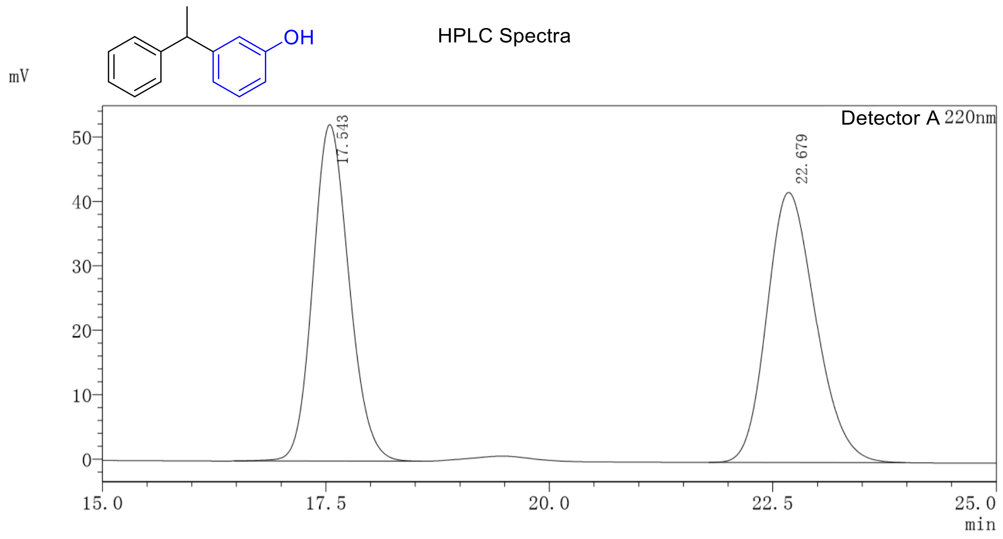

Area Percent Report

Detector A 220nm

Number	Remaining Time	Area	Height	Note	Area %
1	17.543	1470405	52207		48.029
2	22.679	1591089	41900	M	51.971
Total		3061495	94107		100.000

HPLC Condition : OJ-H, n-hexane:iPrOH = 80/20, 1.0 ml/min, 220 nm

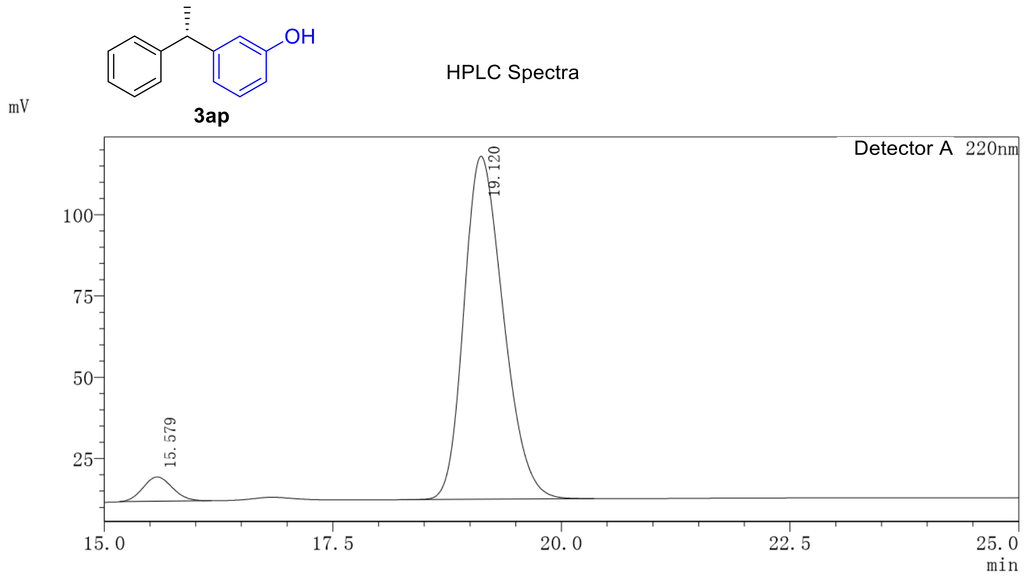

Area Percent Report

Detector A 220nm

Number	Remaining Time	Area	Height	Note	Area %
1	15.579	168936	7498	M	5.087
2	19.120	3151679	105517		94.913
Total		3320614	113015		100.000

Supplementary Figure 124. HPLC spectra for **3ap**

HPLC Condition : OJ-H , n-hexane/iPrOH = 90/10, 1.0 mL/min, 220 nm

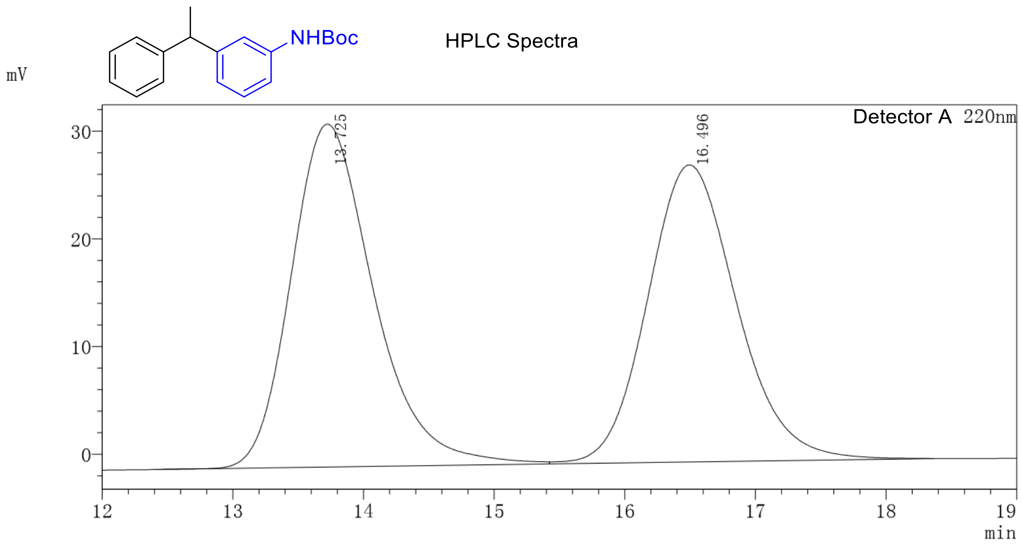

Area Percent Report

Detector A 220nm

Number	Remaining Time	Area	Height	Note	Area %
1	13.725	1402106	31846		51.696
2	16.496	1310094	27580	V	48.304
Total		2712201	59426		100.000

HPLC Condition : OJ-H , n-hexane/iPrOH = 90/10, 1.0 mL/min, 220 nm

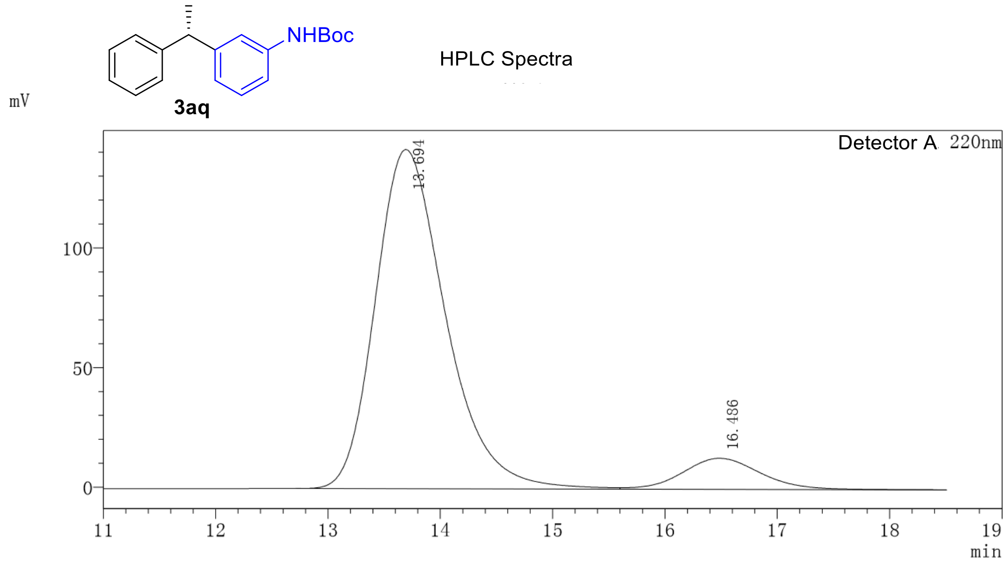

Area Percent Report

Detector A 220nm

Number	Remaining Time	Area	Height	Note	Area %
1	13.694	6164236	141700		90.766
2	16.486	627112	12982	V	9.234
Total		6791348	154681		100.000

Supplementary Figure 125. HPLC spectra for **3aq**

HPLC Condition： AD-H, n-hexane:iPrOH = 99/1, 1.0 ml/min, 220 nm

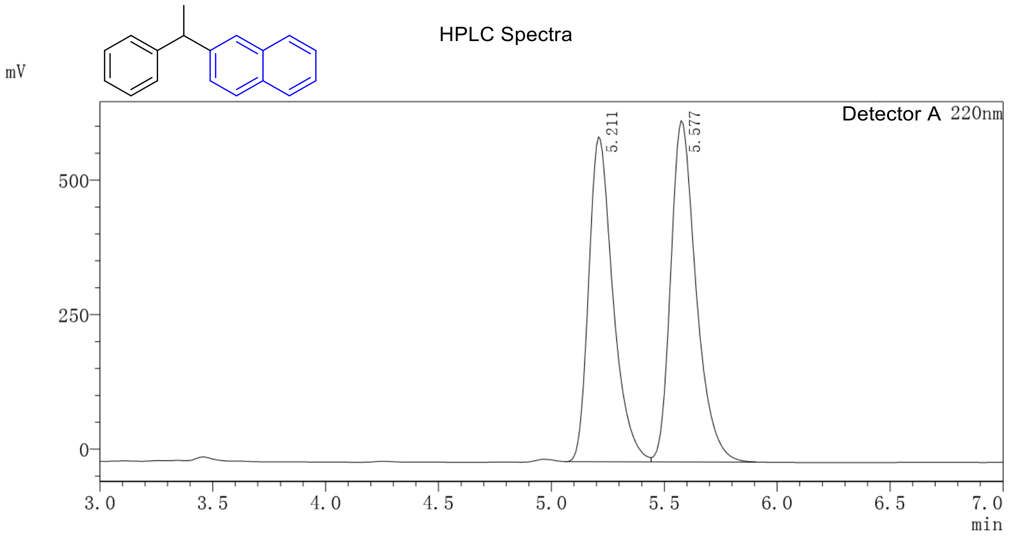

Area Percent Report

Detector A 220nm

Number	Remaining Time	Area	Height	Note	Area %
1	5.211	4532090	603352		47.685
2	5.577	4972197	633864	V M	52.315
Total		9504287	1237216		100.000

HPLC Condition ： AD-H, n-hexane/iPrOH =99/1, 1.0 mL/min, 220 nm

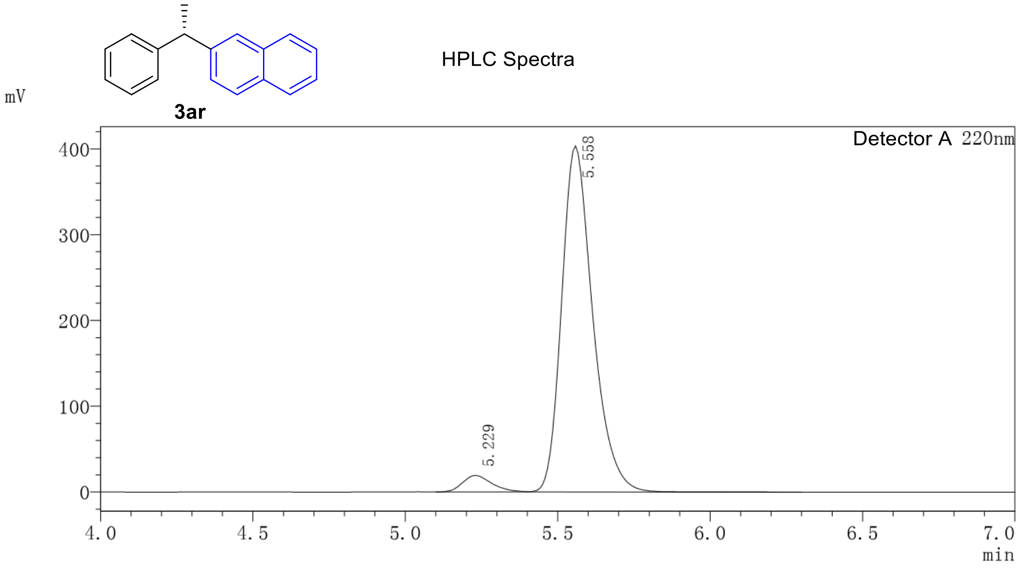

Area Percent Report

Detector A 220nm

Number	Remaining Time	Area	Height	Note	Area %
1	5.229	127974	19151		4.278
2	5.558	2863225	403300	V	95.722
Total		2991199	422451		100.000

Supplementary Figure 126. HPLC spectra for **3ar**

HPLC Condition： OJ-H, n-hexane/iPrOH = 90/10, 1.0 mL/min, 220 nm

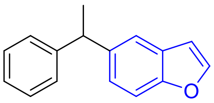

HPLC Spectra

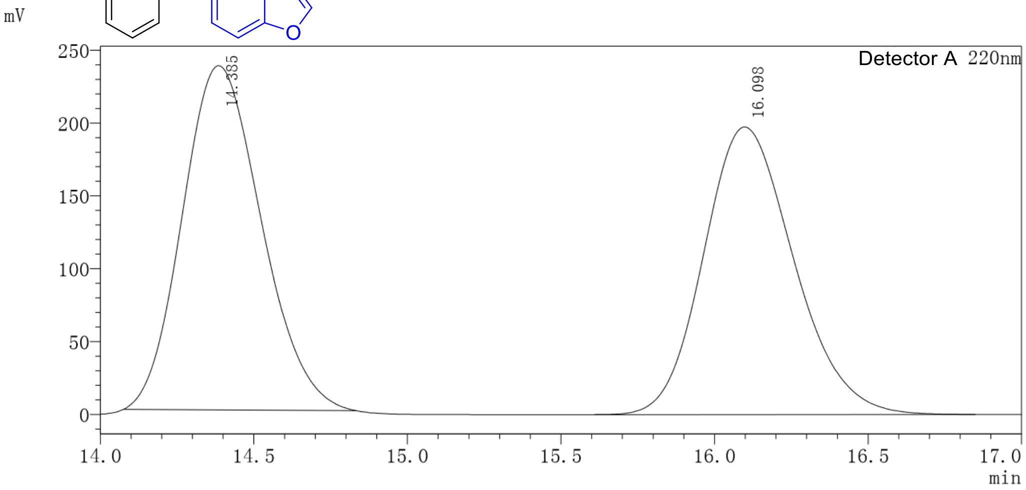

Area Percent Report

Detector A 220nm

Number	Remaining Time	Area	Height	Note	Area %
1	14.385	4232656	236199	M	51.190
2	16.098	4035877	197294	M	48.810
Total		8268533	433493		100.000

HPLC Condition： OJ-H, n-hexane/iPrOH = 90/10, 1.0 mL/min, 220 nm

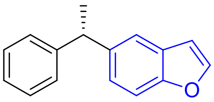

3as

HPLC Spectra

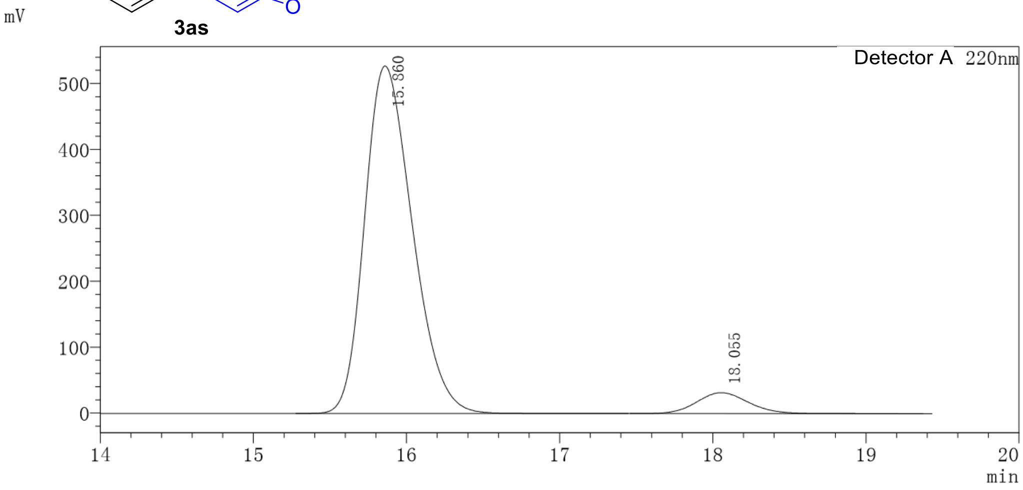

Area Percent Report

Detector A 220nm

Number	Remaining Time	Area	Height	Note	Area %
1	15.860	11425070	527181		93.900
2	18.055	742179	31555		6.100
Total		12167250	558736		100.000

Supplementary Figure 127. HPLC spectra for **3as**

HPLC Condition：OJ-H, n-hexane/iPrOH = 90/10, 1.0 mL/min, 220 nm

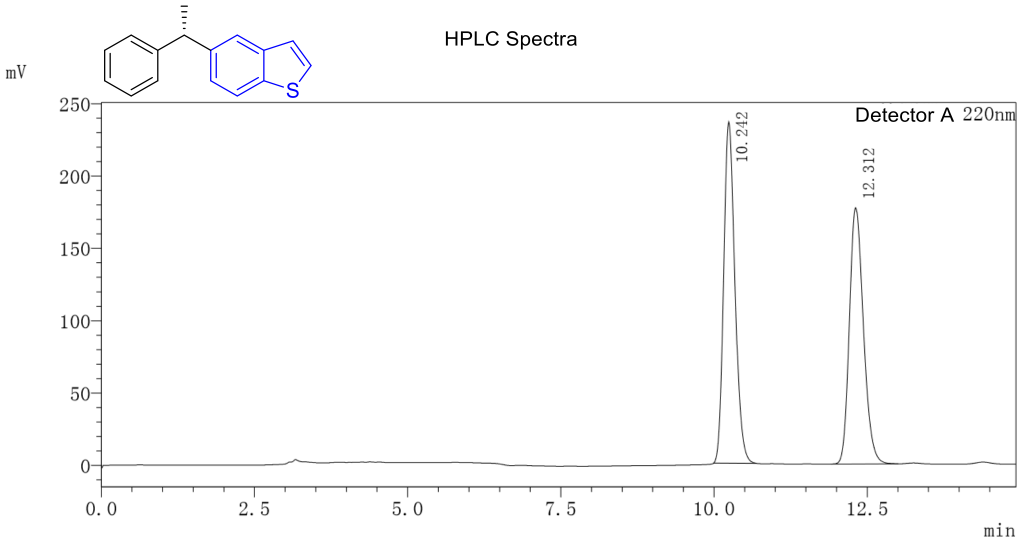

Area Percent Report

Detector A 220nm

Number	Remaining Time	Area	Height	Note	Area %
1	10.242	3013388	236082	M	51.796
2	12.312	2804359	177140		48.204
Total		5817747	413222		100.000

HPLC Condition：OJ-H, n-hexane/iPrOH = 90/10, 1.0 mL/min, 220 nm

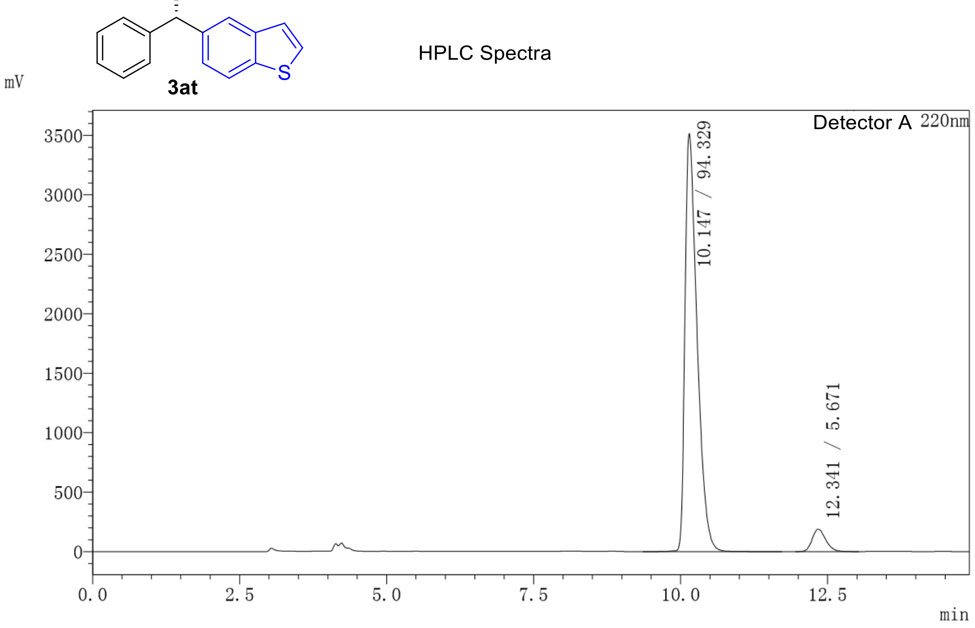

Area Percent Report

Detector A 220nm

Number	Remaining Time	Area	Height	Note	Area %
1	10.147	50234068	3514422		94.329
2	12.341	3020084	188722		5.671
Total		53254152	3703143		100.000

Supplementary Figure 128. HPLC spectra for **3at**

HPLC Condition : OJ-H, n-hexane/iPrOH = 85/15, 1.0 ml/min, 220 nm

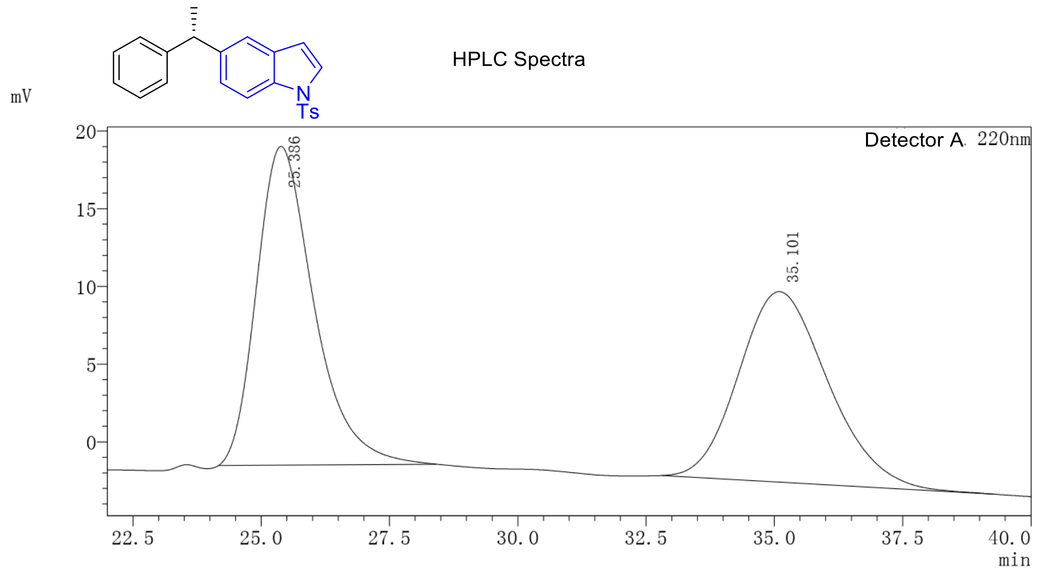

Area Percent Report

Detector A 220nm

Number	Remaining Time	Area	Height	Note	Area %
1	25.386	1596403	20505	M	51.711
2	35.101	1490783	12263		48.289
Total		3087186	32768		100.000

[illegible] : OJ-H, n-hexane:iPrOH = 85/15, 1.0 ml/min, 220 nm
HPLC Condition

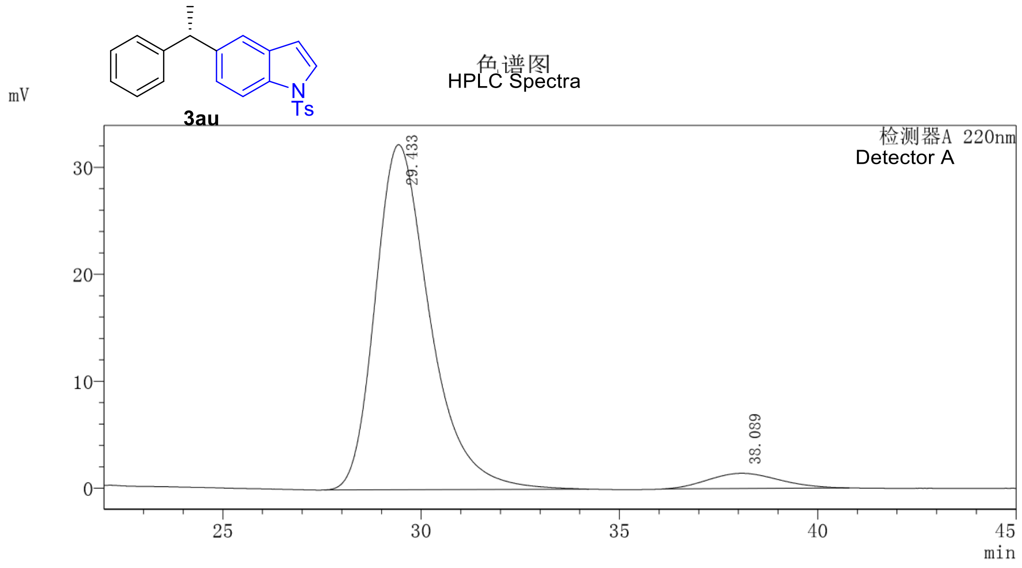

峰表

Area Percent Report

检测器A 220nm
Detector

保留时间		面积	高度	标记	面积%
Number	Remaining Time	Area	Height	Note	Area %
2	38.089	179279	1426		5.468
总计 Total		3278452	33685		100.000

Supplementary Figure 129. HPLC spectra for **3au**

HPLC Condition : OJ-H*2, n-hexane:iPrOH = 90/10, 1.0 ml/min, 220 nm

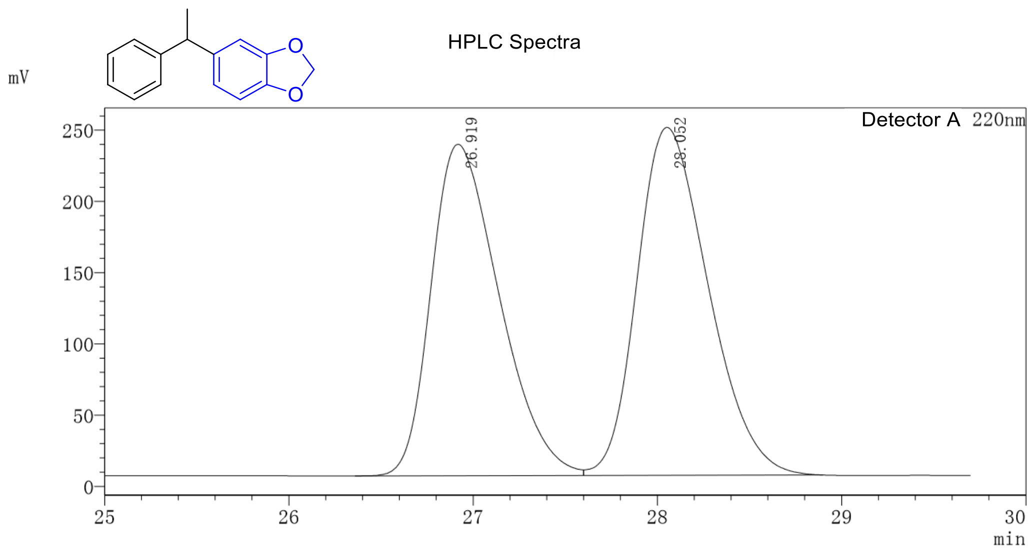

Area Percent Report

Detector A 220nm

Number	Remaining Time	Area	Height	Note	Area %
1	26.919	5982856	232614	M	47.730
2	28.052	6552022	244238	V M	52.270
Total		12534878	476853		100.000

HPLC Condition : 2*OJ-H , n-hexane/iPrOH = 90/10, 1.0 mL/min, 220 nm

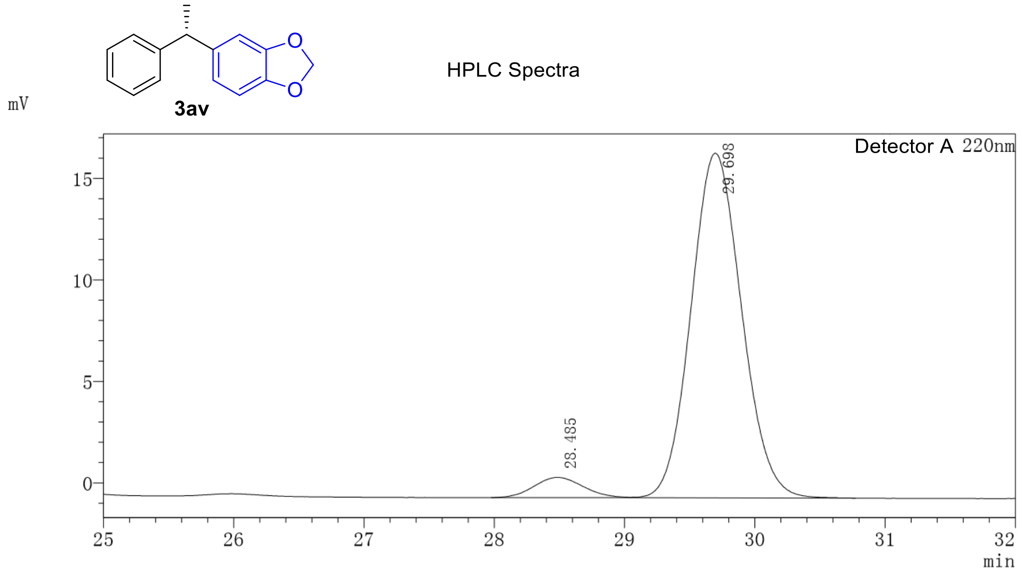

Area Percent Report

Detector A 220nm

Number	Remaining Time	Area	Height	Note	Area %
1	28.485	26218	1000		5.412
2	29.698	458239	16978	V	94.588
Total		484457	17978		100.000

Supplementary Figure 130. HPLC spectra for **3av**

HPLC Condition ： OJ-H, n-hexane/iPrOH = 90/10, 1.0 mL/min, 220 nm

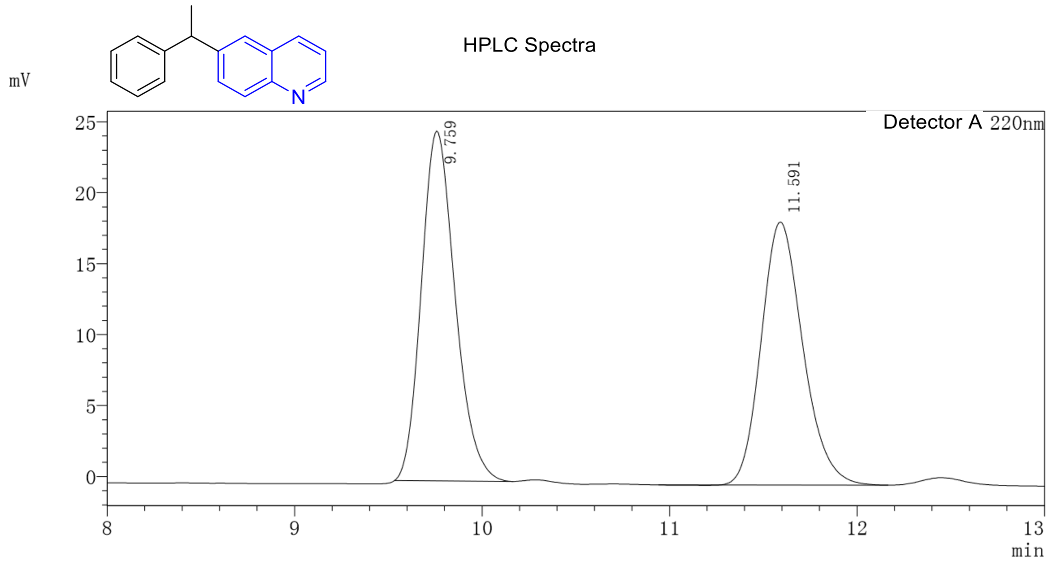

Area Percent Report

Detector A 220nm

Number	Remaining Time	Area	Height	Note	Area %
1	9.759	309302	24660	M	51.844
2	11.591	287305	18550	M	48.156
Total		596607	43209		100.000

HPLC Condition : OJ-H, n-hexane:iPrOH = 90/10, 1.0 ml/min, 220 nm

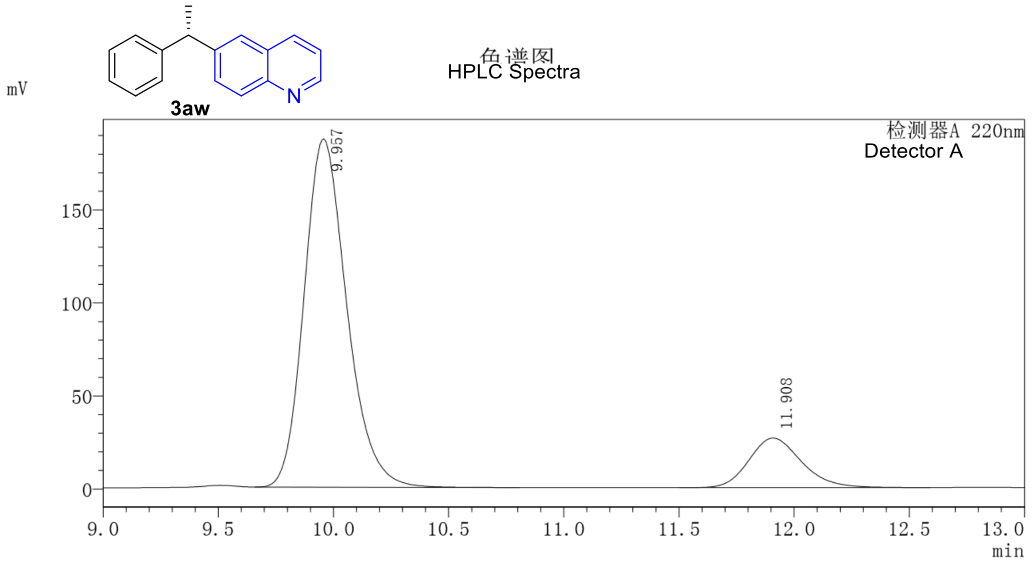

峰表

Area Percent Report

检测器A 220nm

Detector Number	保留时间 Remaining Time	面积 Area	高度 Height	标记 Note	面积% Area %
2	11.908	424360	26631		14.918
总计 Total		2844591	213845		100.000

Supplementary Figure 131. HPLC spectra for **3aw**

描述 : OD-H, n-hexane:iPrOH = 99.9/0.1, 0.5 ml/min, 220 nm
HPLC Condition

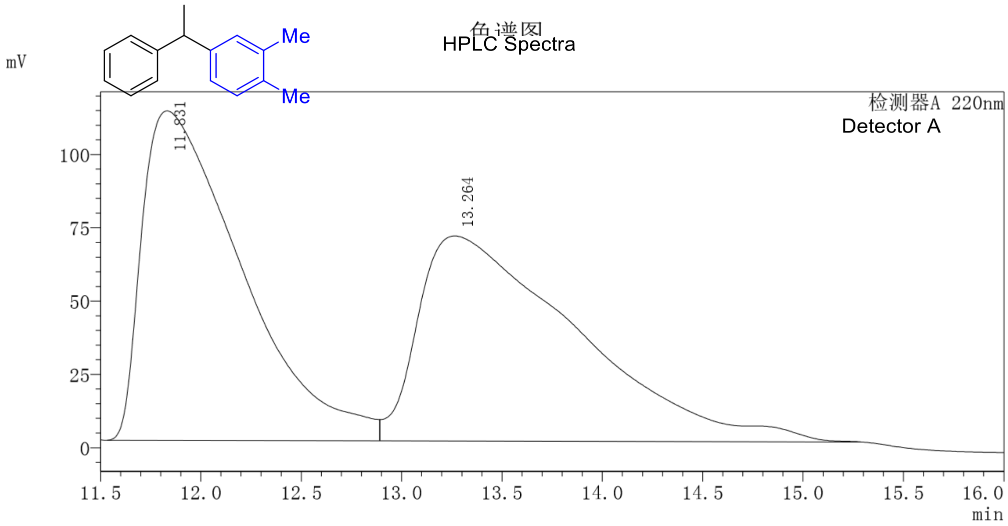

峰表

Area Percent Report

检测器A 220nm

Detector Number	保留时间 Remaining Time	面积 Area	高度 Height	标记 Note	面积% Area %
2	13.264	3871252	69949	V M	49.899
总计 Total		7758222	182344		100.000

描述 : OD-H, n-hexane:iPrOH = 99.9/0.1, 0.5 ml/min, 220 nm

HPLC Condition

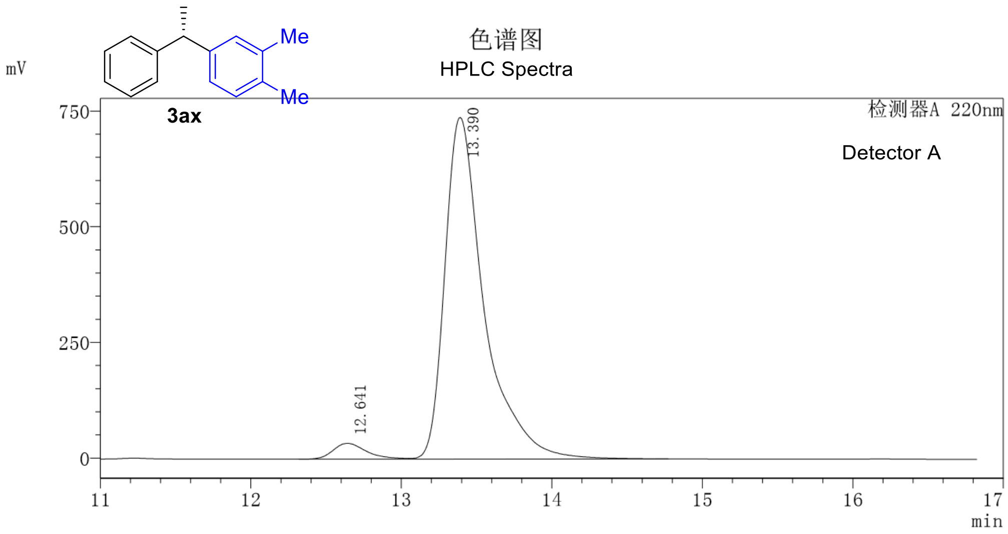

峰表

Area Percent Report

检测器A 220nm

峰号 Detector Number	保留时间 Remaining Time	面积 Area	高度 Height	标记 Note	面积% Area %
[illegible]	12.641	525182	34256		3.791
[illegible]	[illegible]	[illegible]	[illegible]	[illegible]	[illegible]
总计 Total		13853031	772477		100.000

Supplementary Figure 132. HPLC spectra for **3ax**

HPLC Condition : OJ-H, n-hexane:iPrOH = 95/5, 0.8 ml/min, 220 nm

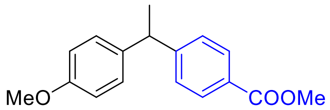

HPLC Spectra

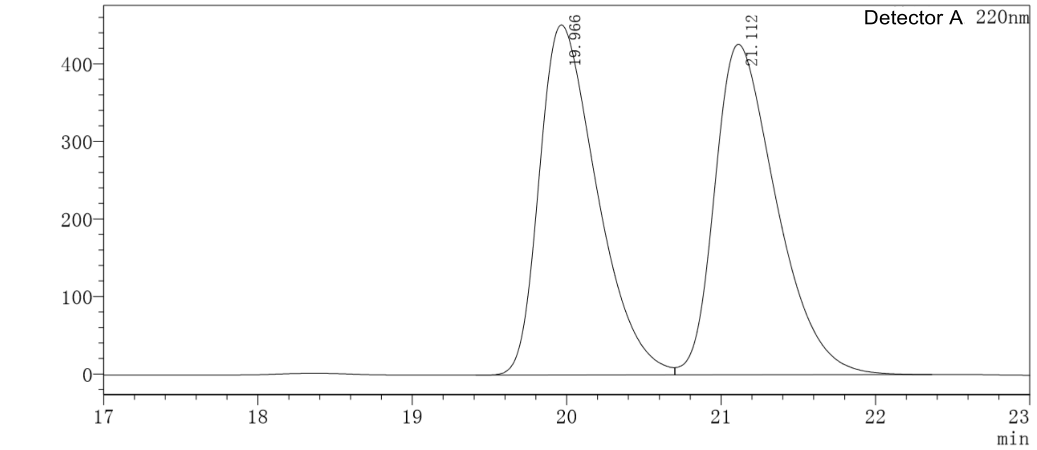

Area Percent Report

Detector A 220nm

Number	Remaining Time	Area	Height	Note	Area %
1	19.966	11748537	451538		49.781
2	21.112	11852045	426085	V	50.219
Total		23600582	877623		100.000

HPLC Condition : OJ-H, n-hexane:iPrOH = 95/5, 0.80 ml/min, 220 nm

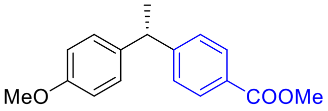

3ba

HPLC Spectra

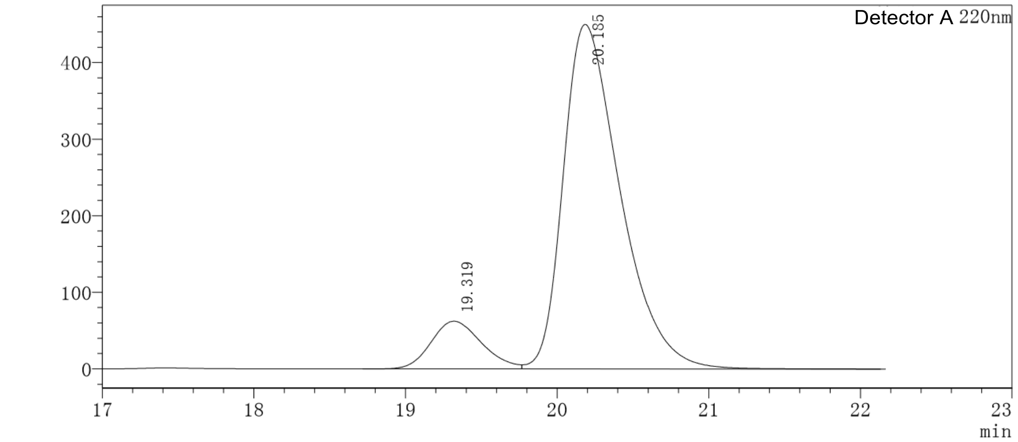

Area Percent Report

Detector A 220nm

Number	Remaining Time	Area	Height	Note	Area %
1	19.319	1450641	62353		10.981
2	20.185	11759803	449966	V	89.019
Total		13210443	512319		100.000

Supplementary Figure 133. HPLC spectra for **3ba**

HPLC Condition : OJ-H, n-hexane/iPrOH =90/10, 1.0 ml/min, 220 nm

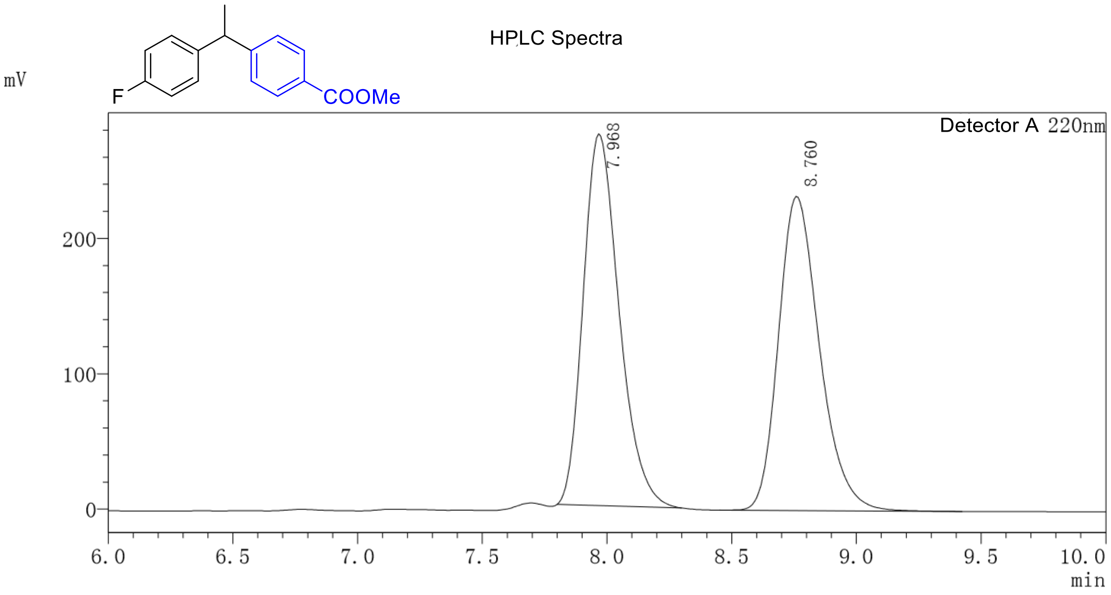

Area Percent Report

Detector A 220nm

Number	Remaining Time	Area	Height	Note	Area %
1	7.968	2747594	274722	M	50.887
2	8.760	2651812	232212		49.113
Total		5399406	506934		100.000

HPLC Condition : OJ-H , n-hexane/iPrOH = 90/10, 1.0 mL/min, 220 nm

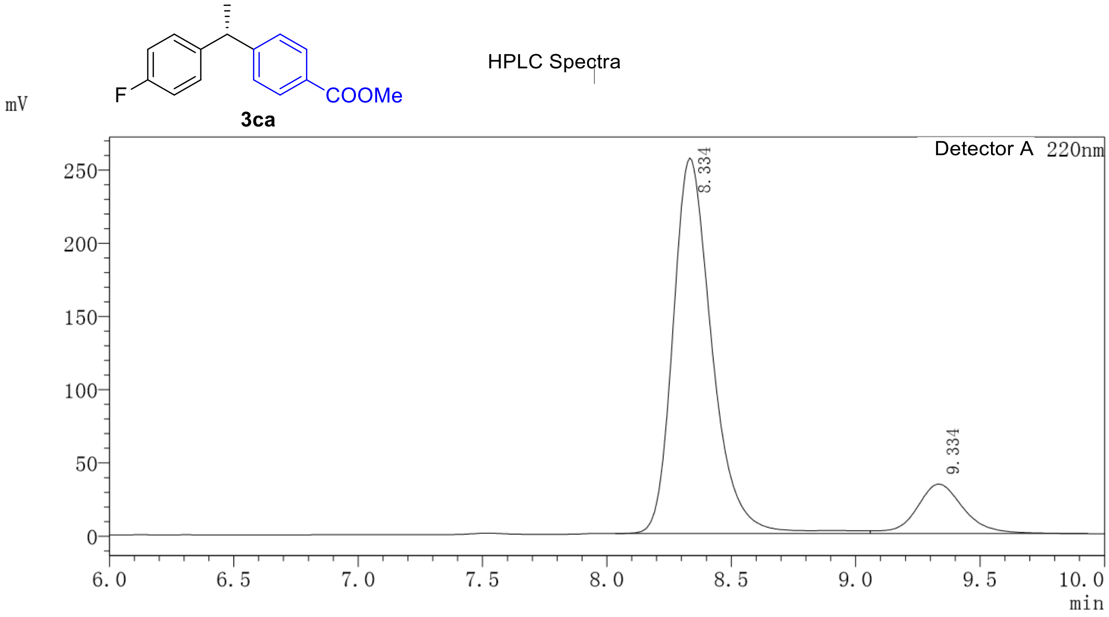

Area Percent Report

Detector A 220nm

Number	Remaining Time	Area	Height	Note	Area %
1	8.334	2795839	256383	S	86.229
2	9.334	446507	33827	V M	13.771
Total		3242346	290210		100.000

Supplementary Figure 134. HPLC spectra for **3ca**

HPLC Condition : AS-H*2, n-hexane/iPrOH = 98/2, 1.0 ml/min, 220 nm

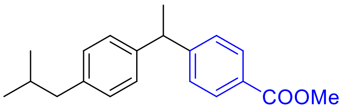

HPLC Spectra

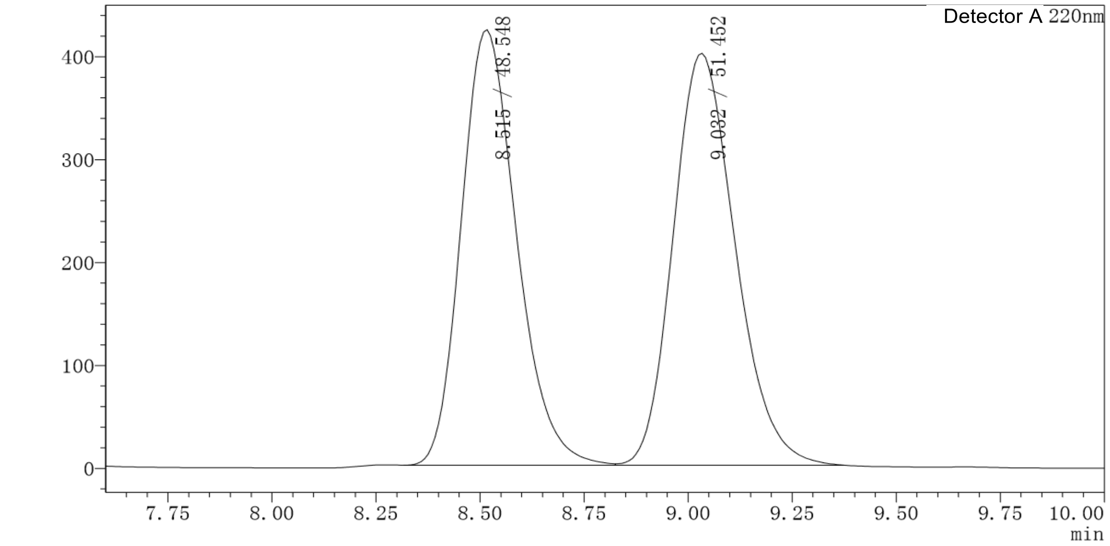

Area Percent Report

Detector A 220nm

Number	Remaining Time	Area	Height	Note	Area %
1	8.515	3911826	423138		48.548
2	9.032	4145857	400239	V M	51.452
Total		8057683	823376		100.000

HPLC Condition : AS-H*2, n-hexane/iPrOH = 98/2, 1.0 ml/min, 220 nm

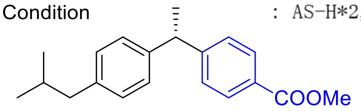

3da

HPLC Spectra

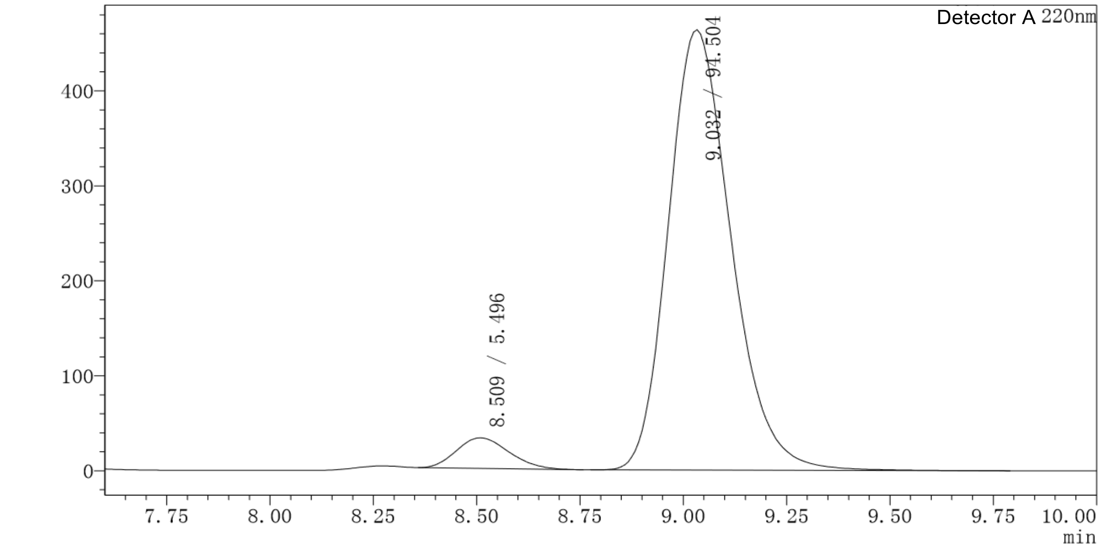

Area Percent Report

Detector A 220nm

Number	Remaining Time	Area	Height	Note	Area %
1	8.509	284795	32338		5.496
2	9.032	4896690	463646		94.504
Total		5181485	495984		100.000

Supplementary Figure 135. HPLC spectra for **3da**

HPLC Condition : 2*OD-H , n-hexane/iPrOH = 90/10, 1.0 mL/min, 220 nm

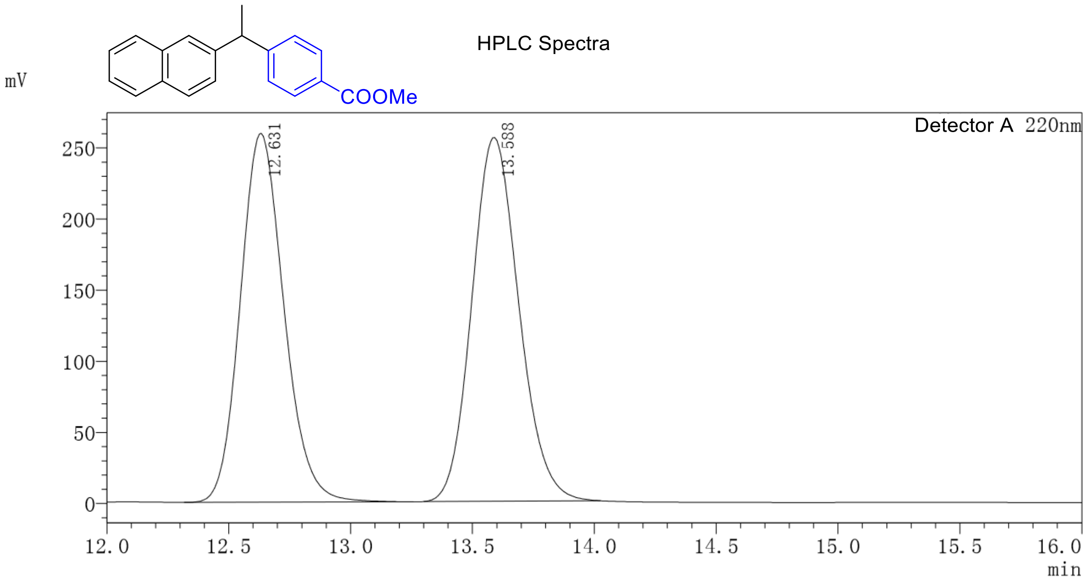

Area Percent Report

Detector A 220nm

Number	Remaining Time	Area	Height	Note	Area %
1	12.631	3227755	259328		48.510
2	13.588	3426010	255590	M	51.490
Total		6653765	514917		100.000

HPLC Condition : 2*OD-H , n-hexane/iPrOH = 90/10, 1.0 mL/min, 220 nm

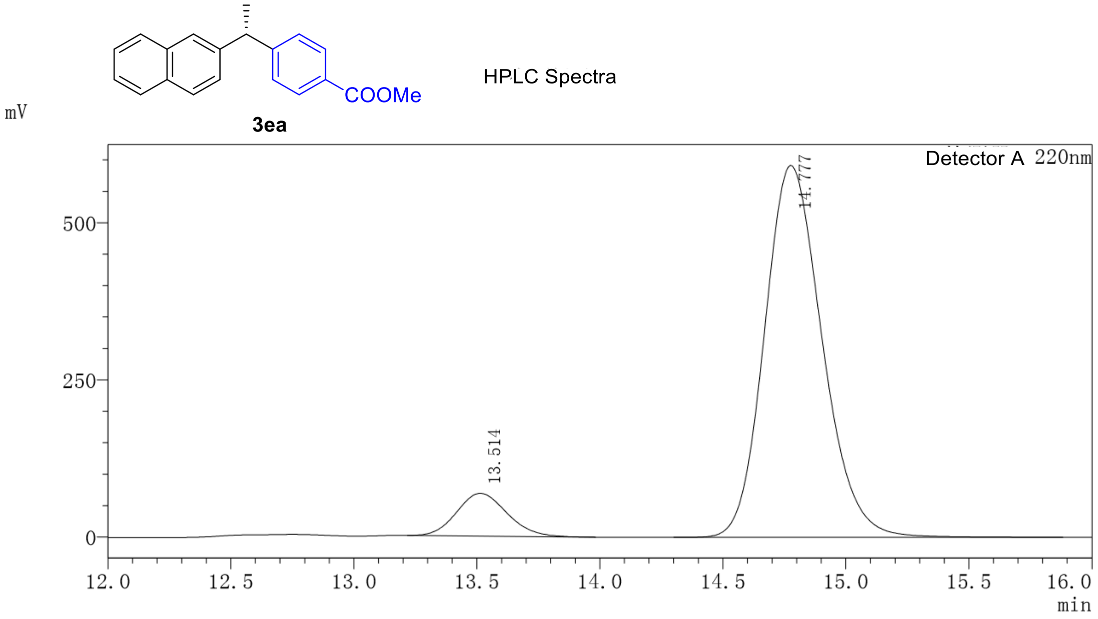

Area Percent Report

Detector A 220nm

Number	Remaining Time	Area	Height	Note	Area %
1	13.514	968182	67970		9.138
2	14.777	9626640	591724		90.862
Total		10594822	659694		100.000

Supplementary Figure 136. HPLC spectra for **3ea**

HPLC Condition : OD-H, n-hexane:iPrOH = 98/2, 1.0ml/min, 220 nm

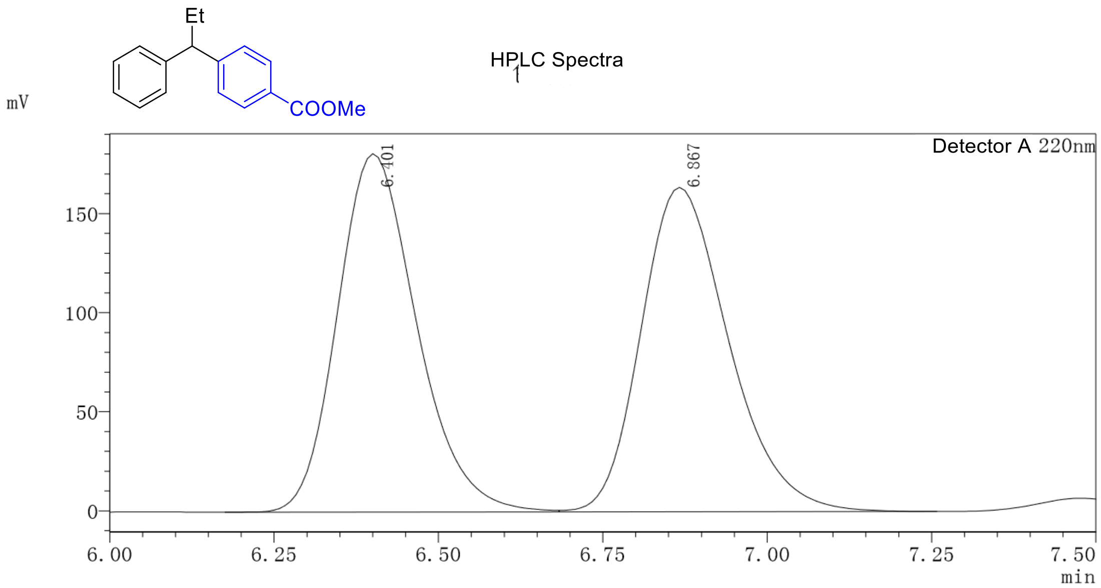

Area Percent Report

Detector A 220nm

Number	Remaining Time	Area	Height	Note	Area %
1	6.401	1515838	180716		50.002
2	6.867	1515738	163721	V	49.998
Total		3031576	344437		100.000

HPLC Condition : OD-H , n-hexane/iPrOH = 98/2, 1.0 mL/min, 220 nm

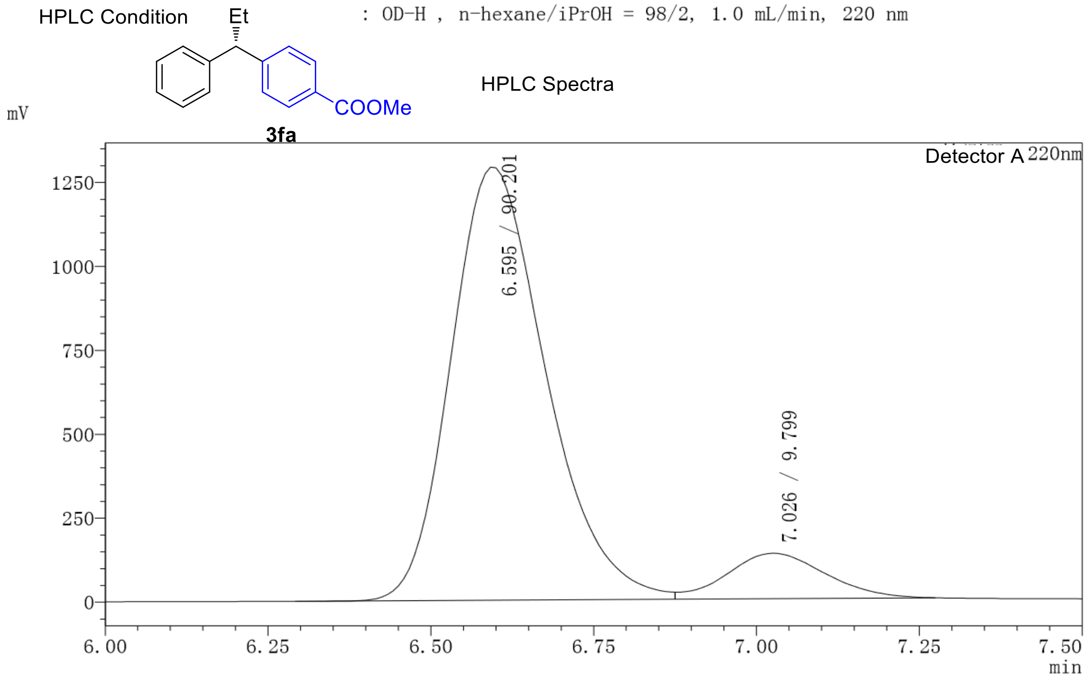

Area Percent Report

Detector A 220nm

Number	Remaining Time	Area	Height	Note	Area %
1	6.595	13071736	1289789		90.201
2	7.026	1419998	135533	V M	9.799
Total		14491735	1425322		100.000

Supplementary Figure 137. HPLC spectra for **3fa**

HPLC Condition : OD-H*2, n-hexane/iPrOH =100/0, 0.5 ml/min, 220 nm

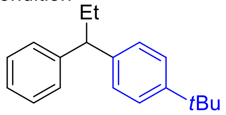

HPLC Spectra

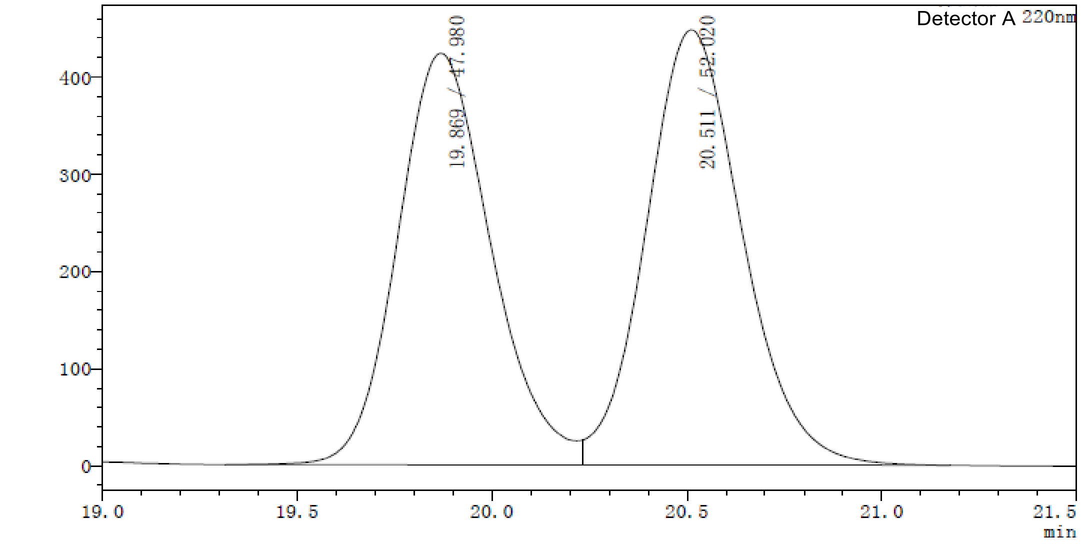

Area Percent Report

Detector A 220nm

Number	Remaining Time	Area	Height	Note	Area %
1	19.869	7088639	423664	M	47.980
2	20.511	7685646	447886	V M	52.020
Total		14774285	871551		100.000

HPLC Condition : OD-H*2, n-hexane/iPrOH =100/0, 0.5 ml/min, 220 nm

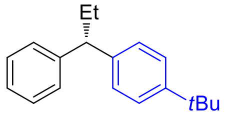

3fd

HPLC Spectra

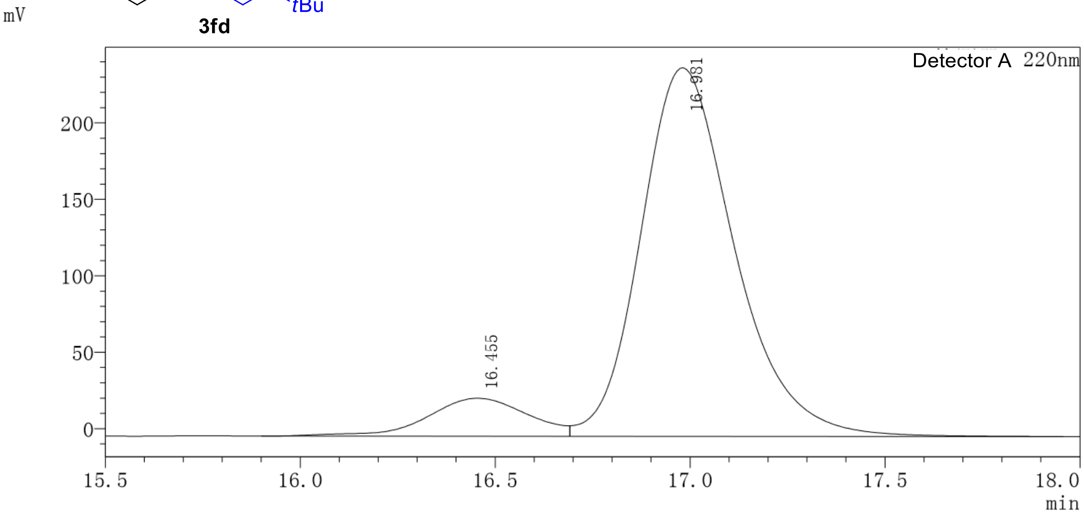

Area Percent Report

Detector A 220nm

Number	Remaining Time	Area	Height	Note	Area %
1	16.455	447172	24853		9.995
2	16.981	4026874	241108	V	90.005
Total		4474047	265961		100.000

Supplementary Figure 138. HPLC spectra for **3fd**

HPLC Condition ： OD-H*2， n-Hexane/iPrOH = 95/5， 1.0 mL/min， 220 nm

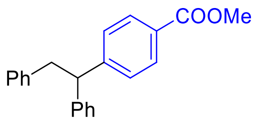

HPLC Spectra

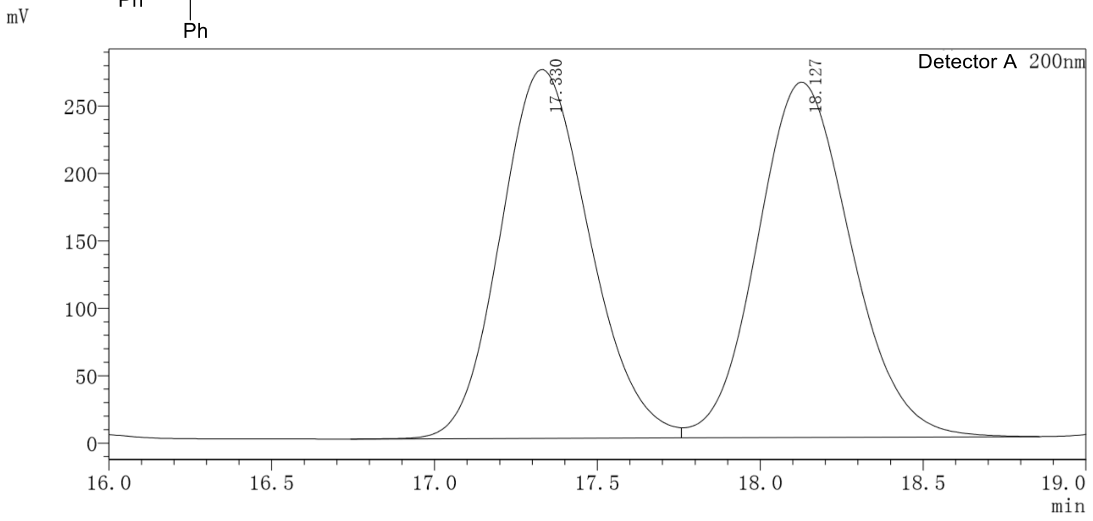

Area Percent Report

Detector A 200nm

Number	Remaining Time	Area	Height	Note	Area %
1	17.330	5217465	273585		49.665
2	18.127	5287789	263575	V	50.335
Total		10505254	537160		100.000

HPLC Condition ： 2*OD-H ， n-hexane/iPrOH = 95/5， 1.0 mL/min， 220 nm

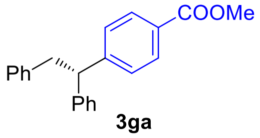

3ga

HPLC Spectra

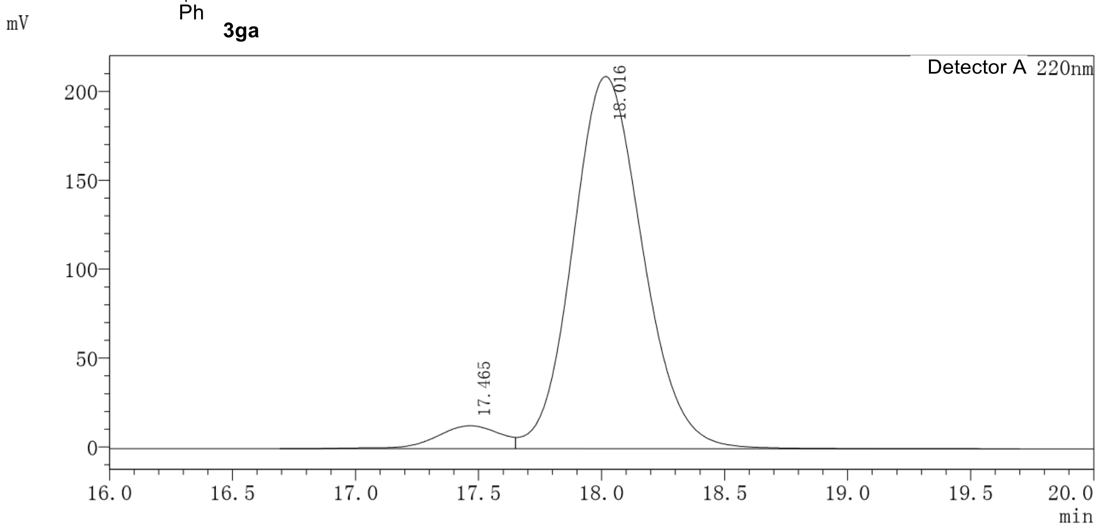

Area Percent Report

Detector A 220nm

Number	Remaining Time	Area	Height	Note	Area %
1	17.465	237153	12947		5.417
2	18.016	4141098	209335	V	94.583
Total		4378251	222282		100.000

Supplementary Figure 139. HPLC spectra for **3ga**

HPLC Condition : OD-H, n-hexane:iPrOH = 90/10, 1.0 ml/min, 220 nm

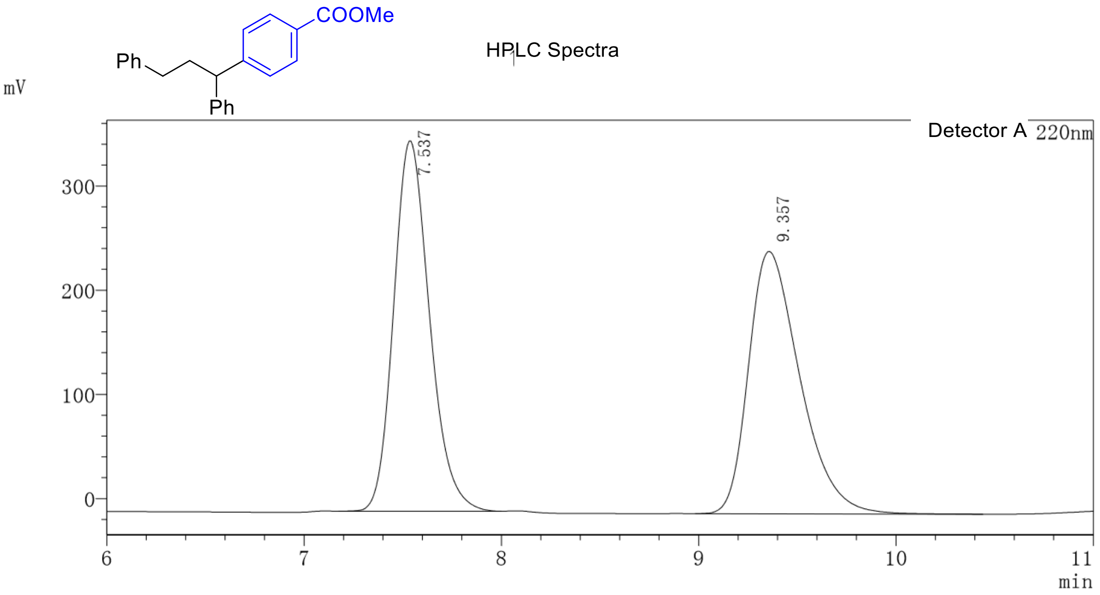

Area Percent Report

Detector A 220nm

Number	Remaining Time	Area	Height	Note	Area %
1	7.537	4505176	355295		49.719
2	9.357	4556134	251817		50.281
Total		9061311	607111		100.000

HPLC Condition : OD-H , n-hexane/iPrOH = 90/10, 1.0 mL/min, 220 nm

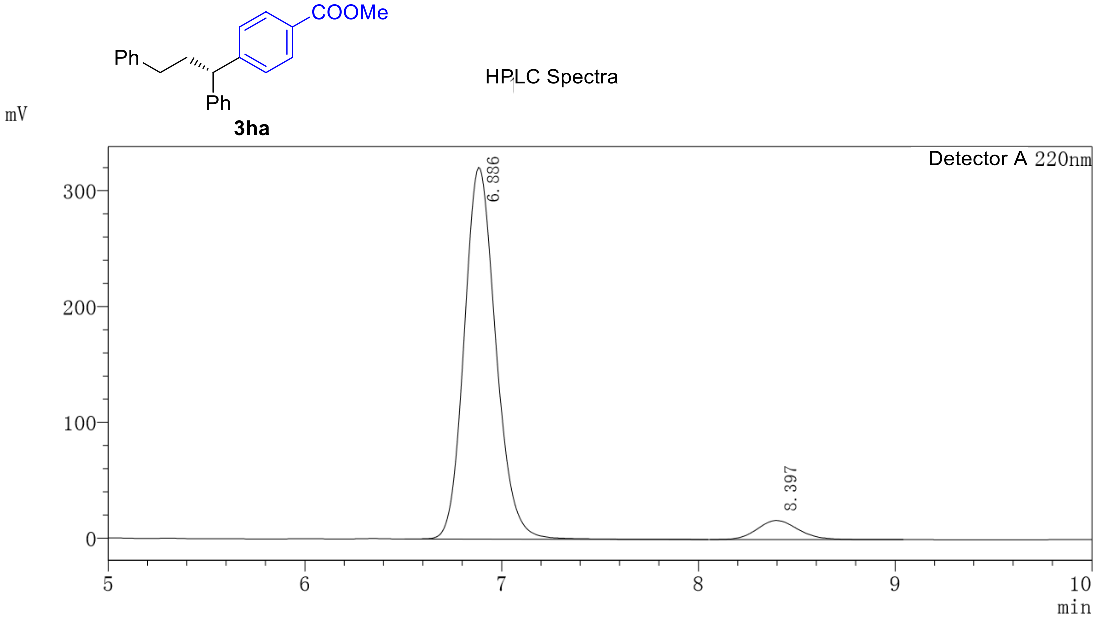

Area Percent Report

Detector A 220nm

Number	Remaining Time	Area	Height	Note	Area %
1	6.886	3584360	320954		93.770
2	8.397	238137	16449		6.230
Total		3822498	337402		100.000

Supplementary Figure 140. HPLC spectra for **3ha**

HPLC Condition : OD-H, n-hexane:iPrOH = 90/10, 1.0 ml/min, 220 nm

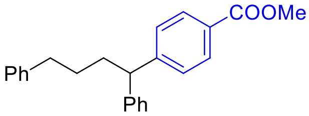

HPLC Spectra

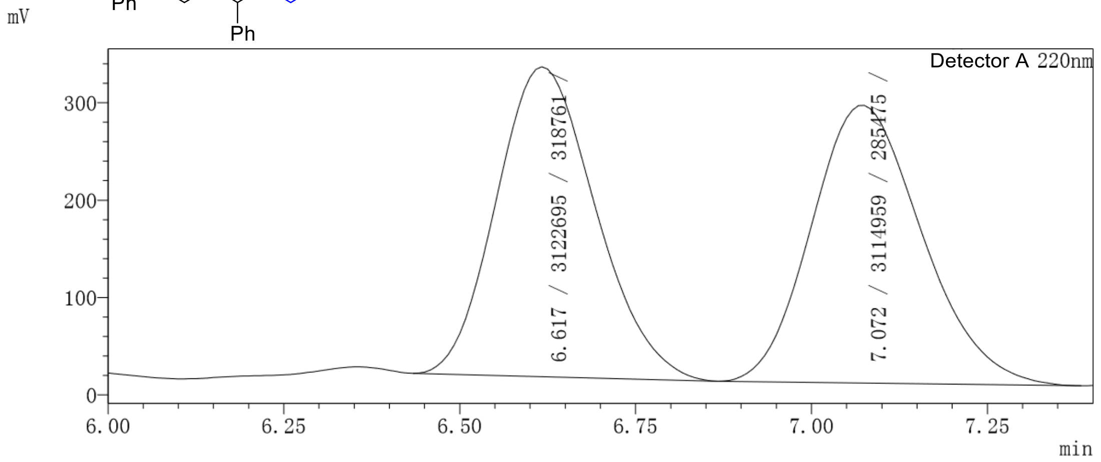

Area Percent Report

Detector A 220nm

Number	Remaining Time	Area	Height	Note	Area %
1	6.617	3122695	318761		50.062
2	7.072	3114959	285475		49.938
Total		6237654	604236		100.000

HPLC Condition : OD-H , n-hexane/iPrOH = 90/10, 1.0 mL/min, 220 nm

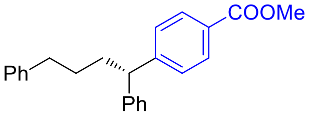

3ia

HPLC Spectra

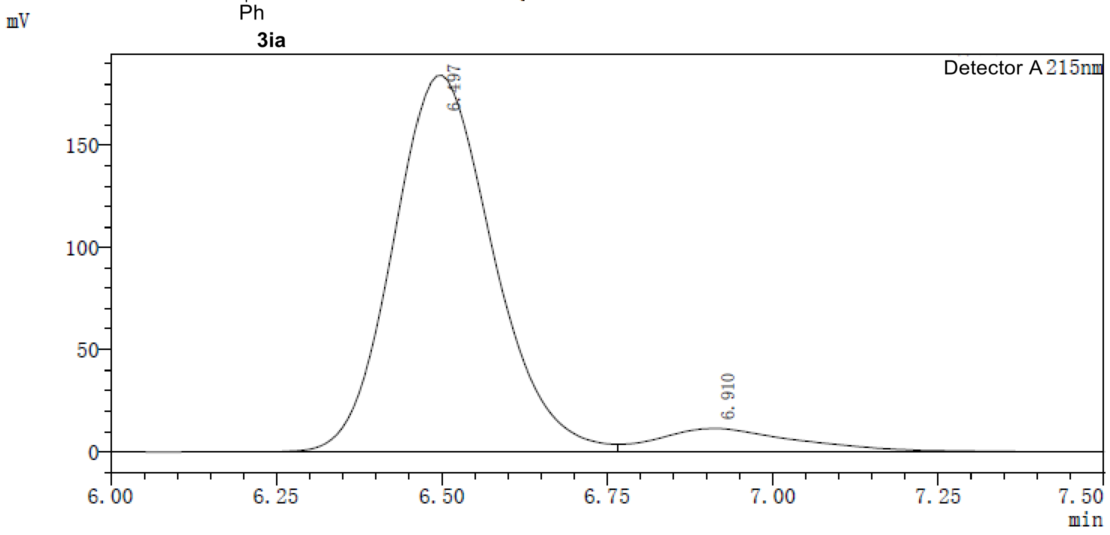

Area Percent Report

Detector A 215nm

Number	Remaining Time	Area	Height	Note	Area %
1	6.497	1922291	183887		92.249
2	6.910	161507	11348	V	7.751
Total		2083797	195234		100.000

Supplementary Figure 141. HPLC spectra for **3ia**

HPLC Condition : OD-H, n-hexane:iPrOH = 90/10, 1.0 ml/min, 220 nm

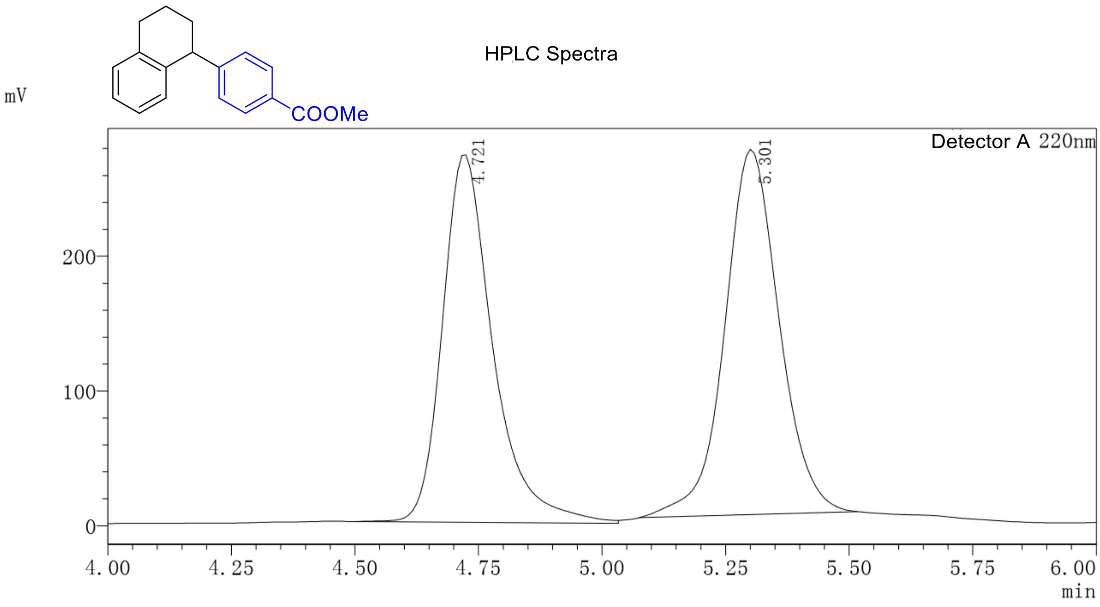

Area Percent Report

Detector A 220nm

Number	Remaining Time	Area	Height	Note	Area %
1	4.721	1940763	272270	M	48.247
2	5.301	2081784	271119	M	51.753
Total		4022547	543389		100.000

HPLC Condition : OD-H, n-hexane/iPrOH = 90/10, 1.0 mL/min, 220 nm

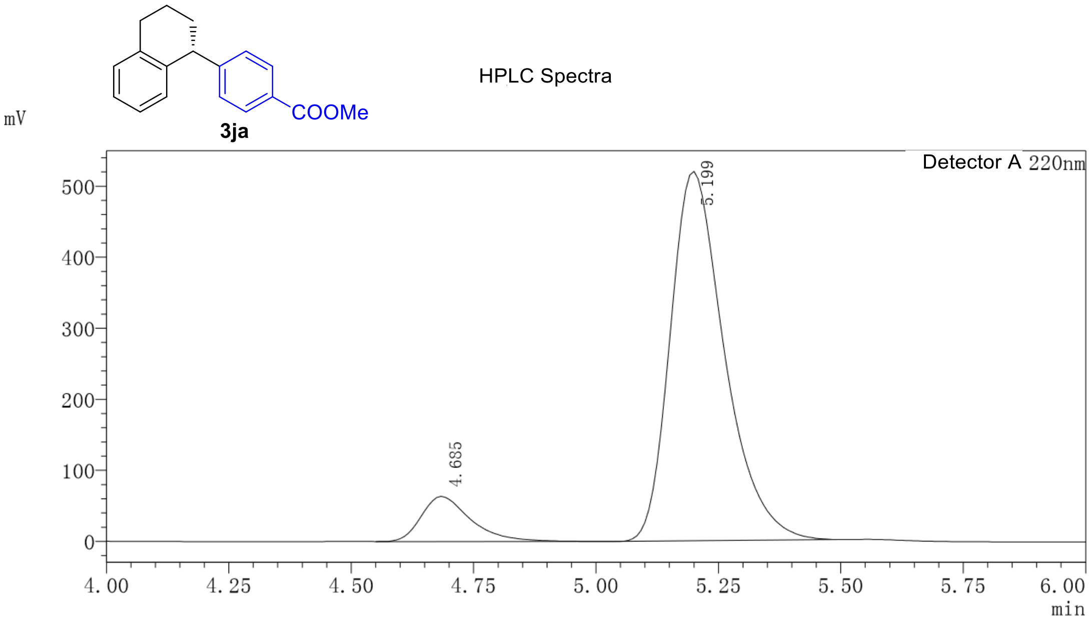

Area Percent Report

Detector A 220nm

Number	Remaining Time	Area	Height	Note	Area %
1	4.685	447475	63546		10.069
2	5.199	3996765	520017		89.931
Total		4444241	583564		100.000

Supplementary Figure 142. HPLC spectra for **3ja**

HPLC Condition : 2*OJ-H , n-hexane/iPrOH = 99.5/0.5, 1.0 mL/min, 220 nm

HPLC Spectra

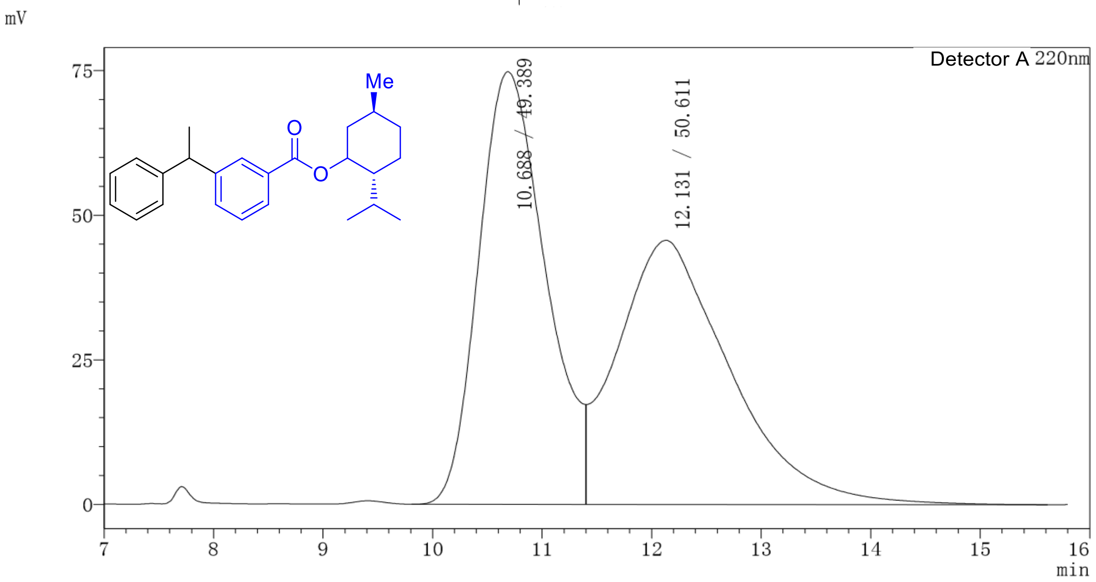

Area Percent Report

Detector A 220nm

Number	Remaining Time	Area	Height	Note	Area %
1	10.688	3182628	74793		49.389
2	12.131	3261423	45652	V	50.611
Total		6444051	120445		100.000

HPLC Condition : OJ-H*2, n-hexane:iPrOH = 99.5/0.5, 1.0 ml/min, 220 nm

HPLC Spectra

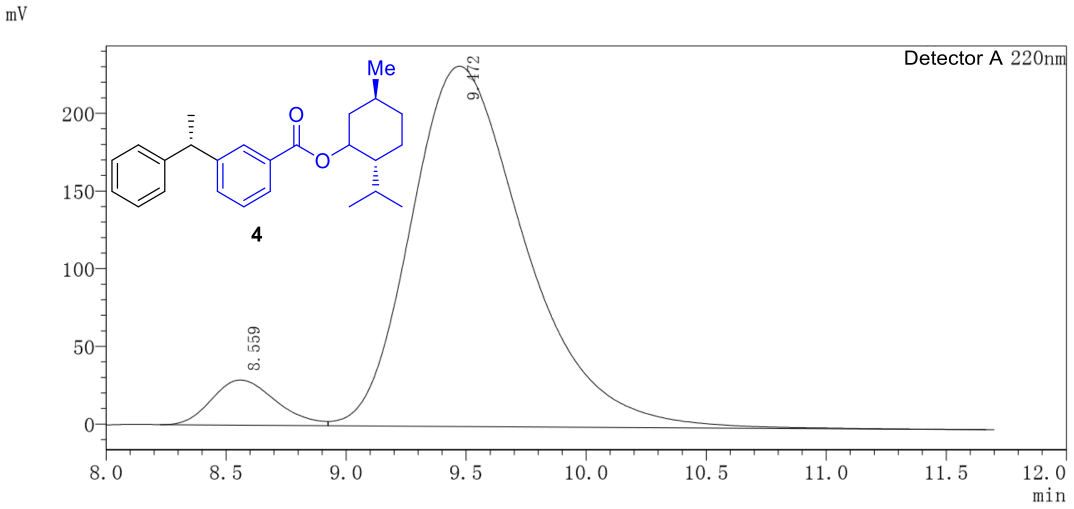

Area Percent Report

Detector A 220nm

Number	Remaining Time	Area	Height	Note	Area %
1	8.559	539920	29033		6.562
2	9.472	7688641	231842	V	93.438
Total		8228561	260876		100.000

Supplementary Figure 143. HPLC spectra for **4**

HPLC Condition ： AS-H*2, n-hexane/iPrOH = 98/2, 1.0 ml/min, 220 nm

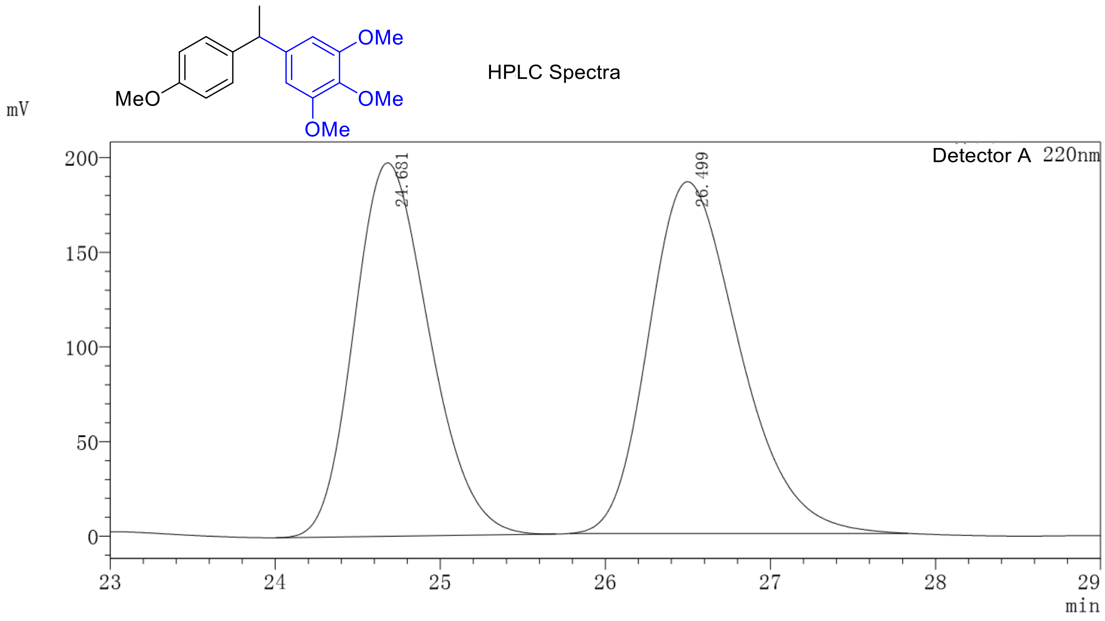

Area Percent Report

Detector A 220nm

Number	Remaining Time	Area	Height	Note	Area %
1	24.681	6254862	197217	M	46.817
2	26.499	7105416	185776	M	53.183
Total		13360278	382994		100.000

HPLC Condition ： AS-H*2, n-hexane/iPrOH = 98/2, 1.0 ml/min, 220 nm

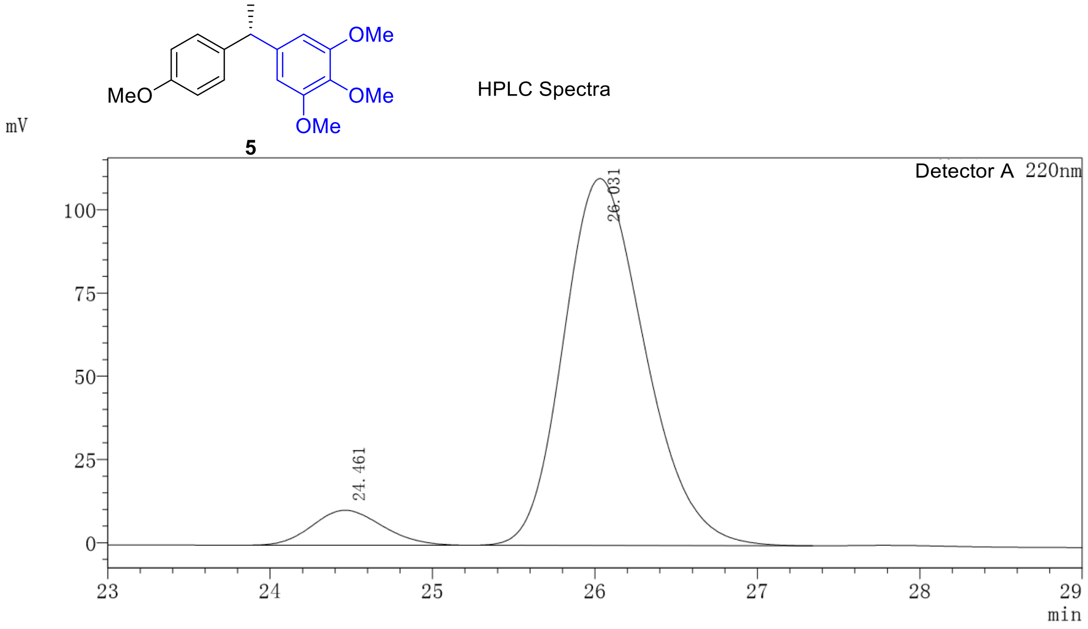

Area Percent Report

Detector A 220nm

Number	Remaining Time	Area	Height	Note	Area %
1	24.461	316447	10513		7.588
2	26.031	3854097	110238		92.412
Total		4170543	120751		100.000

Supplementary Figure 144. HPLC spectra for **5**

Supplementary Tables

Supplementary Table 1. Optimations on photocatalyst & nickel catalysts

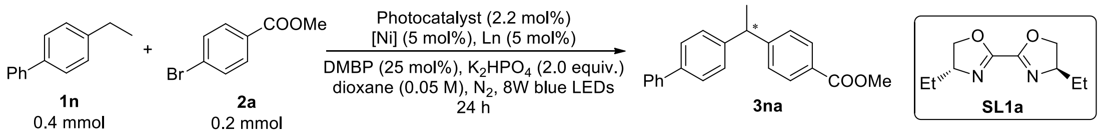

Entry	[Ni]	Ln	Photocatalyst	Yield	er
1[a]	$Ni(NO_3)_2$•$6H_2O$	dtbbpy	P1	0	-
2[b]	$Ni(NO_3)_2$•$6H_2O$	dtbbpy	P1	2	-
3[c]	$Ni(NO_3)_2$•$6H_2O$	dtbbpy	P2	8	-
4[c]	$Ni(NO_3)_2$•$6H_2O$	dtbbpy	P3	14	-
6[c]	$Ni(acac)_2$	dtbbpy	P3	0	-
7[c]	$Ni(OAc)_2$•$4H_2O$	dtbbpy	P3	0	-
8[c]	$NiBr_2$•H_2O	dtbbpy	P3	5	-
9[c]	$NiCl_2$•DME	dtbbpy	P3	14	-
10[d]	$NiCl_2$•DME	dtbbpy	P3	20	-
11[d]	$NiCl_2$•DME	**SL1a**	P3	30	16.5:83.5
12[d]	$Ni(NO_3)_2$•$6H_2O$	**SL1a**	P3	0	-
13[d]	$NiBr_2$•DME	**SL1a**	P3	36	20.5:79.5

[a]The reaction was carried out at room temperature (25 ºC) under the irradiation of 35 W CFL. [b]The reaction was carried out at 50 ºC under the irradiation of 35 W CFL. [c]The reaction was carried out at 50 ºC under the irradiation of 8 W blue LEDs. [d]The reaction was carried out using 20 mol% of $NiCl_2$•DME and 4,4'-di-*tert*-butyl-2,2'-bipyridine (dtbbpy) at 50 ºC under the irradiation of 8 W blue LEDs.

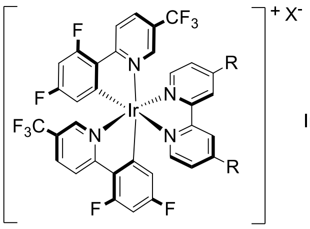

P1: R = H, X = PF_6
$Ir(dFCF_3ppy)_2(bpy)(PF_6)$
P2: R = *t*Bu, X = PF_6
$Ir(dFCF_3ppy)_2(dtbbpy)(PF_6)$
P3: R = *t*Bu, X = Cl
$Ir(dFCF_3ppy)_2(dtbbpy)Cl$

Supplementary Table 2. Optimations on chiral ligands

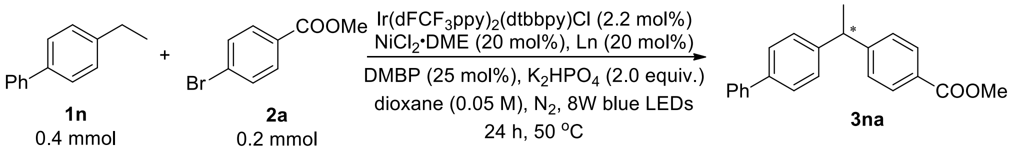

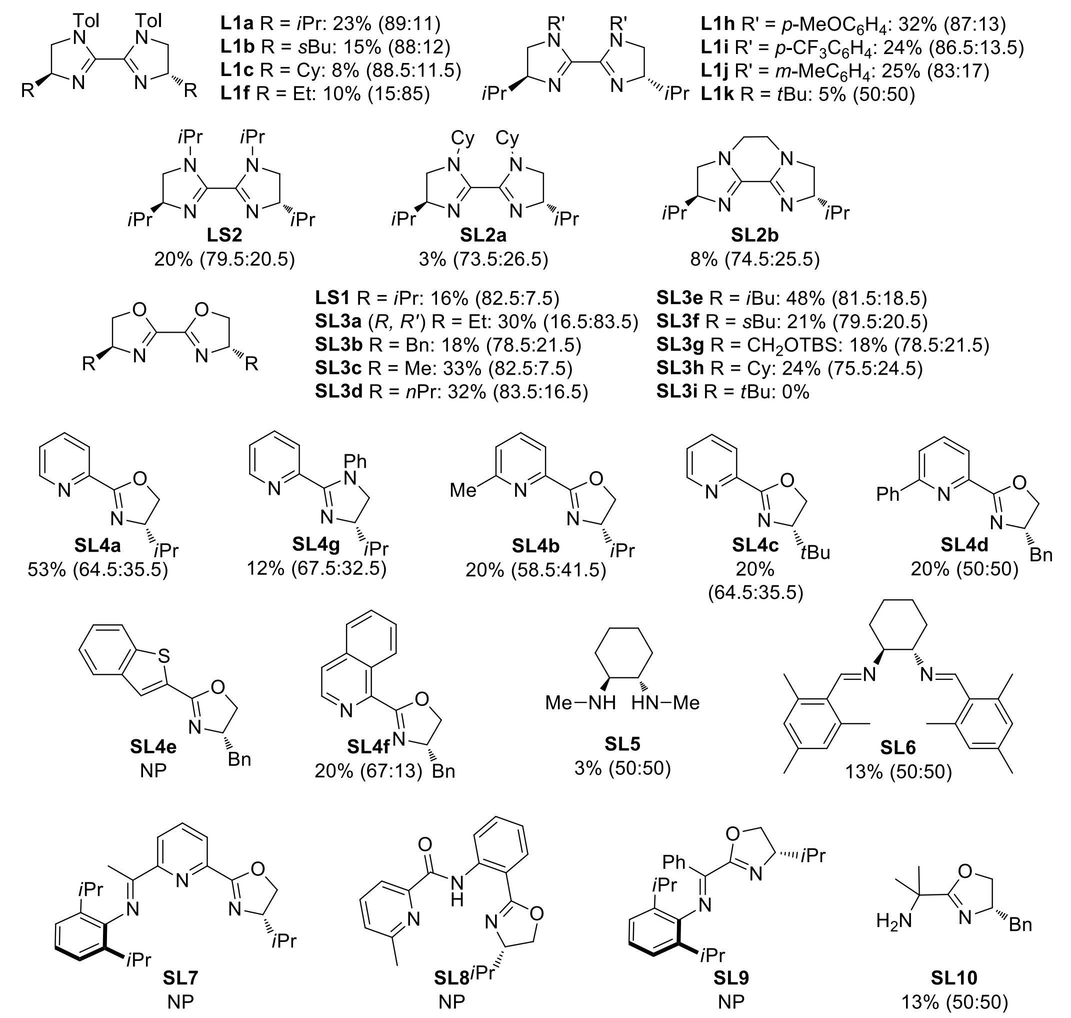

Supplementary Methods

THF, toluene, and 1,4-dioxane were distilled from sodium benzophenone ketyl prior to use. CH_3CN and CH_2Cl_2 were distilled from CaH_2 prior to use. 8 W blue LEDs were used as light source. $NiCl_2$·DME was purchased from Strem or prepared according to the previously reported method[1]. The other commercially available chemicals were used as received. NMR spectra were recorded on a Bruker-400 instrument or a Wuhan Zhongke-Niujin-400 instrument. ^1H NMR chemical shifts were referenced to tetramethylsilane signal (0 ppm), ^{13}C NMR chemical shifts were referenced to the solvent resonance (77.00 ppm, $CDCl_3$). The following abbreviations (or combinations) were used to explain multiplicities: s = singlet, d = doublet, t = triplet, m = multiplet, br = broad. IR spectra were recorded on a Perkin-Elmer Spectrum One FTIR spectrometer with diamond ATR accessory. Melting points were obtained using a WRR melting point apparatus (Laboratory Devices, Shanghai Precision & Scientific Instrument Co., Ltd.). High-resolution mass spectra (HRMS) were recorded on EI-TOF (electron ionization-time of flight) or Waters XEVOG2-S ESI-TOF mass spectrometer. Optical rotations were measured on a Perkin-Elmer 241 polarimeter equipped with a Na-lamp. HPLC analyses were performed on a Shimadzu SPD-20A. The enantiometric ratio (*er*) was expressed nearest 0.1. GC analyses were performed on a Shimadzu GC-2014.

Preparation of Photocatalyst

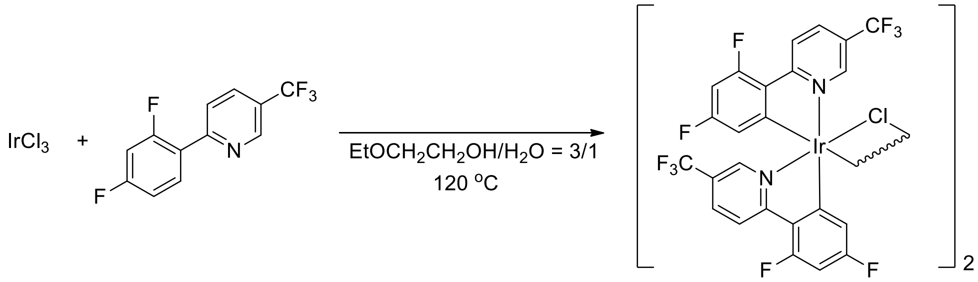

The iridium dimer was synthesized according to the previously reported method.[2] To an overdried 100 mL schlenk flask equipped with a reflux condenser was added $IrCl_3$ (1.0 equiv., 4.0 mmol, 1.4191 g) and 2-(2,4-difluorophenyl)-5-(trifluoromethyl)pyridine (2.2 equiv., 8.8 mmol, 2.2870 g). 64 mL of degassed solvent ($EtOCH_2CH_2OH/H_2O$ = 3/1) was added and the reaction was stirred at 120 °C under N_2 atomsphere for 20 h. Upon completion, the reaction was cooled to room temperature and filtered. The filtercake was washed with water and petroleum ether and dried under vacuum. Iridium dimer was obtained in 63% (1.8917 g, 1.3 mmol) yield as a yellow powder.

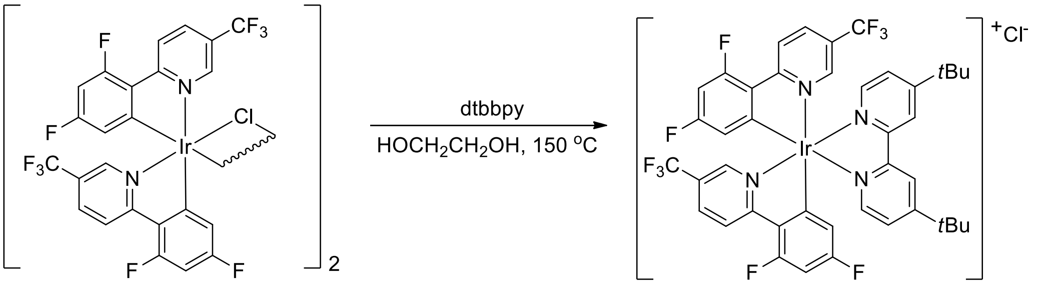

The iridium dimer was synthesized according to the previously reported method. [3] To an overdried 100 mL schlenk flask equipped with a reflux condenser was added iridium dimer (1.0 equiv., 0.5 mmol, 0.7451 g), 4,4'-di-*tert*-butyl-2,2'-bipyridine (2.4 equiv., 1.2 mmol, 0.3201 g) and 30 mL of ethylene glycol. The reaction was stirred at 150 °C for 24 h and cooled to room temperature. Water was added and the

aqueous layer was extracted three times with chloroform. The combined organic layers were dried with Na_2SO_4, filtered, and concentrated *in vacuo*. Ir(dFCF_3ppy)$_2$(dtbbpy)Cl was obtained by recrystallization from DCM/Et_2O in 91% (0.9199 g, 0.91 mmol) yield as a yellow solid. ^{1}H NMR (400 MHz, Acetone-d6) δ 9.74 (d, *J* = 1.2 Hz, 2H), 8.66 (d, *J* = 8.8 Hz, 2H), 8.46 (d, *J* = 8.8 Hz, 2H), 8.19 (s, 2H), 8.16 (d, *J* = 6.0 Hz, 2H), 7.84 (s, 2H), 7.79 (dd, *J* = 6.0, 1.6 Hz, 2H), 6.95 – 6.79 (m, 2H), 5.99 (dd, *J* = 8.4, 2.0 Hz, 2H), 1.53 (s, 18H). ^{19}F NMR (376 MHz, Acetone-d6) δ -63.63 (d, *J* = 12.0 Hz), -104.90 (d, *J* = 11.6 Hz), -108.11 (s). All spectra were in complete agreement with the previously reported values.[2]

Procedure A for the preparations of BiIM Ligands:

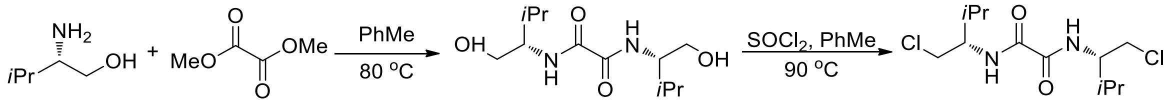

The biimidazole ligands were synthesized according to the previously reported method.[4,5] (*S*)-2-amino-3-isopropane-1-ol (2.0 equiv., 10 mmol, 1.0645 g) and dimethyloxalate (1.0 equiv., 10 mmol, 0.5968 g) were dissolved in toluene (100 mL) and heated to 80 °C. The reaction was allowed to stir overnight with the diamide precipitating out of solution as a white solid. Reaction was cooled to room temperature and concentrated *in vacuo* to. The crude diol was dissolved in toluene (20 mL) and heated to 70 °C whereupon thionyl chloride (2.0 equiv., 10 mmol, 0.8 mL) was added. Reaction was stirred at 70 °C for 30 minutes then heated to 90 °C for 2 h. The reaction was cooled to room temperature and concentrated under reduced pressure to afford the dichloro-intermediate which was used without further purification.

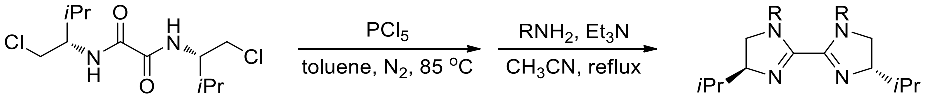

To an overdried three-necked flask was added the crude dichloro-intermediate (1.0 equiv., 1.35 mmol, 0.4000 g) and phosphorus pentachloride (2.4 equiv., 3.23 mmol, 0.6730 g) in 10 mL of toluene under N_2 atmosphere. The reaction was allowed to stir at 85 °C for 4.5 h before it was cooled to room temperature. Toluene was evaporate under reduced pressure and Et_3N (11.2 equiv., 15.2 mmol, 1.12 mL) and isopropylamine (6.0 equiv., 8.1 mmol, 0.4767 g) in 20 mL of CH_3CN was added. The reaction was heated to reflux under N_2 until TLC showed complete consumption of the starting material. After cooling to room temperature, water was added and the aqueous layer was separated and extracted three times with DCM. The organic layer was dried with Na_2SO_4, filtered, and concentrated under reduced pressure. The residue was purified by flash column chromatography (PE:EA = 5:1) to give **LS2** (0.2284 g, 0.75 mmol) in 55% yield as a pale yellow oil. All the biimidazole ligands (**LS2**, **SL2a, SL2b**[5], **L1a** – **L1k**) in this research were synthesized by following Procedure A.

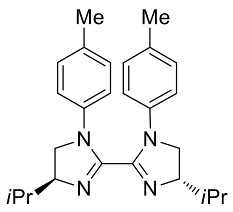

L1a

(*4S,4'S*)-4,4'-diisopropyl-1,1'-di-*p*-tolyl-4,4',5,5'-tetrahydro-*1H,1'H*-2,2'-biimidazole (L1a): brown solid. 75% yield. m.p. 135 – 137 °C (PE/EA). IR (neat, cm^{-1}): 2956, 2925, 2870, 1592, 1517. Optical Rotation: $[\alpha]_D^{20}$ = -267.4 (c 1.01, $CHCl_3$). ^{1}H NMR (400 MHz, $CDCl_3$) δ 6.86 (d, *J* = 8.0 Hz, 4H), 6.58 – 6.54 (m, 4H), 4.05 – 3.90 (m, 2H), 3.62 (t, *J* = 8.8 Hz, 2H), 3.49 (dd, *J* = 11.6, 9.2 Hz, 2H), 2.24 (s, 6H), 2.00 – 1.85 (m, 1H), 1.08 (d, *J* = 6.8 Hz, 6H), 0.99 (d, *J* = 6.8 Hz, 6H). ^{13}C NMR (101 MHz, $CDCl_3$) δ 153.7, 137.4, 132.7, 128.7, 119.6, 71.0, 53.8,

32.7, 20.7, 19.4, 18.8. HRMS (EI, m/z): calcd for $C_{26}H_{34}N_4$ (M^+) m/z: 402.2783, found: 402.2784.

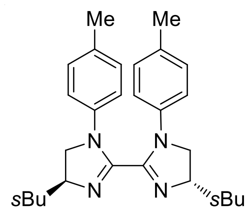

L1b

(*4S,4'S*)-4,4'-di-*sec*-butyl-1,1'-di-*p*-tolyl-4,4',5,5'-tetrahydro-*1H,1'H*-2,2'-biimidazole (L1b): light yellow solid. 30% yield. m.p. 114 – 117 °C (PE/EA). IR (neat, cm^{-1}): 2960, 2925, 2873, 1592, 1518. Optical Rotation: $[\alpha]_D^{20}$ = -250.4 (c 1.00, $CHCl_3$). ^1H NMR (400 MHz, $CDCl_3$) δ 6.86 (d, J = 8.0 Hz, 4H), 6.56 (d, J = 8.0 Hz, 4H), 4.14 – 4.06 (m, 2H), 3.59 (t, J = 9.6 Hz, 2H), 3.55 – 3.41 (m, 2H), 2.24 (s, 6H), 1.86 – 1.59 (m, 4H), 1.29 – 1.17 (m, 2H), 1.07 – 0.78 (m, 12H). ^{13}C NMR (101 MHz, $CDCl_3$) δ 153.6, 137.4, 132.7, 128.7, 119.6, 69.5, 53.3, 39.1, 26.2, 20.7, 14.9, 11.5. HRMS (EI, m/z): calcd for $C_{28}H_{38}N_4$ (M^+) m/z: 430.3096, found: 430.3098.

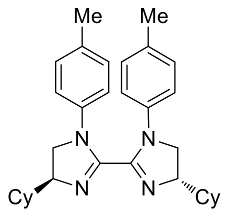

L1c

(*4S,4'S*)-4,4'-dicyclohexyl-1,1'-di-*p*-tolyl-4,4',5,5'-tetrahydro-*1H,1'H*-2,2'-biimidazole (L1c): white solid. 59% yield. m.p. 187 – 188 °C (PE/EA). IR (neat, cm^{-1}): 2921, 2852, 1590, 1516, 1448. Optical Rotation: $[\alpha]_D^{20}$ = -260.6 (c 1.01, $CHCl_3$). ^1H NMR (400 MHz, $CDCl_3$) δ 6.86 (d, J = 8.0 Hz, 4H), 6.56 (d, J = 8.0 Hz, 4H), 4.05 – 3.90 (m, 2H), 3.62 (t, J = 10.0 Hz, 2H), 3.50 (t, J = 10.4 Hz, 2H), 2.24 (s, 6H), 1.99 (d, J = 12.0 Hz, 2H), 1.82 – 1.45 (m, 12H), 1.40 – 0.95 (m, 10H). ^{13}C NMR (101 MHz, $CDCl_3$) δ 153.5, 137.4, 132.7, 128.7, 119.6, 70.0, 53.8, 42.5, 29.8, 29.2, 26.5, 26.22, 26.17, 20.7. HRMS (EI, m/z): calcd for $C_{32}H_{42}N_4$ (M^+) m/z: 482.3409, found: 482.3407.

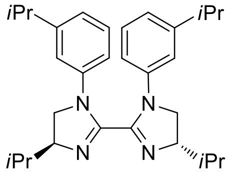

L1d

(*4S,4'S*)-4,4'-diisopropyl-1,1'-bis(3-isopropylphenyl)-4,4',5,5'-tetrahydro-*1H, 1'H*-2,2'-biimidazole (L1d): yellow solid. 52% yield. m.p. 85 – 87 °C (PE/EA). IR (neat, cm^{-1}): 2959, 2926, 1638, 1586, 1492, 1471. Optical Rotation: $[\alpha]_D^{20}$ = -229.8 (c 1.00, $CHCl_3$). ^1H NMR (400 MHz, $CDCl_3$) δ 6.96 (t, J = 7.6 Hz, 2H), 6.77 (d, J = 7.6 Hz, 2H), 6.53 – 6.32 (m, 4H), 4.05 – 3.90 (m, 2H), 3.67 (t, J = 9.6 Hz, 2H), 3.55 (dd, J = 11.2, 9.2 Hz, 2H), 2.80 – 2.60 (m, 2H), 2.04 – 1.86 (m, 2H), 1.21 – 1.06 (m, 18H), 1.00 (d, J = 6.8 Hz, 6H). ^{13}C NMR (101 MHz, $CDCl_3$) δ 153.5, 149.3, 139.6, 128.1, 121.3, 117.6, 117.0, 71.0, 54.0, 34.0, 32.9, 24.1, 23.8, 19.6, 18.7, 18.4. HRMS (EI, m/z): calcd for $C_{30}H_{42}N_4$ (M^+) m/z: 458.3409, found: 458.3411.

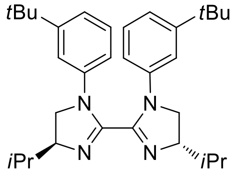

L1e

(*4S,4'S*)-1,1'-bis(3-(tert-butyl)phenyl)-4,4'-diisopropyl-4,4',5,5'-tetrahydro-*1H,1'H*-2,2'-biimidazole (L1e): white solid. 42% yield. m.p. 116 – 118 °C (PE/EA). IR (neat, cm^{-1}): 2958, 2870, 1641, 1581, 1493, 1475. Optical Rotation: $[\alpha]_D^{20}$ = -148.8 (c 0.99, $CHCl_3$). ^1H NMR (400 MHz, $CDCl_3$) δ 7.04 – 6.87 (m, 4H), 6.69 (s, 2H), 6.49 – 6.45 (m, 2H), 4.02 – 3.87 (m, 2H), 3.73 (t, J = 9.6 Hz, 2H), 3.53 (t, J = 10.0 Hz, 2H), 2.00 – 1.87 (m, 2H), 1.22 (s, 18H), 1.12 (d, J = 6.8 Hz, 6H), 0.96 (d, J = 6.8 Hz, 6H). ^{13}C NMR (101 MHz, $CDCl_3$) δ 153.5, 151.6, 139.6, 127.8, 120.4, 116.9, 116.7, 71.2, 54.3, 34.6, 33.0, 31.4, 19.7, 18.8. HRMS (EI, m/z): calcd for $C_{26}H_{34}N_4$ (M^+) m/z: 486.3722, found: 486.3723.

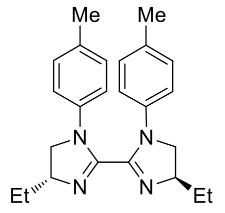

L1f

(*4R,4'R*)-4,4'-diethyl-1,1'-di-*p*-tolyl-4,4',5,5'-tetrahydro-*1H,1'H*-2,2'-biimidazole (L1f): white solid. 27% yield. m.p. 90 – 91 °C (PE/EA). IR (neat, cm^{-1}): 2924, 2856, 1631, 1592, 1518, 1462, 1319. Optical Rotation: $[\alpha]_D^{20}$ = -250.0 (c 1.02, $CHCl_3$). ^1H NMR (400 MHz, $CDCl_3$) δ 6.89 (d, *J* = 8.4 Hz, 4H), 6.59 (d, *J* = 8.4 Hz, 4H), 4.20 – 4.05 (M, 2H), 3.74 (t, *J* = 9.2 Hz, 2H), 3.41 (t, *J* = 9.2 Hz, 2H), 2.25 (s, 6H), 1.90 – 1.76 (m, 2H), 1.70 – 1.55 (m, 2H), 1.03 (t, *J* = 7.2 Hz, 6H). ^{13}C NMR (101 MHz, $CDCl_3$) δ 153.5, 137.3, 132.7, 128.7, 119.6, 66.2, 55.8, 28.6, 20.6, 10.4. HRMS (EI, m/z): calcd for $C_{24}H_{30}N_4$ (M^+) m/z: 374.2470, found: 374.2468.

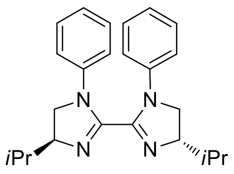

L1g

(*4S,4'S*)-4,4'-diisopropyl-1,1'-diphenyl-4,4',5,5'-tetrahydro-*1H,1'H*-2,2'-biimidazole (L1g): white solid. 23% yield. m.p. 104 – 106 °C (PE/EA). IR (neat, cm^{-1}): 2957, 2926, 1639, 1591, 1501, 1473. Optical Rotation: $[\alpha]_D^{20}$ = -361.2 (c 0.99, $CHCl_3$). ^1H NMR (400 MHz, $CDCl_3$) δ 7.04 (t, *J* = 7.6 Hz, 4H), 6.89 (t, *J* = 7.6 Hz, 2H), 6.63 (d, *J* = 7.6 Hz, 4H), 4.07 – 4.00 (m, 2H), 3.64 (t, *J* = 9.6 Hz, 2H), 3.55 – 3.50 (m, 2H), 2.01 – 1.93 (m, 2H), 1.11 (d, *J* = 6.8 Hz, 6H), 1.02 (d, *J* = 6.8 Hz, 6H). ^{13}C NMR (101 MHz, $CDCl_3$) δ 153.3, 139.6, 128.1, 123.0, 119.1, 71.0, 53.5, 32.6, 19.4, 18.8. HRMS (EI, m/z): calcd for $C_{28}H_{38}N_4$ (M^+) m/z: 374.2470, found: 374.2466.

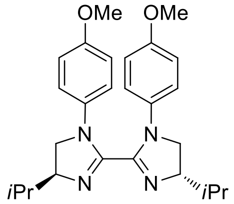

L1h

(*4S,4'S*)-4,4'-diisopropyl-1,1'-bis(4-methoxyphenyl)-4,4',5,5'-tetrahydro-*1H,1'H*-2,2'-biimidazole (L1h): white solid. 43% yield. m.p. 92 – 93 °C (PE/EA). Optical Rotation: $[\alpha]_D^{20}$ = -215.0 (c 1.00, $CHCl_3$). {Lit.[15] $[\alpha]_D^{25}$ = -257 (c 0.11, $CHCl_3$)}. ^1H NMR (400 MHz, $CDCl_3$) δ 6.66 – 6.58 (m, 8H), 4.05 – 3.90 (m, 2H), 3.76 (s, 6H), 3.58 (t, *J* = 8.8 Hz, 2H), 3.42 (t, *J* = 9.6 Hz, 2H), 1.98 – 1.84 (m, 2H), 1.07 (d, *J* = 6.8 Hz, 6H), 0.97 (d, *J* = 6.8 Hz, 6H). All spectra were in complete agreement with the previously reported values.[5]

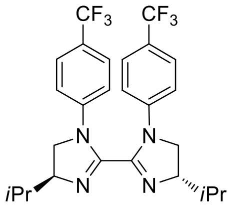

L1i

(*4S,4'S*)-4,4'-diisopropyl-1,1'-bis(4-(trifluoromethyl)phenyl)-4,4',5,5'-tetrahydro-*1H,1'H*-2,2'-biimidazole (L1i): white solid. 27% yield. m.p. 208 – 209 °C (PE/EA). IR (neat, cm^{-1}): 2957, 2925, 1616, 1579, 1524. Optical Rotation: $[\alpha]_D^{20}$ = -304.4 (c 1.01, $CHCl_3$). ^1H NMR (400 MHz, $CDCl_3$) δ 7.28 (d, *J* = 8.8 Hz, 4H), 6.65 (d, *J* = 8.0 Hz, 4H), 4.15 – 4.05 (m, 2H), 3.70 (t, *J* = 9.2 Hz, 2H), 3.64 – 3.48 (m, 2H), 2.10 – 1.95 (m, 2H), 1.15 (d, *J* = 6.4 Hz, 6H), 1.07 (d, *J* = 6.8 Hz, 6H). ^{13}C NMR (101 MHz, $CDCl_3$) δ 152.3, 142.2, 125.5 (q, *J* = 3.6 Hz), 124.9 (q, 33.1 Hz), 124.0 (q, 272.5 Hz), 118.1, 71.3, 53.3, 32.6, 19.4, 18.8. ^{19}F NMR (376 Hz, $CDCl_3$) δ -62.1. HRMS (EI, m/z): calcd for $C_{26}H_{28}F_6N_4$ (M^+) m/z: 510.2218, found: 510.2221.

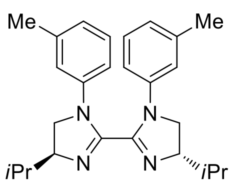

L1j

(*4S,4'S*)-4,4'-diisopropyl-1,1'-di-*m*-tolyl-4,4',5,5'-tetrahydro-*1H,1'H*-2,2'-biimidazole (L1j): white solid. 27% yield. m.p. 88 – 89 °C (PE/EA). IR (neat, cm^{-1}): 2957, 2925, 1586, 1494, 1470. Optical Rotation: $[\alpha]_D^{20}$ = -318.6 (c 0.82, $CHCl_3$). ^1H NMR (400 MHz, $CDCl_3$) δ 6.93 (t, *J* = 7.6 Hz, 2H), 6.72 (d, *J* = 7.6 Hz, 2H), 6.48 (d, *J* = 8.0 Hz, 2H), 6.38 (s, 2H), 4.09 – 3.89 (m, 2H), 3.61 (t, *J* = 8.8 Hz, 2H), 3.56 – 3.39 (m, 2H), 2.17 (s, 6H), 2.05 – 1.85 (m, 2H), 1.12 (d, *J* = 6.8 Hz, 6H), 1.01 (d, *J* = 6.8 Hz, 6H). ^{13}C NMR (101 MHz, $CDCl_3$) δ 153.6, 139.6, 138.0, 127.9, 123.8, 119.8, 116.7, 71.0, 53.7, 32.7, 21.2, 19.5, 18.9. HRMS (EI, m/z): calcd for $C_{26}H_{34}N_4$ (M^+) m/z: 402.2783, found:

402.2783.

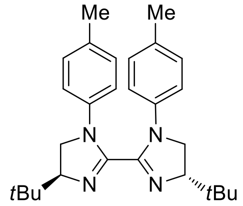

L1k

(*4S,4'S*)-4,4'-di-tert-butyl-1,1'-di-*p*-tolyl-4,4',5,5'-tetrahydro-1*H,1'H*-2,2'-biimidazole (L1k): white solid. 61% yield. m.p. 124 – 126 °C (PE/EA). IR (neat, cm^{-1}): 3033, 2865, 1617, 1590, 1517, 1477. Optical Rotation: $[\alpha]_D^{20}$ = -313.4 (c 1.00, $CHCl_3$). ^1H NMR (400 MHz, $CDCl_3$) δ 6.82 (d, *J* = 8.0 Hz, 4H), 6.51 (d, *J* = 8.4 Hz, 4H), 4.02 (dd, *J* = 13.2 10.4Hz, 2H), 3.58 (dd, *J* = 13.2, 9.2 Hz, 2H), 3.46 (t, *J* = 9.6 Hz, 2H), 2.24 (s, 6H), 1.05 (s, 18H). ^{13}C NMR (101 MHz, $CDCl_3$) δ 153.8, 137.3, 132.6, 128.6, 119.5, 74.5, 52.2, 33.7, 26.6, 20.7. HRMS (EI, m/z): calcd for $C_{28}H_{38}N_4$ (M^+) m/z: 430.3096, found: 430.3094.

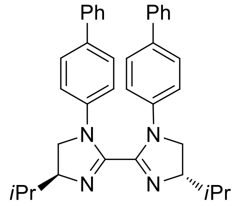

L1l

(*4S,4'S*)-1,1'-di([1,1'-biphenyl]-4-yl)-4,4'-diisopropyl-4,4',5,5'-tetrahydro-*1H, 1'H*-2,2'-biimidazole (L1l): white solid. 19% yield. m.p. 188 – 190 °C (PE/EA). IR (neat, cm^{-1}): 2956, 2924, 1638, 1591, 1524, 1487. Optical Rotation: $[\alpha]_D^{20}$ = -140.7 (c 1.00, $CHCl_3$). ^1H NMR (400 MHz, $CDCl_3$) δ 7.50 (d, *J* = 7.6 Hz, 4H), 7.43 (t, *J* = 7.6 Hz, 4H), 7.37 – 7.20 (m, 6H), 6.71 (d, *J* = 8.4 Hz, 4H), 4.15 – 3.95 (m, 2H), 3.69 (t, *J* = 9.2 Hz, 2H), 3.64 – 3.51 (m, 2H), 2.10 – 1.90 (m, 2H), 1.15 (d, *J* = 6.8 Hz, 6H), 1.06 (d, *J* = 6.8 Hz, 6H). ^{13}C NMR (101 MHz, $CDCl_3$) δ 153.3, 140.5, 138.9, 136.0, 128.8, 127.0, 126.8, 126.7, 119.4, 71.2, 53.6, 32.8, 19.5, 18.9. HRMS (EI, m/z): calcd for $C_{36}H_{38}N_4$ (M^+) m/z: 526.3096, found: 526.3098.

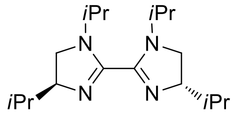

LS2

(*4S,4'S*)-1,1',4,4'-tetraisopropyl-4,4',5,5'-tetrahydro-*1H,1'H*-2,2'-biimidazole (LS2): yellow oil. 56% yield. IR (neat, cm^{-1}): 2960, 2872, 1633, 1567, 1464, 1365. Optical Rotation: $[\alpha]_D^{20}$ = -112.8 (c 1.03, $CHCl_3$). ^1H NMR (400 MHz, $CDCl_3$) δ 4.13 – 3.99 (m, 2H), 3.90 – 3.80 (m, 2H), 3.37 (t, *J* = 9.6 Hz, 2H), 3.04 (t, *J* = 9.2 Hz, 2H), 1.85 – 1.72 (m, 2H), 1.18 (d, *J* = 6.4 Hz, 6H), 1.07 (d, *J* = 6.8 Hz, 6H), 0.98 (d, *J* = 6.8 Hz, 6H), 0.91 (d, *J* = 6.8 Hz, 6H). ^{13}C NMR (101 MHz, $CDCl_3$) δ 156.6, 70.4, 46.0, 44.7, 33.2, 20.7, 20.2, 18.8, 18.3. HRMS (EI, m/z): calcd for $C_{18}H_{34}N_4$ (M^+) m/z: 306.2783, found: 306.2786.

Procedure B for the preparations of BiOX Ligands::

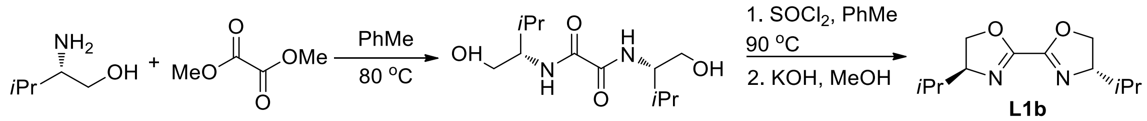

L1b

The synthesises of **LS1** was according to the previously reported method described by Reisman and coworkers.[6] (*S*)-2-amino-3-isopropane-1-ol (2.0 equiv., 10 mmol, 1.0645 g) and dimethyloxalate (1.0 equiv., 10 mmol, 0.5968 g) were dissolved in toluene (100 mL) and heated to 80 °C. The reaction was allowed to stir overnight with the diamide precipitating out of solution as a white solid. Reaction was cooled to room temperature and concentrated *in vacuo*. The crude diol was dissolved in toluene (20 mL) and heated to 70 °C whereupon thionyl chloride (2.0 equiv., 10 mmol, 0.8 mL) was added. Reaction was stirred at 70 °C for 30 minutes then heated to 90 °C for 2 h. The reaction was cooled to room temperature and poured into 20% KOH solution at 0 °C. The aqueous layer was separated and extracted three times with DCM and the combined organic layers were washed with 20% KOH solution, saturated $NaHCO_3$ solution and brine. The organic layer was dried with Na_2SO_4, filtered, and concentrated under reduced

pressure to afford the dichloro-intermediate as a sticky brown solid. The crude dichloro-intermediate was immediately dissolved in MeOH (50 mL) and KOH (12.5 mmol, 0.70 g) was added. The reaction was heated to reflux for 14 hours. The reaction was cooled to room temperature and concentrated to remove MeOH. The residue was purified by flash column chromatography (PE:EA = 2:1) to give **LS1** (0.7945 g, 3.5 mmol) in 70% yield as a white solid. All the biimidazole ligands (**LS1**, **SL3a**[7], **SL3b**, **SL3c**, **SL3d**, **SL3e**, **SL3f**, **SL3g**, **SL3h, SL3i**[8]) in this research were synthesized by following Procedure A.

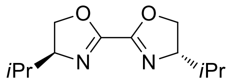

LS1

(*4S,4'S*)-4,4'-diisopropyl-4,4',5,5'-tetrahydro-2,2'-bioxazole (LS1): **LS1** was obtained by following Procedure B in 70% yield as a white solid. m.p. 73 – 75 °C (PE/EA). IR (neat, cm^{-1}): 2960, 1679, 1620, 1469, 1367, 1250. Optical Rotation: $[\alpha]_D^{20}$ = -157.3 (c 1.09, $CHCl_3$). ^{1}H NMR (400 MHz, $CDCl_3$) δ 4.57 – 4.34 (m, 2H), 4.22 – 3.99 (m, 4H), 1.95 – 1.74 (m, 2H), 1.03 (dd, *J* = 6.8, 1.6 Hz, 6H), 0.93 (dd, *J* = 6.8, 1.6 Hz, 6H). ^{13}C NMR (101 MHz, $CDCl_3$) δ 154.5, 73.1, 71.0, 32.4, 18.9, 18.2. HRMS (EI, m/z): calcd for $C_{12}H_{20}N_2O_2$ (M^+) m/z: 224.1525, found: 224.1522.

Other *N,N*-bidentate chiral ligands such as Pydine-oxazole ligands (**SL4a**[9], **SL4b**[10], **SL4c**[11], **SL4d**[12], **SL4f**[13]), pydine-imidazole ligand (**SL4g**), chiral biiminyl ligand (**SL6**), iminyl oxazole ligand (**SL10**) and amino oxazole ligand (**SL9**[14]) are prepared according to the previously reported procedures.

Standard Conditions A for chiral 1,1-diaryl alkanes

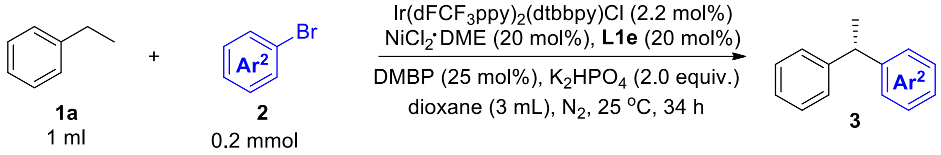

To a 20 mL vial with a stir bar was added **L1e** (0.0195 g, 0.04 mmol, 20 mol%), $NiCl_2$•DME (0.0087 g, 0.04 mmol, 20 mol%) and 1 mL of dioxane in a N_2-filled glovebox. The reaction was stirred at 50 °C for 30 minutes before cooled to room temperature. 2 mL of dioxane, 1 mL of ethyl benzene **1a**, benzyl bromide **2** (0.2 mmol, 1.0 equiv.), Ir(dFCF$_3$ppy)$_2$(dtbbpy)Cl (0.0044 g, 0.0044 mmol, 2.2 mol%), DMBP (bis(4-methoxyphenyl)methanone) (0.0121 g, 0.05 mmol, 25 mol%) and K_2HPO_4 (0.0696 g, 0.4 mmol, 2.0 equiv.) were added consistently. The vial was sealed with a Teflon cap and then allowed to remove from the glovebox. The reaction was stirred at 600 rpm under the irradiation of 8W blue LEDs in a distance of 5 cm at room temperature (25 °C) for 34 h. The reaction was quenched by adding Et_2O, filtered through a short pad of silica and eluted with Et_2O. The solution was concentrated under reduced pressure to afford the crude residue which was purified by flash column chromatography.

Standard Conditions B for chiral 1,1-diaryl alkanes

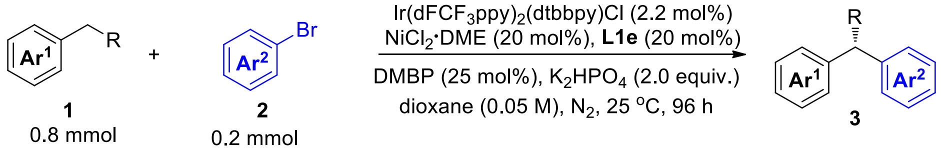

To a 20 mL vial with a stir bar was added **L1e** (0.0195 g, 0.04 mmol, 20 mol%), $NiCl_2$•DME (0.0087 g, 0.04 mmol, 20 mol%) and 1 mL of dioxane in a N_2-filled glovebox. The reaction was stirred at 50 °C for 30 minutes before cooled to room temperature. 3 mL of dioxane, **1** (0.8 mmol, 4.0 equiv.), benzyl bromide **2** (0.2 mmol, 1.0 equiv.), Ir(dFCF$_3$ppy)$_2$(dtbbpy)Cl (0.0044 g, 0.0044 mmol, 2.2 mol%), DMBP

(0.0121 g, 0.05 mmol, 25 mol%) and K_2HPO_4 (0.0696 g, 0.4 mmol, 2.0 equiv.) was added consistently. The vial was sealed with a Teflon cap and then allowed to remove from the glovebox. The reaction was stirred at 600 rpm under the irradiation of 8W blue LEDs in a distance of 5 cm at room temperature (25 ^{o}C) for 96 h. The reaction was quenched by adding Et_2O, filtered through a short pad of silica and eluted with Et_2O. The solution was concentrated under reduced pressure to afford the crude residue which was purified by flash column chromatography.

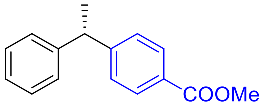

(*S*)-methyl 4-(1-phenylethyl)benzoate (3aa): 3aa was obtained by following Standard Conditions B using 84.2 mg (0.8 mmol) of **1a**, 42.6 mg (0.2 mmol) of **2a**, 4.2 mg (0.0044 mmol) of Ir(dFCF$_3$ppy)$_2$(dtbbpy)Cl, 8.3 mg (0.04 mmol) of $NiCl_2$•DME, 19.5 mg (0.04 mmol) of **L1e**, 12.3 mg (0.05 mmol) of DMBP and 68.6 mg (0.4 mmol) of K_2HPO_4 to afford 28.4 mg (60% yield) of **3aa** in 92.5:7.5 er as a colorless oil. IR (neat, cm^{-1}): 2967, 1722, 1610, 1435, 1279. Optical Rotation: $[\alpha]_D^{20}$ = +6.4 (c 0.64, $CHCl_3$). Chiral HPLC: (OD-H*2, 1.0 mL/min, *i*-PrOH/hexane = 99.8/0.2, λ = 220 nm): t_R (major) = 43.5 min, t_R (minor) = 45.5 min. ^{1}H NMR (400 MHz, $CDCl_3$) δ 7.95 (d, *J* = 8.0 Hz, 2H), 7.42 – 7.25 (m, 4H), 7.24 – 7.10 (m, 3H), 4.20 (q, *J* = 6.8 Hz, 1H), 3.89 (s, 3H), 1.65 (d, *J* = 7.2 Hz, 3H). ^{13}C NMR (101 MHz, $CDCl_3$) δ 167.0, 151.7, 145.4, 129.7, 128.5, 128.0, 127.65, 127.57, 126.3, 52.0, 44.8, 21.6. HRMS (EI, m/z): calcd for $C_{16}H_{16}O_2$ (M^+) m/z: 240.1150, found: 240.1151.

Run 2: **3aa** was obtained by following Standard Conditions A in 24 h using 43.6 mg (0.2 mmol) of **2a**, 4.8 mg (0.0044 mmol) of Ir(dFCF$_3$ppy)$_2$(dtbbpy)Cl, 8.7 mg (0.04 mmol) of $NiCl_2$•DME, 19.5 mg (0.04 mmol) of **L1e**, 12.6 mg (0.05 mmol) of DMBP and 70.0 mg (0.4 mmol) of K_2HPO_4 to afford **3aa** in 44% yield (determined by ^{1}H NMR using TMSPh as an internal standard) in 95:5 er as a colorless oil.

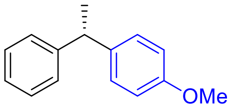

(*S*)-1-methoxy-4-(1-phenylethyl)benzene (3ab): 3ab was obtained by following Standard Conditions A using 37.1 mg (0.2 mmol) of **2b**, 4.2 mg (0.0044 mmol) of Ir(dFCF$_3$ppy)$_2$(dtbbpy)Cl, 8.6 mg (0.04 mmol) of $NiCl_2$•DME, 19.5 mg (0.04 mmol) of **L1e**, 12.4 mg (0.05 mmol) of DMBP and 69.7 mg (0.4 mmol) of K_2HPO_4 to afford 24.3 mg (58% yield) of **3ab** in 95.5:4.5 er as a colorless oil. Optical Rotation: $[\alpha]_D^{20}$ = +7.7 (c 0.90, $CHCl_3$).{Lit.[15] $[\alpha]_D^{20}$ = +8.2 (c 0.72, $CHCl_3$), 89% ee}. Chiral HPLC: (AS-H*2, 1.0 mL/min, *i*-PrOH/hexane = 99.9/0.1, λ = 220 nm): t_R (major) = 14.9 min, t_R (minor) = 12.8 min. ^{1}H NMR (400 MHz, $CDCl_3$) δ 7.31 – 7.23 (m, 2H), 7.22 – 7.16 (m, 2H), 7.16 – 7.10 (m, 2H), 6.86 – 6.78 (m, 2H), 4.10 (q, *J* = 7.2 Hz, 1H), 3.77 (s, 3H), 1.61 (d, *J* = 7.2 Hz, 3H). ^{13}C NMR (101 MHz, $CDCl_3$) δ 157.8, 146.8, 138.6, 128.5, 128.3, 127.5, 125.9, 113.7, 55.2, 43.9. 22.0. All spectra were in complete agreement with the previously reported values.[15]

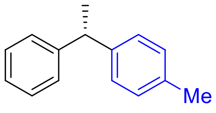

(*S*)-1-methyl-4-(1-phenylethyl)benzene (3ac): 3ac was obtained by following Standard Conditions A using 34.6 mg (0.2 mmol) of **2c**, 4.6 mg (0.0044 mmol) of Ir(dFCF$_3$ppy)$_2$(dtbbpy)Cl, 8.8 mg (0.04 mmol) of $NiCl_2$•DME, 19.3 mg (0.04 mmol) of **L1e**, 12.5 mg (0.05 mmol) of DMBP and 69.5 mg (0.4 mmol) of K_2HPO_4 to afford 23.8 mg (61% yield) of **3ac** in 95:5 er as a colorless oil. Optical Rotation: $[\alpha]_D^{20}$ = +3.1 (c 0.46, $CHCl_3$). {Lit.[16] $[\alpha]_D^{22}$ = +0.5 (c 0.10, $CHCl_3$), 62% ee}. Chiral HPLC: (OD-H, 1.0 mL/min, *i*-PrOH/hexane = 99.9/0.1, λ = 220 nm): t_R (major) = 7.4 min, t_R (minor) = 8.0 min. ^{1}H NMR (400 MHz, $CDCl_3$) δ 7.30 – 7.25 (m, 2H), 7.24 – 7.18 (m, 2H), 7.17 – 7.14 (m, 1H), 7.14 – 7.06 (m, 4H), 4.12 (q, *J* = 7.2 Hz, 1H), 2.30 (s, 3H), 1.62 (d, *J* = 7.6 Hz, 3H). ^{13}C NMR (101 MHz, $CDCl_3$) δ 136.6, 143.4, 135.5, 129.0, 128.3, 127.55, 127.46, 125.9, 44.3, 21.9, 21.0. All spectra were in complete agreement with the previously reported values.[16]

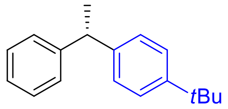

(*S*)-1-(*tert*-butyl)-4-(1-phenylethyl)benzene (3ad): 3ad was obtained by following Standard Conditions A using 42.6 mg (0.2 mmol) of **2d**, 4.5 mg (0.0044 mmol) of Ir(dFCF$_3$ppy)$_2$(dtbbpy)Cl, 8.7 mg (0.04 mmol) of NiCl$_2$•DME, 16.4 mg (0.04 mmol) of **L1e**, 12.3 mg (0.05 mmol) of DMBP and 70.3 mg (0.4 mmol) of K$_2$HPO$_4$ to afford 39.9 mg (84% yield) of **3ad** in 96:4 er as a colorless oil. Optical Rotation: $[\alpha]_D^{20}$ = +7.6 (c 1.24, CHCl$_3$, 89% ee). {Lit.[16] $[\alpha]_D^{22}$ = +2.5 (c 0.10, CHCl$_3$), 65% ee}. Chiral HPLC: (OD-H, 0.5 mL/min, 100% hexane, λ = 220 nm): t_R (major) = 12.1 min, t_R (minor) = 11.7 min. ^{1}H NMR (400 MHz, CDCl$_3$) δ 7.32 – 7.21 (m, 6H), 7.20 – 7.12 (m, 3H), 4.12 (q, *J* = 7.2 Hz, 1H), 1.63 (d, *J* = 7.2 Hz, 3H), 1.29 (s, 9H). ^{13}C NMR (101 MHz, CDCl$_3$) δ 148.7, 146.6, 143.2, 128.3, 127.6, 127.1, 125.9, 125.2, 44.3, 34.3, 31.4, 21.9. All spectra were in complete agreement with the previously reported values.[16]

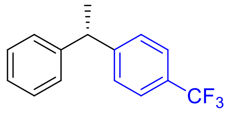

(*S*)-1-(1-phenylethyl)-4-(trifluoromethyl)benzene (3ae): 3ae was obtained by following Standard Conditions A using 45.9 mg (0.2 mmol) of **2e**, 4.3 mg (0.0044 mmol) of Ir(dFCF$_3$ppy)$_2$(dtbbpy)Cl, 8.7 mg (0.04 mmol) of NiCl$_2$•DME, 19.0 mg (0.04 mmol) of **L1e**, 12.5 mg (0.05 mmol) of DMBP and 70.0 mg (0.4 mmol) of K$_2$HPO$_4$ to afford 23.0 mg (45% yield) of **3ae** in 94.5:5.5 er as a colorless oil. IR (neat, cm^{-1}): 2956, 2924, 1460, 1377, 1326. Optical Rotation: $[\alpha]_D^{20}$ = -3.4 (c 0.35, CHCl$_3$). Chiral HPLC: (OJ-H, 1.0 mL/min, 100% hexane, λ = 220 nm): t_R (major) = 7.8 min, t_R (minor) = 8.4 min. ^{1}H NMR (400 MHz, CDCl$_3$) δ 7.53 (d, *J* = 8.0 Hz, 2H), 7.36 – 7.27 (m, 4H), 7.25 – 7.17 (m, 3H), 4.21 (q, *J* = 7.2 Hz, 1H), 1.66 (d, *J* = 7.2 Hz, 3H). ^{13}C NMR (101 MHz, CDCl$_3$) δ 150.4, 145.2, 128.5, 128.3 (q, *J* = 32.4 Hz), 127.9, 127.6, 124.2 (q, *J* = 273.2 Hz), 126.4, 125.3 (q, *J* = 3.6 Hz), 44.6, 21.6. ^{19}F NMR (376 MHz, CDCl$_3$) δ -62.4. HRMS (EI, m/z): calcd for $C_{15}H_{13}F_3$ (M^+) m/z: 250.0969, found: 250.0968.

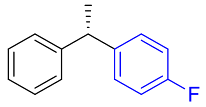

(*S*)-1-fluoro-4-(1-phenylethyl)benzene (3af): 3af was obtained by following Standard Conditions A using 35.8 mg (0.2 mmol) of **2f**, 4.2 mg (0.0044 mmol) of Ir(dFCF$_3$ppy)$_2$(dtbbpy)Cl, 8.8 mg (0.04 mmol) of NiCl$_2$•DME, 18.8 mg (0.04 mmol) of **L1e**, 12.4 mg (0.05 mmol) of DMBP and 69.2 mg (0.4 mmol) of K$_2$HPO$_4$ to afford 29.0 mg (71% yield) of **3af** in 93.5:6.5 er as a colorless oil. IR (neat, cm^{-1}): 2956, 2924, 2854, 1509, 1458. Optical Rotation: $[\alpha]_D^{20}$ = +3.0 (c 0.19, CHCl$_3$). Chiral HPLC: (OJ-H, 1.0 mL/min, 100% hexane, λ = 220 nm): t_R (major) = 16.6 min, t_R (minor) = 24.7 min. ^{1}H NMR (400 MHz, CDCl$_3$) δ 7.32 – 7.24 (m, 2H), 7.24 – 7.12 (m, 5H), 6.96 (t, *J* = 8.4 Hz, 2H), 4.13 (q, *J* = 7.2 Hz, 1H), 1.62 (d, *J* = 7.2 Hz, 3H). ^{13}C NMR (101 MHz, CDCl$_3$) δ 161.2 (d, *J* = 244.8 Hz), 146.1, 142.0 (d, *J* = 3.3 Hz), 129.0 (d, *J* = 8.2 Hz), 128.4, 127.5, 126.1, 115.0 (d, *J* = 21.4 Hz), 44.0, 22.0. ^{19}F NMR (376 MHz, CDCl$_3$) δ -117.5. HRMS (EI, m/z): calcd for $C_{14}H_{13}F$ (M^+) m/z: 200.1001, found: 200.1001.

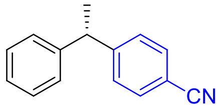

(*S*)-4-(1-phenylethyl)benzonitrile (3ag): 3ag was obtained by following Standard Conditions A using 36.8 mg (0.2 mmol) of **2g**, 4.3 mg (0.0044 mmol) of Ir(dFCF$_3$ppy)$_2$(dtbbpy)Cl, 8.4 mg (0.04 mmol) of NiCl$_2$•DME, 19.5 mg (0.04 mmol) of **L1e**, 12.5 mg (0.05 mmol) of DMBP and 69.5 mg (0.4 mmol) of K$_2$HPO$_4$ to afford 20.5 mg (50% yield) of **3ag** in 93:7 er as a colorless oil. Optical Rotation: $[\alpha]_D^{20}$ = +5.9 (c 0.74, CHCl$_3$, 77% ee). {Lit.[16] $[\alpha]_D^{22}$ = +3.1 (c 0.10, CHCl$_3$), 57% ee}. Chiral HPLC: (OJ-H, 1.0 mL/min, *i*-PrOH/hexane = 98/2, λ = 220 nm): t_R (major) = 18.8 min, t_R (minor) = 20.1 min. ^{1}H NMR (400 MHz, CDCl$_3$) δ 7.56 (d, *J* = 8.0 Hz, 2H), 7.35 – 7.27 (m, 4H), 7.27 – 7.21 (m, 1H), 7.20 – 7.14 (m, 2H), 4.20 (q, *J* = 7.2 Hz, 1H), 1.65 (d,

J = 7.2 Hz, 3H). ^{13}C NMR (101 MHz, $CDCl_3$) δ 151.9, 144.6, 132.2, 128.6, 128.4, 127.5, 126.6, 119.0, 109.9, 44.8, 21.4. All spectra were in complete agreement with the previously reported values.[16]

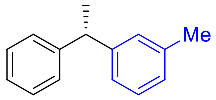

(*S*)-1-methyl-3-(1-phenylethyl)benzene (3ah): 3ah was obtained by following Standard Conditions A for 48 h using 35.1 mg (0.2 mmol) of **2h**, 4.3 mg (0.0044 mmol) of Ir(dFCF$_3$ppy)$_2$(dtbbpy)Cl, 8.4 mg (0.04 mmol) of NiCl$_2$•DME, 19.8 mg (0.04 mmol) of **L1e**, 12.0 mg (0.05 mmol) of DMBP and 69.5 mg (0.4 mmol) of K_2HPO_4 to afford 32.1 mg (82% yield) of **3ah** in 95.5:4.5 er as a colorless oil. Optical Rotation: $[\alpha]_D^{20}$ = +1.3 (c 0.41, $CHCl_3$). {Lit.[16] $[\alpha]_D^{20}$ = -0.7 (c 1.12, $CHCl_3$), 91.7% ee (*R*)}. Chiral HPLC: (OJ-H, 1.0 mL/min, 100% hexane, λ = 220 nm): t_R (major) = 22.3 min, t_R (minor) = 25.3 min. ^{1}H NMR (400 MHz, $CDCl_3$) δ 7.31 – 7.25 (m, 2H), 7.24 – 7.20 (m, 2H), 7.20 – 7.13 (m, 2H), 7.06 – 6.96 (m, 3H), 4.11 (q, J = 7.2 Hz, 1H), 2.31 (s, 3H), 1.62 (d, J = 7.2 Hz, 3H). ^{13}C NMR (101 MHz, $CDCl_3$) δ 146.4, 146.3, 137.8, 128.4, 128.3, 128.2, 127.6, 126.8, 125.9, 124.6, 44.7, 21.9, 21.5. All spectra were in complete agreement with the previously reported values.[16]

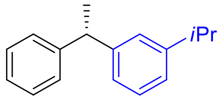

(*S*)-1-isopropyl-3-(1-phenylethyl)benzene (3ai): 3ai was obtained by following Standard Conditions A using 39.7 mg (0.2 mmol) of **2i**, 4.5 mg (0.0044 mmol) of Ir(dFCF$_3$ppy)$_2$(dtbbpy)Cl, 8.6 mg (0.04 mmol) of NiCl$_2$•DME, 19.8 mg (0.04 mmol) of **L1e**, 12.0 mg (0.05 mmol) of DMBP and 69.5 mg (0.4 mmol) of K_2HPO_4 to afford 39.0 mg (87% yield) of **3ai** in 94:6 er as a colorless oil. IR (neat, cm^{-1}): 2962, 2927, 1602, 1491, 1456. Optical Rotation: $[\alpha]_D^{20}$ = -2.1 (c 1.85, $CHCl_3$). Chiral HPLC: (OJ-H, 1.0 mL/min, 100% hexane, λ = 220 nm): t_R (major) = 7.2 min, t_R (minor) = 7.8 min. ^{1}H NMR (400 MHz, $CDCl_3$) δ 7.32 – 7.14 (m, 6H), 7.11 – 6.98 (m, 3H), 4.13 (q, J = 7.2 Hz, 1H), 2.92 – 2.80 (m, 1H), 1.64 (d, J = 7.6 Hz, 3H), 1.22 (d, J = 6.8 Hz, 6H). ^{13}C NMR (101 MHz, $CDCl_3$) δ 148.8, 146.5, 146.2, 128.3 128.2, 127.6, 126.0, 125.9, 125.0, 123.9, 44.9, 34.1, 24.03, 24.00, 22.0. HRMS (EI, m/z): calcd for $C_{17}H_{20}$ (M^+) m/z: 224.1565, found: 224.1567.

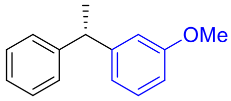

(*S*)-1-methoxy-3-(1-phenylethyl)benzene (3aj): 3aj was obtained by following Standard Conditions A using 37.4 mg (0.2 mmol) of **2j**, 4.7 mg (0.0044 mmol) of Ir(dFCF$_3$ppy)$_2$(dtbbpy)Cl, 8.4 mg (0.04 mmol) of NiCl$_2$•DME, 19.5 mg (0.04 mmol) of **L1e**, 12.5 mg (0.05 mmol) of DMBP and 69.8 mg (0.4 mmol) of K_2HPO_4 to afford 28.4 mg (67% yield) of **3aj** in 94.5:5.5 er as a colorless oil. IR (neat, cm^{-1}): 2956, 2925, 1599, 1489, 1456. Optical Rotation: $[\alpha]_D^{20}$ = +2.5 (c 0.90, $CHCl_3$) Chiral HPLC: (OD-H*2, 1.0 mL/min, *i*-PrOH/hexane = 98/2, λ = 220 nm): t_R (major) = 12.3 min, t_R (minor) = 14.3 min.. ^{1}H NMR (400 MHz, $CDCl_3$) δ 7.32 – 7.13 (m, 6H), 6.85 – 6.66 (m, 3H), 4.12 (q, J = 7.2 Hz, 1H), 3.77 (s, 3H), 1.63 (d, J = 7.2 Hz, 3H). ^{13}C NMR (101 MHz, $CDCl_3$) δ 159.6, 148.0, 146.1, 129.3, 128.3, 127.5, 126.0, 120.1, 113.8, 110.9, 55.1, 44.8, 21.8. HRMS (EI, m/z): calcd for $C_{15}H_{16}O$ (M^+) m/z: 212.1201, found: 212.1203.

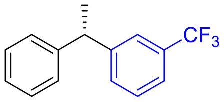

(*S*)-1-(1-phenylethyl)-3-(trifluoromethyl)benzene (3ak): 3ak was obtained by following Standard Conditions A using 45.9 mg (0.2 mmol) of **2k**, 4.4 mg (0.0044 mmol) of Ir(dFCF$_3$ppy)$_2$(dtbbpy)Cl, 8.7 mg (0.04 mmol) of NiCl$_2$•DME, 20.0 mg (0.04 mmol) of **L1e**, 12.0 mg (0.05 mmol) of DMBP and 69.4 mg (0.4 mmol) of K_2HPO_4 to afford 30.9 mg (63% yield) of **3ak** in 95:5 er as a colorless oil. IR (neat, cm^{-1}): 2958, 2926, 1455, 1377, 1330. Optical Rotation: $[\alpha]_D^{20}$ = +2.0 (c 1.38, $CHCl_3$). Chiral HPLC: (OJ-H, 1.0 mL/min, 100% hexane, λ = 220 nm): t_R (major) = 8.2 min, t_R (minor) = 8.7 min. ^{1}H NMR (400 MHz, $CDCl_3$) δ 7.49 (s, 1H), 7.53 – 7.42 (m, 1H),

7.35 – 7.27 (m, 2H), 7.40 – 7.32 (m, 2H), 7.25 – 7.17 (m, 3H), 4.26 (q, J = 7.2 Hz, 1H), 1.72 (d, J = 7.2 Hz, 3H). ^{13}C NMR (101 MHz, $CDCl_3$) δ 147.3, 145.3, 131.1 (q, J = 1.1 Hz), 130.6 (q, J = 32.0 Hz)), 128.8, 128.6, 127.5, 126.4, 124.23 (q, J = 273.6 Hz), 124.22 (q, J = 4.0 Hz), 123.0 (q, J = 3.6 Hz), 44.6, 21.7. ^{19}F NMR (376 MHz, $CDCl_3$) δ -62.5. HRMS (EI, m/z): calcd for $C_{15}H_{13}F_3$ (M^+) m/z: 250.0969, found: 250.0967.

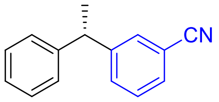

(*S*)-3-(1-phenylethyl)benzonitrile (3al): 3al was obtained by following Standard Conditions A using 36.6 mg (0.2 mmol) of **2l**, 4.4 mg (0.0044 mmol) of Ir(dFCF$_3$ppy)$_2$(dtbbpy)Cl, 8.6 mg (0.04 mmol) of $NiCl_2$•DME, 16.6 mg (0.04 mmol) of **L1e**, 12.0 mg (0.05 mmol) of DMBP and 69.3 mg (0.4 mmol) of K_2HPO_4 to afford 24.6 mg (59% yield) of **3al** in 90:10 er as a colorless oil. IR (neat, cm^{-1}): 2961, 2925, 2229, 1600, 1454. Optical Rotation: $[\alpha]_D^{20}$ = +10.5 (c 0.70, $CHCl_3$). Chiral HPLC: (OJ-H, 1.0 mL/min, *i*-PrOH/hexane = 85/15, λ = 220 nm): t_R (major) = 9.6 min, t_R (minor) = 10.0 min. ^{1}H NMR (400 MHz, $CDCl_3$) δ 7.50 (s, 1H), 7.49 – 7.42 (m, 2H), 7.40 – 7.27 (m, 3H), 7.26 – 7.15 (m, 3H), 4.17 (q, J = 7.2 Hz, 1H), 1.64 (d, J = 7.2 Hz, 3H). ^{13}C NMR (101 MHz, $CDCl_3$) δ 147.8, 144.7, 132.3, 131.2, 129.8, 129.1, 128.7, 127.5, 126.6, 119.0, 112.4, 44.4, 21.5. HRMS (EI, m/z): calcd for $C_{15}H_{13}N$ (M^+) m/z: 207.1048, found: 207.1049.

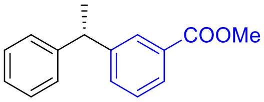

(*S*)-methyl 3-(1-phenylethyl)benzoate (3am): 3am was obtained by following Standard Conditions A using 43.1 mg (0.2 mmol) of **2m**, 4.5 mg (0.0044 mmol) of Ir(dFCF$_3$ppy)$_2$(dtbbpy)Cl, 8.6 mg (0.04 mmol) of $NiCl_2$•DME, 19.5 mg (0.04 mmol) of **L1e**, 12.2 mg (0.05 mmol) of DMBP and 68.6 mg (0.4 mmol) of K_2HPO_4 to afford 31.4 mg (65% yield) of **3am** in 93.5:6.5 er as a colorless oil. Optical Rotation: $[\alpha]_D^{20}$ = +6.8 (c 0.73, $CHCl_3$). Chiral HPLC: (OJ-H, 1.0 mL/min, *i*-PrOH/hexane = 99/1, λ = 220 nm): t_R (major) = 21.2 min, t_R (minor) = 20.3 min. ^{1}H NMR (400 MHz, $CDCl_3$) δ 7.94 (s, 1H), 7.86 (d, J = 7.6 Hz, 1H), 7.39 (d, J = 7.6 Hz, 1H), 7.37 – 7.24 (m, 3H), 7.23 – 7.15 (m, 3H), 4.20 (q, J = 7.2 Hz, 1H), 3.89 (s, 3H), 1.66 (d, J = 7.2 Hz, 3H). ^{13}C NMR (101 MHz, $CDCl_3$) δ 167.2, 146.7, 145.7, 132.4, 130.2, 128.6, 128.5, 128.4, 127.5, 127.4, 126.2, 52.1, 44.6, 21.7. All spectra were in complete agreement with the previously reported values.[17]

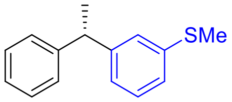

(*S*)-methyl(3-(1-phenylethyl)phenyl)sulfane (3an): 3an was obtained by following Standard Conditions A using 43.9 mg (0.2 mmol) of **2n**, 4.3 mg (0.0044 mmol) of Ir(dFCF$_3$ppy)$_2$(dtbbpy)Cl, 8.8 mg (0.04 mmol) of $NiCl_2$•DME, 19.2 mg (0.04 mmol) of **L1e**, 12.1 mg (0.05 mmol) of DMBP and 69.6 mg (0.4 mmol) of K_2HPO_4 to afford 16.7 mg (34% yield) of **3an** in 95.5:4.5 er as a colorless oil. IR (neat, cm^{-1}): 3026, 2966, 2922, 1589, 1451. Optical Rotation: $[\alpha]_D^{20}$ = -3.2 (c 0.53, $CHCl_3$). Chiral HPLC: (OJ-H, 1.0 mL/min, *i*-PrOH/hexane = 99/1, λ = 220 nm): t_R (major) = 18.2 min, t_R (minor) = 16.3 min. ^{1}H NMR (400 MHz, $CDCl_3$) δ 7.31 – 7.24 (m, 2H), 7.24 – 7.16 (m, 4H), 7.13 (s, 1H), 7.07 (d, J = 7.6 Hz, 1H), 6.98 (d, J = 7.6 Hz, 1H), 4.11 (q, J = 6.8 Hz, 1H), 2.45 (s, 3H), 1.62 (d, J = 7.2 Hz, 3H). ^{13}C NMR (101 MHz, $CDCl_3$) δ 147.0, 145.9, 138.3, 128.8, 128.4, 127.6, 126.1, 126.0, 124.6, 124.2, 44.7, 21.8, 15.8. HRMS (EI, m/z): calcd for $C_{15}H_{16}S$ (M^+) m/z: 228.0973, found: 228.0971.

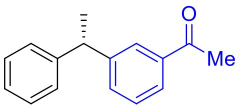

(*S*)-1-(3-(1-phenylethyl)phenyl)ethanone (3ao): 3ao was obtained by following Standard Conditions A using 39.9 mg (0.2 mmol) of **2o**, 4.4 mg (0.0044 mmol) of Ir(dFCF$_3$ppy)$_2$(dtbbpy)Cl, 8.7 mg (0.04 mmol) of $NiCl_2$•DME,

16.2 mg (0.04 mmol) of **L1e**, 11.8 mg (0.05 mmol) of DMBP and 68.8 mg (0.4 mmol) of K_2HPO_4 to afford 29.7 mg (66% yield) of **3ao** in 92.5:7.5 er as a colorless oil. IR (neat, cm^{-1}): 2957, 2925, 1686, 1493, 1459. Optical Rotation: $[\alpha]_D^{20}$ = -1.0 (c 0.49, $CHCl_3$). Chiral HPLC: (OD-H*2, 1.0 mL/min, *i*-PrOH/hexane = 98/2, λ = 220 nm): t_R (major) = 21.8 min, t_R (major) = 23.0 min. ^{1}H NMR (400 MHz, $CDCl_3$) δ 7.86 (s, 1H), 7.77 (d, *J* = 7.2 Hz, 1H), 7.44 – 7.34 (m, 2H), 7.29 (t, *J* = 7.6 Hz, 2H), 7.24 – 7.16 (m, 3H), 4.21 (q, *J* = 7.2 Hz, 1H), 2.57 (s, 3H), 1.67 (d, *J* = 7.2 Hz, 3H). ^{13}C NMR (101 MHz, $CDCl_3$) δ 198.3, 147.0, 145.6, 137.2, 132.5, 128.6, 128.5, 127.5, 127.2, 126.33, 126.26, 44.7, 26.7, 21.7. HRMS (EI, m/z): calcd for $C_{16}H_{16}O$ (M^+) m/z: 224.1201, found: 224.1201.

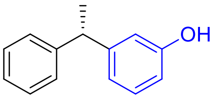

(*S*)-3-(1-phenylethyl)phenol (3ap): 3ap was obtained by following Standard Conditions A using 34.9 mg (0.2 mmol) of **2p**, 4.2 mg (0.0044 mmol) of Ir(dFCF$_3$ppy)$_2$(dtbbpy)Cl, 8.9 mg (0.04 mmol) of $NiCl_2$•DME, 19.6 mg (0.04 mmol) of **L1e**, 12.9 mg (0.05 mmol) of DMBP and 69.4 mg (0.4 mmol) of K_2HPO_4 to afford 18.5 mg (46% yield) of **3ap** in 95:5 er as a colorless oil. IR (neat, cm^{-1}): 3359, 2966, 2928, 1595, 1491, 1455. Optical Rotation: $[\alpha]_D^{20}$ = +3.2 (c 1.04, $CHCl_3$). Chiral HPLC: (OJ-H, 1.0 mL/min, *i*-PrOH/hexane = 80/20, λ = 220 nm): t_R (major) = 19.1 min, t_R (minor) = 15.6 min. ^{1}H NMR (400 MHz, $CDCl_3$) δ 7.87 – 7.75 (m, 2H), 7.44 – 7.35 (m, 4H), 6.81 (d, *J* = 7.6 Hz, 1H), 6.69 – 6.62 (m, 2H), 4.71 (brs, 1H), 4.10 (q, *J* = 7.2 Hz, 1H), 1.61 (d, *J* = 7.2 Hz, 3H). ^{13}C NMR (101 MHz, $CDCl_3$) δ 155.5, 148.4, 146.0, 129.5, 128.4, 127.6, 126.1, 120.2, 114.6, 112.9, 44.6, 21.7. HRMS (EI, m/z): calcd for $C_{14}H_{14}O$ (M^+) m/z: 198.1045, found: 198.1047.

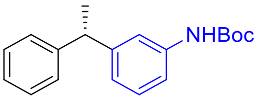

(*S*)-*tert*-butyl (3-(1-phenylethyl)phenyl)carbamate (3aq): 3aq was obtained by following Standard Conditions A using 56.6 mg (0.2 mmol) of **2q**, 4.6 mg (0.0044 mmol) of Ir(dFCF$_3$ppy)$_2$(dtbbpy)Cl, 8.4 mg (0.04 mmol) of $NiCl_2$•DME, 20.0 mg (0.04 mmol) of **L1e**, 12.5 mg (0.05 mmol) of DMBP and 70.6 mg (0.4 mmol) of K_2HPO_4 to afford 45.4 mg (73% yield) of **3aq** in 90.5:9.5 er as a colorless oil. IR (neat, cm^{-1}): 3337, 2924, 1706, 1607, 1530, 1492. Optical Rotation: $[\alpha]_D^{20}$ = -2.0 (c 2.03, $CHCl_3$). Chiral HPLC: (OJ-H, 1.0 mL/min, *i*-PrOH/hexane = 90/10, λ = 220 nm): t_R (major) = 13.7 min, t_R (minor) = 16.5 min. ^{1}H NMR (400 MHz, $CDCl_3$) δ 7.30 – 7.25 (m, 3H), 7.24 – 7.13 (m, 5H), 6.89 (d, *J* = 7.2 Hz, 1H), 6.40 (brs, 1H), 4.12 (q, *J* = 7.2 Hz, 1H), 1.62 (d, *J* = 7.2 Hz, 3H), 1.50 (s, 9H). ^{13}C NMR (101 MHz, $CDCl_3$) δ 208.9, 147.4, 146.1, 138.3, 129.0, 128.4, 128.3, 127.6, 126.0, 122.3, 44.7, 28.3, 24.5, 21.8. HRMS (EI, m/z): calcd for $C_{14}H_{15}N$ (M^+-$C_5H_8O_2$) m/z: 197.1204, found: 197.1206.

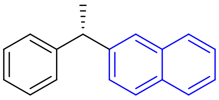

(*S*)-2-(1-phenylethyl)naphthalene (3ar): 3ar was obtained by following Standard Conditions A using 41.3 mg (0.2 mmol) of **2r**, 4.6 mg (0.0044 mmol) of Ir(dFCF$_3$ppy)$_2$(dtbbpy)Cl, 8.8 mg (0.04 mmol) of $NiCl_2$•DME, 19.6 mg (0.04 mmol) of **L1e**, 11.8 mg (0.05 mmol) of DMBP and 69.3 mg (0.4 mmol) of K_2HPO_4 to afford 22.6 mg (48% yield) of **3ar** in 95.5:4.5 er as a colorless oil. Optical Rotation: $[\alpha]_D^{20}$ = +48.0 (c 0.55, $CHCl_3$). {Lit.[18] $[\alpha]_D^{24}$ = +21.0 (c 1.00, $CHCl_3$), 98% ee}. Chiral HPLC: (AD-H, 1.0 mL/min, *i*-PrOH/hexane = 99/1, λ = 220 nm): t_R (major) = 5.6 min, t_R (minor) = 5.2 min. ^{1}H NMR (400 MHz, $CDCl_3$) δ 7.84 – 7.65 (m, 4H), 7.47 – 7.37 (m, 2H), 7.35 – 7.24 (m, 5H), 7.23 – 7.17 (m, 1H), 4.31 (q, *J* = 7.2 Hz, 1H), 1.73 (d, *J* = 7.2 Hz, 3H). ^{13}C NMR (101 MHz, $CDCl_3$) δ 146.2, 143.8, 133.5, 132.1, 128.4, 127.9, 127.74, 127.71, 127.5, 126.8, 126.1, 125.9, 125.3, 44.8, 21.7. All spectra were in complete agreement with the previously reported values.[18]

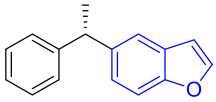

(*S*)-5-(1-phenylethyl)benzofuran (3as): 3as was obtained by following Standard Conditions A using 39.5 mg (0.2 mmol) of **2s**, 4.3 mg (0.0044 mmol) of Ir(dFCF$_3$ppy)$_2$(dtbbpy)Cl, 8.6 mg (0.04 mmol) of NiCl$_2$•DME, 19.4 mg (0.04 mmol) of **L1e**, 12.0 mg (0.05 mmol) of DMBP and 70.0 mg (0.4 mmol) of K$_2$HPO$_4$ to afford 29.8 mg (67% yield) of **3as** in 94:6 er as a colorless oil. IR (neat, cm^{-1}): 2965, 2928, 1601, 1493, 1467. Optical Rotation: $[\alpha]_D^{20}$ = +12.9 (c 1.20, CHCl$_3$). Chiral HPLC: (OJ-H, 1.0 mL/min, *i*-PrOH/hexane = 90/10, λ = 220 nm): t_R (major) = 15.9 min, t_R (minor) = 18.0 min. ^{1}H NMR (400 MHz, CDCl$_3$) δ 7.58 (d, *J* = 2.0 Hz, 1H), 7.45 (d, *J* = 1.2 Hz, 1H), 7.40 (d, *J* = 8.4 Hz, 1H), 7.32 – 7.21 (m, 4H), 7.20 – 7.13 (m, 2H), 6.73 – 6.67 (m, 1H), 4.26 (q, *J* = 7.2 Hz, 1H), 1.68 (d, *J* = 7.2 Hz, 3H). ^{13}C NMR (101 MHz, CDCl$_3$) δ 153.6, 146.7, 145.1, 141.0, 128.3, 127.6, 127.4, 126.0, 124.4, 119.6, 111.1, 106.6, 44.6, 22.3. HRMS (EI, m/z): calcd for C$_{16}$H$_{14}$O (M$^+$) m/z: 222.1045, found: 222.1045.

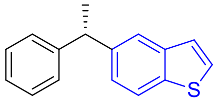

(*S*)-5-(1-phenylethyl)benzo[b]thiophene (3at): 3at was obtained by following Standard Conditions A using 42.5 mg (0.2 mmol) of **2t**, 4.7 mg (0.0044 mmol) of Ir(dFCF$_3$ppy)$_2$(dtbbpy)Cl, 8.3 mg (0.04 mmol) of NiCl$_2$•DME, 19.1 mg (0.04 mmol) of **L1e**, 12.2 mg (0.05 mmol) of DMBP and 70.0 mg (0.4 mmol) of K$_2$HPO$_4$ to afford 29.9 mg (62% yield) of **3at** in 94:6 er as a colorless oil. IR (neat, cm^{-1}): 2962, 2925, 1493, 1454, 1261. Optical Rotation: $[\alpha]_D^{20}$ = +15.7 (c 1.12, CHCl$_3$). Chiral HPLC: (OJ-H, 1.0 mL/min, *i*-PrOH/hexane = 90/10, λ = 220 nm): t_R (major) = 10.1 min, t_R (minor) = 12.3 min. ^{1}H NMR (400 MHz, CDCl$_3$) δ 7.77 (d, *J* = 8.0 Hz, 1H), 7.68 (s, 1H), 7.41 (d, *J* = 5.2, 1H), 7.31 – 7.22 (m, 6H), 7.22 – 7.12 (m, 1H), 4.28 (q, *J* = 7.2 Hz, 1H), 1.70 (d, *J* = 7.2, 3H). ^{13}C NMR (101 MHz, CDCl$_3$) δ 146.4, 142.7, 139.8, 137.5, 128.4, 127.0, 126.5, 126.0, 124.8, 123.8, 122.3, 122.0, 44.6, 22.1. HRMS (EI, m/z): calcd for C$_{16}$H$_{14}$S (M$^+$) m/z: 238.0816, found: 238.0817.

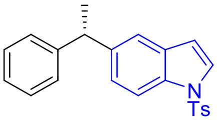

(*S*)-5-(1-phenylethyl)-1-tosyl-*1H*-indole (3au): 3au was obtained by following Standard Conditions A using 70.6 mg (0.2 mmol) of **2u**, 4.3 mg (0.0044 mmol) of Ir(dFCF$_3$ppy)$_2$(dtbbpy)Cl, 9.0 mg (0.04 mmol) of NiCl$_2$•DME, 16.3 mg (0.04 mmol) of **L1e**, 12.1 mg (0.05 mmol) of DMBP and 69.7 mg (0.4 mmol) of K$_2$HPO$_4$ to afford 54.6 mg (72% yield) of **3au** in 94.5:5.5 er as a colorless oil. IR (neat, cm^{-1}): 2957, 2925, 1597, 1459, 1373. Optical Rotation: $[\alpha]_D^{20}$ = -0.5 (c 1.46, CHCl$_3$). Chiral HPLC: (OJ-H, 1.0 mL/min, *i*-PrOH/hexane = 85/15, λ = 220 nm): t_R (major) = 29.4 min, t_R (minor) = 38.1 min. ^{1}H NMR (400 MHz, CDCl$_3$) δ 7.88 – 7.85 (m, 1H), 7.74 (d, *J* = 6.8 Hz, 2H), 7.52 – 7.48 (m, 1H), 7.37 – 7.34 (m, 1H), 7.31 – 7.08 (m, 8H), 6.56 (d, *J* = 2.8 Hz, 1H), 4.19 (q, *J* = 7.2 Hz, 1H), 2.30 (s, 3H), 1.63 (d, *J* = 7.2 Hz, 3H). ^{13}C NMR (101 MHz, CDCl$_3$) δ 146.4, 144.8, 141.6, 135.3, 133.2, 130.8, 129.8, 128.3, 127.6, 126.8, 126.4, 126.0, 124.8, 119.7, 113.3, 108.9, 44.6, 22.1, 21.5. HRMS (EI, m/z): calcd for C$_{23}$H$_{21}$NO$_2$S (M$^+$) m/z: 375.1293, found: 375.1293.

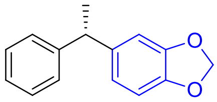

(*S*)-5-(1-phenylethyl)benzo[d][1,3]dioxole (3av): 3av was obtained by following Standard Conditions A using 40.7 mg (0.2 mmol) of **2v**, 4.4 mg (0.0044 mmol) of Ir(dFCF$_3$ppy)$_2$(dtbbpy)Cl, 8.5 mg (0.04 mmol) of NiCl$_2$•DME, 19.4 mg (0.04 mmol) of **L1e**, 12.0 mg (0.05 mmol) of DMBP and 69.0 mg (0.4 mmol) of K$_2$HPO$_4$ to afford 23.0 mg (50% yield) of **3av** in 94.5:5.5 er as a colorless oil. IR (neat, cm^{-1}): 2957, 2925, 1488, 1458, 1377. Optical Rotation: $[\alpha]_D^{20}$ = +8.3 (c 0.66, CHCl$_3$). Chiral HPLC: (OJ-H*2, 1.0 mL/min, *i*-PrOH/hexane = 90/10, λ =

220 nm): t_R (major) = 29.698 min, t_R (minor) = 28.485 min. ^{1}H NMR (400 MHz, $CDCl_3$) δ 7.46 – 7.23 (m, 2H), 7.23 – 7.10 (m, 3H), 6.76 – 6.66 (m, 3H), 5.90 (s, 2H), 4.07 (q, J = 7.2 Hz, 1H), 1.59 (d, J = 7.2 Hz, 3H). ^{13}C NMR (101 MHz, $CDCl_3$) δ 147.6, 146.4, 145.7, 140.5, 128.4, 127.4, 126.0, 120.3, 108.2, 108.0, 100.8, 44.4, 22.0. HRMS (EI, m/z): calcd for $C_{15}H_{14}O_2$ (M^+) m/z: 226.0994, found: 226.0994.

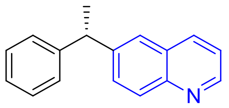

(*S*)-6-(1-phenylethyl)quinoline (3aw): 3aw was obtained by following Standard Conditions A using 42.0 mg (0.2 mmol) of **2w**, 4.7 mg (0.0044 mmol) of Ir(dFCF$_3$ppy)$_2$(dtbbpy)Cl, 8.7 mg (0.04 mmol) of NiCl$_2$•DME, 19.5 mg (0.04 mmol) of **L1e**, 12.4 mg (0.05 mmol) of DMBP and 69.6 mg (0.4 mmol) of K_2HPO_4 to afford 37.2 mg (79% yield) of **3aw** in 85:15 er as a colorless oil. IR (neat, cm^{-1}): 3059, 2965, 2926, 1721. 1596, 1496. Optical Rotation: $[\alpha]_D^{20}$ = +31.9 (c 0.53, $CHCl_3$, 67% ee). Chiral HPLC: (OJ-H, 1.0 mL/min, *i*-PrOH/hexane = 80/20, λ = 220 nm): t_R (major) = 10.0 min, t_R (minor) = 11.9 min. ^{1}H NMR (400 MHz, $CDCl_3$) δ 8.86 (dd, J = 4.4, 1.6 Hz, 1H), 8.15 – 8.07 (m, 1H), 8.00 (d, J = 8.8 Hz, 1H), 7.65 (d, J = 2.0 Hz, 1H), 7.56 (dd, J = 8.8, 2.0 Hz, 1H), 7.36 (dd, J = 8.4 Hz, 4.4 Hz, 1H), 7.34 – 7.28 (m, 2H), 7.27 – 7.24 (m, 2H), 7.24 – 7.18 (m, 1H), 4.35 (q, J = 7.2 Hz, 1H), 1.75 (d, J = 7.2 Hz, 3H). ^{13}C NMR (101 MHz, $CDCl_3$) δ 149.8, 147.2, 145.7, 144.6, 135.8, 130.4, 129.4, 128.5, 128.2, 127.7, 126.3, 125.1, 121.1, 44.7, 21.7. HRMS (EI, m/z): calcd for $C_{17}H_{15}N$ (M^+) m/z: 233.1204, found: 233.1205.

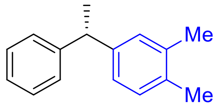

(*S*)-1,2-dimethyl-4-(1-phenylethyl)benzene (3ax): 3ax was obtained by following Standard Conditions A using 37.3 mg (0.2 mmol) of **2x**, 4.5 mg (0.0044 mmol) of Ir(dFCF$_3$ppy)$_2$(dtbbpy)Cl, 8.9 mg (0.04 mmol) of NiCl$_2$•DME, 19.2 mg (0.04 mmol) of **L1e**, 12.1 mg (0.05 mmol) of DMBP and 69.6 mg (0.4 mmol) of K_2HPO_4 to afford 29.2 mg (67% yield) of **3ax** (with 2.5% homocoupling product) in 96:4 er as a colorless oil. IR (neat, cm^{-1}): 2967, 2928, 1497, 1451, 1374. Optical Rotation: $[\alpha]_D^{20}$ = +1.2 (c 1.06, $CHCl_3$). Chiral HPLC: (OD-H, 1.0 mL/min, *i*-PrOH/hexane = 99.9/0.1, λ = 220 nm): t_R (major) = 13.4 min, t_R (minor) = 12.6 min. ^{1}H NMR (400 MHz, $CDCl_3$) δ 7.35 – 7.10 (M, 5H), 7.09 – 6.83 (m, 3H), 4.08 (q, J = 7.2 Hz, 1H), 2.21 (s, 6H), 1.61 (d, J = 7.2 Hz, 3H). ^{13}C NMR (101 MHz, $CDCl_3$) δ 146.7, 143.9, 136.4, 134.1, 129.6, 129.0, 128.3, 127.5, 125.9, 124.9, 44.4, 21.9, 19.8, 19.3. HRMS (EI, m/z): calcd for $C_{16}H_{18}$ (M^+) m/z: 210.1409, found: 210.1407.

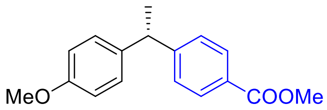

(*R*)-methyl 4-(1-(4-methoxyphenyl)ethyl)benzoate (3ba): 3ba was obtained by following Standard Conditions B using 109.1 mg (0.8 mmol) of **1b**, 42.6 mg (0.2 mmol) of **2a**, 4.5 mg (0.0044 mmol) of Ir(dFCF$_3$ppy)$_2$(dtbbpy)Cl, 8.6 mg (0.04 mmol) of NiCl$_2$•DME, 19.8 mg (0.04 mmol) of **L1e**, 12.5 mg (0.05 mmol) of DMBP and 69.8 mg (0.4 mmol) of K_2HPO_4 to afford 35.2 mg (66% yield) of **3ba** in 89:11 er as a colorless oil. IR (neat, cm^{-1}): 2963, 1722, 1612, 1512, 1281. Optical Rotation: $[\alpha]_D^{20}$ = -1.1 (c 1.31, $CHCl_3$). Chiral HPLC: (OJ-H, 0.8 mL/min, *i*-PrOH/hexane = 95/5, λ = 220 nm): t_R (major) = 20.2 min, t_R (minor) = 19.3 min. ^{1}H NMR (400 MHz, $CDCl_3$) δ 7.94 (d, J = 7.6 Hz, 2H), 7.27 (d, J = 8.4 Hz, 2H), 7.11 (d, J = 8.4 Hz, 2H), 6.83 (d, J = 8.4 Hz, 2H), 4.15 (q, J = 7.2 Hz, 1H), 3.89 (s, 3H), 3.78 (s, 3H), 1.62 (d, J = 7.2 Hz, 3H). ^{13}C NMR (101 MHz, $CDCl_3$) δ 167.1, 158.0, 152.1, 137.6, 129.7, 128.5, 127.9, 127.5, 113.8, 55.2, 52.0, 44.0, 21.8. HRMS (EI, m/z): calcd for $C_{17}H_{18}O_3$ (M^+) m/z: 270.1256, found: 270.1257.

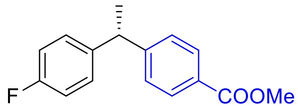

(*R*)-methyl 4-(1-(4-fluorophenyl)ethyl)benzoate (3ca): 3ca was obtained

by following Standard Conditions B using 99.1 mg (0.8 mmol) of **1c**, 43.4 mg (0.2 mmol) of **2a**, 4.6 mg (0.0044 mmol) of Ir(dFCF$_3$ppy)$_2$(dtbbpy)Cl, 8.7 mg (0.04 mmol) of $NiCl_2$•DME, 19.5 mg (0.04 mmol) of **L1e**, 12.4 mg (0.05 mmol) of DMBP and 70.3 mg (0.4 mmol) of K_2HPO_4 to afford 29.1 mg (56% yield) of **3ca** in 86:14 er as a colorless oil. IR (neat, cm^{-1}): 2967, 1723, 1610, 1510, 1281. Optical Rotation: $[\alpha]_D^{20}$ = +5.5 (c 0.69, $CHCl_3$). Chiral HPLC: (OJ-H, 1.0 mL/min, *i*-PrOH/hexane = 90/10, λ = 220 nm): t_R (major) = 8.3 min, t_R (minor) = 9.3 min. ^{1}H NMR (400 MHz, $CDCl_3$) δ 7.95 (d, *J* = 8.0 Hz, 2H), 7.26 (d, *J* = 8.0 Hz, 2H), 7.15 (dd, *J* = 8.4, 5.6 Hz, 2H), 6.97 (t, *J* = 8.4 Hz, 2H), 4.18 (q, *J* = 7.2 Hz, 1H), 3.89 (s, 3H), 1.63 (d, *J* = 7.2 Hz, 3H). ^{19}F NMR (376 MHz, $CDCl_3$) δ -116.9. ^{13}C NMR (101 MHz, $CDCl_3$) δ 167.0, 161.4 (d, *J* = 245.5 Hz), 151.4, 141.1 (d, *J* = 3.0 Hz), 129.8, 129.0 (d, *J* = 7.7 Hz), 128.1, 127.5, 115.2 (d, *J* = 21.4 Hz) 52.0, 44.0, 21.7. HRMS (EI, m/z): calcd for $C_{16}H_{15}FO_2$ (M^+) m/z: 258.1056, found: 258.1056.

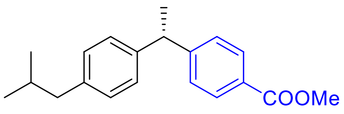

(*S*)-methyl 4-(1-(4-isobutylphenyl)ethyl)benzoate (3da): 3da was obtained by following Standard Conditions B using 129.9 mg (0.8 mmol) of **1d**, 43.2 mg (0.2 mmol) of **2a**, 4.7 mg (0.0044 mmol) of Ir(dFCF$_3$ppy)$_2$(dtbbpy)Cl, 8.6 mg (0.04 mmol) of $NiCl_2$•DME, 19.5 mg (0.04 mmol) of **L1e**, 12.3 mg (0.05 mmol) of DMBP and 69.4 mg (0.4 mmol) of K_2HPO_4 to afford 20.7 mg (35% yield) of **3da** in 94.5:5.5 er as a colorless oil with 43% recovery of **1d**. IR (neat, cm^{-1}): 2955, 2927, 1724, 1609, 1435. Optical Rotation: $[\alpha]_D^{20}$ = +0.9 (c 0.62, $CHCl_3$). Chiral HPLC: (AS-H*2, 1.0 mL/min, *i*-PrOH/hexane = 98/2, λ = 220 nm): t_R (major) = 9.0 min, t_R (minor) = 8.5 min. ^{1}H NMR (400 MHz, $CDCl_3$) δ 7.95 (d, *J* = 8.0 Hz, 2H), 7.28 (d, *J* = 8.4 Hz, 2H), 7.16 – 7.00 (m, 4H), 4.17 (q, *J* = 7.2 Hz, 1H), 3.89 (s, 3H), 2.42 (d, *J* = 6.8 Hz, 2H), 1.86 – 1.78 (m, 1H), 1.63 (d, *J* = 7.2 Hz, 3H), 0.88 (d, *J* = 6.8 Hz, 6H). ^{13}C NMR (101 MHz, $CDCl_3$) δ 167.1, 152.0, 142.6, 139.6, 129.7, 129.2, 127.8, 127.6, 127.2, 52.0, 45.0, 44.4, 30.2, 22.4, 21.6. HRMS (ESI, m/z): calcd for $C_{20}H_{25}O_2$ ($M+H^+$) m/z: 297.1855, found: 297.1853.

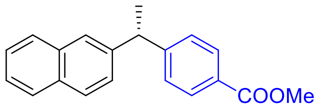

(*R*)-methyl 4-(1-(naphthalen-2-yl)ethyl)benzoate (3ea): 3ea was obtained by following Standard Conditions B using 124.8 mg (0.8 mmol) of **1e**, 43.8 mg (0.2 mmol) of **2a**, 4.7 mg (0.0044 mmol) of Ir(dFCF$_3$ppy)$_2$(dtbbpy)Cl, 8.7 mg (0.04 mmol) of $NiCl_2$•DME, 19.5 mg (0.04 mmol) of **L1e**, 11.7 mg (0.05 mmol) of DMBP and 68.8 mg (0.4 mmol) of K_2HPO_4 to afford 21.1 mg (36% yield) of **3ea** in 90.5:9.5 er as a colorless oil with 66% recovery of **1e**. IR (neat, cm^{-1}): 2927, 1721, 1609, 1435, 1279. Optical Rotation: $[\alpha]_D^{20}$ = -29.2 (c 1.08, $CHCl_3$). Chiral HPLC: (OD-H*2, 1.0 mL/min, *i*-PrOH/hexane = 90/10, λ = 220 nm): t_R (major) = 14.8 min, t_R (minor) = 13.5 min. ^{1}H NMR (400 MHz, $CDCl_3$) δ 7.96 (d, *J* = 8.4 Hz, 2H), 7.79 (d, *J* = 7.6 Hz, 2H), 7.74 (d, *J* = 8.4 Hz, 1H), 7.68 (s, 1H), 7.52 – 7.38 (m, 2H), 7.32 (d, *J* = 8.4 Hz, 2H), 7.28 – 7.23 (m, 1H), 4.36 (q, *J* = 7.2 Hz, 1H), 3.88 (s, 3H), 1.74 (d, *J* = 7.2 Hz, 3H). ^{13}C NMR (101 MHz, $CDCl_3$) δ 167.0, 151.5, 142.8, 133.5, 132.2, 129.8, 128.13, 128.09, 127.8, 127.7, 127.6, 126.6, 126.1, 125.6, 125.5, 52.0, 44.9, 21.5. HRMS (EI, m/z): calcd for $C_{20}H_{18}O_2$ (M^+) m/z: 290.1307, found: 290.1306.

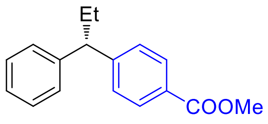

(*S*)-methyl 4-(1-phenylpropyl)benzoate (3fa): 3fa was obtained by following Standard Conditions B using 96.0 mg (0.8 mmol) of **1f**, 43.6 mg (0.2 mmol) of **2a**, 4.2 mg (0.0044 mmol) of Ir(dFCF$_3$ppy)$_2$(dtbbpy)Cl, 8.8 mg (0.04 mmol) of $NiCl_2$•DME, 19.5 mg (0.04 mmol) of **L1e**, 11.9 mg (0.05 mmol) of DMBP and 69.9 mg (0.4 mmol) of K_2HPO_4 to afford 23.1 mg (45% yield) of **3fa** in 90:10 er as a colorless oil. IR (neat, cm^{-1}): 2960, 2930,

1720, 1610, 1435. Optical Rotation: $[\alpha]_D^{20}$ = +2.7 (c 0.85, $CHCl_3$). Chiral HPLC: (OD-H, 1.0 mL/min, *i*-PrOH/hexane = 98/2, λ = 220 nm): t_R (major) = 6.6 min, t_R (minor) = 7.0 min. ^{1}H NMR (400 MHz, $CDCl_3$) δ 7.94 (d, *J* = 8.0 Hz, 2H), 7.35 – 7.24 (m, 4H), 7.23 – 7.14 (m, 3H), 3.88 (s, 3H), 3.84 (t, *J* = 7.6 Hz, 1H), 2.13 – 2.00 (m, 2H), 0.90 (t, *J* = 7.2 Hz, 3H). ^{13}C NMR (101 MHz, $CDCl_3$) δ 167.0, 150.5, 144.2, 129.7, 128.5, 128.0, 127.93, 127.86, 126.3, 53.2, 51.9, 28.3, 12.6. HRMS (EI, m/z): calcd for $C_{17}H_{18}O_2$ (M^+) m/z: 254.1307, found: 254.1310.

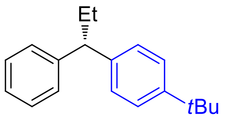

(*S*)-1-(*tert*-butyl)-4-(1-phenylpropyl)benzene 3fd: 3fd was obtained by following Standard Conditions B using 96.7 mg (0.8 mmol) of **1f**, 43.4 mg (0.2 mmol) of **2d**, 4.6 mg (0.0044 mmol) of Ir(dFCF$_3$ppy)$_2$(dtbbpy)Cl, 8.8 mg (0.04 mmol) of $NiCl_2$•DME, 19.7 mg (0.04 mmol) of **L1e**, 12.0 mg (0.05 mmol) of DMBP and 69.3 mg (0.4 mmol) of K_2HPO_4 to afford 19.6 mg (38% yield) of **3fd** in 90:10 er as a colorless oil. IR (neat, cm^{-1}): 2961, 2871, 1600, 1493, 1454. Optical Rotation: $[\alpha]_D^{20}$ = +6.4 (c 0.89, $CHCl_3$). Chiral HPLC: (OD-H*2, 1.0 mL/min, 100% hexane, λ = 220 nm): t_R (major) = 17.0 min, t_R (minor) = 16.4 min. ^{1}H NMR (400 MHz, $CDCl_3$) δ 7.29 – 7.22 (m, 6H), 7.17 – 7.14 (m, 3H), 3.75 (t, *J* = 8.0 Hz, 1H), 2.10 – 2.02 (m, 2H), 1.28 (s, 9H), 0.89 (t, *J* = 7.2 Hz, 3H), ^{13}C NMR (101 MHz, $CDCl_3$) δ 148.4, 145.3, 142.1, 128.3, 128.0, 127.4, 125.9, 125.2, 52.8, 34.3, 31.4, 28.7, 12.8. HRMS (ESI, m/z): calcd for $C_{19}H_{24}Na$ (M+Na$^+$) m/z: 275.1776, found: 275.1782.

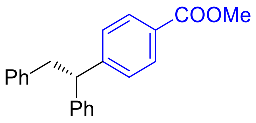

(*S*)-methyl 4-(1,2-diphenylethyl)benzoate (3ga): 3ga was obtained by following Standard Conditions B using 146.5 mg (0.8 mmol) of **1g**, 44.0 mg (0.2 mmol) of **2a**, 4.3 mg (0.0044 mmol) of Ir(dFCF$_3$ppy)$_2$(dtbbpy)Cl, 8.7 mg (0.04 mmol) of $NiCl_2$•DME, 19.5 mg (0.04 mmol) of **L1e**, 12.5 mg (0.05 mmol) of DMBP and 69.5 mg (0.4 mmol) of K_2HPO_4 to afford 27.4 mg (42% yield) of **3ga** in 94.5:5.5 er as a colorless oil with 85% recovery of **1g**. IR (neat, cm^{-1}): 3027, 2949, 1720, 1608, 1278. Optical Rotation: $[\alpha]_D^{20}$ = -33.2 (c 0.54, $CHCl_3$). Chiral HPLC: (OD-H*2, 1.0 mL/min, *i*-PrOH/hexane = 95/5, λ = 220 nm): t_R (major) = 18.0 min, t_R (minor) = 17.4 min. ^{1}H NMR (400 MHz, $CDCl_3$) δ 7.90 (d, *J* = 8.4 Hz, 2H), 7.31 – 7.08 (m, 10H), 6.98 (d, *J* = 6.8 Hz, 2H), 4.28 (t, *J* = 7.6 Hz, 1H), 3.87 (s, 3H), 3.45 – 3.28 (m, 2H). ^{13}C NMR (101 MHz, $CDCl_3$) δ 167.0, 149.7, 143.6, 139.7, 129.7, 129.0, 128.5, 128.14, 128.12, 128.0, 126.5, 126.0, 53.1, 52.0, 41.8. HRMS (EI, m/z): calcd for $C_{22}H_{20}O_2$ (M^+) m/z: 316.1463, found: 316.1461.

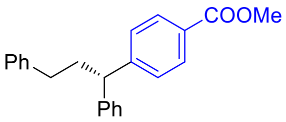

(*S*)-methyl 4-(1,3-diphenylpropyl)benzoate (3ha): 3ha was obtained by following Standard Conditions B using 78.3 mg (0.4 mmol) of **1h**, 42.7 mg (0.2 mmol) of **2a**, 4.2 mg (0.0044 mmol) of Ir(dFCF$_3$ppy)$_2$(dtbbpy)Cl, 8.7 mg (0.04 mmol) of $NiCl_2$•DME, 19.6 mg (0.04 mmol) of **L1e**, 12.3 mg (0.05 mmol) of DMBP and 69.5 mg (0.4 mmol) of K_2HPO_4 to afford 30.3 mg (46% yield) of **3ha** in 93.5:6.5 er as a colorless oil with 72% recovery of **1h**. IR (neat, cm^{-1}): 2947, 1722, 1609, 1495, 1280. Optical Rotation: $[\alpha]_D^{20}$ = -6.1 (c 0.40, $CHCl_3$). Chiral HPLC: (OD-H, 1.0 mL/min, *i*-PrOH/hexane = 90/10, λ = 220 nm): t_R (major) = 6.9 min, t_R (minor) = 8.4 min. ^{1}H NMR (400 MHz, $CDCl_3$) δ 7.96 (d, *J* = 8.4 Hz, 2H), 7.37 – 7.16 (m, 10H), 7.12 (d, *J* = 6.8 Hz, 2H), 3.97 (t, *J* = 7.6 Hz, 1H), 3.89 (s, 3H), 2.63 – 2.52 (m, 2H), 2.44 – 2.35 (m, 2H). ^{13}C NMR (101 MHz, $CDCl_3$) δ 167.0, 150.2, 143.8, 141.7, 129.8, 128.6, 128.40, 128.39, 128.2, 127.93, 127.86, 126.5, 125.9, 52.0, 50.6, 37.0, 33.9. HRMS (EI, m/z): calcd for $C_{23}H_{22}O_2$ (M^+) m/z: 330.1620, found: 330.1618.

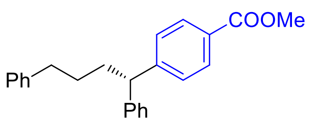

(*S*)-methyl 4-(1,4-diphenylbutyl)benzoate (3ia): 3ia was obtained by following Standard Conditions B using 167.5 mg (0.8 mmol) of **1i**, 43.6 mg (0.2 mmol) of **2a**, 4.7 mg (0.0044 mmol) of Ir(dFCF$_3$ppy)$_2$(dtbbpy)Cl, 8.8 mg (0.04 mmol) of NiCl$_2$•DME, 19.5 mg (0.04 mmol) of **L1e**, 12.3 mg (0.05 mmol) of DMBP and 70.5 mg (0.4 mmol) of K$_2$HPO$_4$ to afford 29.7 mg (43% yield) of **3ia** in 92:8 er as a colorless oil with 86% recovery of **1i**. IR (neat, cm^{-1}): 2927, 1722, 1610, 1454, 1280. Optical Rotation: $[\alpha]_D^{20}$ = -1.0 (c 1.27, CHCl$_3$). Chiral HPLC: (OD-H, 1.0 mL/min, *i*-PrOH/hexane = 95/5, λ = 220 nm): t_R (major) = 7.0 min, t_R (minor) = 7.4 min. ^{1}H NMR (400 MHz, CDCl$_3$) δ 7.93 (d, *J* = 8.4 Hz, 2H), 7.32 – 7.14 (m, 10H), 7.11 (d, *J* = 7.2 Hz, 2H), 3.95 (t, *J* = 7.6 Hz, 1H), 3.87 (s, 3H), 2.63 (t, *J* = 7.6 Hz, 2H), 2.15 – 2.02 (m, 2H), 1.79 – 1.47 (m, 2H). ^{13}C NMR (101 MHz, CDCl$_3$) δ 167.0, 150.4, 144.1, 142.1, 129.8, 128.5, 128.4, 128.3, 128.1, 127.9, 127.8, 126.4, 125.8, 52.0, 51.3, 35.8, 34.9, 29.6. HRMS (EI, m/z): calcd for C$_{24}$H$_{24}$O$_2$ (M$^+$) m/z: 344.1776, found: 344.1775.

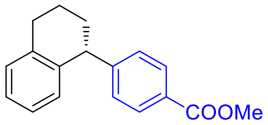

(*R*)-methyl 4-(1,2,3,4-tetrahydronaphthalen-1-yl)benzoate (3ja): 3ja was obtained by following Standard Conditions B using 106.1 mg (0.8 mmol) of **1j**, 42.8 mg (0.2 mmol) of **2a**, 4.5 mg (0.0044 mmol) of Ir(dFCF$_3$ppy)$_2$(dtbbpy)Cl, 8.6 mg (0.04 mmol) of NiCl$_2$•DME, 19.8 mg (0.04 mmol) of **L1e**, 12.0 mg (0.05 mmol) of DMBP and 69.0 mg (0.4 mmol) of K$_2$HPO$_4$ to afford 19.5 mg (37% yield) of **3ja** in 90:10 er as a colorless oil with 79% recovery of **1j** determined by ^{1}H NMR using TMSPh as an internal standard. IR (neat, cm^{-1}): 2929, 1722, 1610, 1435, 1279. Optical Rotation: $[\alpha]_D^{20}$ = -24.1 (c 0.42, CHCl$_3$). Chiral HPLC: (OD-H, 1.0 mL/min, *i*-PrOH/hexane = 90/10, λ = 220 nm): t_R (major) = 5.2 min, t_R (minor) = 4.7 min. ^{1}H NMR (400 MHz, CDCl$_3$) δ 7.95 (d, *J* = 8.4 Hz, 2H), 7.22 – 7.11 (m, 4H), 7.09 – 6.98 (m, 1H), 6.78 (d, *J* = 7.6 Hz, 1H), 4.18 (t, *J* = 6.4 Hz, 1H), 3.90 (s, 3H), 2.97 – 2.71 (m, 2H), 2.23 – 2.11 (m, 1H), 1.94 – 1.71 (m, 3H). ^{13}C NMR (101 MHz, CDCl$_3$) δ 167.1, 153.0, 138.5, 137.6, 130.1, 129.6, 129.1, 128.8, 128.0, 126.2, 125.8, 52.0, 45.7, 33.1, 29.7, 20.9. HRMS (EI, m/z): calcd for C$_{18}$H$_{18}$O$_2$ (M$^+$) m/z: 266.1307, found: 266.1308.

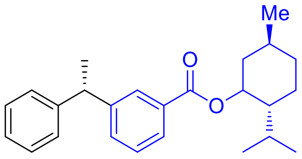

(2*R*,5*S*)-2-isopropyl-5-methylcyclohexyl 3-((*S*)-1-phenylethyl)-benzoate (4): 4 was obtained by following Standard Conditions A using 67.1 mg (0.2 mmol) of **S4**, 4.4 mg (0.0044 mmol) of Ir(dFCF$_3$ppy)$_2$(dtbbpy)Cl, 8.8 mg (0.04 mmol) of NiCl$_2$•DME, 19.5 mg (0.04 mmol) of **L1e**, 11.7 mg (0.05 mmol) of DMBP and 69.9 mg (0.4 mmol) of K$_2$HPO$_4$ to afford 44.7 mg (61% yield) of **4** in 93.5:6.5 er as a colorless oil. IR (neat, cm^{-1}): 3028, 2956, 2870, 1714, 1653, 1274. Optical Rotation: $[\alpha]_D^{20}$ = -63.0 (c 2.52, CHCl$_3$). Chiral HPLC: (OJ-H*2, 1.0 mL/min, *i*-PrOH/hexane = 99.5/0.5, λ = 220 nm): t_R (major) = 9.5 min, t_R (minor) = 8.6 min. ^{1}H NMR (400 MHz, CDCl$_3$) δ 7.95 (s, 1H), 7.86 (d, *J* = 7.6Hz, 1H), 7.40 – 7.10 (m, 7H), 4.91 (td, *J* = 10.8 Hz, 4.4 Hz, 1H), 4.21 (q, *J* = 7.2 Hz, 1H), 2.15 – 2.10 (m, 1H), 2.00 – 1.89 (m, 1H), 1.77 – 1.68 (m, 2H), 1.66 (d, *J* = 7.2 Hz, 3H), 1.62 – 1.59 (m, 2H), 1.20 – 1.13 (m, 2H), 1.00 – 0.85 (m, 7H), 0.78 (d, *J* = 7.2 Hz, 3H). ^{13}C NMR (101 MHz, CDCl$_3$) δ 157.9, 153.0, 142.5, 138.3, 136.2, 128.4, 113.7, 104.6, 60.8, 56.0, 55.2, 44.2, 22.2. HRMS (EI, m/z): calcd for C$_{25}$H$_{32}$O$_2$ (M$^+$) m/z: 364.2402, found: 364.2401.

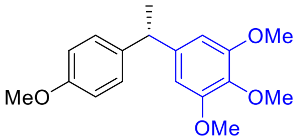

(*S*)-1,2,3-trimethoxy-5-(1-(4-methoxyphenyl)ethyl)benzene (5): 5 was obtained by following Standard Conditions A using 1 mL of **1b**, 0.0498 mg (0.8 mmol) of 5-bromo-1,2,3-trimethoxybenzene **S5**, 4.4 mg (0.0044 mmol)

of Ir(dFCF$_3$ppy)$_2$(dtbbpy)Cl, 8.7 mg (0.04 mmol) of NiCl$_2$•DME, 19.6 mg (0.04 mmol) of **L1e**, 11.8 mg (0.05 mmol) of DMBP and 69.2 mg (0.4 mmol) of K_2HPO_4. Upon completion, the reaction was quenched by adding Et_2O, filtered through a short pad of silica and eluted with Et_2O. The organic layer was evaporated and 5 mL of methanol and 7.5 mg (0.2 mmol) of $NaBH_4$ was added to the residue and stirred for 5 h to reduce DMBP. The reaction was concentrated under *vacuo* and purified by flash column chromatography to afford 13.8 mg (23% yield) of **5** in 92.5:7.5 er as a colorless oil. IR (neat, cm^{-1}): 2934, 2836, 1590, 1511, 1461. Optical Rotation: $[\alpha]_D^{20}$ = +10.0 (c 0.15, $CHCl_3$). Chiral HPLC: (AS-H*2, 1.0 mL/min, *i*-PrOH/hexane = 98/2, λ = 220 nm): t_R (major) = 26.0 min, t_R (minor) = 24.5 min. ^{1}H NMR (400 MHz, $CDCl_3$) δ 7.14 (d, *J* = 8.8 Hz, 2H), 6.84 (d, *J* = 8.4 Hz, 2H), 6.42 (s, 2H), 4.04 (q, *J* = 7.6 Hz, 1H), 3.82 (s, 3H), 3.81 (s, 6H), 3.79 (s, 3H), 1.59 (d, *J* = 7.2 Hz, 3H). ^{13}C NMR (101 MHz, $CDCl_3$) δ 157.9, 153.0, 142.5, 138.3, 136.2, 128.4, 113.7, 104.6, 60.8, 56.0, 55.2, 44.2, 22.2. HRMS (ESI, m/z): calcd for (M+H$^+$) $C_{18}H_{23}O_4$ m/z: 303.1596, found: 303.1597.

Supplementary Discussion

Control experiments

Several mechanistic experiments were carried out to investigate the catalytic circle. As shown in **Supplementary Table 3**, control experiments revealed that without photocatalyst, nickel catalyst and light, this reaction could not be initiated with no **3aa** produced and 70% to 91% recovery of **2a**. The result in entry 5 and entry 6 showed that bromine free radical might be hydrogen atom abstractor and DMBP might be a co-catalyst in HAT process.

Supplementary Table 3. Control experiments.

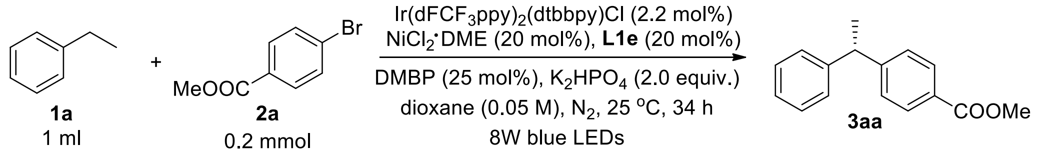

Entry	Variations	Yield of 3aa	Recovery of 2a
**1**	w/o P.C.	0	70
**2**	w/o Ni/Ln	0	88
**3**	w/o light	0	91
**4**	UV light (365 nm), w/o P.C.	0	91
**5**[a]	w/o DMBP	46	18
**6**[b]	w/o DMBP	35	53
**7**	4 mL **1a** as solvent	49[c]	0
**8**	**2a-Cl** instead of **2a**	0	66
**9**	**2a-Cl** instead of **2a**, 1.0 eq. of KBr	58	24
**10**	**2a-I** instead of **2a**	0	83

[a] Under Standard Conditions A. [b] Under Standard Conditions B. [c]52% yield homocoupling byproduct of **1a**

Kinetic experiments

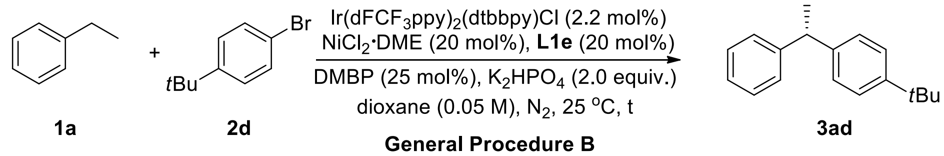

1a **2d** **3ad**

Supplementary Figure 145. Kinetic experiments

For the reaction of ethylbenzene **1a** (0.05 M) and arylbromide **2d** (0.05 M): To a 20 mL vial with a stir bar was added **L1e** (0.0195 g, 0.04 mmol, 20 mol%), $NiCl_2$•DME (0.0087 g, 0.04 mmol, 20 mol%) and 1 mL of dioxane in a N_2-filled glovebox. Reaction was stirred at 50 °C for 30 minutes before cooled to room temperature. 3 mL of dioxane, **1a** (0.0212 g, 0.2 mmol, 1.0 equiv.), **2d** (0.0427 g, 0.2 mmol, 1.0 equiv.), Ir(dFCF$_3$ppy)$_2$(dtbbpy)Cl (0.0044 g, 0.0044 mmol, 2.2 mol%), DMBP (0.0121 g, 0.05 mmol, 25 mol%) and K_2HPO_4 (0.0696 g, 0.4 mmol, 2.0 equiv.) was added consistently. Undecane (42 μL, 0.2 mmol, 1.0 equiv.) was added as an internal standard. The reaction was stirred at 600 rpm under the irradiation of 8W blue LEDs in a distance of 5 cm at room temperature (25 °C) in glove box. 100 μL of the reaction mixture was taken out at 4, 10, 16, 22, 35 h and quenched with 1 mL of *n*-hexane. The yield of **3ad** was determined by GC with undecane as an internal standard.

For the reaction of ethylbenzene **1a** (0.10 M), arylbromide **2d** (0.05 M): The procedure for this reaction was the same as above but instead of **1a** (0.0212 g, 0.05 M), **1a** (0.0427 g, 0.10 M) was added in the reaction.

For the reaction of ethylbenzene **1a** (0.15 M), arylbromide **2d** (0.05 M): The procedure for this reaction was the same as above but instead of **1a** (0.0212 g, 0.05 M), **1a** (0.0640 g, 0.15 M) was added in the reaction.

For the reaction of ethylbenzene **1a** (0.20 M), arylbromide **2d** (0.05 M): The procedure for this reaction was the same as above but instead of **1a** (0.0212 g, 0.05 M), **1a** (0.0851 g, 0.20 M) was added in the reaction.

The molar concentrations of the product **3ad** were calculated by integrating against undecane as an internal standard. The molar concentration of product **3ad** was plotted against the reaction time and the slope of linear portion of the curve was used to determine the initial rates of the reaction. The table showing molar concentration of product **3ad** in different concentration of **1a** and *k* value, graph showing the rate at different concentration of **1a** and graph showing *k* vesus [**1a**] are shown below.

Supplementary Table 4. The molar concentration of **3ad** and *k* value with concentration of **1a**

Time (h)	A (0.05 M of **1a**)	B (0.10 M of **1a**)	C (0.15 M of **1a**)	D (0.20 M of **1a**)
4	0	0.000266	0.000246	0.000097
10	0.000380	0.001147	0.001379	0.000729
16	0.000534	0.002104	0.002511	0.001872
22	0.000757	0.002686	0.003465	0.003891
35	0.002535	0.004731	0.006068	0.007752
k (M/h)	7.852E-05	1.42142E-04	1.8639 E-04	2.5567 E-04

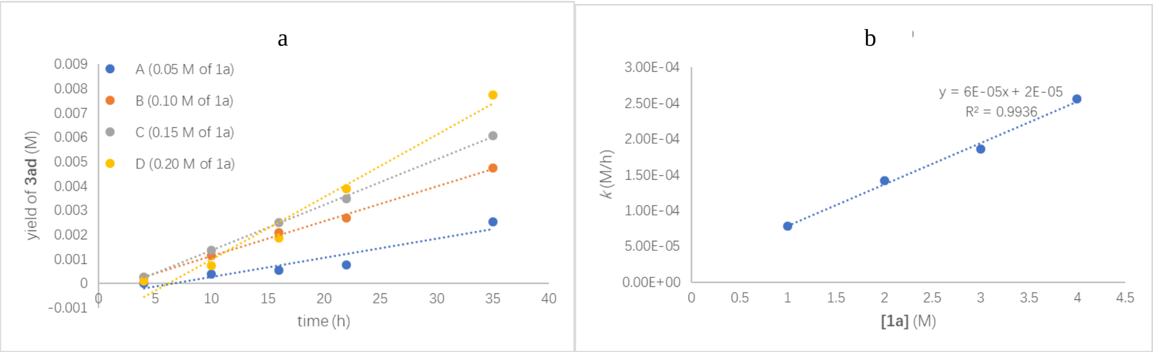

Supplementary Figure 146. (a) Plot of the rise of product **3ad** with 0.05 M. 0.10 M, 0.15 M and 0.20 M of **1a** in different time interval. (b) The curve of initial rate with concentration of **1a**.

For the reaction of ethylbenzene **1a** (0.05 M) and arylbromide **2d** (0.05 M): To a 20 mL vial with a stir bar was added **L1e** (0.0195 g, 0.04 mmol, 20 mol%), $NiCl_2$•DME (0.0087 g, 0.04 mmol, 20 mol%) and 1 mL of dioxane in a N_2-filled glovebox. Reaction was stirred at 50 °C for 30 minutes before cooled to room temperature. 3 mL of dioxane, **1a** (0.0212 g, 0.2 mmol, 1.0 equiv.), **2d** (0.0427 g, 33 µL, 0.2 mmol, 1.0 equiv.), Ir(dFCF$_3$ppy)$_2$(dtbbpy)Cl (0.0044 g, 0.0044 mmol, 2.2 mol%), DMBP (0.0121 g, 0.05 mmol, 25 mol%) and K_2HPO_4 (0.0696 g, 0.4 mmol, 2.0 equiv.) was added consistently. Undecane (42 µL, 0.2 mmol, 1.0 equiv.) was added as an internal standard. The reaction was stirred at 600 rpm under the irradiation of 8W blue LEDs in a distance of 5 cm at room temperature (25 °C) in glove box. 100 µL of the reaction mixture was taken out at 12, 17, 22, 35, 48 h and quenched with 1 mL of *n*-hexane. The yield of **3ae** was determined by GC with undecane as an internal standard.

For the reaction of ethylbenzene **1a** (0.05 M), arylbromide **2d** (0.10 M): The procedure for this reaction was the same as above but instead of **2d** (33 µL, 0.05 M), **2d** (66 µL, 0.10 M) was added in the reaction.

For the reaction of ethylbenzene **1a** (0.05 M), arylbromide **2d** (0.15 M): The procedure for this reaction was the same as above but instead of **2d** (33 µL, 0.05 M), **2d** (99 µL, 0.15 M) was added in the reaction.

For the reaction of ethylbenzene **1a** (0.05 M), arylbromide **2d** (0.20 M): The procedure for this reaction was the same as above but instead of **2d** (33 µL, 0.05 M), **2d** (132 µL, 0.10 M) was added in the reaction.

The molar concentrations of the product **3ad** were calculated by integrating against undecane as an internal standard. The molar concentration of product **3ad** was plotted against the reaction time and the slope of linear portion of the curve was used to determine the initial rates of the reaction. The table showing molar concentration of product **3ad** in different concentration of **2d** and *k* value, graph showing the rate at different concentration of **2d** and graph showing *k* vesus [**2d**] are shown below.

Supplementary Table 5. The yield of **3ad** with concentration of **2d**

Time (h)	A (0.05 M of **2d**)	B (0.10 M of **2d**)	C (0.15 M of **2d**)	D (0.20 M of **2d**)
12	0.000312	0.000512	0.000597	0.000518
17	0.000551	0.000785	0.000772	0.000655
22	0.000688	0.000953	0.000941	0.000881
35	0.001030	0.001419	0.001420	0.001337
48	0.001345	0.002052	0.001897	0.001842
k (M/h)	2.766E-05	4.129E-05	3.620E-05	3.708E-05

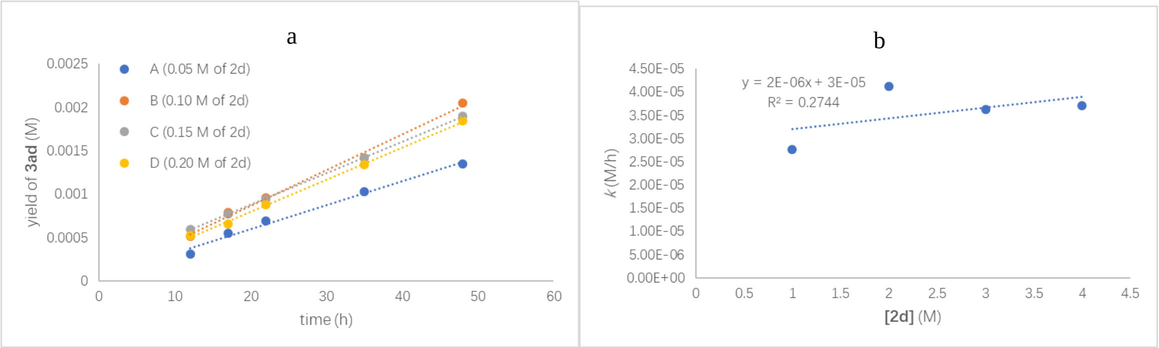

Supplementary Figure 147 (a) Plot of the rise of product **3ad** with 0.05 M. 0.10 M, 0.15 M and 0.20 M of **2d** in different time interval. (b) The curve of initial rate with concentration of **2d**.

Radical-clock Experiment

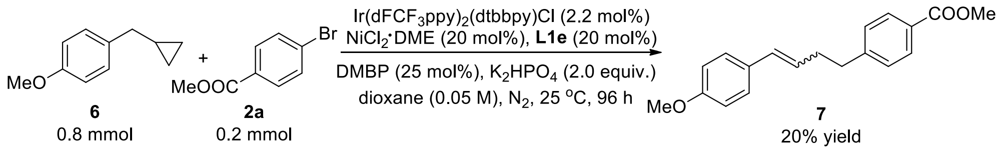

Supplementary Figure 148. Radical-clock Experiment.

Methyl 4-(4-(4-methoxyphenyl)but-3-en-1-yl)benzoate (**7**): **7** was obtained by following Standard Conditions B using 129.0 mg (0.8 mmol) of **6**[19], 44.0 mg (0.2 mmol) of **2a**, 4.4 mg (0.0044 mmol) of Ir(dFCF$_3$ppy)$_2$(dtbbpy)Cl, 8.3 mg (0.04 mmol) of NiCl$_2$•DME, 21.1 mg (0.04 mmol) of **L1e**, 12.4 mg (0.05 mmol) of DMBP and 70.4 mg (0.4 mmol) of K_2HPO_4 to afford 12.2 mg (20% yield, E/Z isomers) of **7** as a colorless oil. 74% recovery of **7** and 100% MS versus **2a** was detected by ^{1}H NMR crude spectrum using TMSPh as an internal standard. IR (neat, cm^{-1}): 3001, 2951, 1721, 1609, 1511. HRMS (ESI, m/z): calcd for $C_{19}H_{21}O_3$ (M+H$^+$) m/z: 297.1491, found: 297.1493.

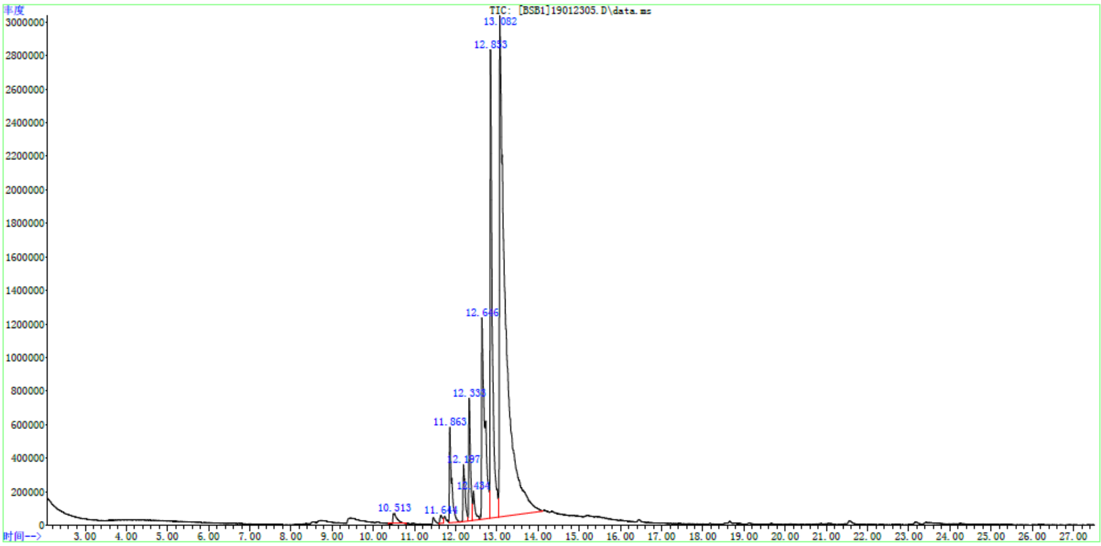

Scan 1775 (13.089 min)

Time

Scan 1775 (13.089 min)

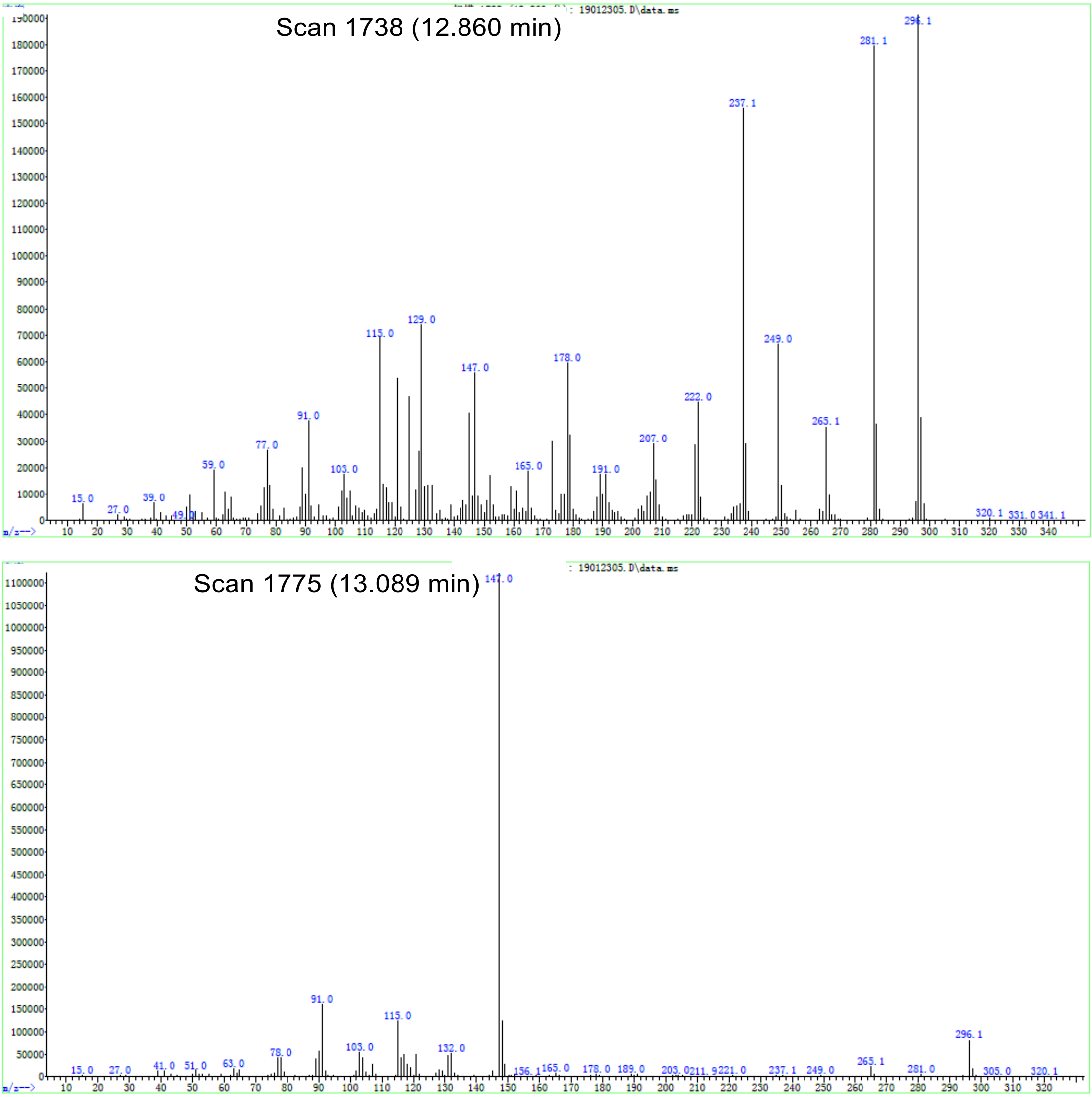

Supplementary Figure 149. GC-MS spectra of **8**

Bromine radical trapping Experiment

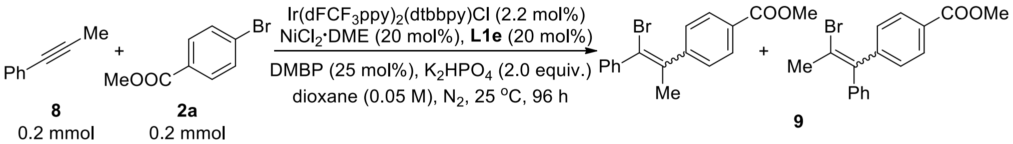

Supplementary Figure 150. Bromine radical trapping Experiment.

9 was obtained by following modified Standard Conditions B using 26.2 mg (0.2 mmol) of **8**[20], 43.1 mg (0.2 mmol) of **2a**, 5.0 mg (0.0044 mmol) of Ir(dFCF$_3$ppy)$_2$(dtbbpy)Cl, 8.6 mg (0.04 mmol) of NiCl$_2$•DME, 19.3 mg (0.04 mmol) of **L1e**, 11.8 mg (0.05 mmol) of DMBP and 69.5 mg (0.4 mmol) of K_2HPO_4 to afford 15.9 mg (24% yield, mixture of four isomers) of **9** as a yellow oil. 100% MS versus **2a** was detected by ^{1}H NMR crude spectrum using TMSPh as an internal standard. Recovery of **8** was undetermined since **8** was easily vaporized under high vacuum. HRMS (ESI, m/z): calcd for $C_{17}H_{16}BrO_2$ (M+H$^+$) m/z: 331.0334, found: 331.0326.

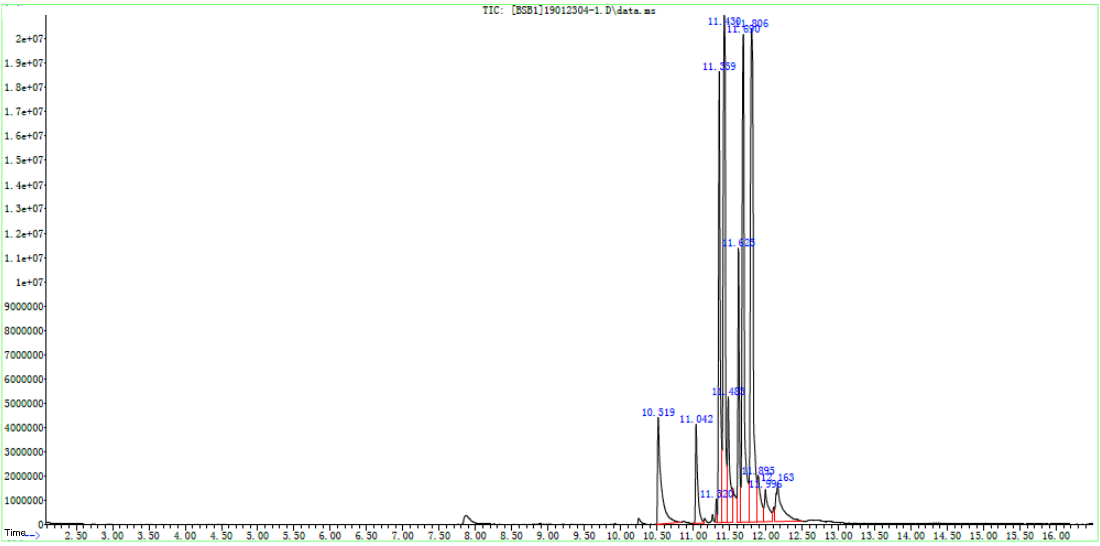
TIC: [BSB1]19012304-1.D\data.ms
10.519
11.042
11.559
11.625
11.488
11.895
12.163
Time-->

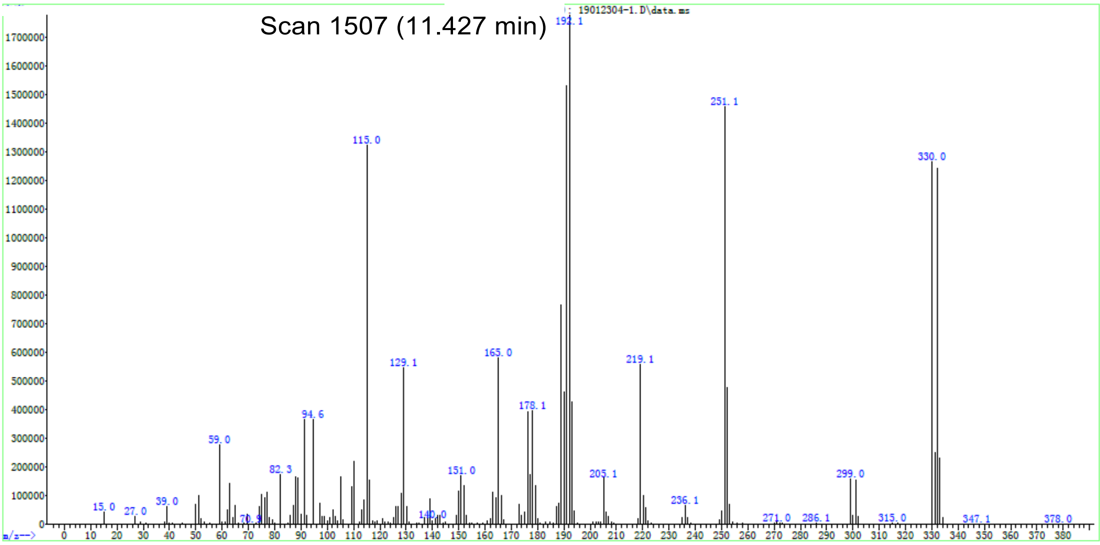
Scan 1507 (11.427 min)
19012304-1.D\data.ms
115.0
129.1
165.0
178.1
219.1
251.1
330.0
299.0
94.6
59.0
82.3
151.0
205.1
236.1
m/z-->

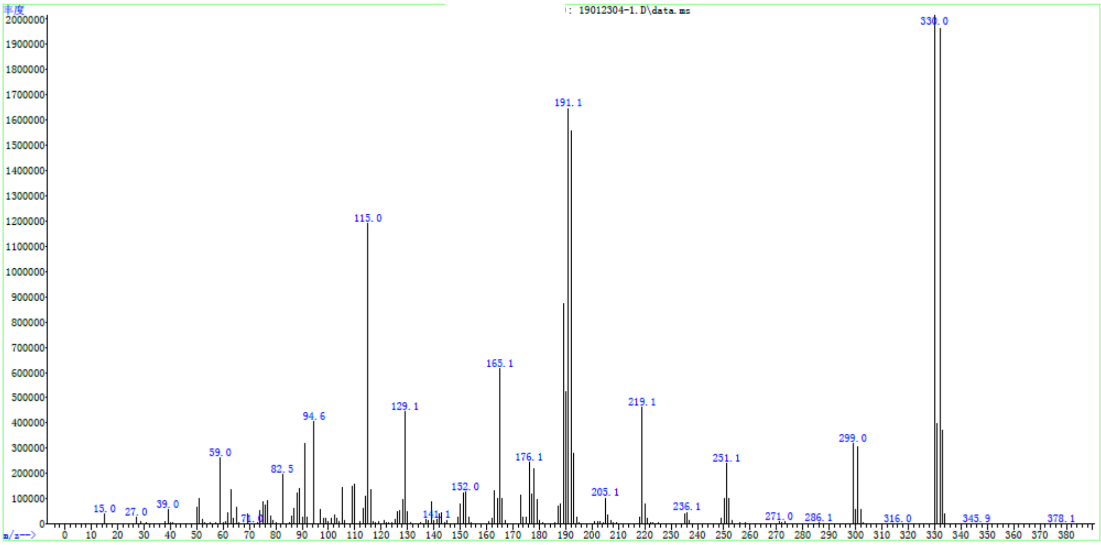
丰度
19012304-1.D\data.ms
330.0
191.1
115.0
165.1
129.1
219.1
94.6
299.0
251.1
176.1
59.0
82.5
152.0
205.1
236.1
m/z-->

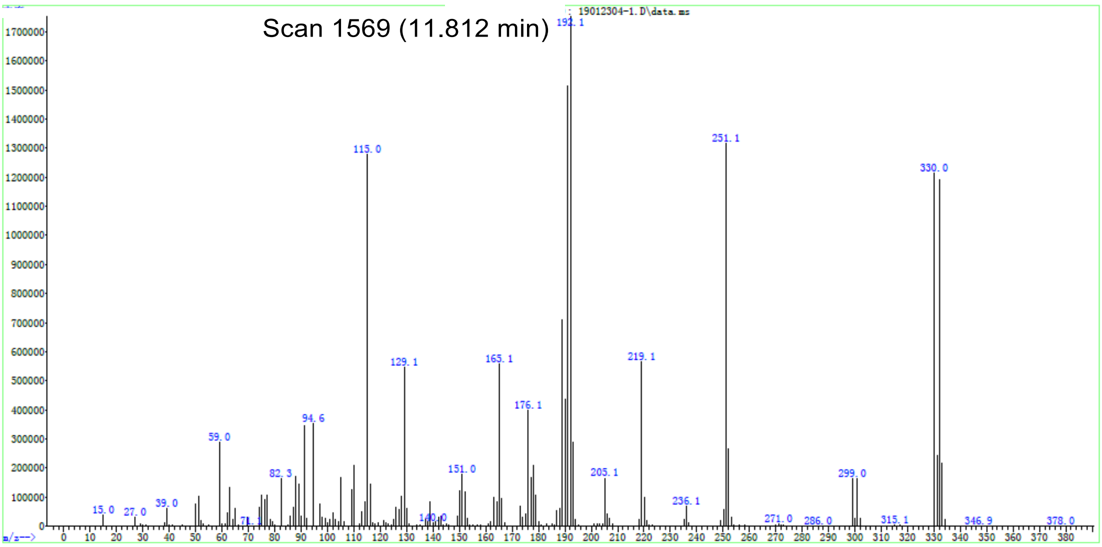

Supplementary Figure 151. GC-MS spectra of **9**

Kinetic Isotope Effect Experiment

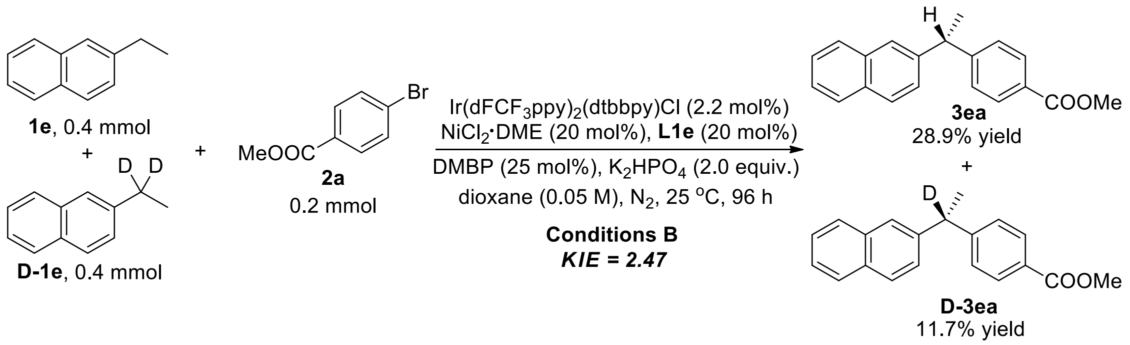

Supplementary Figure 152. Kinetic Isotope Effect Experiment.

The kinetic isotope effect experiment was carried out by following Standard Conditions B using 62.7 mg (0.4 mmol, 2.0 equiv.) of **1e**, 62.6 mg (0.4 mmol, 2.0 equiv.) of deuterated **1e**[21] (**D-1e**), 42.4 mg (0.2 mmol, 1.0 equiv.) of **2a**, 4.2 mg (0.0044 mmol, 2.2 mol%) of Ir(dFCF$_3$ppy)$_2$(dtbbpy)Cl, 8.6 mg (0.04 mmol, 20 mol%) of NiCl$_2$•DME, 19.2 mg (0.04 mmol, 20 mol%) of **L1e**, 12.1 mg (0.05 mmol, 25mol%) of DMBP and 69.4 mg (0.4 mmol, 2.0 equiv.) of K_2HPO_4. The yield of **3ea** was determined by ^{1}H NMR using 10 µL of trimethyl(phenyl)silane (TMSPh) as an internal standard. The yield of deuterated **3ea** (**D-3ea**) was determined by ^{2}D NMR using $CHCl_3$ as solvent and 10 µL of $CDCl_3$ as an internal standard.

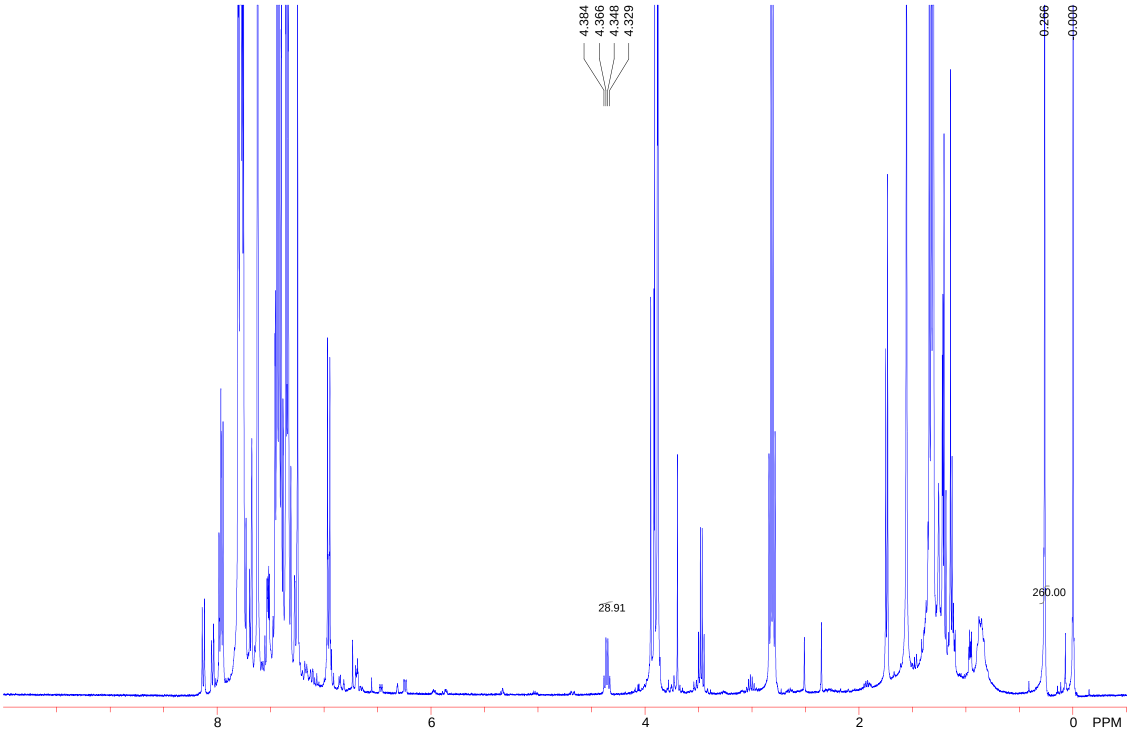

Supplementary Figure 153. ^{1}H NMR crude spectrum of the kinetic isotope effect experiment.

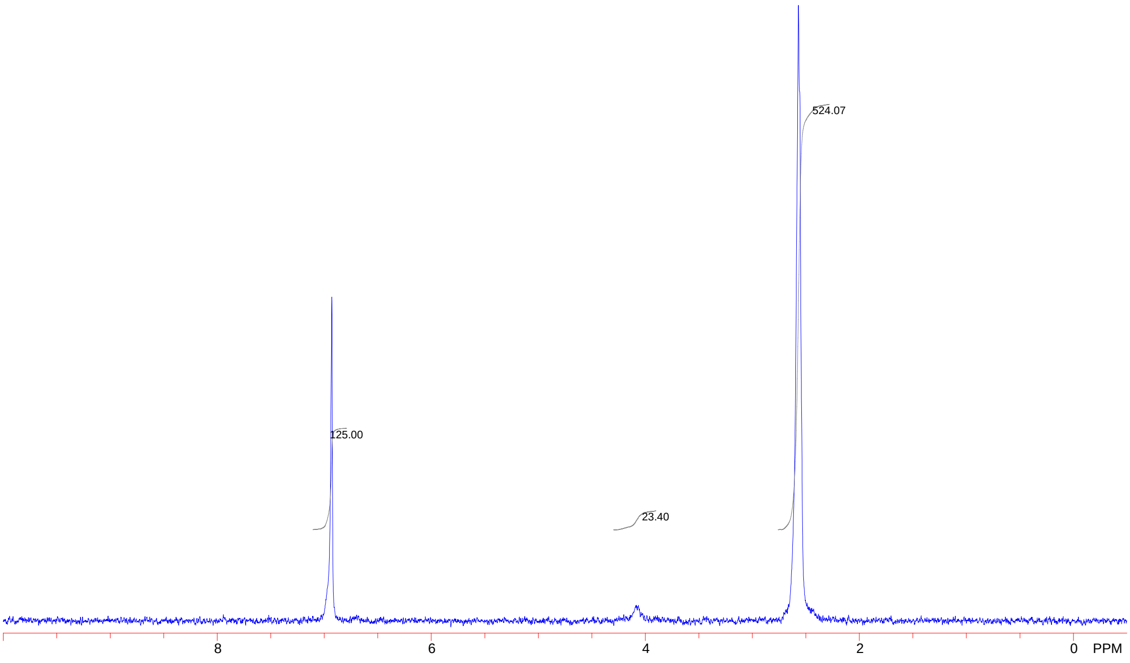

Supplementary Figure 154. ^{2}D NMR crude spectrum of the kinetic isotope effect experiment.

Catalytic Active Species Experiment

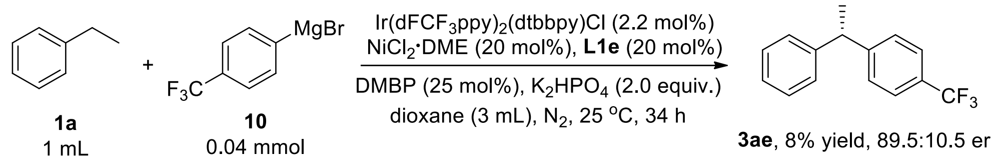

Supplementary Figure 155. Catalytic Active Species Experiment.

The catalytic active species experiment was carried out by following modified conditions of Standard Conditions A. To a 20 mL vial with a stir bar was added 19.9 mg (0.04 mmol, 20 mol%) of **L1e**, 8.9 mg (0.04 mmol, 20 mol%) of $NiCl_2$•DME and 1 mL of dioxane in a N_2-filled glovebox. Reaction was stirred at 50 °C for 30 minutes before cooled to room temperature. Arylmagnesium bromide **10** (0.1 M in THF, 0.4 mL, 0.04 mmol) was added and the reaction was stirred for additional 1 h at room temperature before the solvent was moved out under vaccum. 3 mL of dioxane, 1 mL of ethyl benzene **1a**, 4.3 mg (0.0044 mmol, 2.2 mol%) of Ir(dFCF$_3$ppy)$_2$(dtbbpy)Cl, 12.1 mg (0.05 mmol, 25 mol%) of DMBP and 70.4 mg (0.4 mmol, 2.0 equiv.) of K_2HPO_4 were added consistently. The vial was sealed with a Teflon cap and then allowed to remove from the glovebox. The reaction was stirred at 600 rpm under the irradiation of 8W blue LEDs at room temperature (25 °C) for 34 h. The reaction was quenched by adding Et_2O, filtered through a short pad of silica and eluted with Et_2O. The solution was concentrated under reduced pressure and **3ae** was obtained in 8% yield determined by ^{1}H NMR using TMSPh as an internal standard. The enantiomeric ratio of **3ae** was 89.5:10.5 determined by chiral HPLC.

Supplementary References

1 Kermagoret, A. & Braunstein, P. Mono- and Dinuclear Nickel Complexes with Phosphino-, Phosphinito-, and Phosphonitopyridine Ligands: Synthesis, Structures, and Catalytic Oligomerization of Ethylene. *Organometallics* **27**, 88-89 (2008).

2 Tellis, J. C., Primer, D. N., & Molander, G. A. Single-electron transmetalation in organoboron cross-coupling by photoredox/nickel dual catalysis. *Science* **345**, 433-436 (2014).

3 Rohe, S. *et al.* Hydrogen Atom Transfer Reactions via Photoredox Catalyzed Chlorine Atom Generation. *Angew. Chem. Int. Ed.* **57**, 15664-15669 (2018).

4 Hao, X. -Q. *et al.* Biimidazoline ligands for palladium-catalyzed asymmetric allylic alkylation. *Tetrahedron: Asymmetry* **26**, 1360-1368 (2015).

5 Boland, N. A. *et al.* Preparation of enantiopure biimidazoline ligands and their use in asymmetric catalysis. *Org. Biomol. Chem.* **2**, 1995-2002 **(**2004).

6 Poremba, K. E. *et al.* Nickel-Catalyzed Asymmetric Reductive Cross-Coupling To Access 1,1-Diarylalkanes. *J. Am. Chem. Soc.* **139**, 5684-5687 (2017).

7 Yasuda, H. *et al.* Effects of bulky ligands and water in Pd-catalyzed oxidative carbonylation of phenol. *J. Mol. Catal. A: Chemical* **236**, 149-155 (2005).

8 Müller, D. *et al.* C_2-Symmetric 4,4′,5,5′-Tetrahydrobi(oxazoles) and 4,4′,5,5′-Tetrahydro-2,2′-methylenebis[oxazoles] as Chiral Ligands for Enantioselective Catalysis Preliminary Communication. *Helvetica Chim Acta* **74**, 232-239 (1991).

9 Frauenlob, R. *et al.* Rapid, *in situ* synthesis of bidentate ligands: chromatography-free generation of catalyst libraries. *Org. Biomol. Chem.* **9**, 6934-6937 (2011).

10 De Crisci, A.G. *et al.* Chemoselective Oxidation of Polyols with Chiral Palladium Catalysts. *Organometallics* **32**, 2257-2266 (2013).

11 Holder, J. C. *et al.* Mechanism and Enantioselectivity in Palladium-Catalyzed Conjugate Addition of Arylboronic Acids to β-Substituted Cyclic Enones: Insights from Computation and Experiment. *J. Am. Chem. Soc.* **135**, 14996-15007 (2013).

12 Wang, T. *et al.* Chiral CNN Pincer Palladium(II) Complexes with 2-Aryl-6-(oxazolinyl)pyridine Ligands: Synthesis, Characterization, and Application to Enantioselective Allylation of Isatins and Suzuki–Miyaura Coupling Reaction. *Organometallics* **33**, 194-205 (2014).

13 Lu, P., Ji, C. -L., & Lu, Z. Nickel-Catalyzed C−H Heteroarylation of Chiral Oxazolines *Asian J. Org. Chem.* **7**, 542-544 (2018).

14 Tang, Y., Liu, J., & Tao, W. Patent Num: WO 2016/058559 Al.
15 Chen, J. -H. *et al.* Cobalt-Catalyzed Asymmetric Hydrogenation of 1,1-Diarylethenes. *Org. Lett.* **18**, 1594-1597 (2016).
16 Gutierrez, O. et al. Nickel-Catalyzed Cross-Coupling of Photoredox-Generated Radicals: Uncovering a General Manifold for Stereoconvergence in Nickel-Catalyzed Cross-Couplings. *J. Am. Chem. Soc.* **137**, 4896-4899 (2015).
17 Wang, X. *et al.* Highly Enantioselective Hydrogenation of Styrenes Directed by 2′-Hydroxyl Groups. *Org. Lett.* **13**, 1881-1883 (2011).
18 Zhou, Q. *et al.* Nickel-Catalyzed Cross-Couplings of Benzylic Pivalates with Arylboroxines: Stereospecific Formation of Diarylalkanes and Triarylmethanes. *J. Am. Chem. Soc.* **135**, 3307-3310 (2013).
19 Li, M. -B., Tang, X. -L., & Tiana, S. -K. Cross-Coupling of Grignard Reagents with Sulfonyl-Activated sp3 Carbon−Nitrogen Bonds. *Adv. Synth. Catal.* **353**, 1980-1984 (2011).
20 Stuart, D. R. *et al.* Indole Synthesis via Rhodium Catalyzed Oxidative Coupling of Acetanilides and Internal Alkynes. *J. Am. Chem. Soc.* **130**, 16474-16475 (2008).
21 Mattson, R. J. & Sloan, C. P. Ortho-directed lithiation in .pi.-deficient diazinyl heterocycles. *J. Org. Chem.* **55**, 3410-3412 (1990).
